# Rhizoaspergillin A and Rhizoaspergillinol A, including a Unique Orsellinic Acid–Ribose–Pyridazinone-*N*-Oxide Hybrid, from the Mangrove Endophytic Fungus *Aspergillus* sp. A1E3

**DOI:** 10.3390/md21110598

**Published:** 2023-11-19

**Authors:** Binbin Wu, Chenglong Xu, Jianjun Chen, Guangying Chen

**Affiliations:** 1 Key Laboratory of Tropical Medicinal Resource Chemistry of Ministry of Education, College of Chemistry and Chemical Engineering, Hainan Normal University, Haikou 571158, China; binbwu168@163.com; 2 Guangdong Provincial Key Laboratory of New Drug Screening, School of Pharmaceutical Sciences, Southern Medical University, 1838 Guangzhou Avenue North, Guangzhou 510515, China; xxuchenglong@163.com

**Keywords:** *Aspergillus*, pyridazinone-*N*-oxide hybrid, sterigmatocystin, antitumor, cell cycle arrest

## Abstract

Two new compounds, named rhizoaspergillin A (**1**) and rhizoaspergillinol A (**2**), were isolated from the mangrove endophytic fungus *Aspergillus* sp. A1E3, associated with the fruit of *Rhizophora mucronata*, together with averufanin (**3**). The planar structures and absolute configurations of rhizoaspergillinol A (**2**) and averufanin (**3**) were established by extensive NMR investigations and quantum-chemical electronic circular dichroism (ECD) calculations. Most notably, the constitution and absolute configuration of rhizoaspergillin A (**1**) were unambiguously determined by single-crystal X-ray diffraction analysis of its *tri*-pivaloyl derivative **4**, conducted with Cu Kα radiation, whereas those of averufanin (**3**) were first clarified by quantum-chemical ECD calculations. Rhizoaspergillin A is the first orsellinic acid–ribose–pyridazinone-*N*-oxide hybrid containing a unique β-oxo-2,3-dihydropyridazine 1-oxide moiety, whereas rhizoaspergillinol A (**2**) and averufanin (**3**) are sterigmatocystin and anthraquinone derivatives, respectively. From the perspective of biosynthesis, rhizoaspergillin A (**1**) could be originated from the combined assembly of three building blocks, viz., orsellinic acid, β-D-ribofuranose, and *L*-glutamine. It is an unprecedented alkaloid-*N*-oxide involving biosynthetic pathways of polyketides, pentose, and amino acids. In addition, rhizoaspergillinol A (**2**) exhibited potent antiproliferative activity against four cancer cell lines. It could dose-dependently induce G2/M phase arrest in HepG2 cells.

## 1. Introduction

Pyridazines and pyridazinones are rare in nature but are common building blocks for heterocyclic organic synthesis [1,2,3,4]. Maleic hydrazide, i.e., 1,2-dihydro-3,6-pyradizinedione, is a synthesized selective herbicide and temporary plant growth regulator commonly used to prevent sprouting of potato tubers, onions, garlic, and radishes, etc., during storage. It can also inhibit crop growth and extend flowering periods [5]. Pyridaben, another example of a pyridazinone, is a broad-spectrum and contact killing acaricide. It is a mitochondrial electron transport inhibitor (METI) acaricide that promotes the formation of damaging oxygen and nitrogen radicals [6,7,8]. Pyridazine *N*-oxides are photoactivatable O(^3^P) precursors for applications in organic synthesis and chemical biology [9], whereas pyridazinone *N*-oxides are relatively stable. To the best of our knowledge, all the pyridazinone *N*-oxides are synthetic compounds. To date, no natural products of pyridazinone *N*-oxides have been reported.

**Figure 1:**
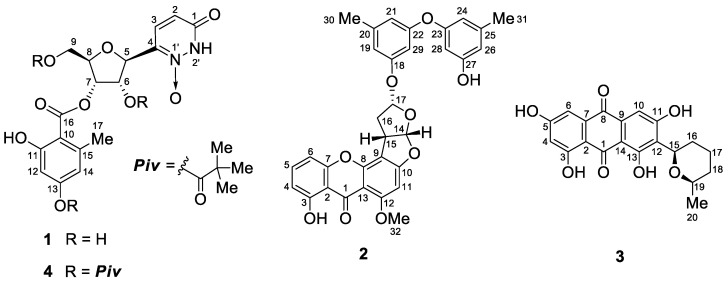
Structures of compounds **1**–**4**.

Mangrove endophytic fungi of the genus *Aspergillus* can produce structurally unique metabolites with diverse bioactivities [10,11,12,13,14,15,16,17,18,19]. In order to search for bioactive natural compounds with new structures, two new compounds, named rhizoaspergillin A (**1**) and rhizoaspergillinol A (**2**), were isolated from the mangrove endophytic fungus *Aspergillus* sp. A1E3, associated with the fruit of *Rhizophora mucronata*, together with averufanin (**3**) [20,21,22,23,24,25] (Figure 1). To our knowledge, rhizoaspergillin A (**1**) is the first alkaloid-*N*-oxide featuring the presence of an unprecedented orsellinic acid–ribose–pyridazinone-*N*-oxide hybrid scaffold containing a unique β-oxo-2,3-dihydropyridazine 1-oxide moiety, whereas rhizoaspergillinol A (**2**) and averufanin (**3**) are sterigmatocystin and anthraquinone derivatives, respectively. Herein, we report the isolation and structural identification of rhizoaspergillin A (**1**) and rhizoaspergillinol A (**2**), along with clarification of the absolute configuration of averufanin (**3**). The antiproliferative activities of compounds **1**–**3** were also evaluated against four cancer cell lines.

## 2. Results and Discussion

Rhizoaspergillin A (**1**) was obtained as an amorphous powder. The molecular formula C_17_H_19_N_2_O_9_ with ten degrees of unsaturation was determined by HR-ESIMS (*m*/*z*: calcd: 395.1085; found: 395.1084 [M + H]^+^). The ^13^C-NMR spectroscopic data and DEPT 135 experimental results for **1** revealed the presence of a methyl group, an oxygenated methylene group, eight methine groups (four oxygenated and four olefinic), and seven nonprotonated carbons (two carbonyl and five olefinic). According to the 1D and 2D NMR spectroscopic data for **1**, nine degrees of unsaturation are due to a carbon–carbon double bond, a carbon–nitrogen double bond, a tetrahydrofuran ring, an amide group, and a benzoate group. Thus, a pyridazinone ring should exist in the molecule.

**Figure 2:**
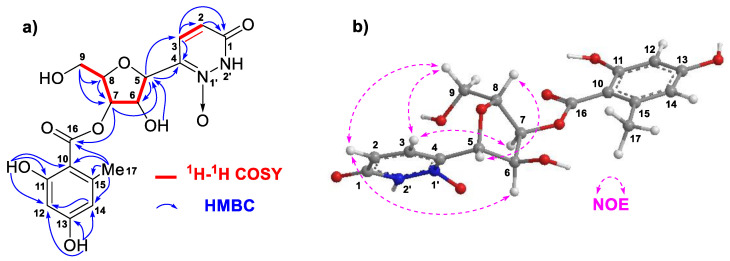
(**a**) Selected ^1^H–^1^H COSY and HMBC correlations for **1**. (**b**) Diagnostic NOE interactions for **1**.

The presence of a β-D-ribofuranose unit, being characterized by the corresponding NMR spectroscopic data [δ_H_ 5.96 (d, *J* = 6.4 Hz, H-5), 4.46 (dd, *J* = 11.0, 5.8 Hz, H-6), 6.00 (d, *J* = 5.6 Hz, 6-OH), 5.45 (dd, *J* = 5.2, 2.8 Hz, H-7), 4.26 (dd, *J* = 5.4, 2.8 Hz, H-8), 3.74 (br s, H_2_-9), 5.38 (t, *J* = 4.8 Hz, 9-OH); δ_C_ 87.8 (CH, C-5), 72.0 (CH, C-6), 74.0 (CH, C-7), 83.0 (CH, C-8), 61.2 (CH_2_, C-9)] (Table 1), was corroborated by ^1^H–^1^H COSY correlations between H-5/H-6, H-6/H-7, H-7/H-8, and H-8/H_2_-9 (Figure 2a). The existence of an orsellinic acid moiety, i.e., a 2,4-dihydroxy-6-methylbenzoic acid unit, being evidenced by the corresponding NMR spectroscopic data [δ_H_ 6.24 (d, *J* = 2.0 Hz, H-12), 6.28 (d, *J* = 2.0 Hz, H-14), 2.47 (s, H_3_-17), 10.85 (s, 11-OH), 10.17 (s, 13-OH); δ_C_ 106.3 (C, C-10), 162.6 (C, C-11), 100.9 (CH, C-12), 162.1 (C, C-13), 111.2 (CH, C-14), 142.4 (C, C-15), 169.1 (C, C-16), 23.1 (CH_3_, C-17)] (Table 1), was confirmed by HMBC correlations between OH-11/C-10, OH-11/C-11, OH-11/C-12, OH-13/C-12, OH-13/C-13, OH-13/C-14, H_3_-17/C-10, H_3_-17/C-14, H_3_-17/C-15, and H_3_-17/C-16. Strong HMBC cross-peaks from protons of Me-17 (δ_H_ 2.47, s) to the nonprotonated C-15 (δ_C_ 142.4) placed it at C-15. Most notably, the key HMBC cross-peak from H-7 to the carbonyl C-16 connected the β-D-ribofuranose unit and the orsellinic acid moiety through the C-7–*O*–C-16 bond (Figure 2a).

The presence of a pyridazinone ring, being characterized by the corresponding NMR spectroscopic data [*δ*_H_ 11.40 (s, H-2′), 5.78 (d, *J* = 8.0 Hz, H-2), 7.96 (d, *J* = 8.0 Hz, H-3); *δ*_C_ 163.3 (C, C-1), 102.6 (CH, C-2), 140.7 (CH, C-3), 151.1 (C, C-4)] (Table 1), was corroborated by the ^1^H–^1^H COSY cross-peak between H-2/H-3 and HMBC correlations between H-2/C-1, H-3/C-1, H-3/C-2, and H-3/C-4 (Figure 2a). The Key HMBC correlation from H-5 to C-4 connected the β-D-ribofuranose unit and the above pyridazinone ring through the C-4–C-5 bond. In addition, the existence of a nitrogen–oxygen bond could be inferred by the molecular formula of **1** to be loaded on N_1′_. Taken together, the planar structure of **1** was elucidated as shown (Figure 2a). 

**Figure 3:**
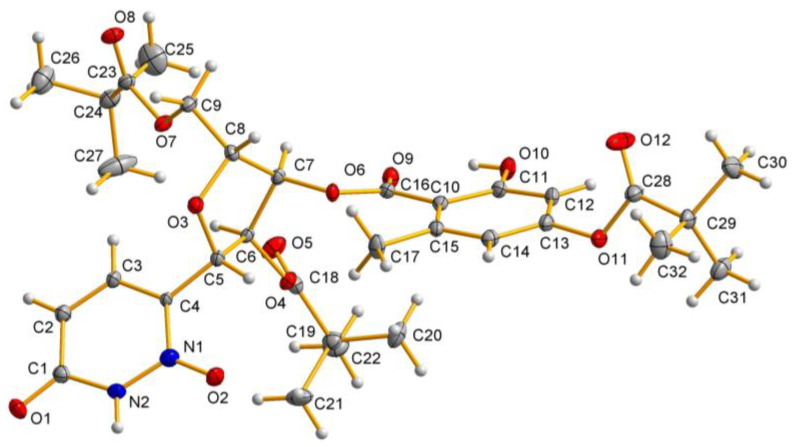
Oak Ridge Thermal-Ellipsoid Plot Program (ORTEP) illustration of the X-ray structure of **4** (Ellipsoids are given at the 30% probability level, Cu Kα).

The relative configuration of **1** was determined by NOE interactions (Figure 2b). Those between H-2/H-6, H-2/H_2_-9, H_2_-9/H-3, and H-3/H-7 revealed their cofacial relationships and were arbitrarily assigned as the α-oriented H-6 and H-7, whereas the diagnostic NOE interaction between H-5 and H-8 assigned their cofacial β-orientation. In order to reconfirm the constitution of **1** and establish its absolute configuration, single-crystal X-ray diffraction analysis was taken into account. However, it is impossible to obtain suitable crystals of **1** due to its poor solubility. Thus, derivatization reaction products were considered. Compound **1** was acylated by pivaloyl chloride to afford **4** (a *tri*-pivaloyl derivative of **1**, Figure 1). Suitable crystals of **4** were obtained in MeOH after considerable effort. Finally, the constitution and absolute configuration of **1** were established by single-crystal X-ray diffraction analysis of **4**, conducted with Cu Kα radiation [Flack parameter—0.12(9)] (Figure 3, CCDC 2291154). The absolute configuration of **1**, named rhizoaspergillin A, was unambiguously determined to be (5*S*,6*S*,7*S*,8*R*). To the best of our knowledge, rhizoaspergillin A is the first reported orsellinic acid–ribose–pyridazinone-*N*-oxide hybrid.

Rhizoaspergillinol A (**2**) was isolated as a light white amorphous powder. The molecular formula C_32_H_26_O_9_ was determined by the positive HR-ESIMS ion at *m*/*z* 555.1644 (calcd for [M + H]^+^, 555.1650), indicating twenty degrees of unsaturation. According to ^1^H- and ^13^C-NMR spectroscopic data of **2** (Table 2), thirteen degrees of unsaturation are due to a keto-carbonyl function and twelve carbon–carbon double bonds. Therefore, the molecule has to be heptacyclic. The ^13^C-NMR spectroscopic data and DEPT 135 experimental results for **2** revealed the presence of three methyl groups (a methoxy and two tertiary), a methylene group, thirteen methine groups (twelve olefinic and one oxygenated), and fifteen nonprotonated carbons (one carbonyl, five olefinic, and nine oxygenated). 

**Figure 4:**
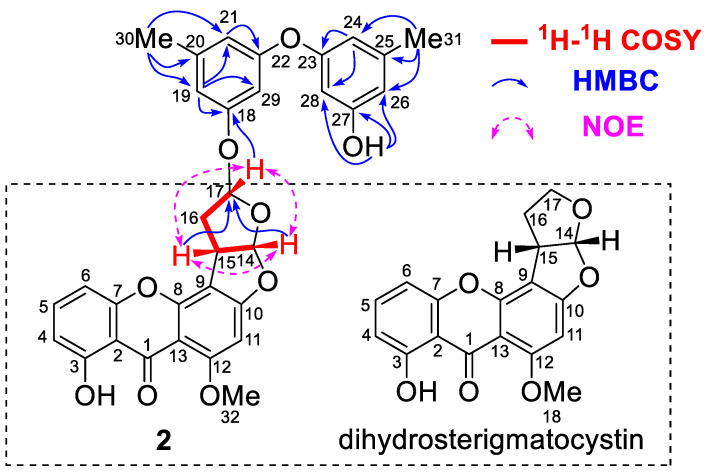
Selected ^1^H–^1^H COSY cross-peaks, HMBC correlations, and NOE interactions for **2**.

The NMR spectroscopic data for **2** resembled those of dihydrosterigmatocystin [26,27], except for the presence of an additional 5,5’-oxybis(3-methylphenol) moiety, namely diorcinol [28,29,30], being characterized by the corresponding NMR spectroscopic data [*δ*_H_ 6.67 (br s, H-19), 6.51 (br s, H-21), 6.400 (d, *J* = 2.0 Hz, H-24), 6.399 (d, *J* = 2.0 Hz, H-26), 6.29 (t, *J* = 2.0 Hz, H-28), 6.56 (d, *J* = 2.0 Hz, H-29), 2.30 (s, H_3_-30), 2.27 (s, H_3_-31), 4.71 (br s, 27-OH); *δ*_C_ 157.6 (C, C-18), 112.0 (CH, C-19), 140.9 (C, C-20), 114.0 (CH, C-21), 157.8 (C, C-22), 158.2 (C, C-23), 111.4 (CH, C-24), 141.0 (C, C-25), 111.1 (CH, C-26), 156.5 (C, C-27), 103.3 (CH, C-28), 105.1 (CH, C-29), 21.5 (Me-30), 21.7 (Me-31)]. The existence of the diorcinol moiety was corroborated by HMBC correlations between H-19/C-18, H-19/C-21, H-19/C-29, H_3_-30/C-19, H_3_-30/C-20, H_3_-30/C-21, H-21/C-22, H-24/C-23, H-24/C-28, H_3_-31/C-24, H_3_-31/C-25, H_3_-31/C-26, 27-OH/C-26, 27-OH/C-27, and 27-OH/C-28. HMBC cross-peaks from protons of Me-30 (*δ*_H_ 2.30, s) to the nonprotonated C-20 (*δ*_C_ 140.9) and those from protons of Me-31 (*δ*_H_ 2.27, s) to the nonprotonated C-25 (*δ*_C_ 141.0) placed Me-30 at C-20 and Me-31 at C-25, respectively. In addition, the key HMBC correlation from H-17 (*δ*_H_ 5.89, t, *J* = 4.9 Hz) to the nonprotonated C-18 (*δ*_C_ 157.6, C) connected the dihydrosterigmatocystin unit and the diorcinol moiety through the C-17–*O*–C-18 bond. Therefore, the constitution of **2** was elucidated as shown (Figure 4).

**Figure 5:**
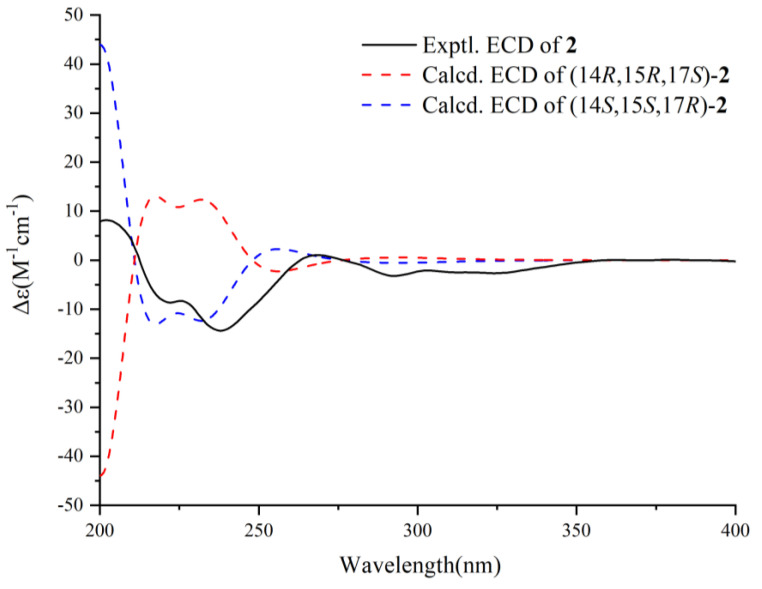
Experimental and calculated ECD curves for **2**.

The relative configuration of **2** was determined by NOE interactions (Figure 4). Those between H-17/H-14 and H-17/H-15 revealed their cofacial relationship. Based on the previously reported absolute configuration of dihydrosterigmatocystin, the absolute configuration of C-17 in **2** was thereby assigned as *R*. In addition, the absolute configuration of **2** was reconfirmed by quantum-chemical electronic circular dichroism (ECD) calculations (Figure S1). The calculated ECD curve of (14*S*,15*S*,17*R*)-**2** showed good agreement with that of the experimental curve of **2** (Figure 5), whereas that of (14*R*,15*R*,17*S*)-**2** exhibited mirrored Cotton effects. Therefore, the absolute configuration of **2**, named rhizoaspergillinol A, was concluded to be (14*S*,15*S*,17*R*).

**Figure 6:**
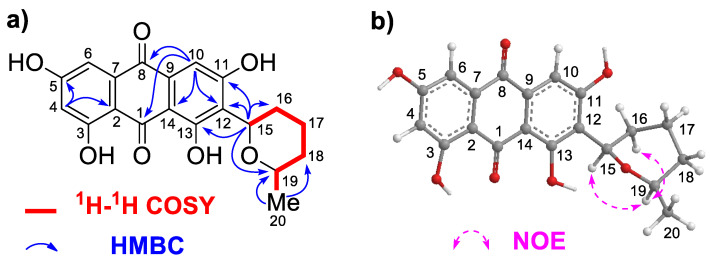
(**a**) Selected ^1^H–^1^H COSY and HMBC correlations for **3**. (**b**) Diagnostic NOE interactions for **3**.

Compound **3** was obtained as amorphous powder. The molecular formula C_20_H_18_O_7_ was established by the positive HR-ESIMS ion at *m*/*z* 371.1136 (calcd for [M + H]^+^, 371.1125), indicating twelve degrees of unsaturation. According to the NMR spectroscopic data for **3** (Table 3), eight degrees of unsaturation are due to two keto-carbonyl function, six carbon–carbon double bonds. Therefore, the molecule has to be tetracyclic. The presence of a 3,5,11,13-tetrahydroxyanthraquinone core, being characterized by the corresponding NMR spectroscopic data [δ_H_ 6.51 (d, *J* =1.2 Hz, H-4), 7.03 (br s, H-6), 7.00 (s, H-10); δ_C_ 188.5 (C, C-1), 108.4 (C, C-2), 165.5 (C, C-3), 108.0 (CH, C-4), 164.3 (C, C-5), 109.0 (CH, C-6), 134.7 (C, C-7), 181.0 (C, C-8), 133.1 (C, C-9), 109.0 (CH, C-10), 161.5 (C, C-11), 119.8 (C, C-12), 162.9 (C, C-13), 108.2 (C, C-14)], was confirmed by HMBC correlations between H-4/C-2, H-4/C-5, H-10/C-1, H-10/C-8, H-10/C-12, and H-10/C-14. The presence of a 20-methyltetrahydropyran moiety was confirmed by ^1^H–^1^H COSY cross-peaks between H-15/H_2_-16, H_2_-16/H_2_-17, H_2_-17/H_2_-18, H_2_-18/H-19, and H-19/H_3_-20 and HMBC correlations between H-15/C-11, H-15/C-12, H-15/C-13, H-15/C-16, H-15/C-19, H_3_-20/C-18, and H_3_-20/C-19. HMBC correlations from H-15 to C-11, C-12, and C-13 connected the 3,5,11,13-tetrahydroxyanthraquinone core and the 20-methyltetrahydropyran moiety through the C-12–C-15 bond (Figure 6a).

**Figure 7:**
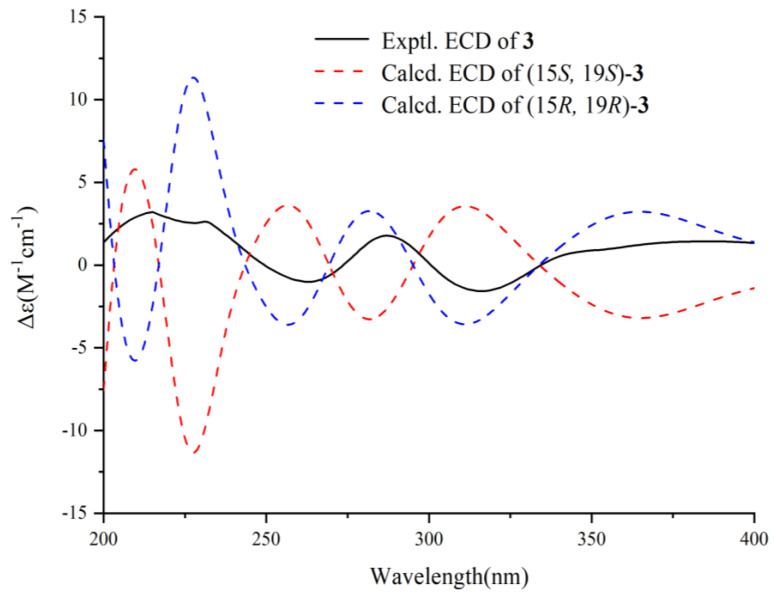
Experimental and calculated ECD curves for **3**.

The strong NOE interaction between H-15 and H-19 revealed their cofacial relationship (Figure 6b). The NMR spectroscopic data for **3** were the same as those of averufanin [21,22,25]. However, two absolute configurations, viz., (15*R*,19*R*) and (15*S*,19*S*), were confused in the literature [20,21,22,23,24,25]. In order to clarify the absolute configuration of **3**, quantum-chemical ECD calculations were employed (Figure S2). The calculated ECD curve of (15*R*,19*R*)-**3** showed good agreement with that of the experimental curve of **3**, whereas that of (15*S*,19*S*)-**3** exhibited mirrored Cotton effects (Figure 7). Therefore, the absolute configuration of **3**, i.e., averufanin, was concluded to be (15*R*,19*R*).

**Scheme 1:**
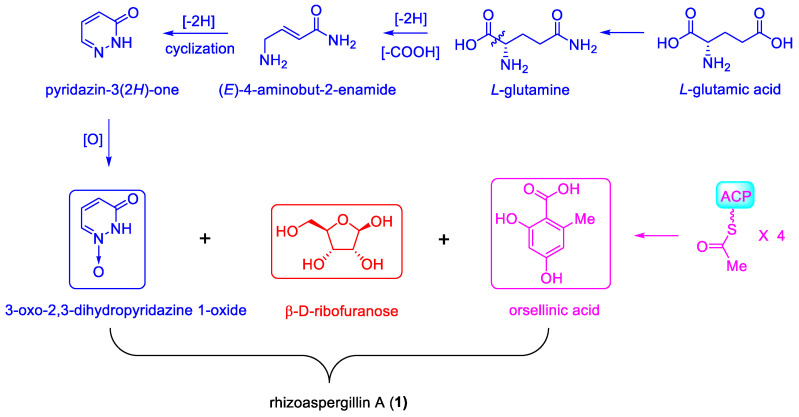
Proposed biosynthetic origin of rhizoaspergillin A (**1**).

The biosynthetic origin of **1** could be traced back to three building blocks, viz., 3-oxo-2,3-dihydropyridazine 1-oxide, β-D-ribofuranose, and orsellinic acid, among which the 3-oxo-2,3-dihydropyridazine 1-oxide moiety could be originated from one unit of *L*-glutamic acid, whereas the orsellinic acid unit could be biosynthesized from four units of acetyl coenzyme A (Scheme 1). The amidation of *L*-glutamic acid could generate *L*-glutamine, of which the decarboxylation and dehydrogenation would produce the intermediate (*E*)-4-aminobut-2-enamide. Subsequent cyclization and dehydrogenation could afford the heterocyclic intermediate pyridazin-3(2*H*)-one, of which the oxidation at the nitrogen atom would yield the crucial building block 3-oxo-2,3-dihydropyridazine 1-oxide. The dehydration of the anomeric center of the β-D-ribofuranose motif could produce a carbocation at C-1, of which nucleophilic attack at C-6 of the 3-oxo-2,3-dihydropyridazine 1-oxide moiety would generate a new carbon–carbon bond between C-1 of the β-D-ribofuranose motif and C-6 of the 3-oxo-2,3-dihydropyridazine 1-oxide moiety [31,32,33,34,35]. Finally, the esterification between 3-OH of the β-D-ribofuranose motif and the 7-carboxyl group of the orsellinic acid unit could produce rhizoaspergillin A (**1**). (Scheme 1)

**Figure 8:**
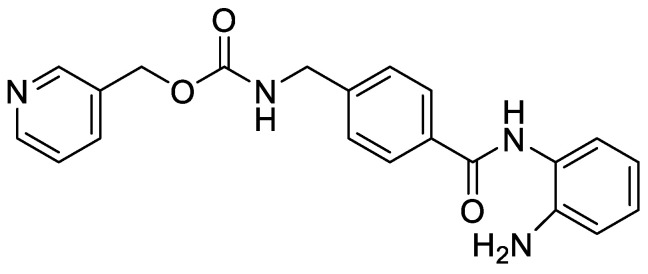
The structure of MS-275 (Entinostat).

**Figure 9:**
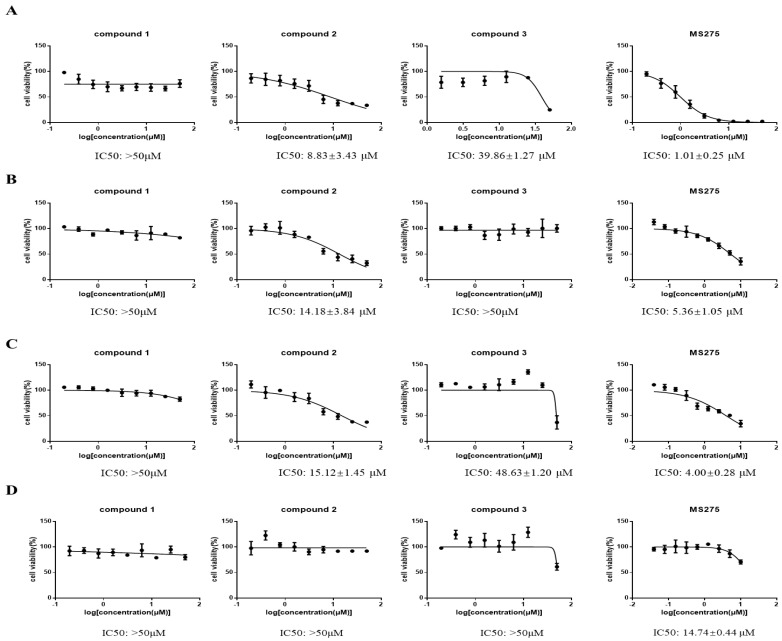
(**A**) MTT curve graph of **1**–**3** and MS-275 against HepG2 cells. (**B**) MTT curve graph of **1**–**3** and MS-275 against LLC cells. (**C**) MTT curve graph of **1**–**3** and MS-275 against B16-F10 cells. (**D**) MTT curve graph of **1**–**3** and MS-275 against MCF7 cells.

The antiproliferative activity of **1**–**3** against four cancer cell lines were evaluated by using the standard MTT assay, with MS-275 (Entinostat, Figure 8) as the positive control [36]. MS-275 is a known histone deacetylase inhibitor as an anticancer agent. As summarized in Table 4 and Figure 9, **2** is the most potent one among the three compounds, with IC_50_ values of 8.83, 14.18, and 15.12 μΜ against HepG2, LLC, and B16-F10 cancer cell lines, respectively. Compounds **1** and **3** showed lower activities with IC_50_ values around or greater than 50.0 μΜ.

**Figure 10:**
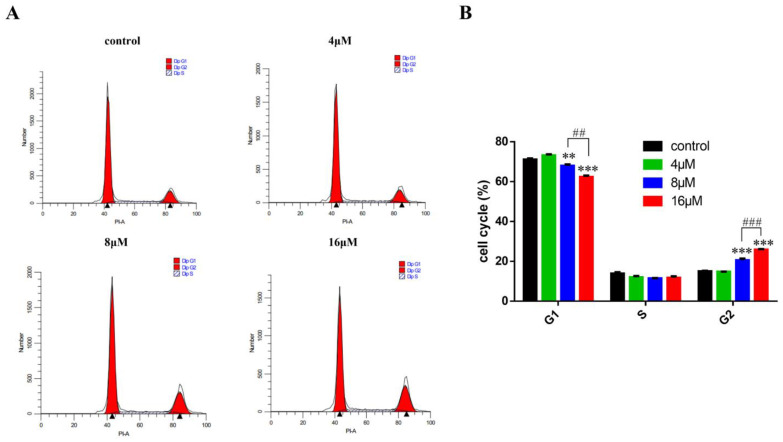
Cell cycle arrest induced by **2**. (**A**) HepG2 cells were incubated with varying concentrations of **2** (4.0, 8.0, and 16.0 μM) for 48 h. (**B**) Histograms showing the percentage of cell cycle distribution following **2** treatment (*n* = 3). The bar graphs are presented as mean ± SD. ***p < 0.001, ** *p* < 0.01 compared with the corresponding control group, ### *p* < 0.001, ## *p* <0.01 compared with the 16.0 μM-treated group, calculated by one-way ANOVA.

The most potent **2** was selected to evaluate its effects on the cell cycle of HepG2 cancer cells using flow cytometry. As shown in Figure 10A,B, after treatment with increasing concentrations of **2** (4.0, 8.0, and 16.0 μΜ) for 48h, the percentages of cells arrested at G2/M phase were increased from 14.83% to 25.91%, as compared to the control group (15.03%). Therefore, **2** could dose-dependently induce G2/M phase arrest in HepG2 cells.

## 3. Materials and Methods

### 3.1. General Experimental Procedures

HR-ESIMS spectra were obtained on a Bruker Daltonics Apex-Ultra 7.0 T (Bruker Corporation, Billerica, MA, USA). Optical rotations were recorded on an MCP500 modular circular polarimeter (Anton Paar GmbH, Graz, Austria) with a 0.5 cm cell at 25 °C and UV spectra were measured on a UV-2600 UV-Vis spectrophotometer (SHIMADZU) at room temperature. IR spectra were obtained on a SHIMADZU IRAffinity-1 Fourier transform infrared spectrometer and ECD spectra were recorded on a circular dichromatic spectrometer (Chirascan, Applied PhotoPhysics, Leatherhead, Surrey, UK). X-ray data were collected using an Agilent SuperNova with AtlasS2 X-ray single-crystal diffractometer with Cu Kα radiation. 1D and 2D NMR spectra were measured on a Bruker AV-400 or 700 MHz NMR spectrometer. High-performance liquid chromatography (HPLC) was performed on a Waters 2535 pump equipped with a 2998 photodiode array detector and C_18_ reversed-phase columns (YMC, Kyoto, Japan; 250 mm × 4.6 mm, length × i.d., 5 μm, for analysis; 250 mm × 10 mm, length × i.d., 5 μm, for preparation). Silica gel (Qingdao Haiyang Chemical Co., Ltd.; 100–200 and 200–300 mesh) and octadecylsilyl silica gel (YMC, Kyoto, Japan, ODS-A-HG, 12 nm, 50 µm) were used for column chromatography (CC).

### 3.2. Fungus Material

The fungal strain *Aspergillus* sp. A1E3 was isolated from the fruit of *Rhizophora mucronata*, collected from the Thai mangrove swamps of the Trang Province in February 2012. It was identified as *Aspergillus* sp. according to ITS rDNA sequence data. The strain was preserved in the School of Pharmaceutical Sciences, Southern Medical University.

### 3.3. Fermentation, Extraction and Isolation

The Fungal strain *Aspergillus* sp. A1E3 was inoculated into Erlenmeyer flasks (500 mL) containing 10‰ sea salt and potato glucose solution in a sterile environment at 25 °C for seven days to prepare the seed culture, which was then inoculated into 100 Erlenmeyer flasks (1000 mL) each containing rice solid medium (100 g rice and 150 mL water containing 10‰ sea salt) at room temperature in static conditions for 28 days. Then the fermentation material of *Aspergillus* sp. A1E3 was extracted three times with EtOAc, which was evaporated under vacuum. The resulting EtOAc extract was dissolved in EtOAc again and washed three times with water. The EtOAc solution was dried under reduced pressure to obtain the residue, which was then completely dissolved in MeOH/H_2_O (*v*/*v*, 1:1). After washing with double volume of *n*-hexane three times, the remaining aqueous methanol solution was dried to yield the resulting solid (70.11 g). The solid extract was then fractionated by silica gel column chromatography (200–300 mesh silica, 180 × 10 cm, i.d.) with a gradient mixture of CHCl_3_/MeOH (*v*/*v*, 100:0, 100:1, 50:1, 98:2, 30:1, 20:1, 10:1, 5:1, 3:1, 2:1, 1:1, 1:2) to afford 170 fractions. Fr. 42 and Fr. 43 were combined (3.89 g) and subjected to a C_18_ reversed-phase column (60 × 6 cm, i.d.), eluted with a gradient mixture of acetone/H_2_O (*v*/*v*, 50:50 to 100:0) to afford 70 subfractions. Then subfractions 22 and 23 were combined and further purified by semi-preparative HPLC (MeCN:H_2_O 58:42) to yield **2** (1.0 mg, t*_R_* = 90.0 min). Fr. 44 (1.01 g) was subjected to a C_18_ reversed-phase column (60 × 6 cm, i.d.), eluted with a gradient mixture of acetone/H_2_O (*v*/*v*, 50:50 to 100:0) to give 50 subfractions, among which subfraction 46 (112.6 mg) was further purified by semi-preparative HPLC (CH_3_CN:H_2_O 65:35) to afford **3** (40.0 mg, t*_R_* = 68.8 min). Fr. 145 (238.0 mg) was dissolved in chloroform and then filtered to give **1** (50.0 mg).

### 3.4. Spectroscopic Data of Compounds

Rhizoaspergillin A (**1**): white amorphous powder; [α${]}_{D}^{25}$—25.4 (*c* 0.10, MeOH); UV (MeOH) λ_max_ (log*ε*) 213 (1.81) nm (Figure S3); IR*v*_max_ 3744, 3649, 3443, 3256, 2361, 2338, 1713, 1678, 1616, 1443, 1379, 1323, 1252, 1173, 1136, 1065 cm^−1^ (Figure S6); ^1^H and ^13^C NMR spectroscopic data (see Table 1); HR-ESIMS *m*/*z* 395.1084 [M + H]^+^ (calcd for C_17_H_20_N_2_O_9_, 395.1085).

Rhizoaspergillinol A (**2**): light white amorphous powder; [α${]}_{D}^{25}$—57.6 (*c* 0.10, MeOH); UV (MeOH) λ_max_ (log*ε*) 206 (1.52) nm (Figure S4); IR*v*_max_ 3439, 2918, 2851, 2359, 1647, 1620, 1582, 1460, 1233, 1155, 1040 cm^−1^ (Figure S7); ^1^H and ^13^C NMR spectroscopic data (see Table 2); HR-ESIMS *m*/*z* 555.1644 [M + H]^+^ (calcd for C_32_H_27_O_9_, 555.1650).

Averufanin (**3**): Deep red amorphous powder; [α${]}_{D}^{25}$ + 27.8 (*c* 0.05, MeOH); UV (MeOH) λ_max_ (log*ε*) 208 (1.88) nm (Figure S5); IR*v*_max_ 3566, 3385, 3231, 2932, 2859, 2359, 2342, 1614, 1395, 1254, 1207, 1167, 1072, 1024, 995 cm^−1^ (Figure S8); ^1^H and ^13^C NMR spectroscopic data (see Table 3); HR-ESIMS *m*/*z* 371.1136 [M + H]^+^ (calcd for C_20_H_19_O_7_, 371.1125).

### 3.5. X-Ray Crystallographic Data of ***4***

C_32_H_42_N_2_O_12_, Mr 646.67. Colorless crystal from MeOH (mp 184.6–185.5). Temperature/K: 149.99(10). Crystal system: orthorhombic. Space group: P21212, a = 10.90168(15) Å, b = 11.02307(18) Å, c = 28.1523(4) Å, α: 90°, β: 90°, γ: 90°. Volume: 3383.06(9) Å3, Z: 4, ρcalcg: 1.270 cm^3^, μ: 0.816 mm^−1^, F(000): 1376.0. Crystal size: 0.26 × 0.05 × 0.03 mm^3^. Radiation: CuKα (λ = 1.54184). 2^Θ^ range for data collection: 6.28 to 129.988°. Index ranges: −12 ≤ h ≤ 12, −12 ≤ k ≤ 9, −33 ≤ l ≤ 33. Reflections collected: 15534. Independent reflections: 5739 [R_int_ = 0.0313, R_sigma_ = 0.0343]. Data/restraints/parameters: 5739/35/426. Goodness-of-fit on F^2^: 1.049. Final R indexes [I ≥ 2σ (I)]: R_1_ = 0.0415, wR_2_ = 0.1143. Final R indexes [all data]: R_1_ = 0.0439, wR_2_ = 0.1172. Largest diff. peak/hole: 0.73/−0.61 e Å^−3^. Flack parameter: −0.12(9) (CCDC 2291154).

### 3.6. Synthesis of the Derivative ***4***

Compound **1** (20.0 mg) was dissolved in pyridine (0.5 mL). DMAP (3.0 mg) was then added, along with 50.0 μL of pivaloyl chloride under ice bath and stirred for 10 min. After the completion of the reaction (monitored by thin-layer chromatography, i.e., TLC), the reaction solution was evaporated under reduced pressure. The resulting residue was dissolved in methanol, purified by semi-preparative HPLC, and eluted with the mixture of MeCN/H_2_O (*v*/*v*, 58:42, 3.0 mL/min) to afford **4** (5.0 mg). Suitable crystals of **4** were obtained in MeOH after considerable effort.

### 3.7. Cell Culture and Cytotoxicity (MTT) Assay

An MTT assay was used to determine the antiproliferative activities of compounds **1**–**3** and MS-275 (positive control) against four cancer cell lines, including a human lung cancer cell line (HepG2), mouse Lewis lung carcinoma (LLC) cells, a mouse skin melanoma cell line (B16-F10), and a human breast cancer cell line (MCF-7). Fetal bovine serum (FBS, 10%) in RPMI-1640 medium was used to culture MCF-7 cell lines and Fetal bovine serum (FBS, 10%) in DMEM medium was used to culture B16-F10, HepG2 and LLC cells. Cells were seeded into 96-well plates at a density of 5000 cells/well and incubated at 37 °C with 5% CO_2_ overnight. The next day, the three compounds and MS-275 (InvivoChem) were dissolved in a complete medium to prepare different concentrations of solution, which were added to each well, followed by incubation at 37 °C for 48 h. Finally, 20 μL of MTT (5 mg/mL dissolved in PBS) was added to each well and incubated with cells at 37 °C for 4 h. Then, the complete medium was removed and the formazan crystals were dissolved in 100 μL of DMSO in each well. The absorbance was measured in a TECAN microplate reader at 490 nm. GraphPad Prism was utilized to calculate the IC_50_ values by a model of nonlinear regression to fit a normalized dose response. All of the experiments were performed independently three times.

### 3.8. Cell Cycle Analysis

HepG2 cells were seeded to a six-well plate at a density of 5 × 10^5^ cells/well followed by incubation at 37 °C overnight. Then, different concentrations of **1**–**3** (4.0, 8.0, 16.0 μM) were added to the plate and incubated for 48 h. The collected cells were washed once with PBS. Then the cells were added 1.0 mL of DNA staining solution and 10.0 μL of permeabilization solution followed by incubation in the dark for 30.0 min. The DNA content of the samples was analyzed by flow cytometry.

## 4. Conclusions

In summary, two new compounds, named rhizoaspergillin A (**1**) and rhizoaspergillinol A (**2**), were obtained from the rice culture of the mangrove endophytic fungus *Aspergillus* sp. A1E3, together with the known compound averufanin (**3**). The planar structures and absolute configurations of rhizoaspergillinol A (**2**) and averufanin (**3**) were established by extensive NMR investigations and quantum-chemical electronic circular dichroism (ECD) calculations, whereas the constitution and absolute configuration of rhizoaspergillin A (**1**) were unambiguously determined by single-crystal X-ray diffraction analysis of its *tri*-pivaloyl derivative **4**, conducted with Cu Kα radiation. In addition, the absolute configuration of averufanin (**3**) was first clarified by ECD calculations. Most notably, rhizoaspergillin A (**1**) is the first reported orsellinic acid–ribose–pyridazinone-*N*-oxide hybrid containing a unique β-oxo-2,3-dihydropyridazine 1-oxide moiety, whereas rhizoaspergillinol A (**2**) is a sterigmatocystin derivative containing an additional diorcinol motif. From the perspective of biosynthesis, rhizoaspergillin A (**1**) could be originated from the combined assembly of three building blocks, viz., orsellinic acid, β-D-ribofuranose, and *L*-glutamine. It is an unprecedented alkaloid-*N*-oxide involving biosynthetic pathways of polyketides, pentose, and amino acids. Rhizoaspergillinol A (**2**) exhibited potent antiproliferative activity against a panel of cancer cell lines. Additionally, it could dose-dependently induce G2/M phase arrest in HepG2 cells. This work demonstrates that mangrove endophytic fungi of the genus *Aspergillus* harbor secondary metabolites with new structures.

## Figures and Tables

**Table 1:** ^1^H- and ^13^C-NMR Data for **1** and its Derivative **4** in DMSO-d_6_ and CDCl_3_, respectively (*δ* in ppm and *J* in Hz).

Position	1 ^a^		4 ^b^	
	*δ*_H_, Multi. (*J*)	*δ*_C_, Type	*δ*_H_, Multi. (*J*)	*δ*_C_, Type
1		163.3, C		162.1, C
2	5.78 d (8.0)	102.6, CH	5.79 d (8.0)	103.4, CH
3	7.96 d (8.0)	140.7, CH	7.39 d (8.0)	139.4, CH
4		151.1, C		149.7, C
5	5.96 d (6.4)	87.8, CH	6.06 d (6.3)	87.8, CH
6	4.46 dd (11.0, 5.8)	72.0, CH	5.47 t (6.3)	72.6, CH
7	5.45 dd (5.2, 2.8)	74.0, CH	5.65 t (4.9)	72.0, CH
8	4.26 dd (5.4, 2.8)	83.0, CH	4.56 m	80.4, CH
9	3.74 2H, br s	61.2, CH_2_	4.39 dd (12.6, 2.8) 4.47 dd (12.6, 3.5)	63.4, CH_2_
10		106.3, C		108.6, C
11		162.6, C		164.9, C
12	6.24 d (2.0)	100.9, CH	6.53 br s	117.0, CH
13		162.1, C		156.2, C
14	6.28 d (2.0)	111.2, CH	6.62 br s	108.9, CH
15		142.4, C		142.9, C
16		169.1, C		170.1, C
17	2.47 s	23.1, CH_3_	2.66 s	24.6, CH_3_
2’	11.40 s		8.19 s	
18				176.2, C
19				38.9, C
20			1.35 s	27.0, CH_3_
21			1.35 s	27.0, CH_3_
22			1.35 s	27.0, CH_3_
23				177.9, C
24				39.3, C
25			1.27 s	27.3, CH_3_
26			1.27 s	27.3, CH_3_
27			1.27 s	27.3, CH_3_
28				177.3, C
29				38.8, C
30			1.09 s	26.8, CH_3_
31			1.09 s	26.8, CH_3_
32			1.09 s	26.8, CH_3_
6-OH	6.00 d (5.6)			
9-OH	5.38 t (4.8)			
11-OH	10.85 s		11.08 s	
13-OH	10.17 s			

^a 1^H- and ^13^C-NMR data measured at 400 and 100 MHz, respectively; ^b 1^H- and ^13^C-NMR data measured at 700 and 175 MHz, respectively.

**Table 2:** ^1^H- and ^13^C-NMR data for **2** and dihydrosterigmatocystin in CDCl_3_ and DMSO-d_6_, respectively (*δ* in ppm and *J* in Hz).

Position	2 (CDCl_3_) ^a^		Dihydrosterigmatocystin (DMSO-d_6_) [26]	
	*δ*_H_, Multi. (*J*)	*δ*_C_, Type	*δ*_H_, Multi. (*J*)	*δ*_C_, Type
1		181.4, C		180.0, C
2		109.0, C		108.1, C
3		162.3, C		161.3, C
4	6.77 d (8.0)	111.4, CH	6.73 dd (8.5, 1.0)	110.5, CH
5	7.52 t (8.0)	135.8, CH	7.61 t (8.5)	136.0, CH
6	6.83 d (8.0)	105.9, CH	6.94 dd (8.5, 1.0)	106.1, CH
7		154.9, C		154.4, C
8		154.5, C		153.8, C
9		106.6, C		105.4, C
10		164.8, C		165.8, C
11	6.43 s	90.6, CH	6.60 s	90.2, CH
12		163.7, C		162.9, C
13		106.1, C		104.8, C
14	6.56 d (5.6)	112.3, CH	6.55 d (5.5)	113.4, CH
15	4.37 m	42.5, CH	4.25 m	43.3, CH
16	2.61 m, 2.73 m	37.4, CH_2_	2.24 m, 2.45 m	30.7, CH_2_
17	5.89 t (4.9)	103.3, CH	3.54 m, 4.10 m	67.2, CH_2_
18		157.6, C		56.5, OCH_3_
19	6.67 br s	112.0, CH		
20		140.9, C		
21	6.51 br s	114.0, CH		
22		157.8, C		
23		158.2, C		
24	6.400 d (2.0)	111.4, CH		
25		141.0, C		
26	6.399 d (2.0)	111.1, CH		
27		156.5, C		
28	6.29 t (2.0)	103.3, CH		
29	6.56 d (2.0)	105.1, CH		
30	2.30 s	21.5, CH_3_		
31	2.27 s	21.7, CH_3_		
32	4.01 s	56.9, CH_3_		
3-OH	13.22 s		13.38 s	
27-OH	4.71 br s			

^a 1^H- and ^13^C-NMR data measured at 700 and 175 MHz, respectively.

**Table 3:** ^1^H- and ^13^C-NMR Data for **3** in DMSO-d_6_ (*δ* in ppm and *J* in Hz).

Position	3 ^a^	
	*δ*_H_, multi. (*J*)	*δ*_C_, type
1		188.5, C
2		108.4, C
3		165.5, C
4	6.51 d (1.2)	108.0, CH
5		164.3, C
6	7.03 br s	109.0, CH
7		134.7, C
8		181.0, C
9		133.1, C
10	7.00 s	109.0, CH
11		161.5, C
12		119.8, C
13		162.9, C
14		108.2, C
15	4.94 d (10.8)	73.3, CH
16	1.60 m, 1.93 m	28.3, CH_2_
17	1.63 m, 1.87 m	23.4, CH_2_
18	1.30 m, 1.65 m	32.5, CH_2_
19	3.61 m	74.6, CH
20	1.18 d (6.0)	21.9, CH_3_

^a 1^H- and ^13^C-NMR data measured at 400 and 100 MHz, respectively.

**Table 4:** In vitro antiproliferative activities of **1**–**3**.

Compounds	IC_50_ (μΜ) ± SD			
	HepG2	LLC	B16-F10	MCF7
**1**	>50.0	>50.0	>50.0	>50.0
**2**	8.83 ± 3.43	14.18 ± 3.84	15.12 ± 1.45	>50.0
**3**	39.86 ± 1.27	>50.0	48.63 ± 1.20	>50.0
MS-275 ^a^	1.01 ± 0.25	5.36 ± 1.05	4.00 ± 0.28	14.74 ± 0.44

^a^ MS-275 was used as a positive control.

## Data Availability

Raw data discussed in this study are available in the Supplementary Materials.

## Supplementary Materials

The following supporting information can be downloaded at: https://www.mdpi.com/article/10.3390/md21110598/s1, HR-ESIMS, 1D and 2D NMR spectra of compounds **1**–**4**, UV and IR spectra of compounds **1**–**3**, along with energy analyses of conformers for compounds **2** and **3**.
