# Supplementary material for: Rhizoaspergillin A and Rhizoaspergillinol A, including a Unique Orsellinic Acid–Ribose–Pyridazinone-N-Oxide Hybrid, from the Mangrove Endophytic Fungus Aspergillus sp. A1E3
Source: Mar Drugs. 2023 Nov 19;21(11):598. doi: 10.3390/md21110598 (PMC10671915; doi:10.3390/md21110598)

Supporting Information

Table of Contents:

1. HR-ESIMS for **1** ··· S3-S4
2. ^{1}H (400 MHz) NMR spectrum of **1** in DMSO-d_6 ··· S5-S8
3. ^{13}C (100 MHz) NMR spectrum of **1** in DMSO-d_6 ··· S9-S12
4. DEPT135 (100 MHz) spectrum of **1** in DMSO-d_6 ··· S13-S15
5. ^{1}H–^{1}H COSY (400 MHz) spectrum of **1** in DMSO-d_6 ··· S16-S18
6. HSQC (400 MHz) spectrum of **1** in DMSO-d_6 ··· S19-S21
7. HMBC (400 MHz) spectrum of **1** in DMSO-d_6 ··· S22-S25
8. NOESY (400 MHz) spectrum of in DMSO-d_6 ··· S26-S27
9. HR-ESIMS for **2** ··· S28-S29
10. ^{1}H (700 MHz) NMR spectrum of **2** in $CDCl_3$ ··· S30-S33
11. ^{13}C (175 MHz) NMR spectrum of **2** in $CDCl_3$ ··· S34-S38
12. DEPT135 (175 MHz) spectrum of **2** in $CDCl_3$ ··· S39-S42
13. ^{1}H–^{1}H COSY (700 MHz) spectrum of **2** in $CDCl_3$ ··· S43-S44
14. HSQC (700 MHz) spectrum of **2** in $CDCl_3$ ··· S45-S47
15. HMBC (700 MHz) spectrum of **2** in $CDCl_3$ ··· S48-S50
16. NOESY (700 MHz) spectrum of **2** in $CDCl_3$ ··· S51-S52
17. HR-ESIMS for **3** ··· S53
18. ^{1}H (400 MHz) NMR spectrum of **3** in DMSO-d_6 ··· S54-S56
19. ^{13}C (100 MHz) NMR spectrum of **3** in DMSO-d_6 ··· S57-S60

20. DEPT135 (100 MHz) spectrum of **3** in DMSO-d_6 ···· S61-S63
21. ^1H–^1H COSY (400 MHz) spectrum of **3** in DMSO-d_6 ···· S64-S66
22. HSQC (400 MHz) spectrum of **3** in DMSO-d_6 ···· S67-S69
23. HMBC (400 MHz) spectrum of **3** in DMSO-d_6 ···· S70-S72
24. NOESY (400 MHz) spectrum of **3** in DMSO-d_6 ···· S73-S74
25. HR-ESIMS for **4** ···· S75-S76
26. ^1H (700 MHz) NMR spectrum of **4** in $CDCl_3$ ···· S77-S79
27. ^{13}C (175 MHz) NMR spectrum of **4** in $CDCl_3$ ···· S80-S84
28. DEPT135 (175 MHz) spectrum of **4** in $CDCl_3$ ···· S85-S87
29. ^1H–^1H COSY (700 MHz) spectrum of **4** in $CDCl_3$ ···· S88-S90
30. HSQC (700 MHz) spectrum of **4** in $CDCl_3$ ···· S91-S93
31. HMBC (700 MHz) spectrum of **4** in $CDCl_3$ ···· S94-S98
32. NOESY (700 MHz) spectrum of **4** in $CDCl_3$ ···· S99-S103
33. Figure S1. Energy analyses of conformers (14*S*,15*S*,17*R*)-2A to (14*S*,15*S*,17*R*)-2J ···· S104
34. Figure S2. Energy analyses of conformers (15*R*,19*R*)-3A to (15*R*,19*R*)-3E ···· S105
35. Figure S3. The UV spectrum of **1** ···· S106
36. Figure S4. The UV spectrum of **2** ···· S107
37. Figure S5. The UV spectrum of **3** ···· S108
38. Figure S6. The IR spectrum of **1** ···· S109
39. Figure S7. The IR spectrum of **2** ···· S110
40. Figure S8. The IR spectrum of **3** ···· S111

HR-ESIMS for **1**

Mass Spectrum SmartFormula Report

Analysis Info

Analysis Name	D:\Data\MS\data\202110\wubinbin_WBB-145-D_pos_52_01_11734.d	Acquisition Date	10/27/2021 4:41:37 PM
Method	LC_Direct Infusion_pos_70-500mz.m	Operator	SCSIO
Sample Name	wubinbin_WBB-145-D_pos	Instrument	maXis 255552.00029
Comment			

Acquisition Parameter

Source Type	ESI	Ion Polarity	Positive	Set Nebulizer	0.4 Bar
Focus	Active	Set Capillary	4500 V	Set Dry Heater	180 °C
Scan Begin	70 m/z	Set End Plate Offset	-500 V	Set Dry Gas	4.0 l/min
Scan End	1500 m/z	Set Charging Voltage	0 V	Set Divert Valve	Waste
		Set Corona	0 nA	Set APCI Heater	0 °C

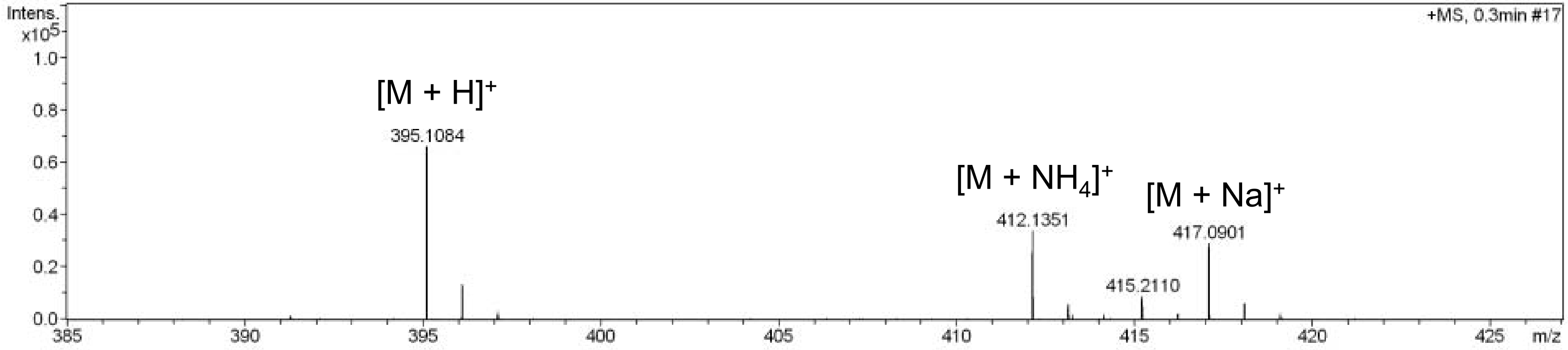

Meas. m/z	#	Ion Formula	Score	m/z	err [ppm]	err [mDa]	mSigma	rdb	e⁻ Conf	N-Rule
395.1084	1	C17H19N2O9	100.00	395.1085	-0.3	-0.1	3.9	9.5	even	ok
417.0901	1	C17H18N2NaO9	100.00	417.0905	0.8	0.4	6.1	9.5	even	ok

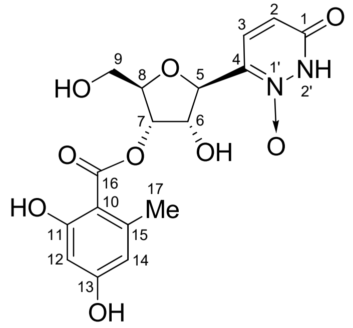

1

HR-ESIMS for **1**

Generic Display Report

Analysis Info

Analysis Name	D:\Data\MS\data\202110\wubinbin_WBB-145-D_pos_52_01_11734.d	Acquisition Date	10/27/2021 4:41:37 PM
Method	LC_Direct Infusion_pos_70-500mz.m	Operator	SCSIO
Sample Name	wubinbin_WBB-145-D_pos	Instrument	maXis
Comment			

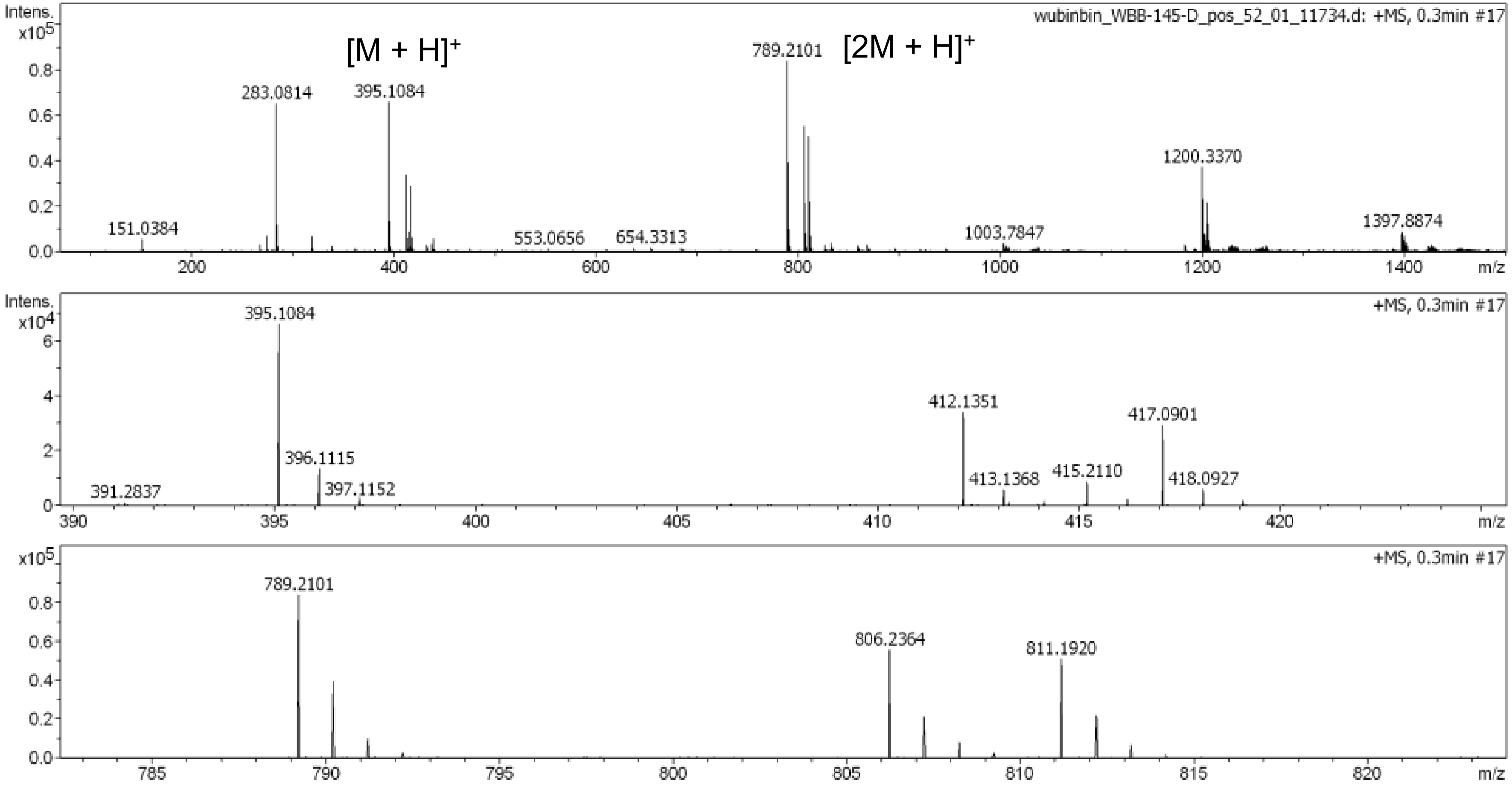

^{1}H (400 MHz) NMR spectrum of **1** in DMSO-d_6

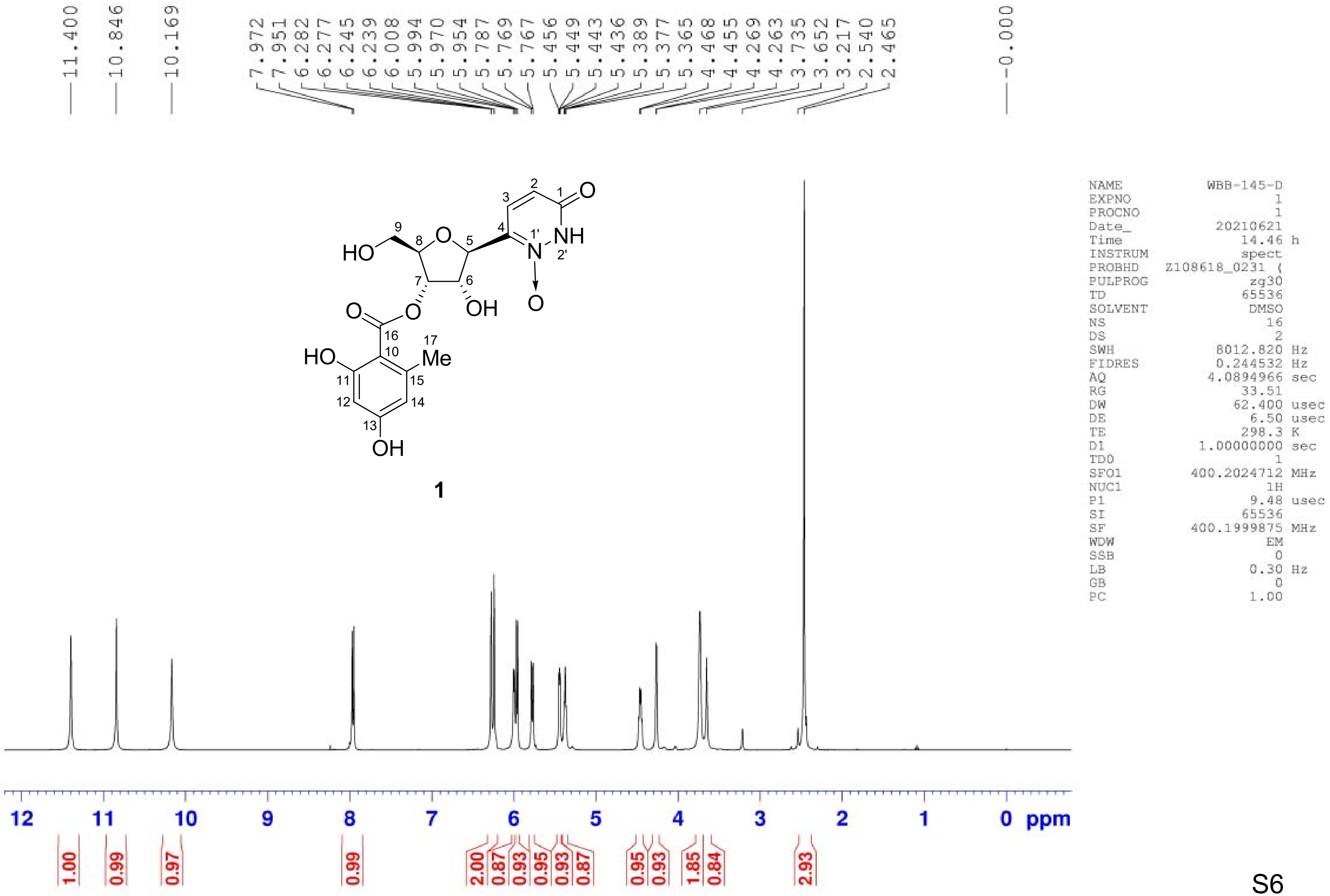

^{1}H (400 MHz) NMR spectrum of **1** in DMSO-d_6

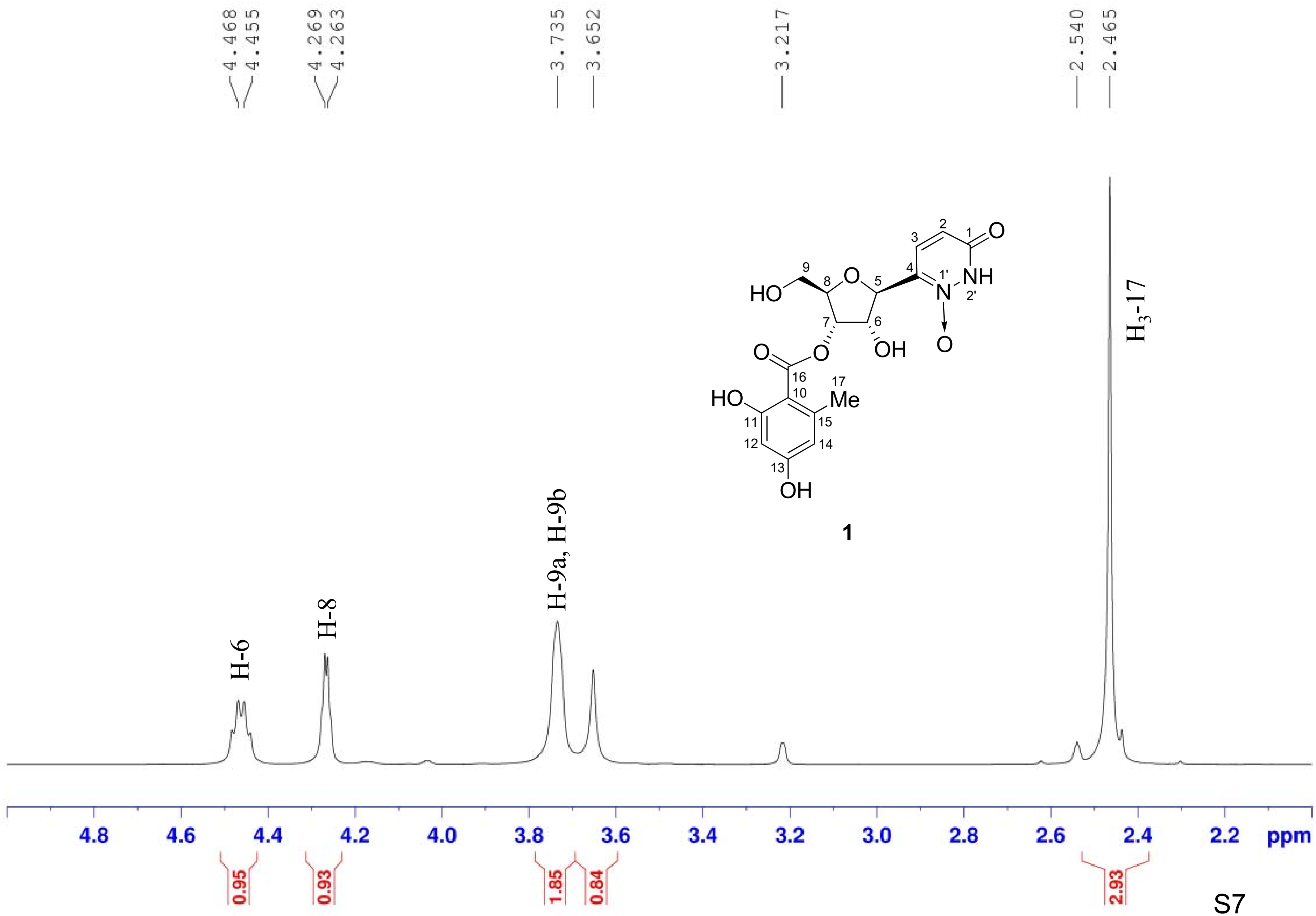

^{1}H (400 MHz) NMR spectrum of **1** in DMSO-d_6

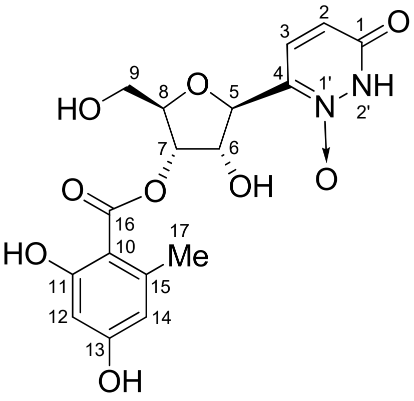

1

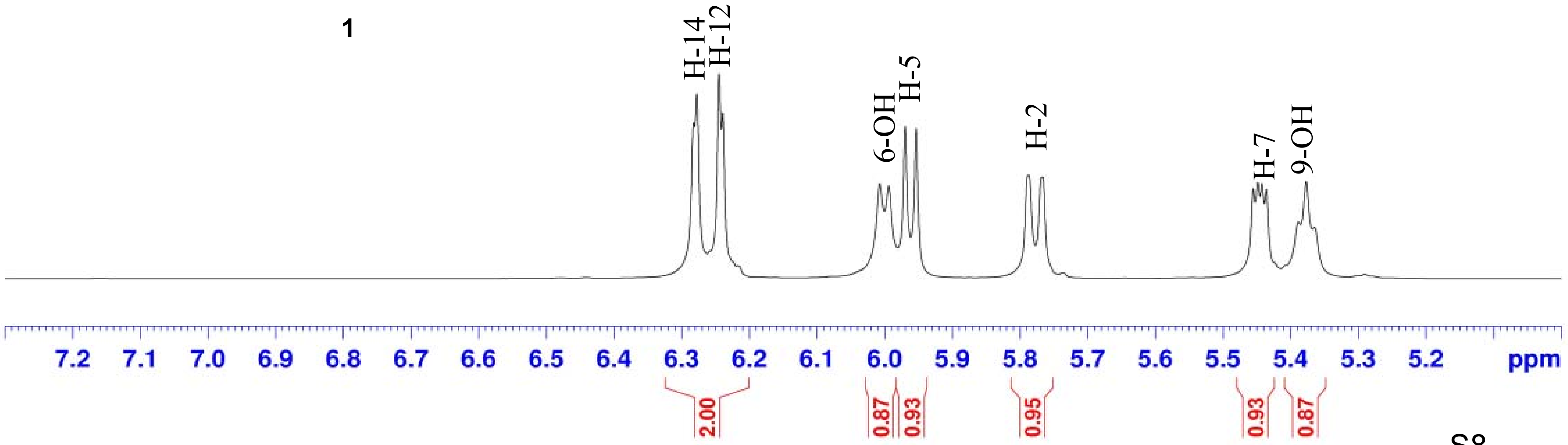

^{1}H (400 MHz) NMR spectrum of **1** in DMSO-d_6

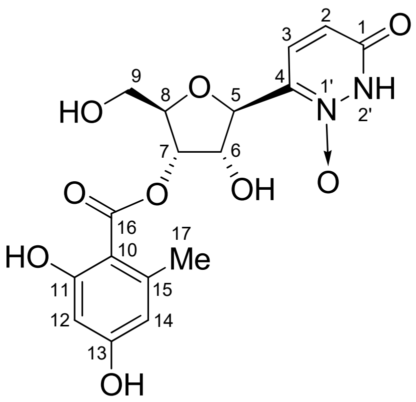

1

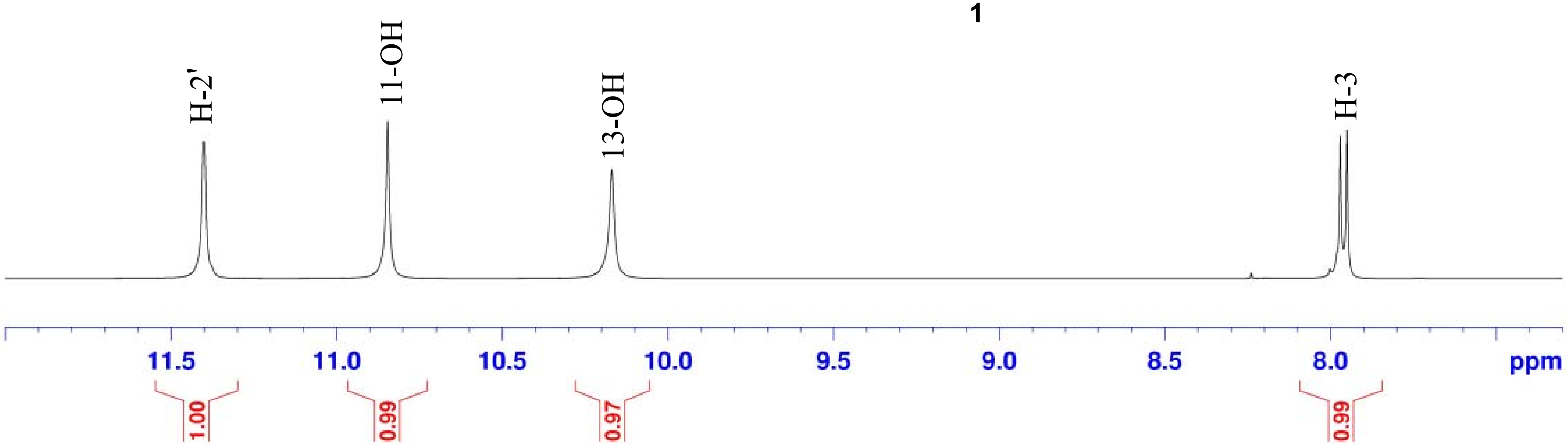

^{13}C (100 MHz) NMR spectrum of **1** in DMSO-d_6

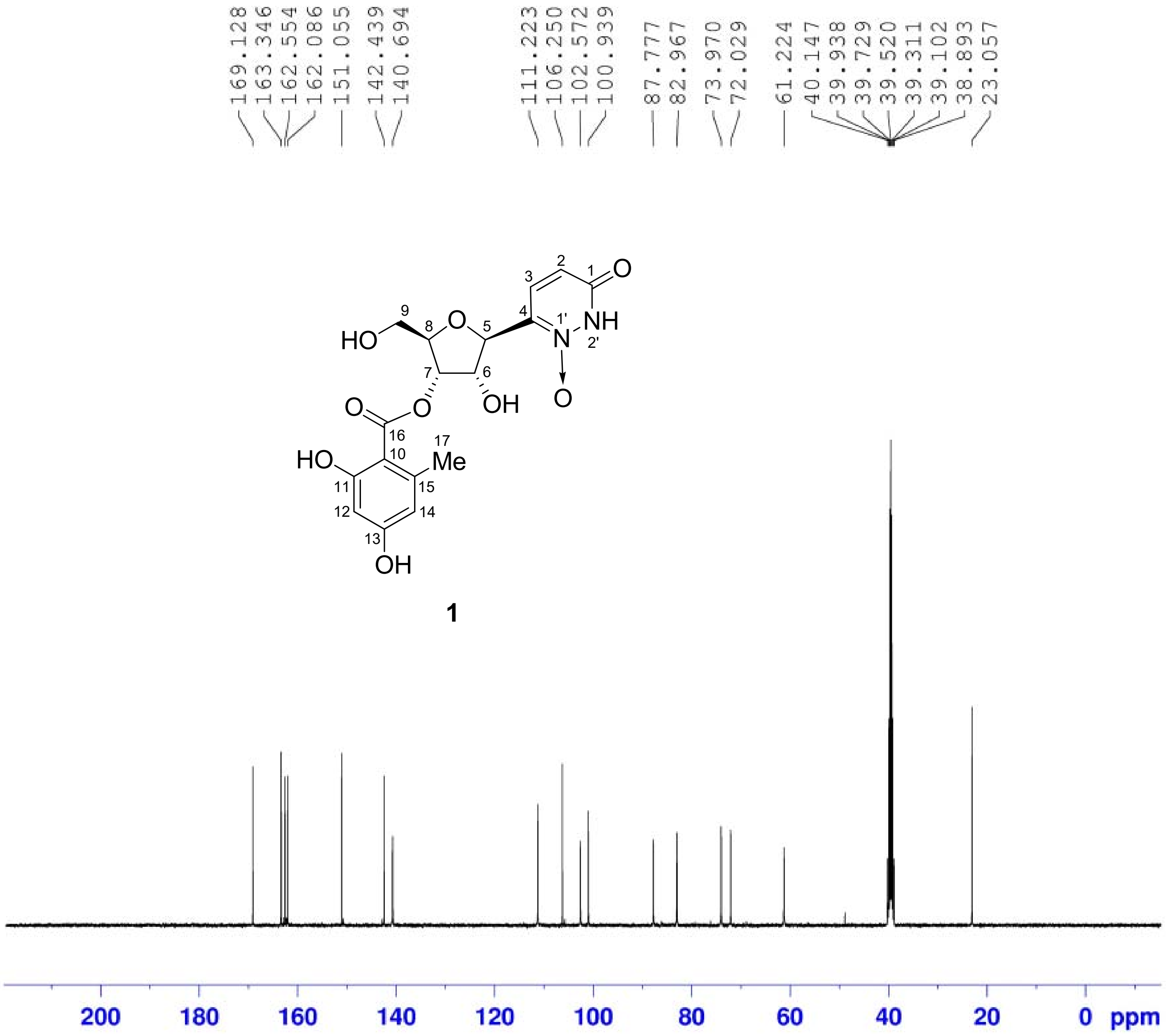

NAME	WBB-145-D
EXPNO	2
PROCNO	1
Date_	20210621
Time	22.07 h
INSTRUM	spect
PROBHD	Z108618_0231 (
PULPROG	zgpg30
TD	65536
SOLVENT	DMSO
NS	300
DS	4
SWH	24038.461 Hz
FIDRES	0.733596 Hz
AQ	1.3631988 sec
RG	2050
DW	20.800 usec
DE	6.50 usec
TE	299.6 K
D1	2.00000000 sec
D11	0.03000000 sec
TD0	1
SFO1	100.6404331 MHz
NUC1	13C
P1	8.25 usec
SI	32768
SF	100.6303921 MHz
WDW	EM
SSB	0
LB	1.00 Hz
GB	0
PC	1.40

^{13}C (100 MHz) NMR spectrum of **1** in DMSO-d_6

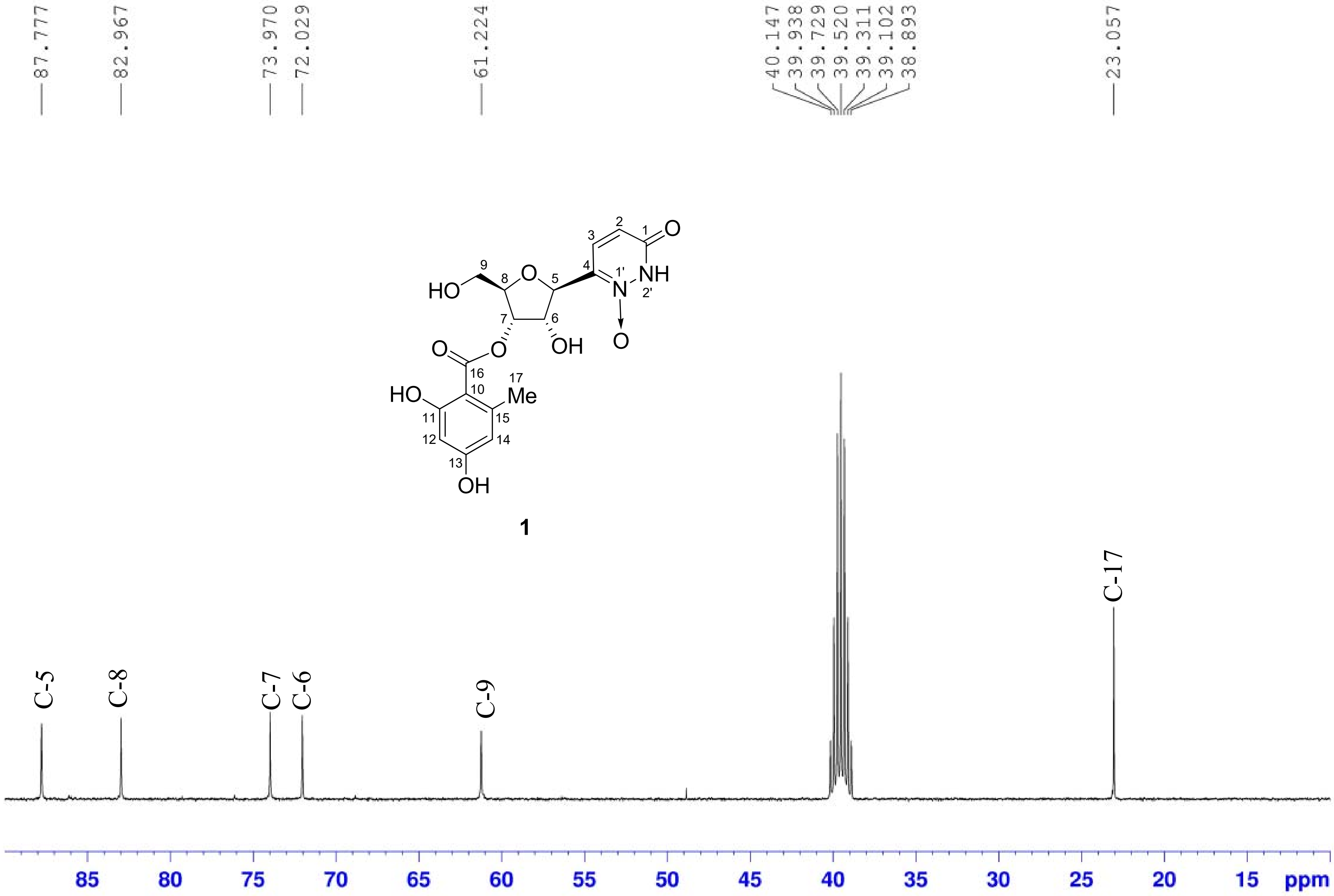

^{13}C (100 MHz) NMR spectrum of **1** in DMSO-d_6

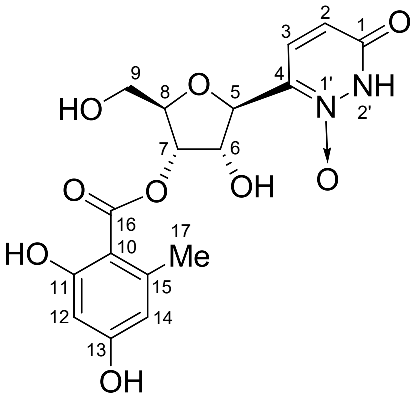

1

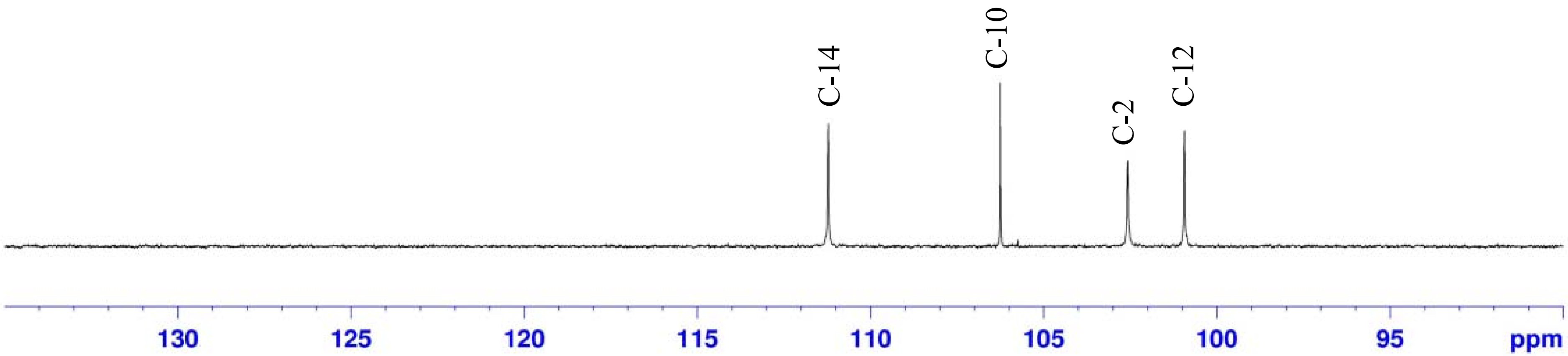

^{13}C (100 MHz) NMR spectrum of **1** in DMSO-d_6

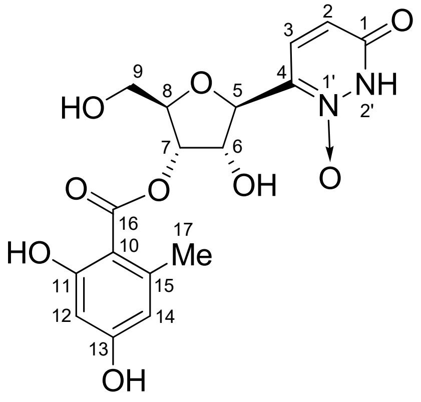

1

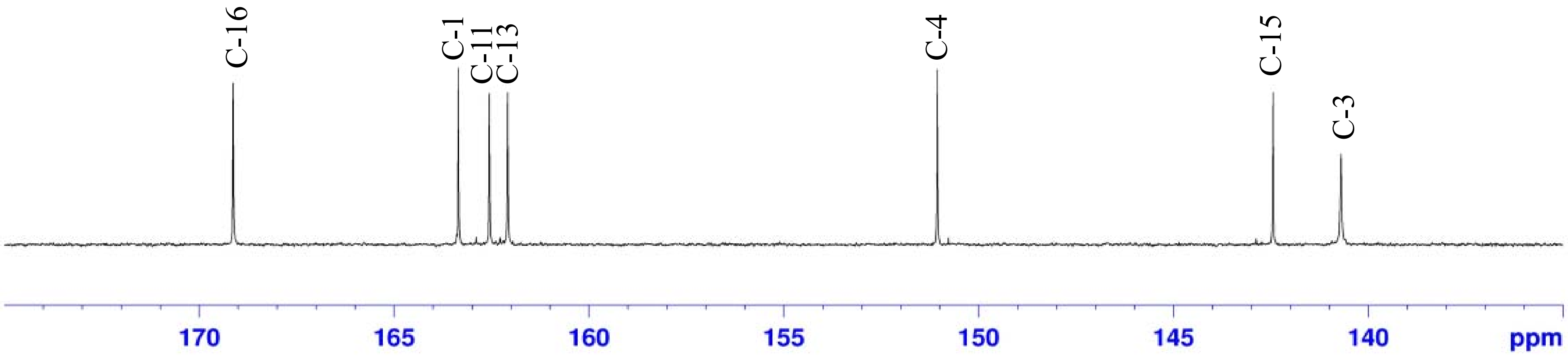

DEPT135 (100 MHz) spectrum of **1** in DMSO-d_6

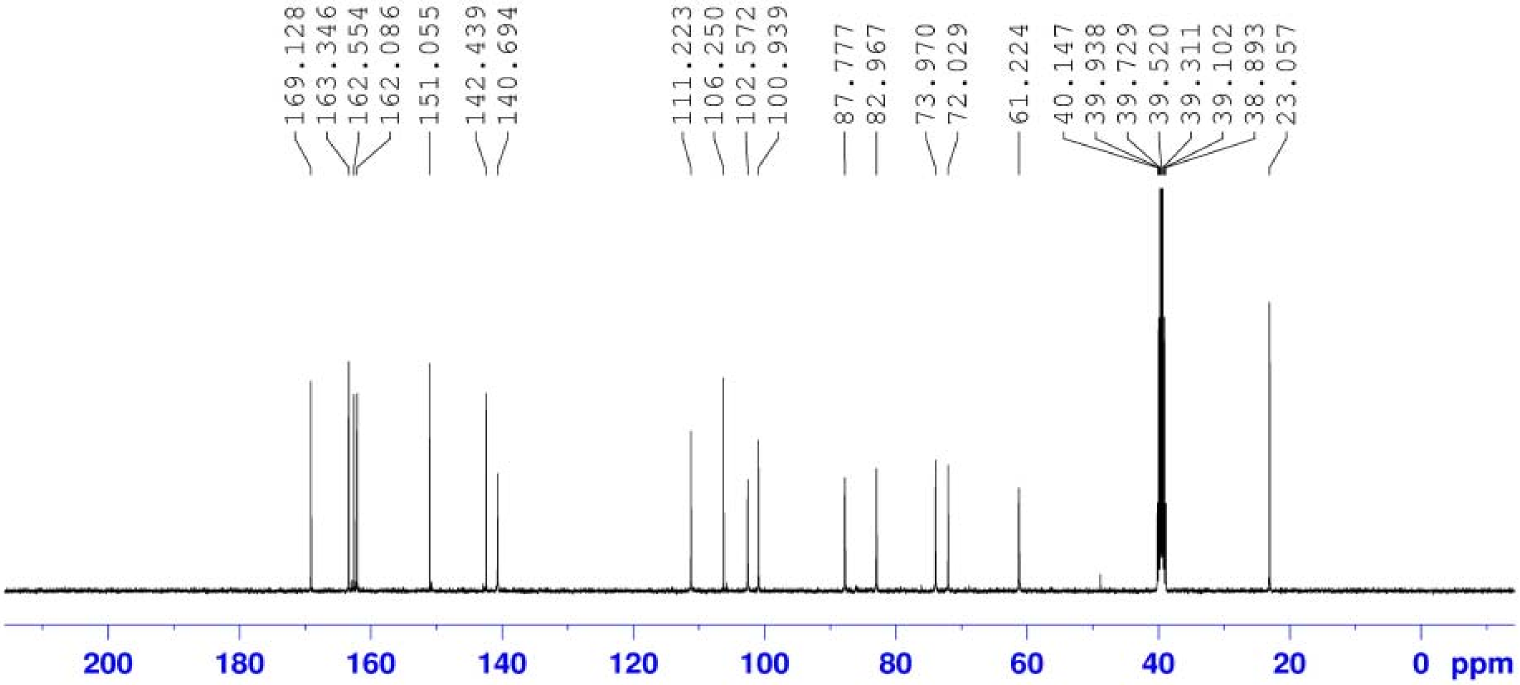

NAME	WBB-145-D	
EXPNO	3	
PROCNO	1	
Date_	20210621	
Time	22.30	h
INSTRUM	spect	
PROBHD	Z108618_0231 (	
PULPROG	deptsp135	
TD	65536	
SOLVENT	DMSO	
NS	150	
DS	8	
SWH	16129.032	Hz
FIDRES	0.492219	Hz
AQ	2.0316660	sec
RG	2050	
DW	31.000	usec
DE	6.50	usec
TE	299.3	K
CNST2	145.0000000	
D1	2.00000000	sec
D2	0.00344828	sec
D12	0.00002000	sec
TD0	1	
SFO1	100.6384205	MHz
NUC1	13C	
P1	8.25	usec
P13	2000.00	usec
SI	32768	
SF	100.6303700	MHz
WDW	EM	
SSB	0	
LB	1.00	Hz
GB	0	
PC	1.40	

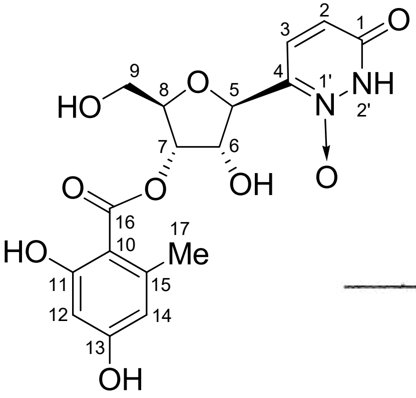

1

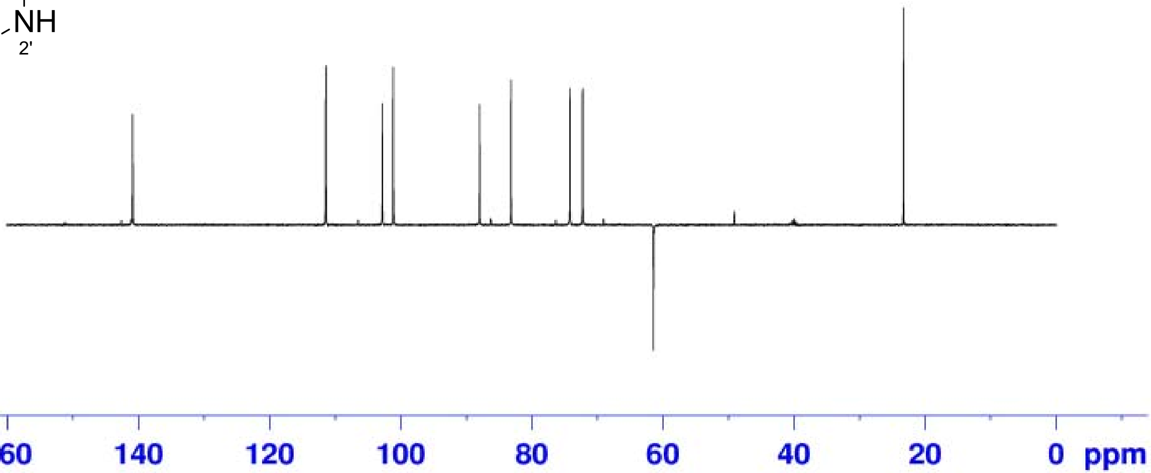

DEPT135 (100 MHz) spectrum of **1** in DMSO-d_6

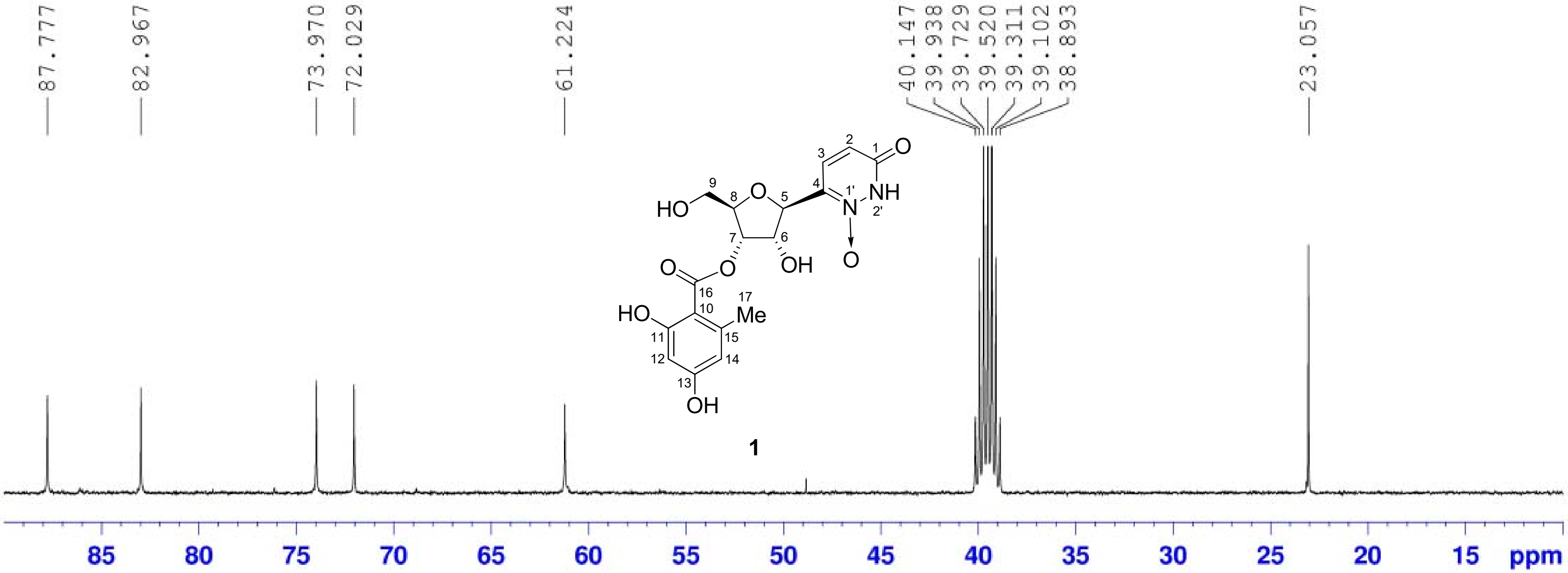

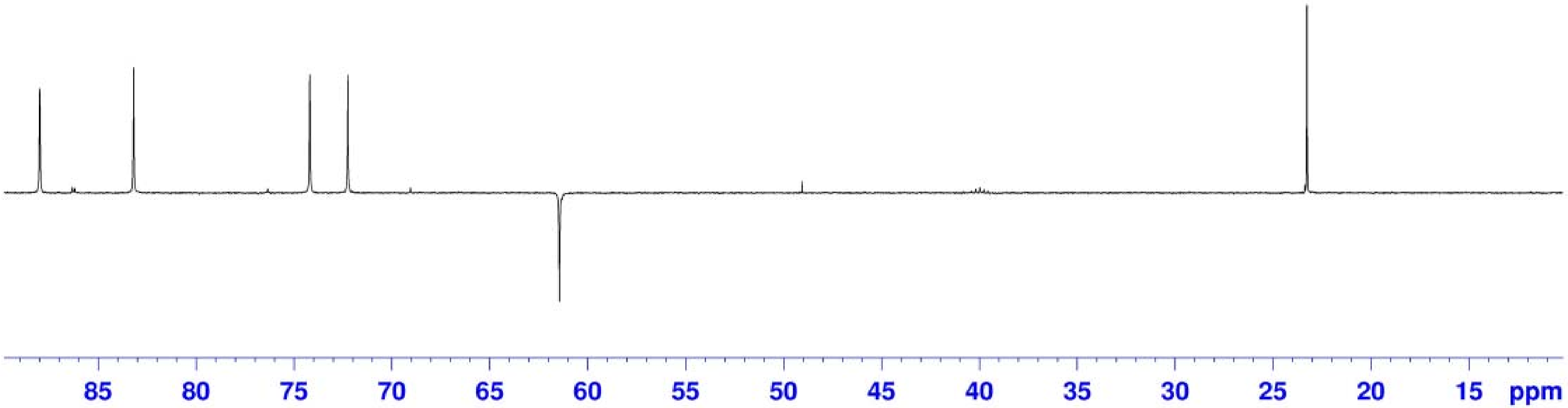

DEPT135 (100 MHz) spectrum of **1** in DMSO-d_6

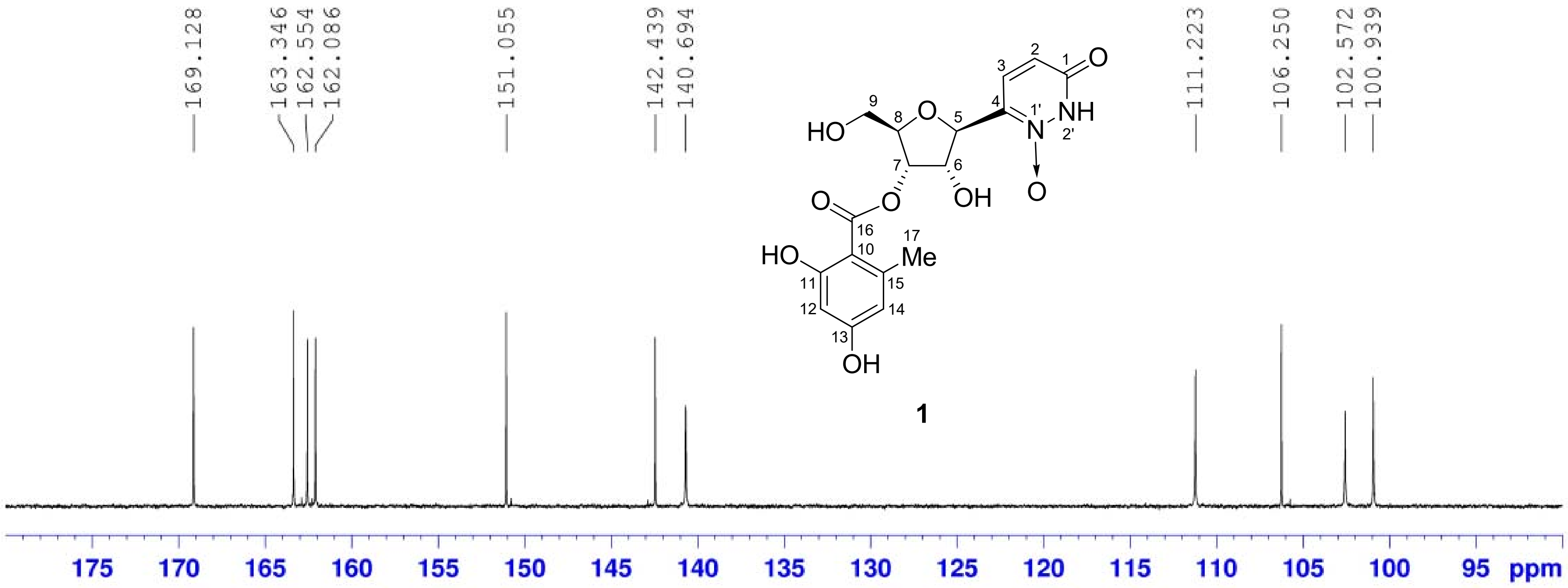

1

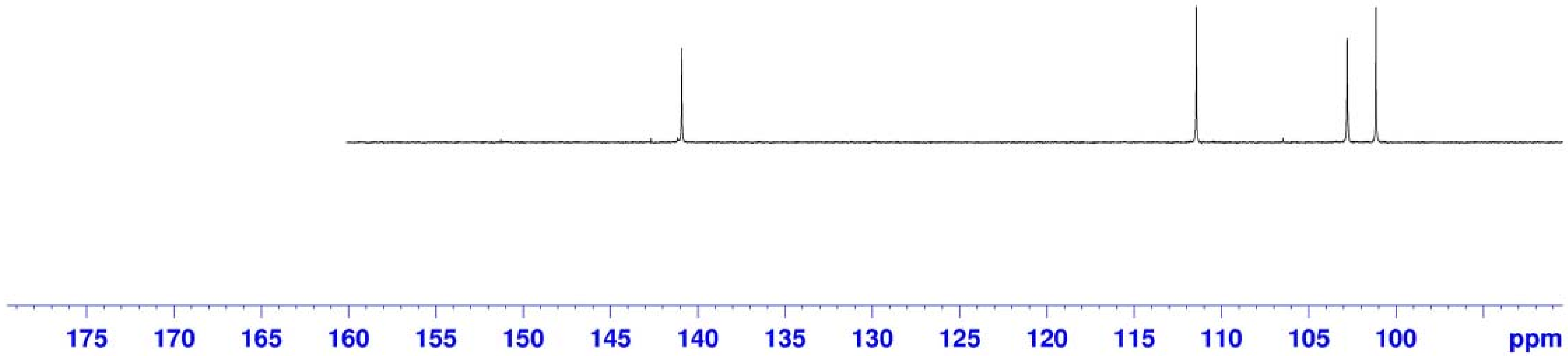

^1H–^1H COSY (400 MHz) spectrum of **1** in DMSO-d_6

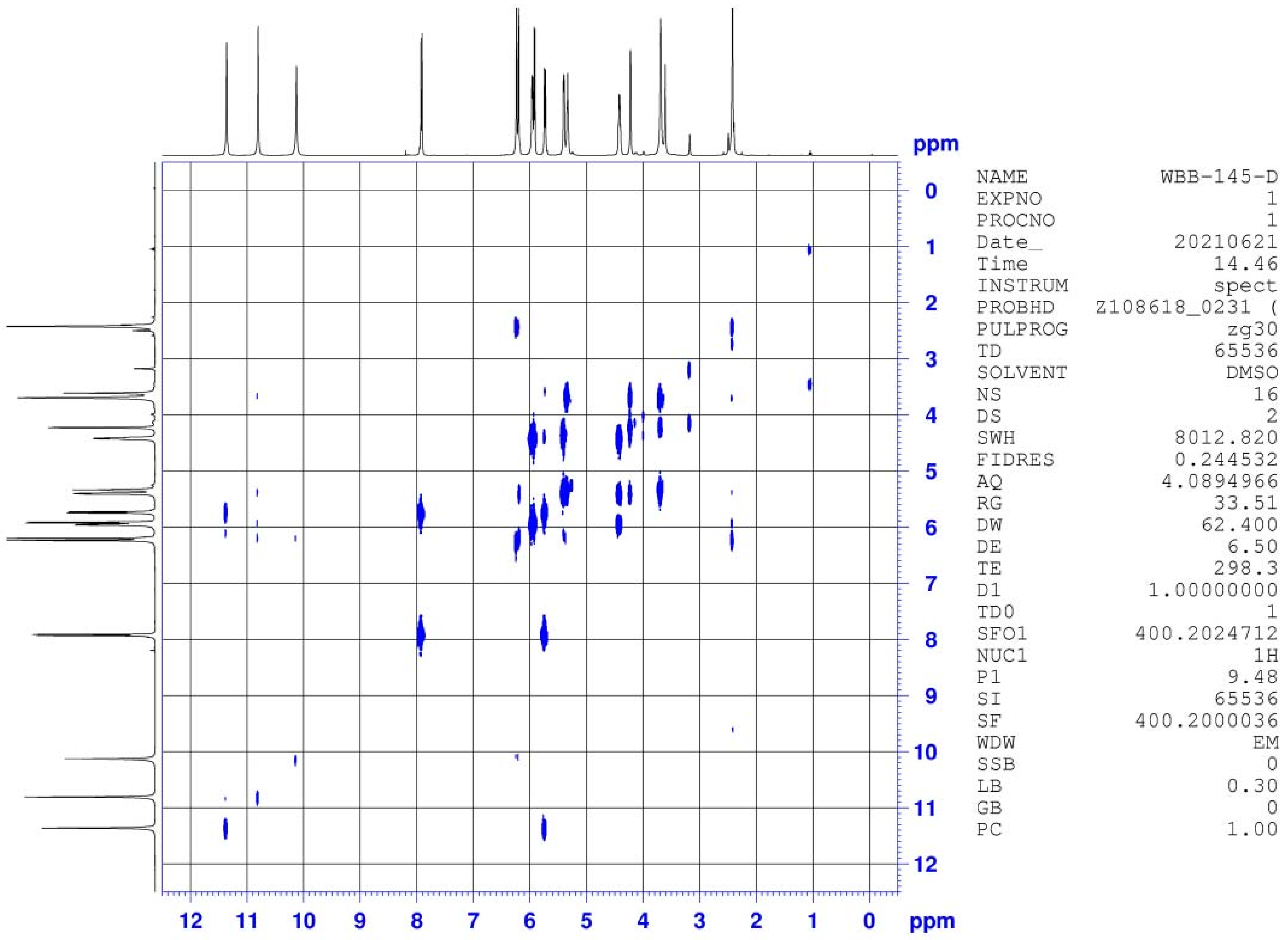

^{1}H–^{1}H COSY (400 MHz) spectrum of **1** in DMSO-d_6

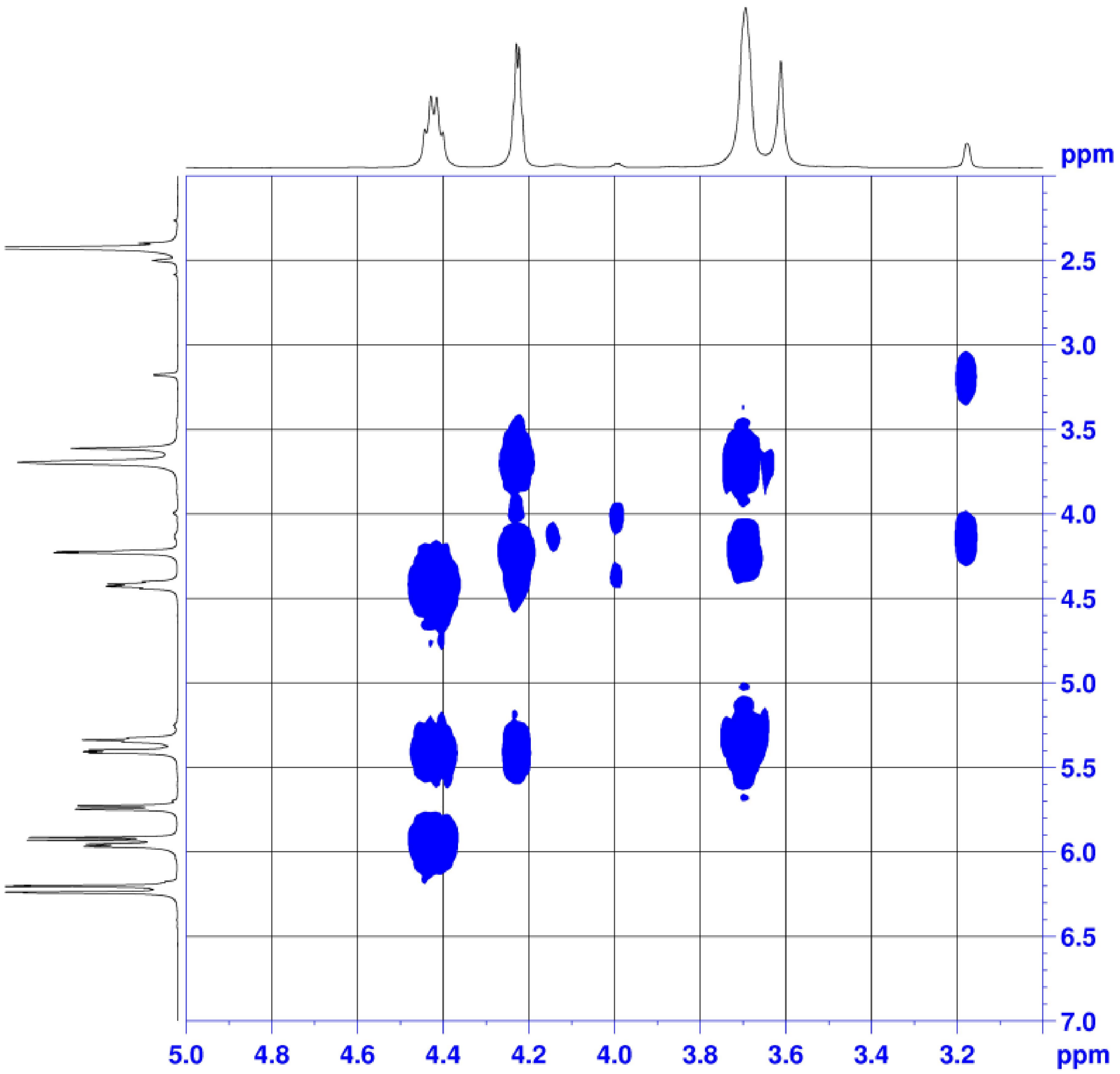

^{1}H–^{1}H COSY (400 MHz) spectrum of **1** in DMSO-d_6

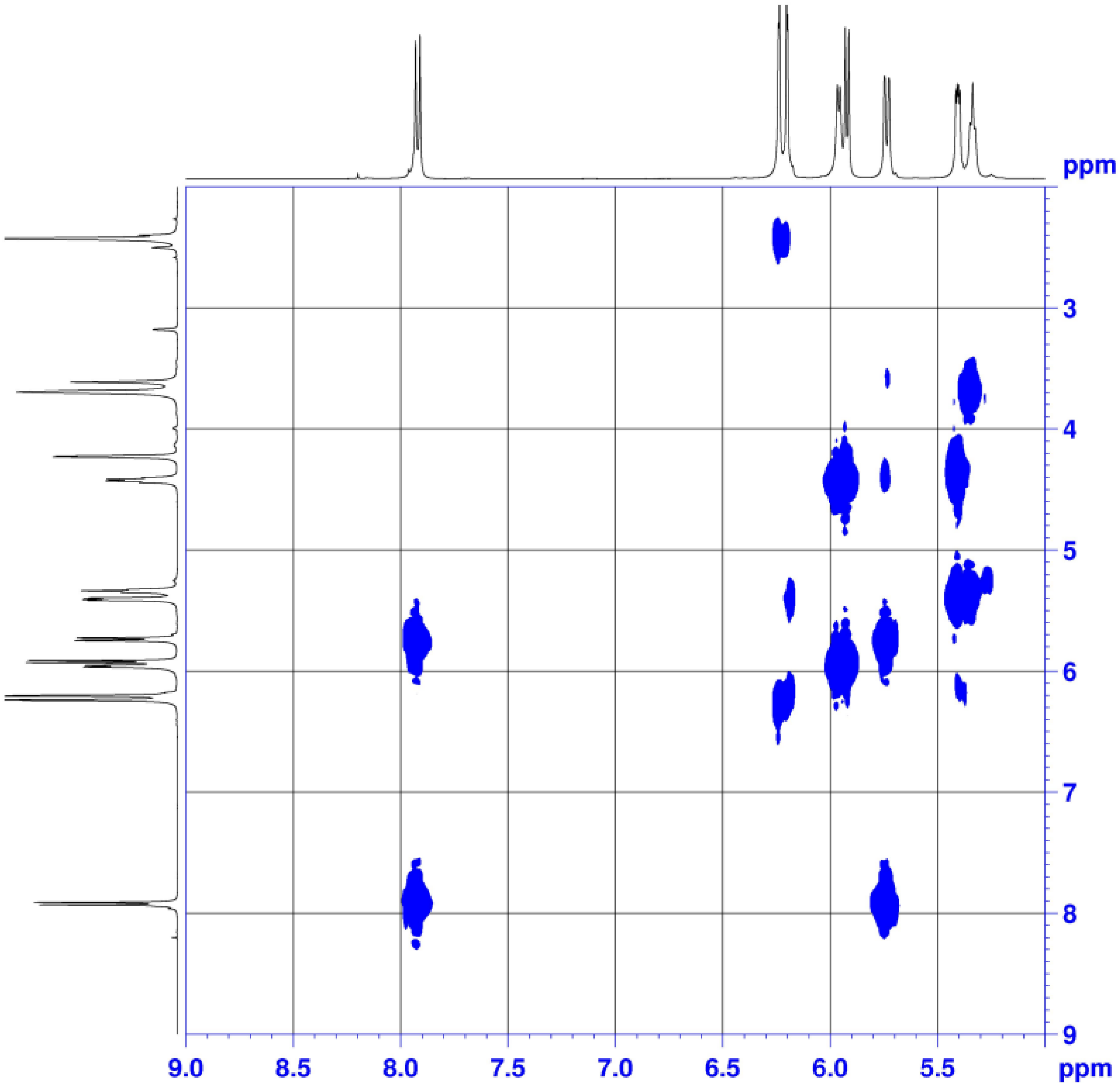

HSQC (400 MHz) spectrum of **1** in DMSO-d_6

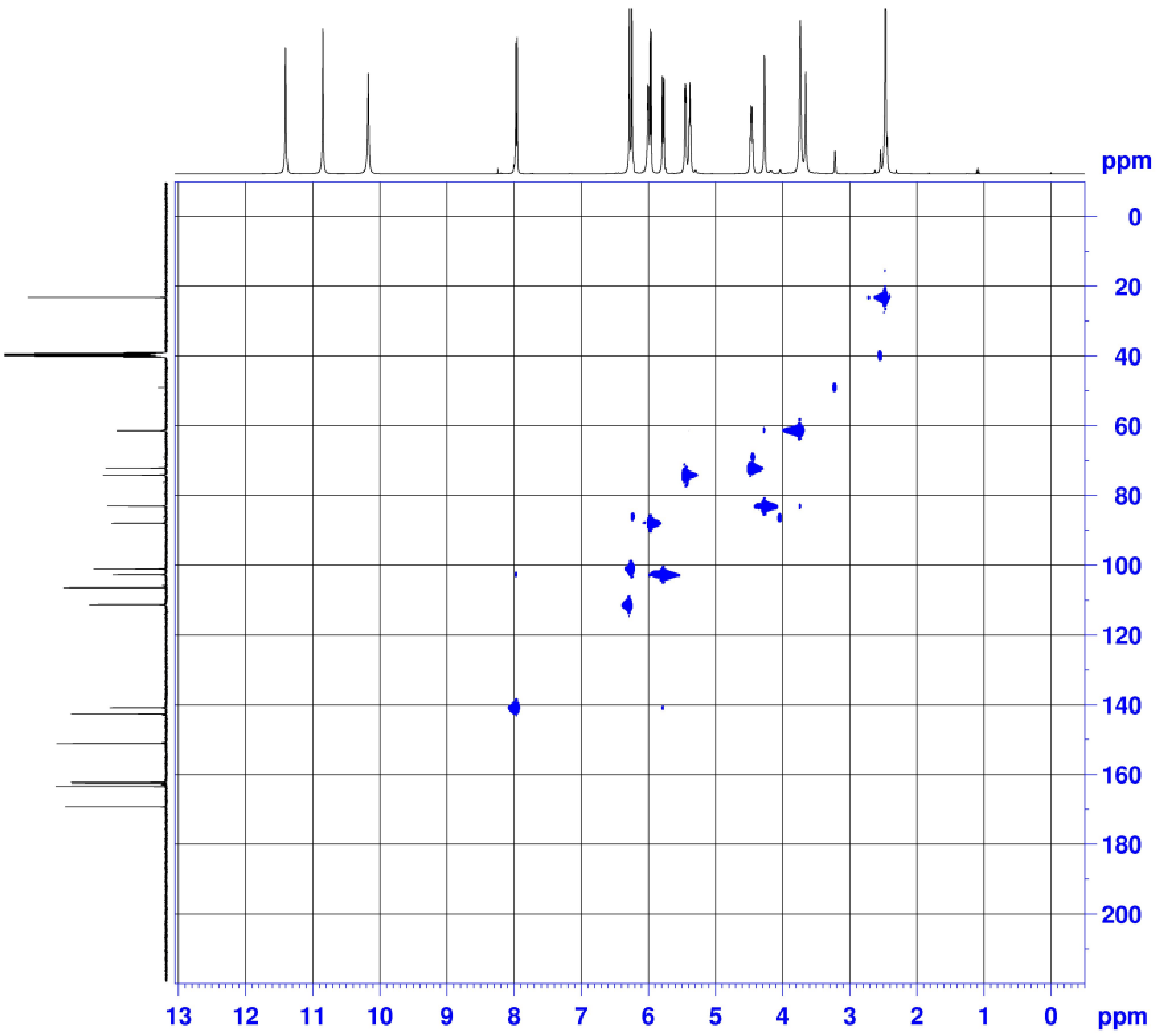

NAME	WBB-145-D	
EXPNO	4	
PROCNO	1	
Date_	20210622	
Time	22.15	h
INSTRUM	spect	
PROBHD	Z108618_0231 (	
PULPROG	hsqcetgpsi	
TD	1024	
SOLVENT	DMSO	
NS	4	
DS	16	
SWH	5597.015	Hz
FIDRES	10.931670	Hz
AQ	0.0915273	sec
RG	2050	
DW	89.333	usec
DE	6.50	usec
TE	297.2	K
CNST2	145.0000000	
D0	0.00000300	sec
D1	1.50000000	sec
D4	0.00172414	sec
D11	0.03000000	sec
D16	0.00020000	sec
D24	0.00110000	sec
IN0	0.00002070	sec
ND0	2	
TD	128	
SFO1	100.6379	MHz
FIDRES	188.707733	Hz
SW	240.015	ppm
FnMODE	Echo-Antiecho	
SI	1024	
SF	400.1999816	MHz
WDW	QSINE	
SSB	2	
LB	0.00	Hz
GB	0	
PC	1.40	
SI	1024	
MC2	echo-antiecho	
SF	100.6303700	MHz
WDW	QSINE	
SSB	2	
LB	0.00	Hz
GB	0	

HSQC (400 MHz) spectrum of **1** in DMSO-d_6

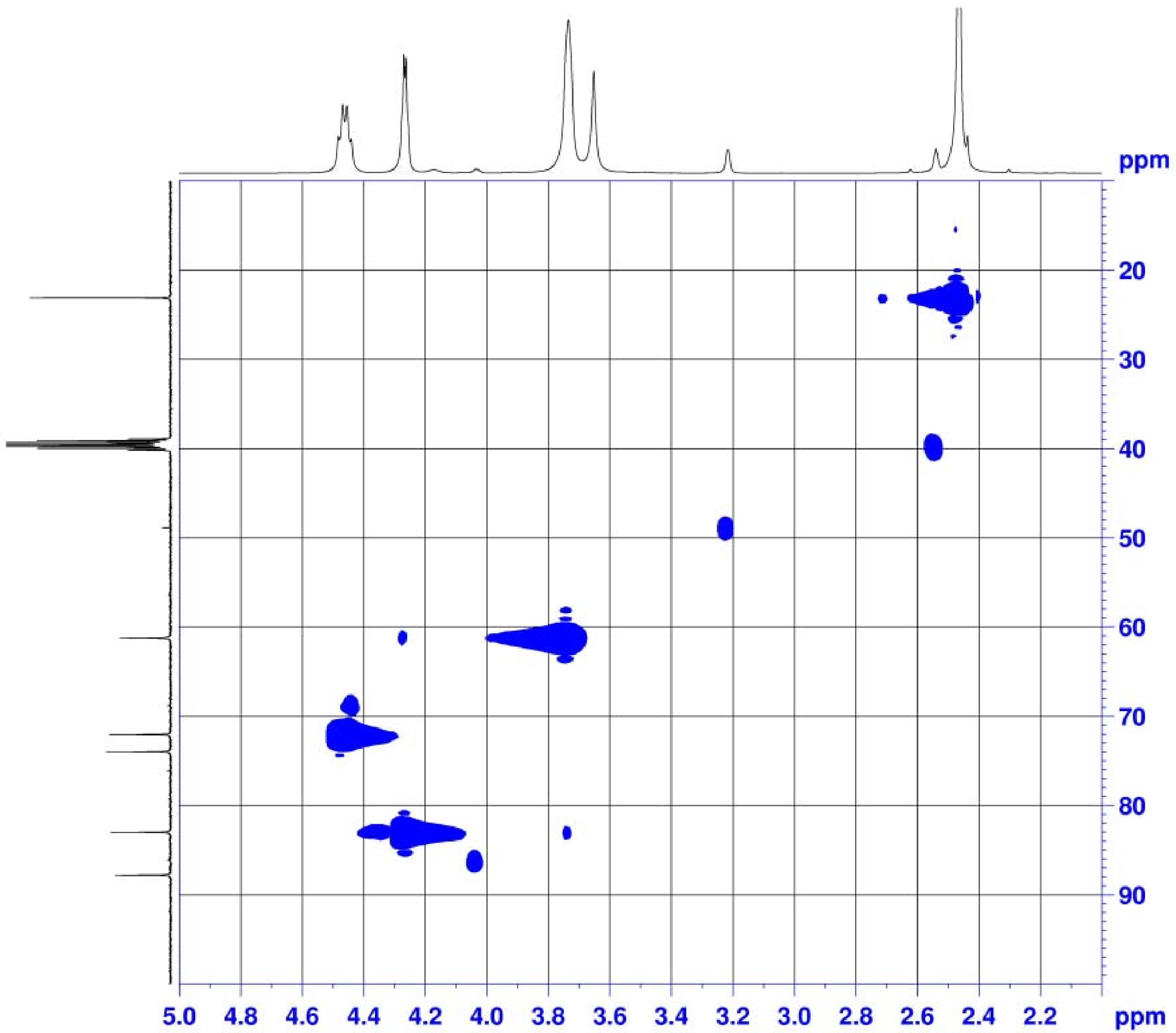

HSQC (400 MHz) spectrum of **1** in DMSO-d_6

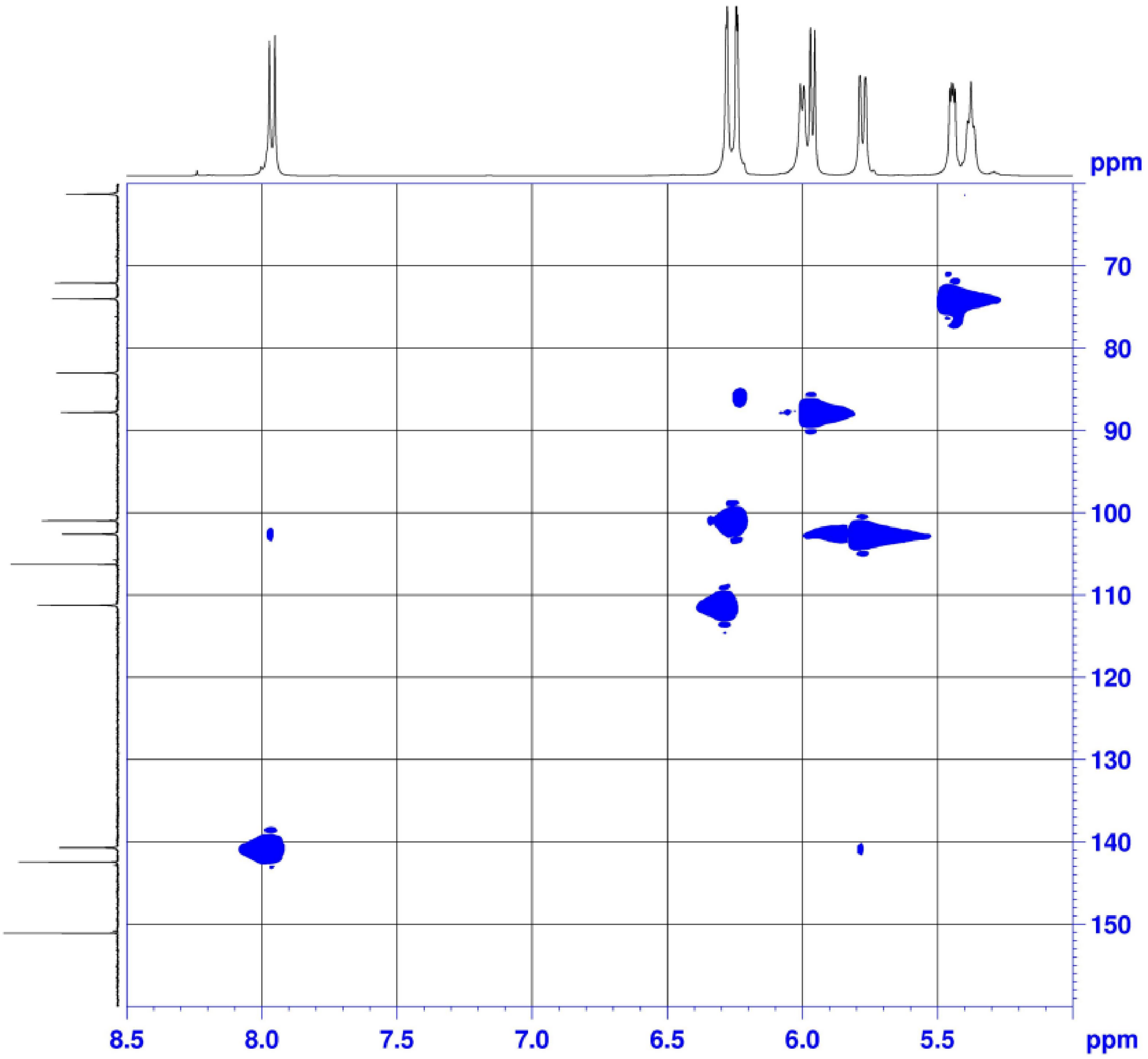

HMBC (400 MHz) spectrum of **1** in DMSO-d_6

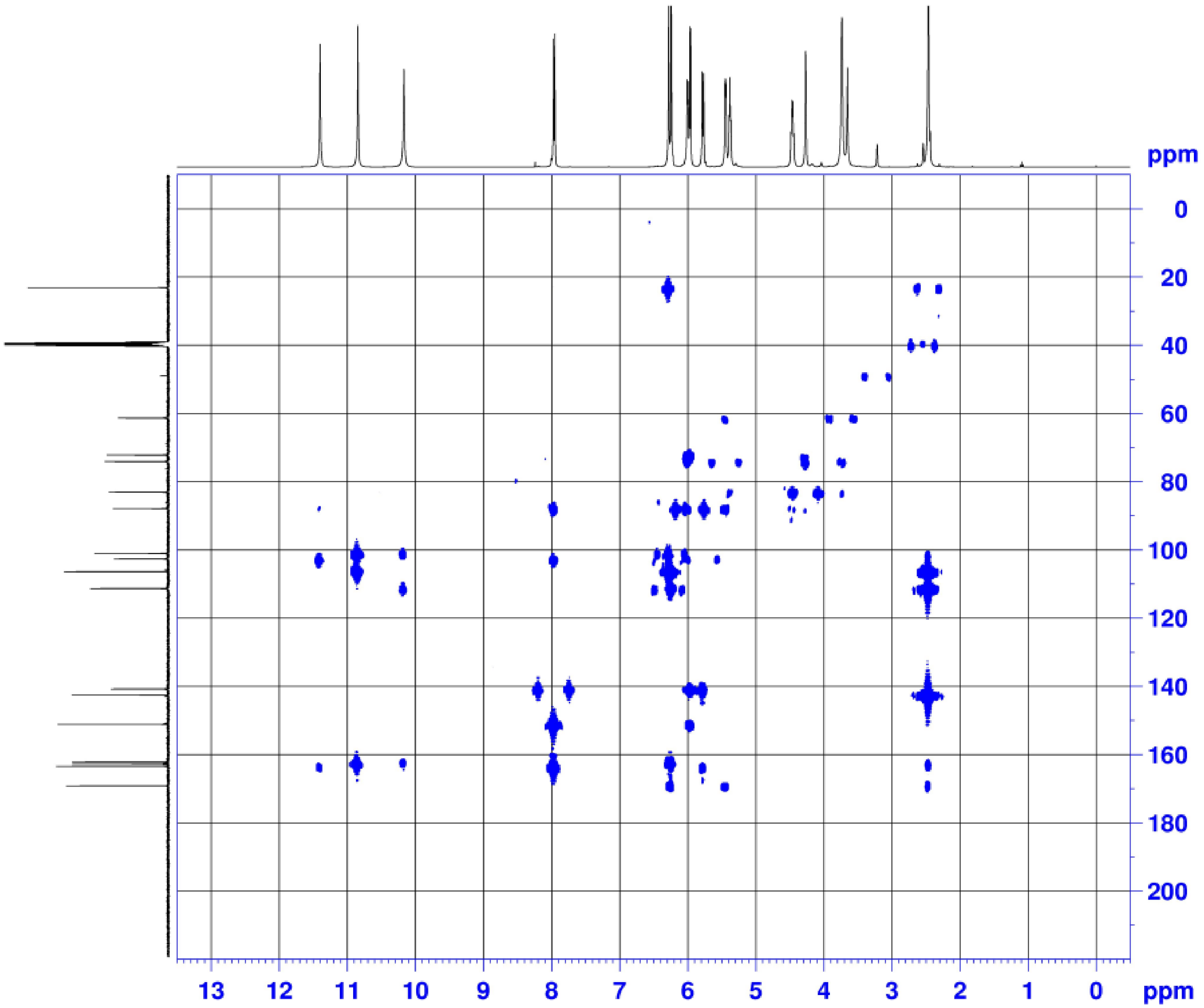

NAME	WBB-145-D
EXPNO	5
PROCNO	1
Date_	20210622
Time	22.31 h
INSTRUM	spect
PROBHD	Z108618_0231 (
PULPROG	hmbcgpndqf
TD	1024
SOLVENT	DMSO
NS	2
DS	16
SWH	6009.615 Hz
FIDRES	11.737530 Hz
AQ	0.0852468 sec
RG	2050
DW	83.200 usec
DE	6.50 usec
TE	297.3 K
CNST13	8.0000000
D0	0.00000300 sec
D1	1.50000000 sec
D6	0.06250000 sec
D16	0.00020000 sec
IN0	0.00002070 sec
ND0	2
TD	128
SFO1	100.6394 MHz
FIDRES	188.707733 Hz
SW	240.011 ppm
FnMODE	QF
SI	1024
SF	400.1999826 MHz
WDW	SINE
SSB	0
LB	0.00 Hz
GB	0
PC	1.40
SI	1024
MC2	QF
SF	100.6303448 MHz
WDW	SINE
SSB	0
LB	0.00 Hz
GB	0

HMBC (400 MHz) spectrum of **1** in DMSO-d_6

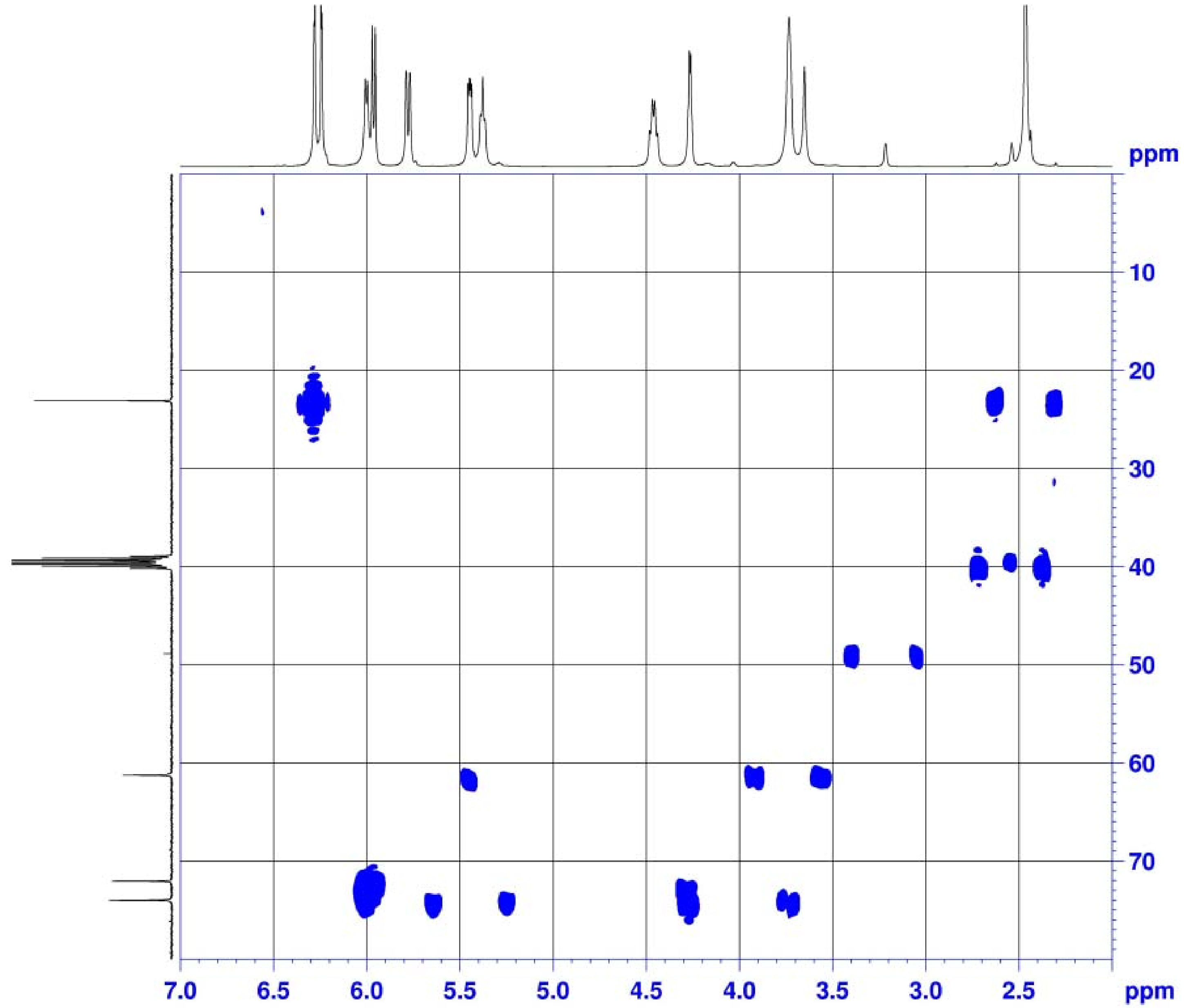

HMBC (400 MHz) spectrum of **1** in DMSO-d_6

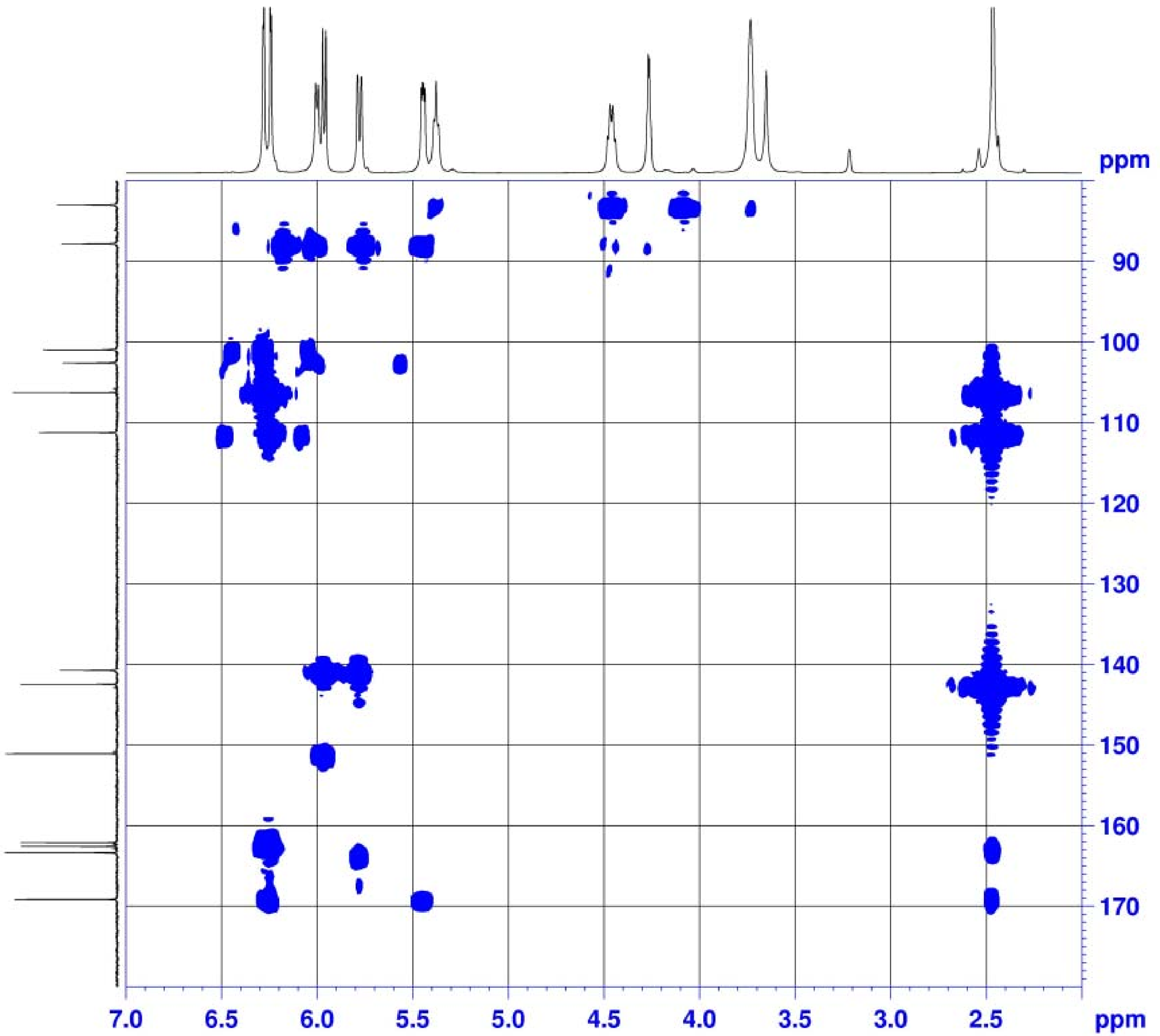

HMBC (400 MHz) spectrum of **1** in DMSO-d_6

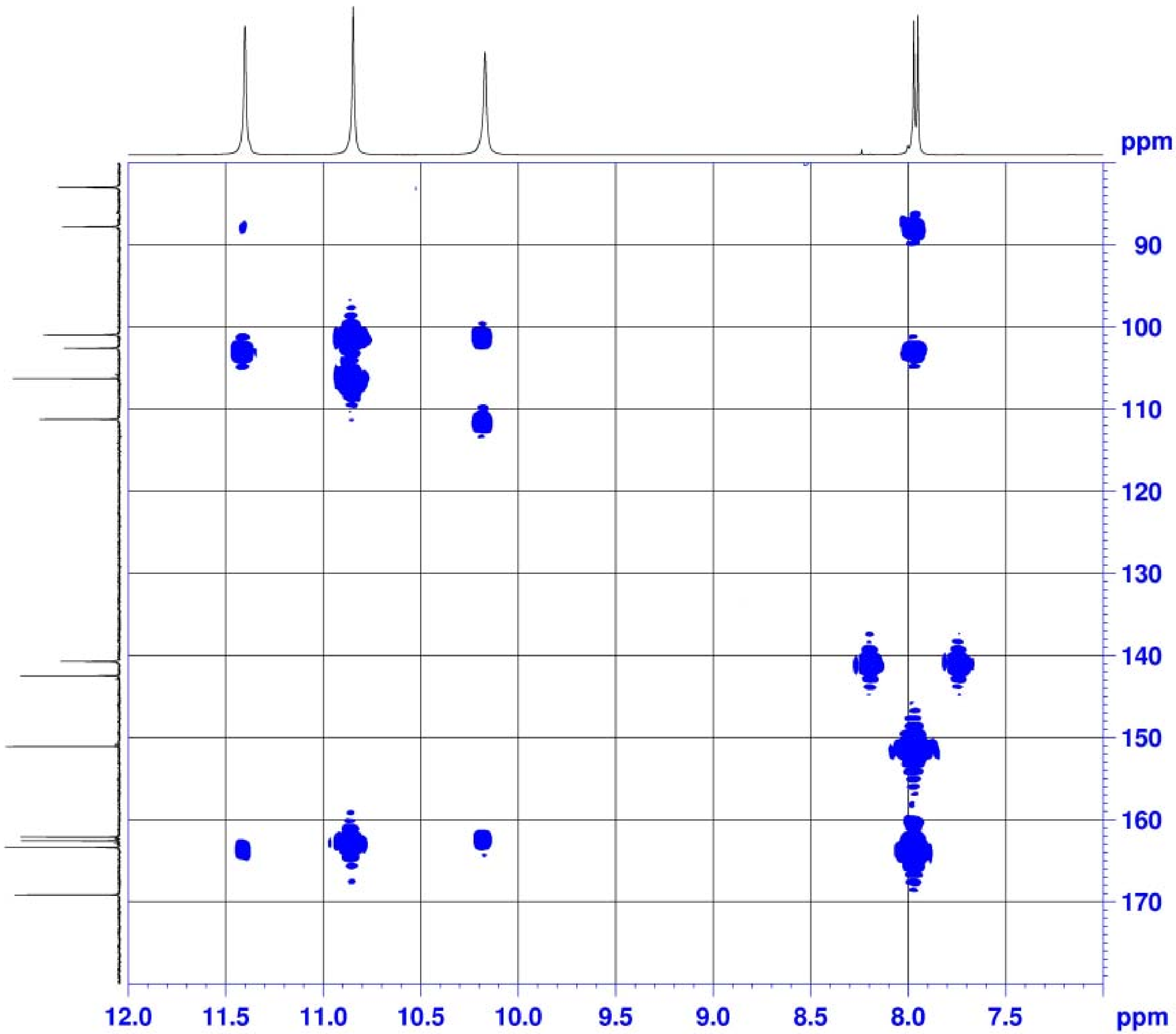

NOESY (400 MHz) spectrum of **1** in DMSO-d_6

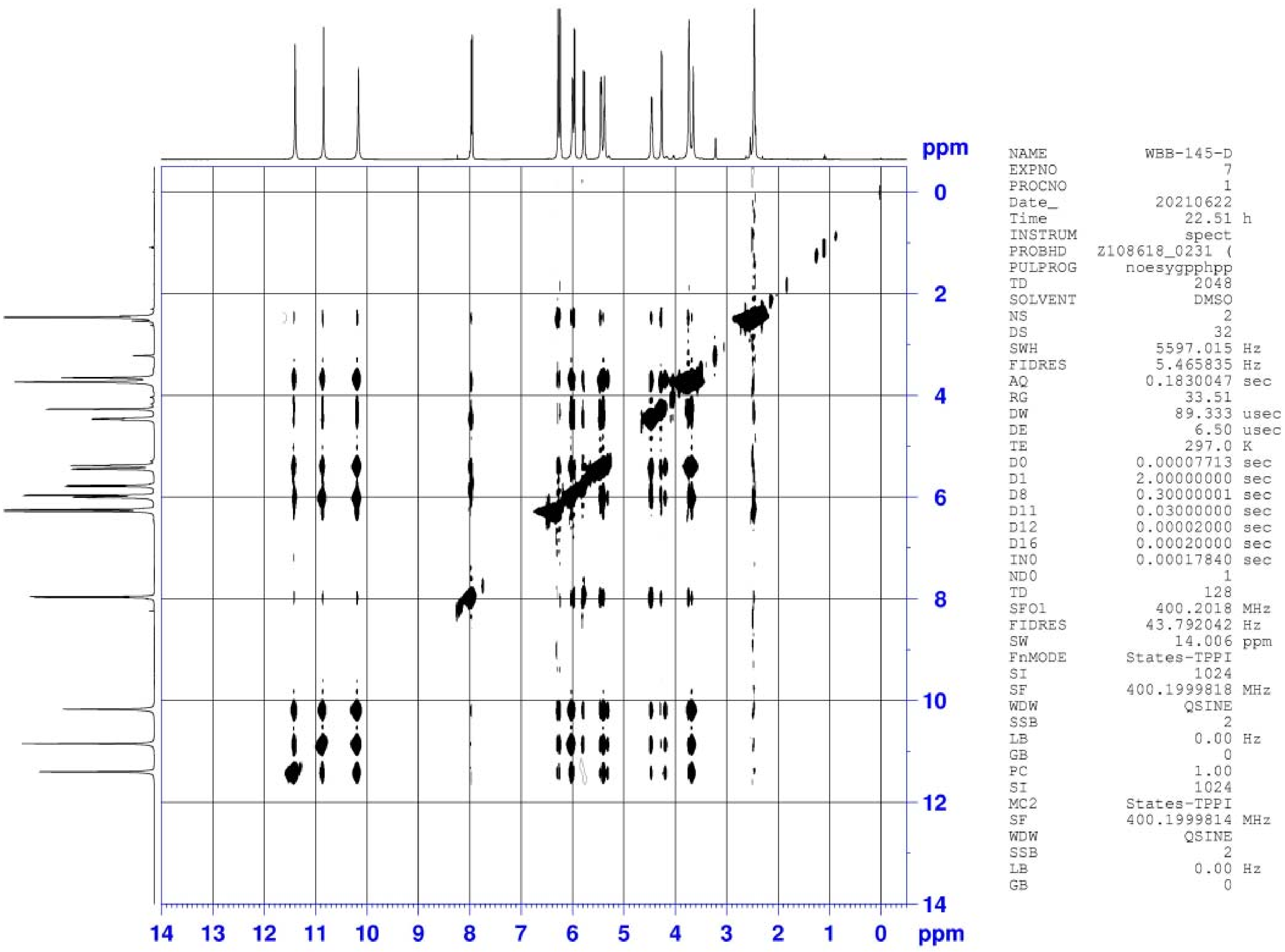

NOESY (400 MHz) spectrum of **1** in DMSO-d_6

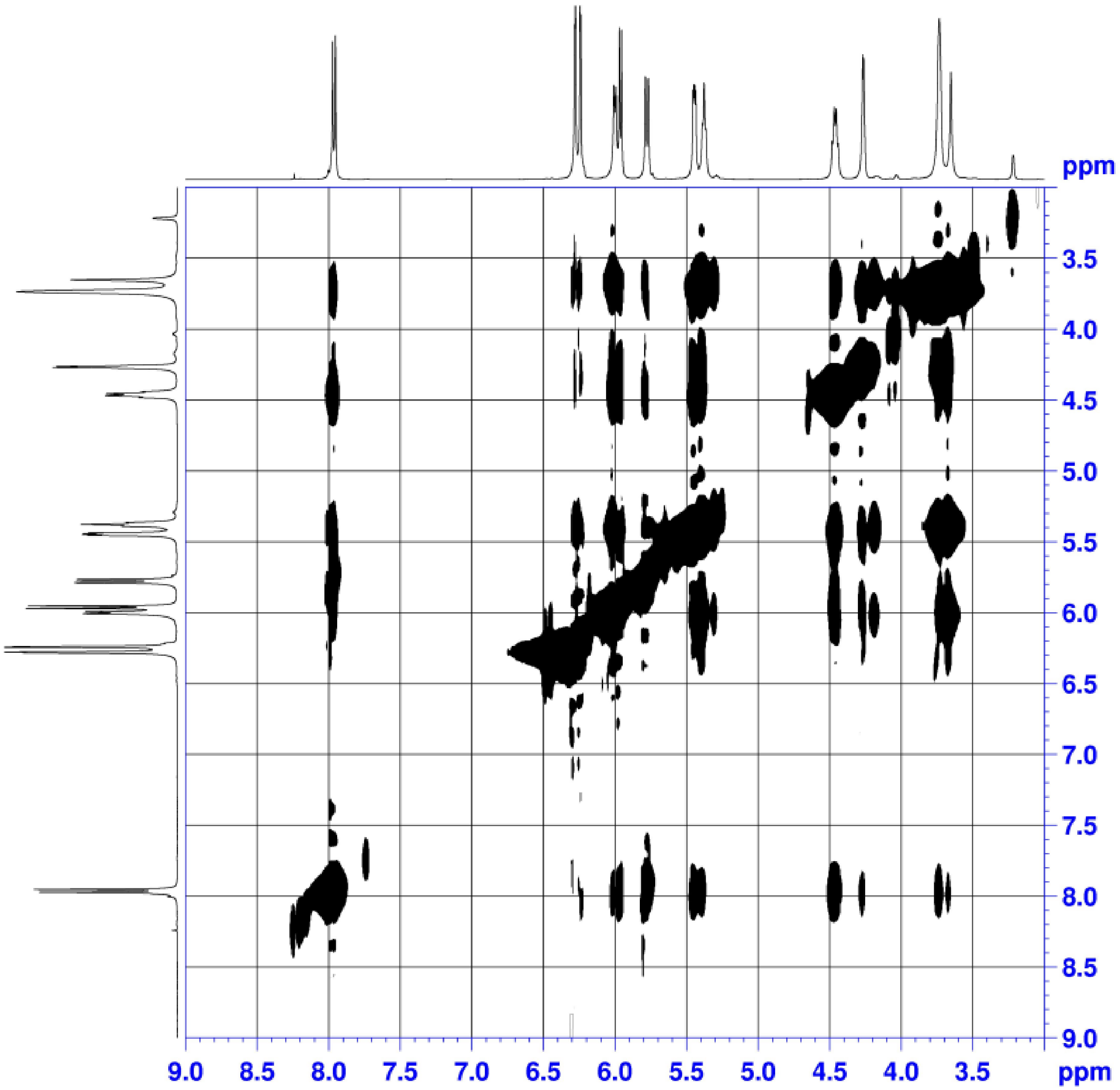

HR-ESIMS for **2**

Mass Spectrum SmartFormula Report

Analysis Info

Analysis Name	D:\Data\MS\data\202104\renyanxia_W_pos_65_01_10385.d	Acquisition Date	4/1/2021 5:30:07 PM
Method	LC_Direct Infusion_pos_70-500mz.m	Operator	SCSIO
Sample Name	renyanxia_W_pos	Instrument	maXis 255552.00029
Comment			

Acquisition Parameter

Source Type	ESI	Ion Polarity	Positive	Set Nebulizer	0.4 Bar
Focus	Active	Set Capillary	4500 V	Set Dry Heater	180 °C
Scan Begin	70 m/z	Set End Plate Offset	-500 V	Set Dry Gas	4.0 l/min
Scan End	1500 m/z	Set Charging Voltage	0 V	Set Divert Valve	Waste
		Set Corona	0 nA	Set APCI Heater	0 °C

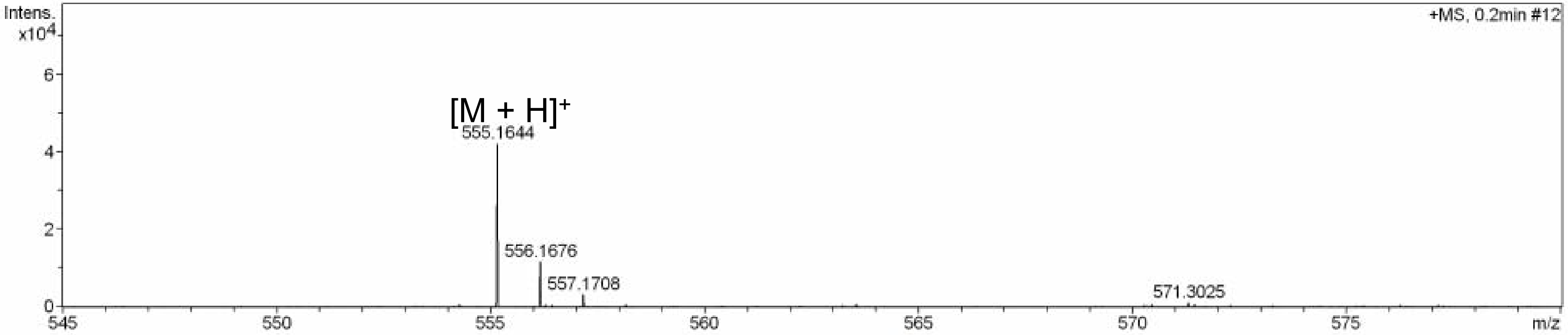

Meas. m/z	#	Ion Formula	Score	m/z	err [ppm]	err [mDa]	mSigma	rdb	e⁻ Conf	N-Rule
555.1644	1	C32H27O9	100.00	555.1650	-1.1	-0.6	37.1	19.5	even	ok

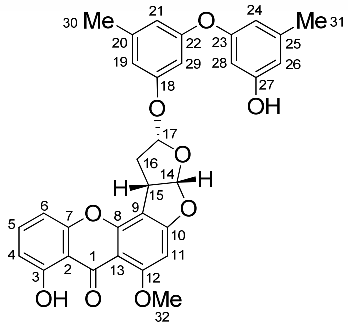

2

HR-ESIMS for **2**

Generic Display Report

Analysis Info

Analysis Name	D:\Data\MS\data\202104\renyanxia_W_pos_65_01_10385.d	Acquisition Date	4/1/2021 5:30:07 PM
Method	LC_Direct Infusion_pos_70-500mz.m	Operator	SCSIO
Sample Name	renyanxia_W_pos	Instrument	maXis
Comment			

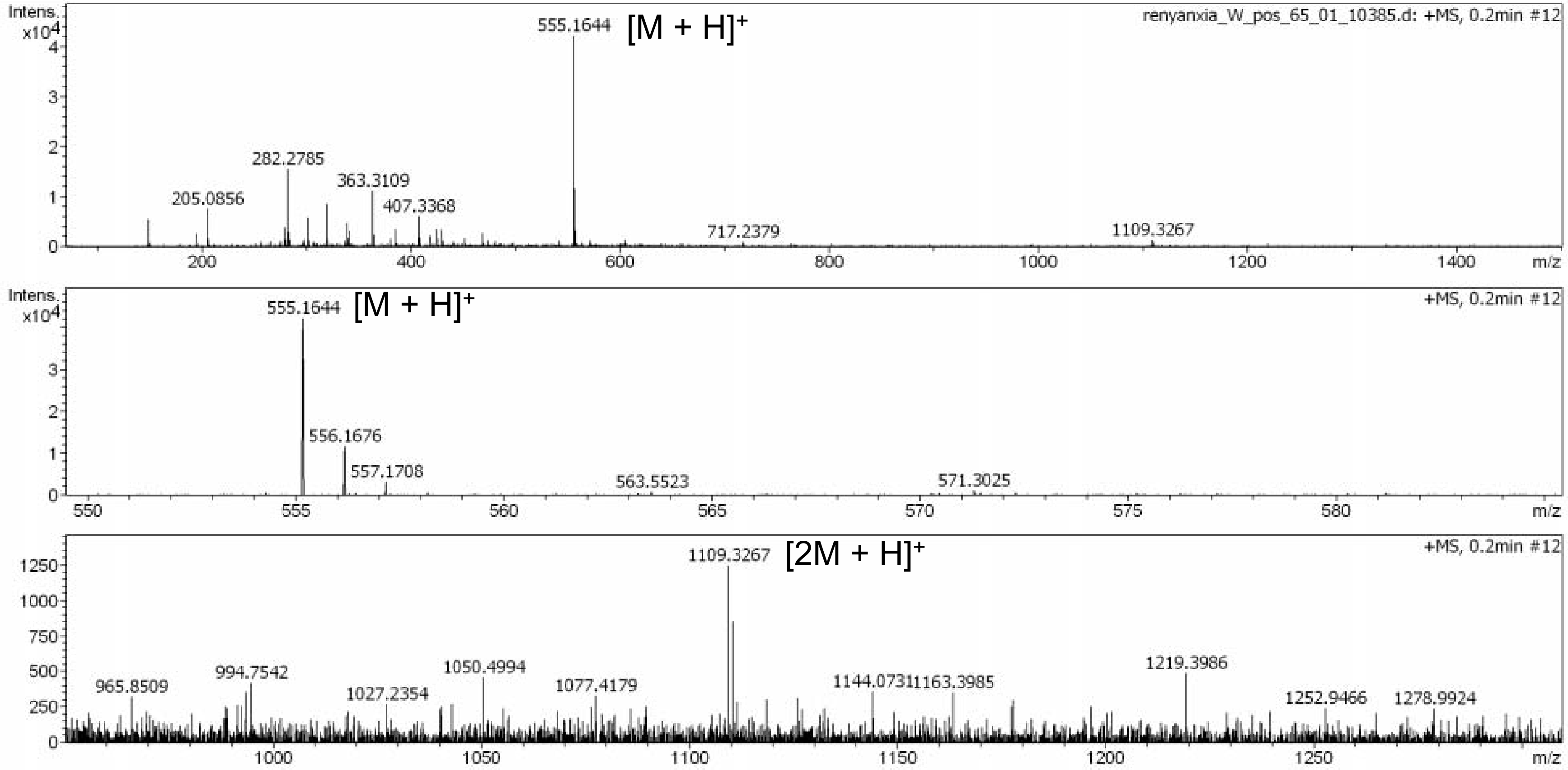

Bruker Compass DataAnalysis 4.1 printed: 4/1/2021 5:35:54 PM by: SCSIO Page 1 of 1

^{1}H (700 MHz) NMR spectrum of **2** in $CDCl_3$

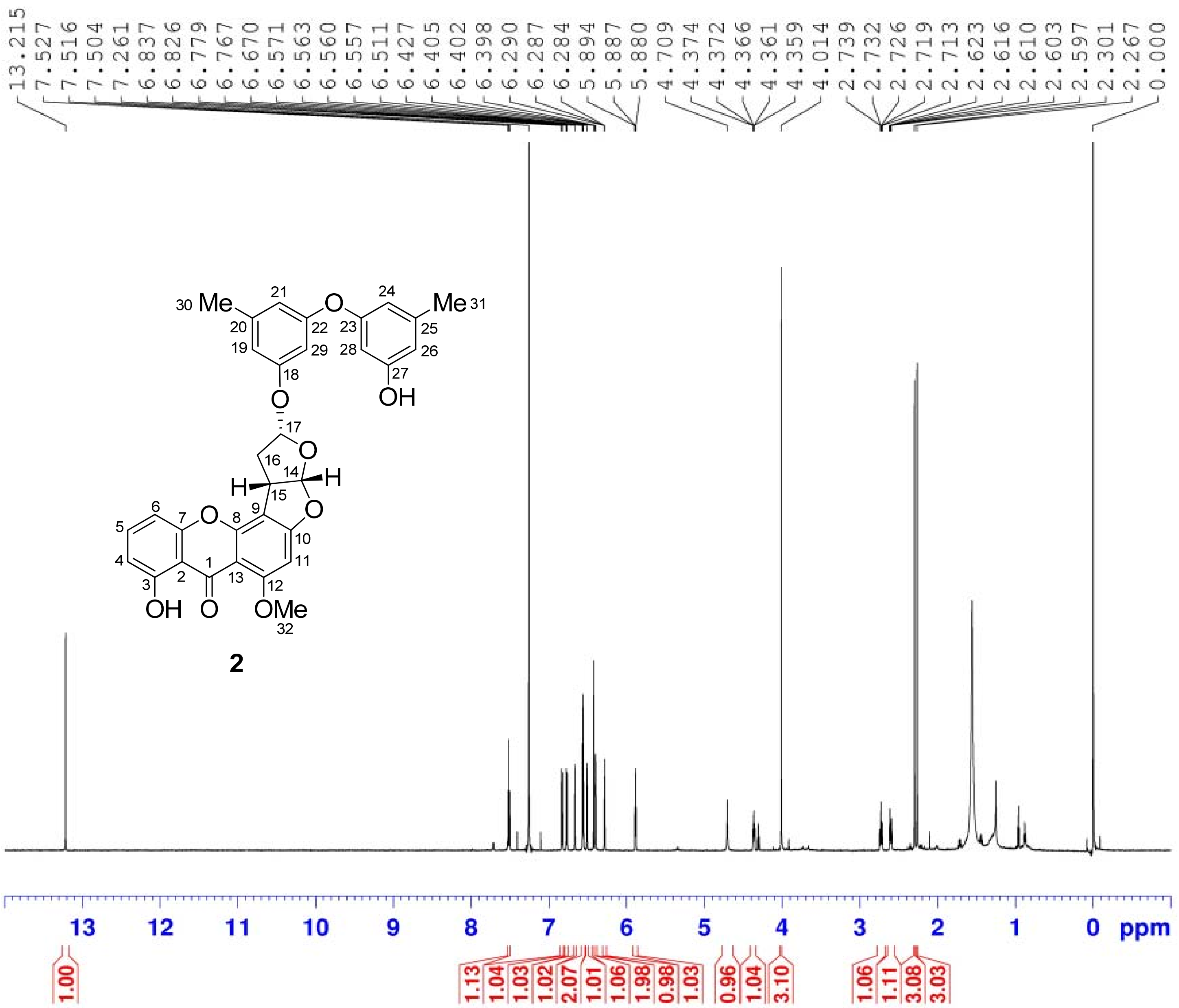

NAME	wubibbib-WBB-42-22-9
EXPNO	10
PROCNO	1
Date_	20210207
Time	15.25 h
INSTRUM	spect
PROBHD	Z120187_0028 (
PULPROG	zg30
TD	65536
SOLVENT	CDCl3
NS	8
DS	2
SWH	14097.744 Hz
FIDRES	0.430229 Hz
AQ	2.3243935 sec
RG	4.87
DW	35.467 usec
DE	10.00 usec
TE	298.0 K
D1	1.00000000 sec
TD0	1
SFO1	700.1849013 MHz
NUC1	1H
P1	7.55 usec
SI	65536
SF	700.1800174 MHz
WDW	EM
SSB	0
LB	0.30 Hz
GB	0
PC	1.00

^1H (700 MHz) NMR spectrum of **2** in $CDCl_3$

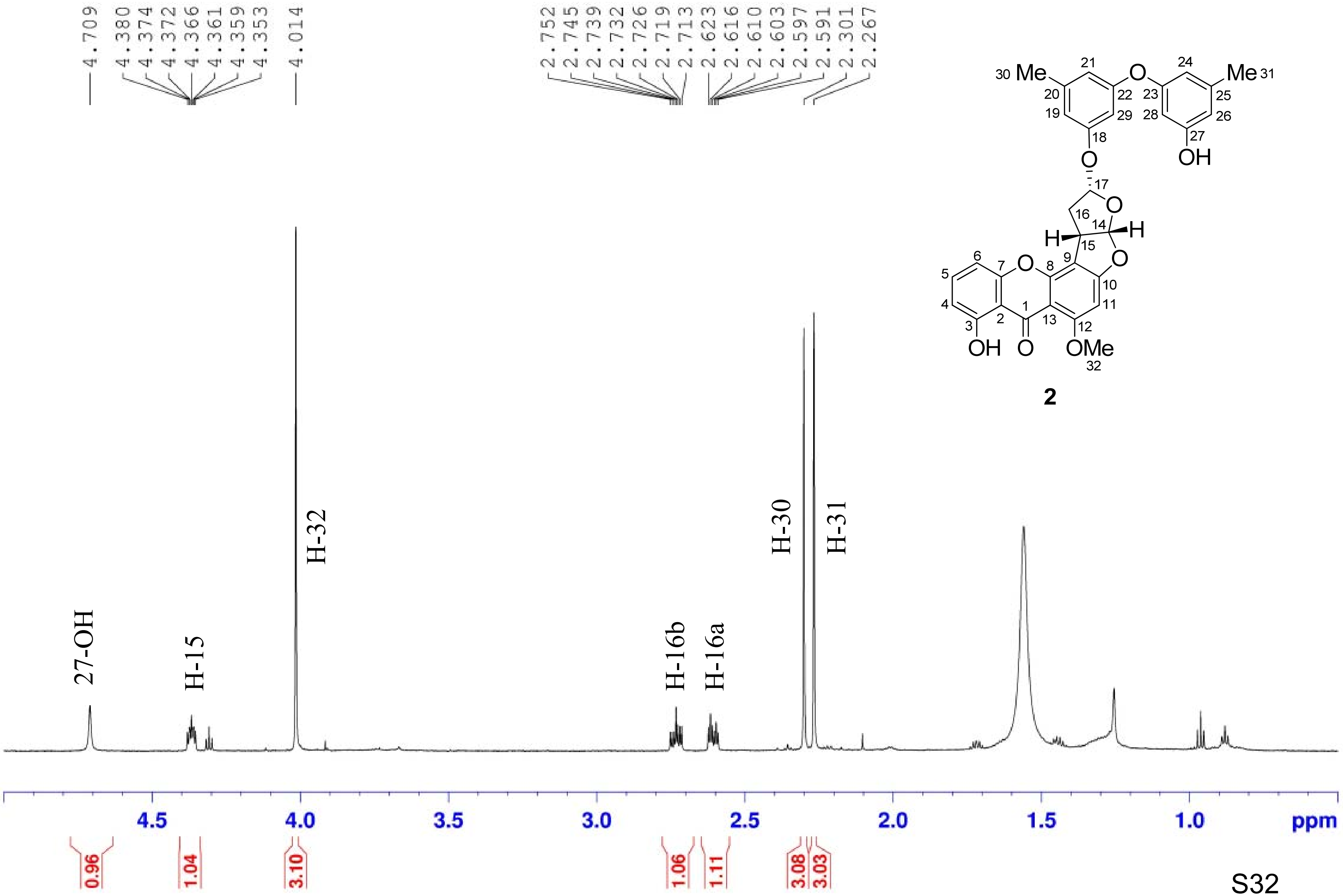

2

^{1}H (700 MHz) NMR spectrum of **2** in $CDCl_3$

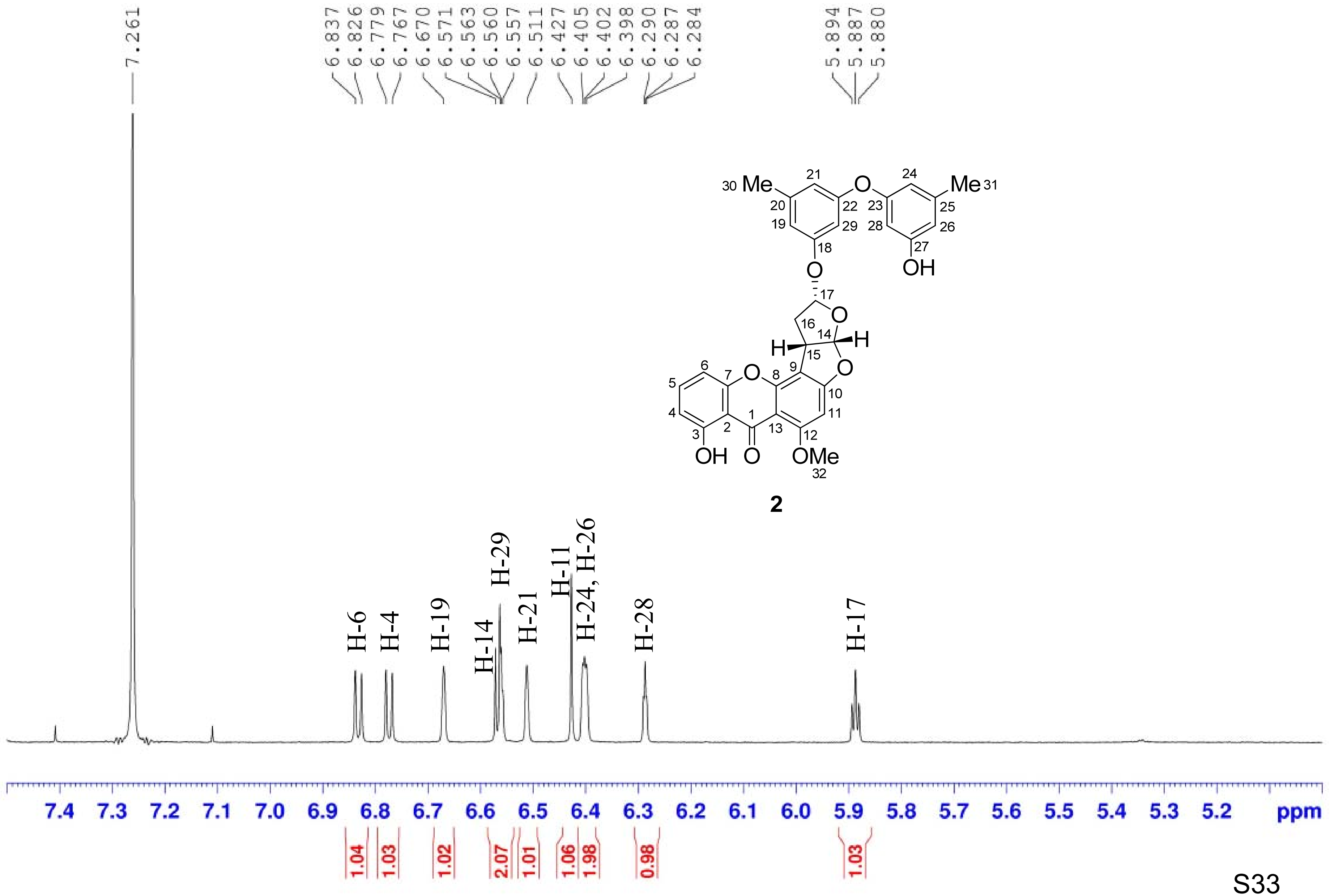

^{1}H (700 MHz) NMR spectrum of **2** in $CDCl_3$

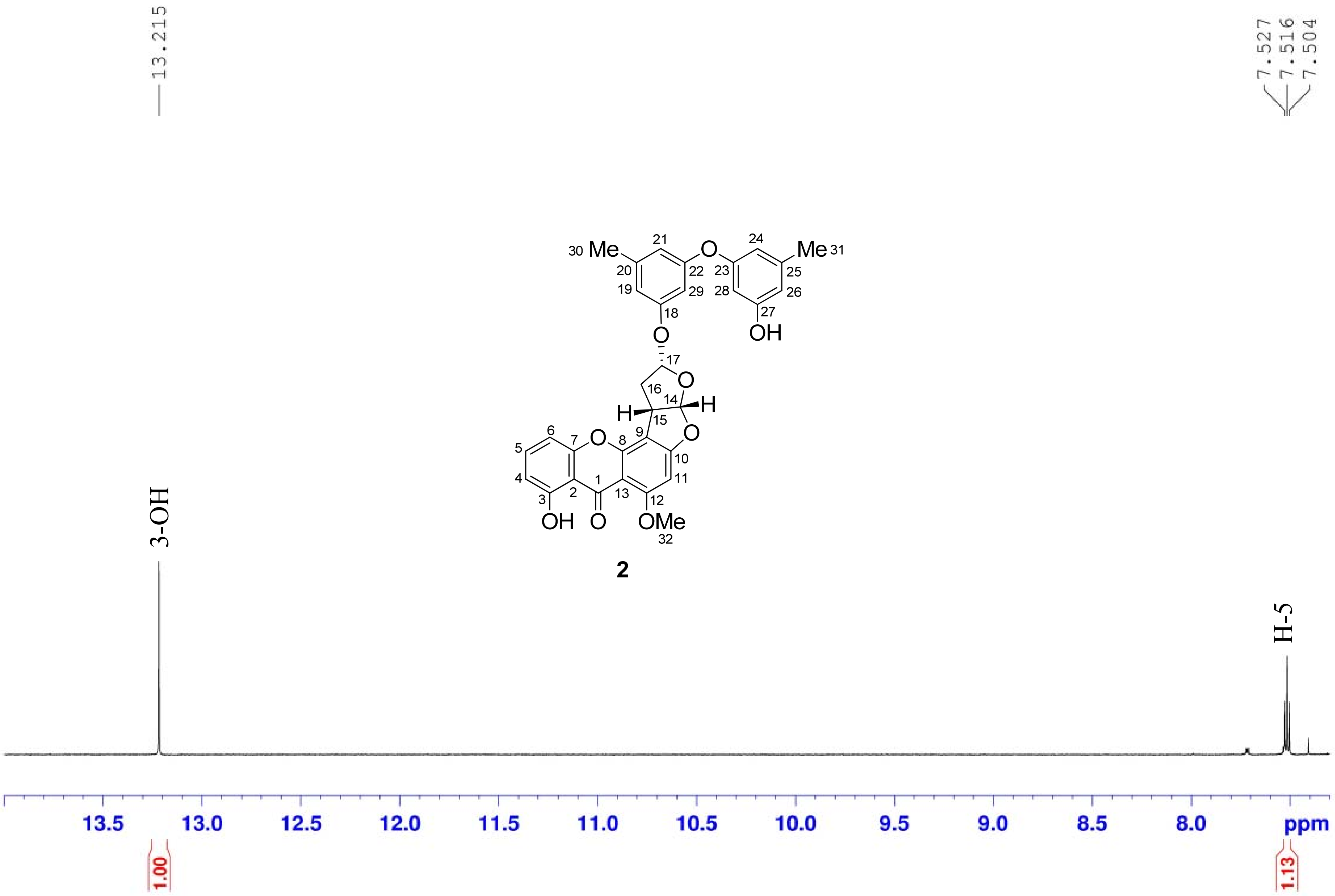

^{13}C (175 MHz) NMR spectrum of **2** in $CDCl_3$

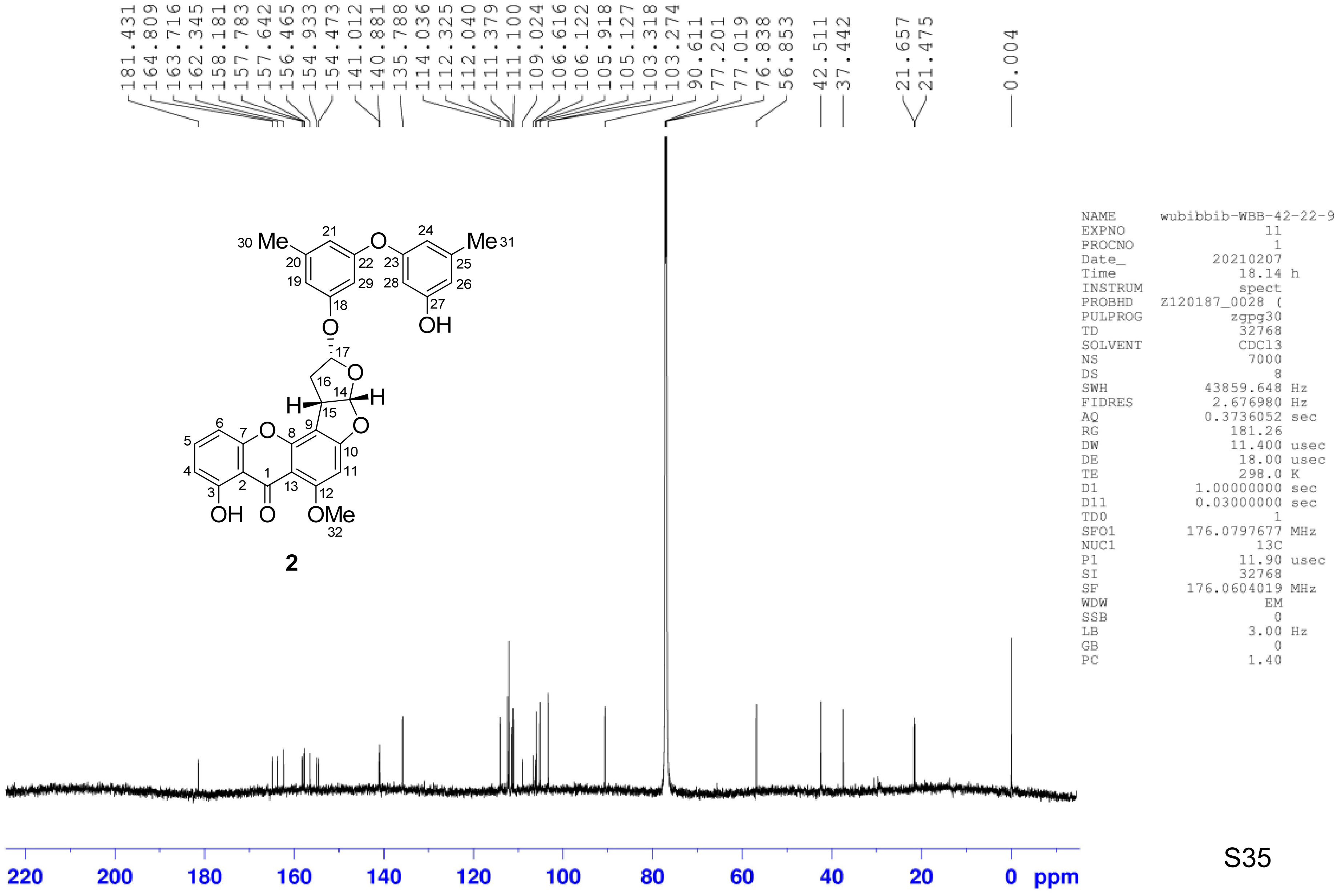

^{13}C (175 MHz) NMR spectrum of **2** in $CDCl_3$

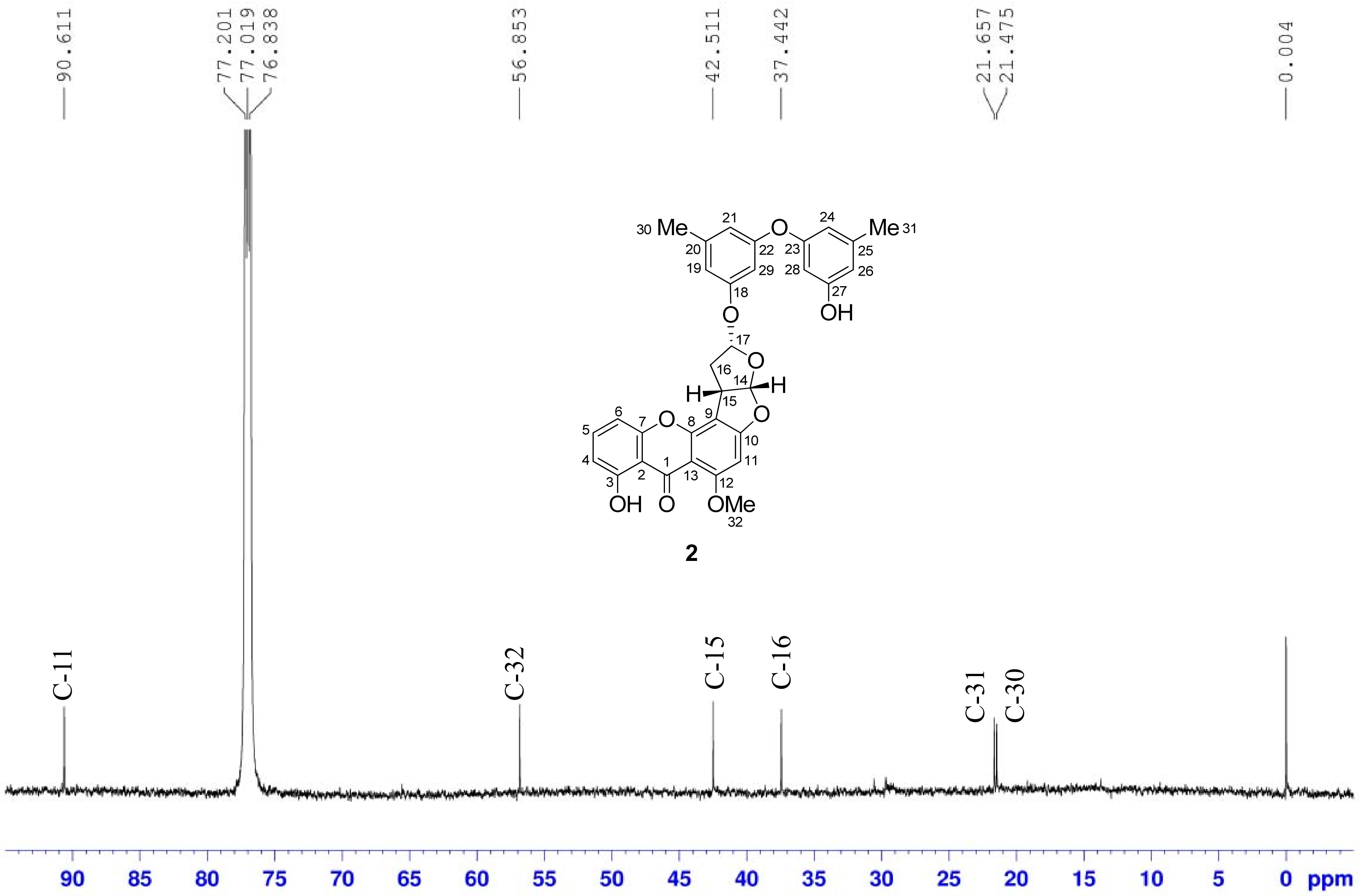

^{13}C (175 MHz) NMR spectrum of **2** in $CDCl_3$

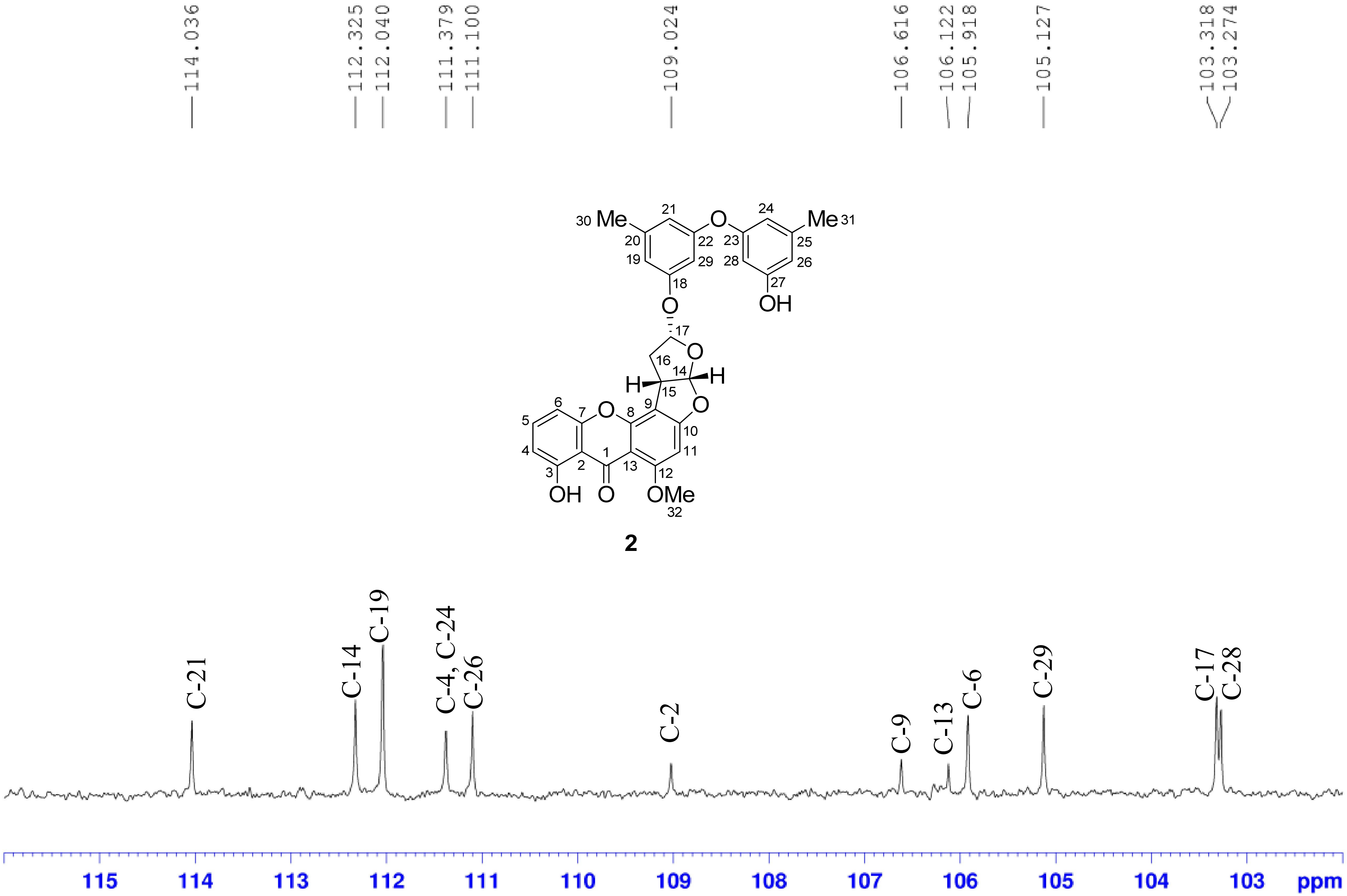

^{13}C (175 MHz) NMR spectrum of **2** in $CDCl_3$

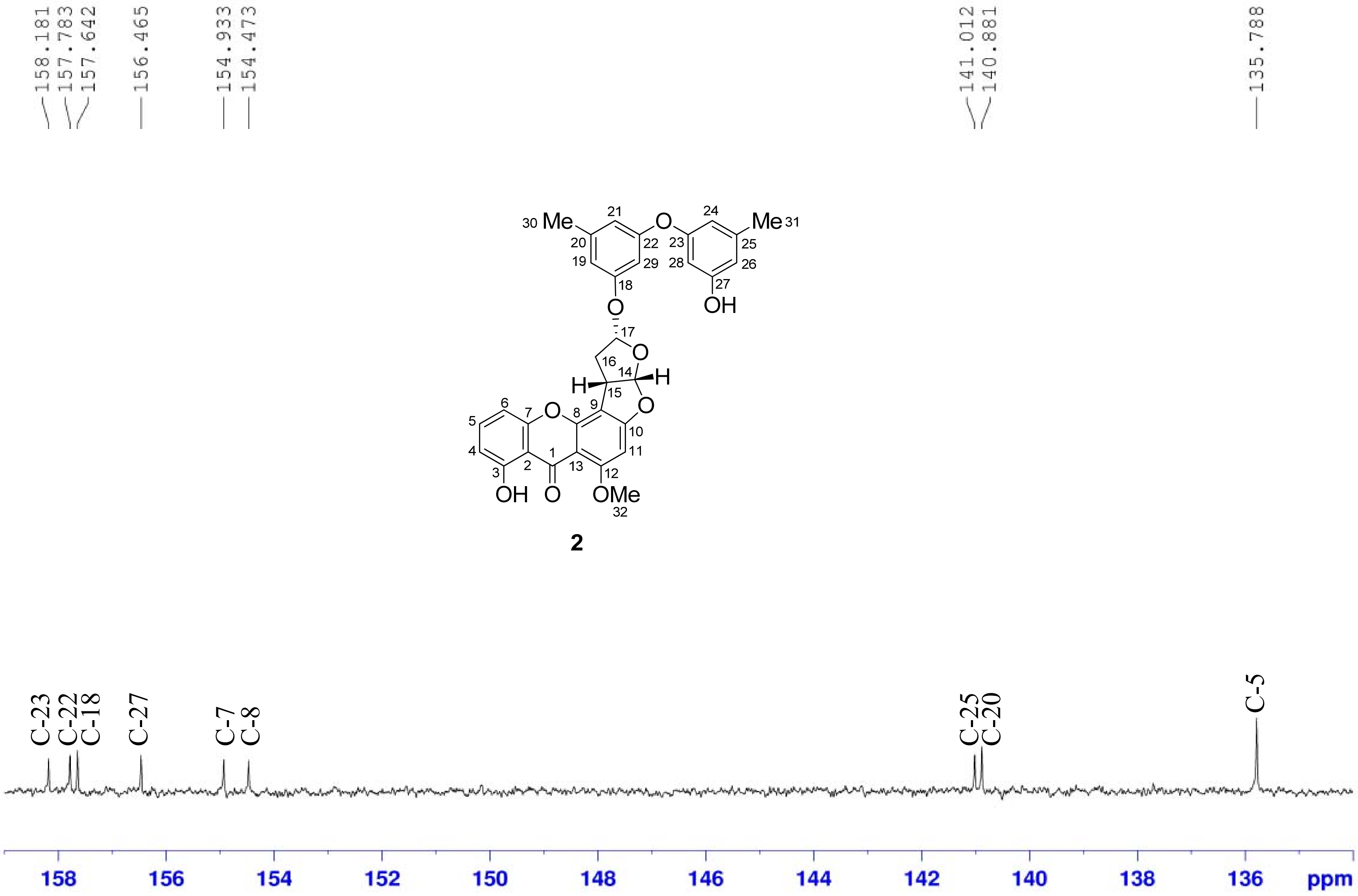

^{13}C (175 MHz) NMR spectrum of **2** in $CDCl_3$

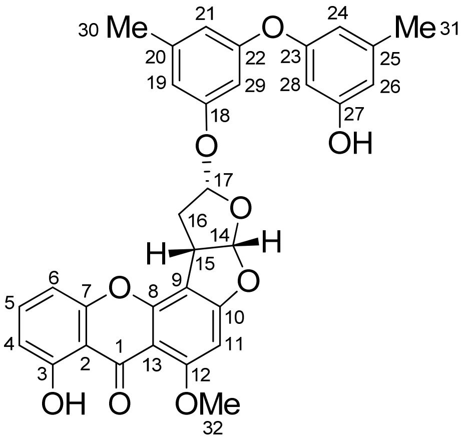

2

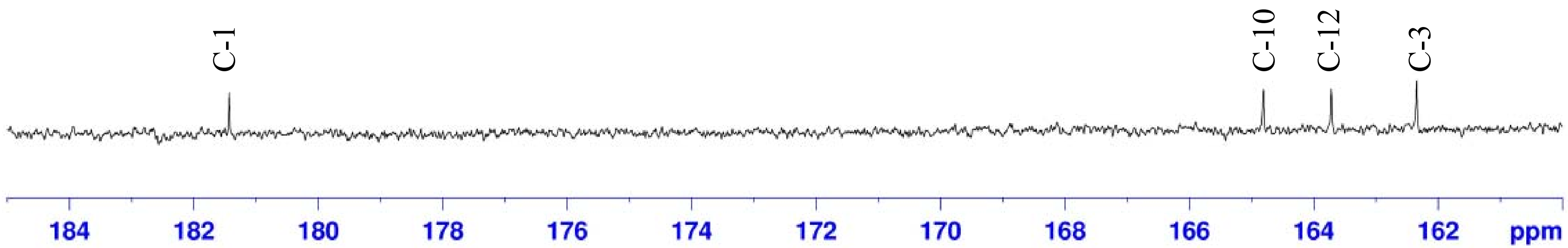

DEPT135 (175 MHz) spectrum of **2** in $CDCl_3$

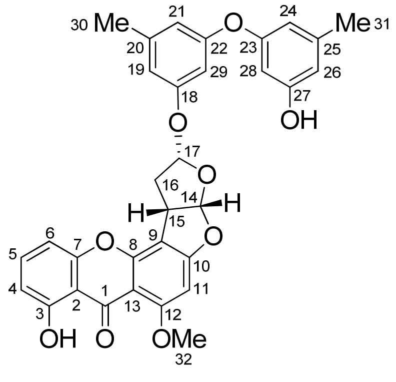

2

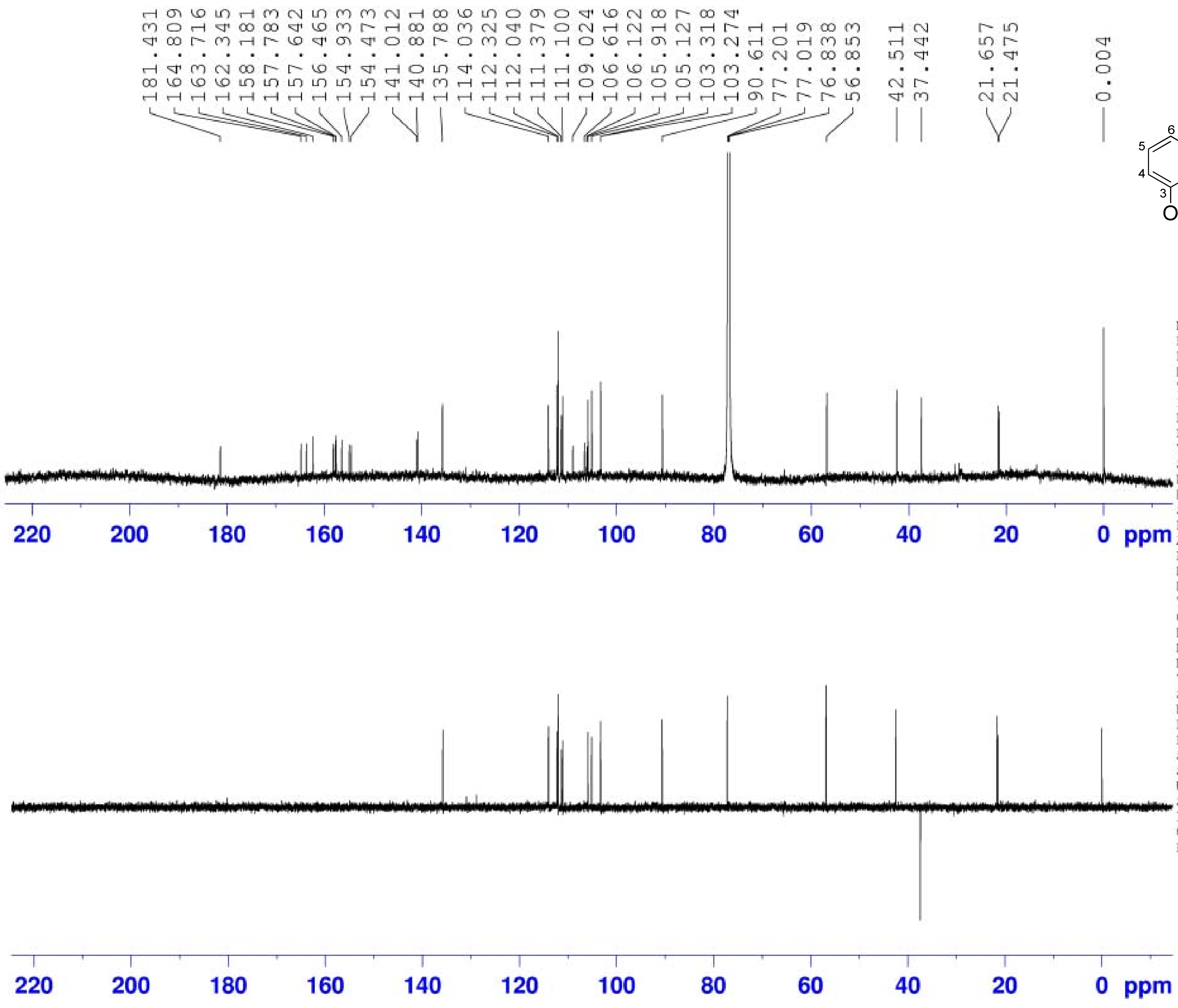

DEPT135 (175 MHz) spectrum of **2** in $CDCl_3$

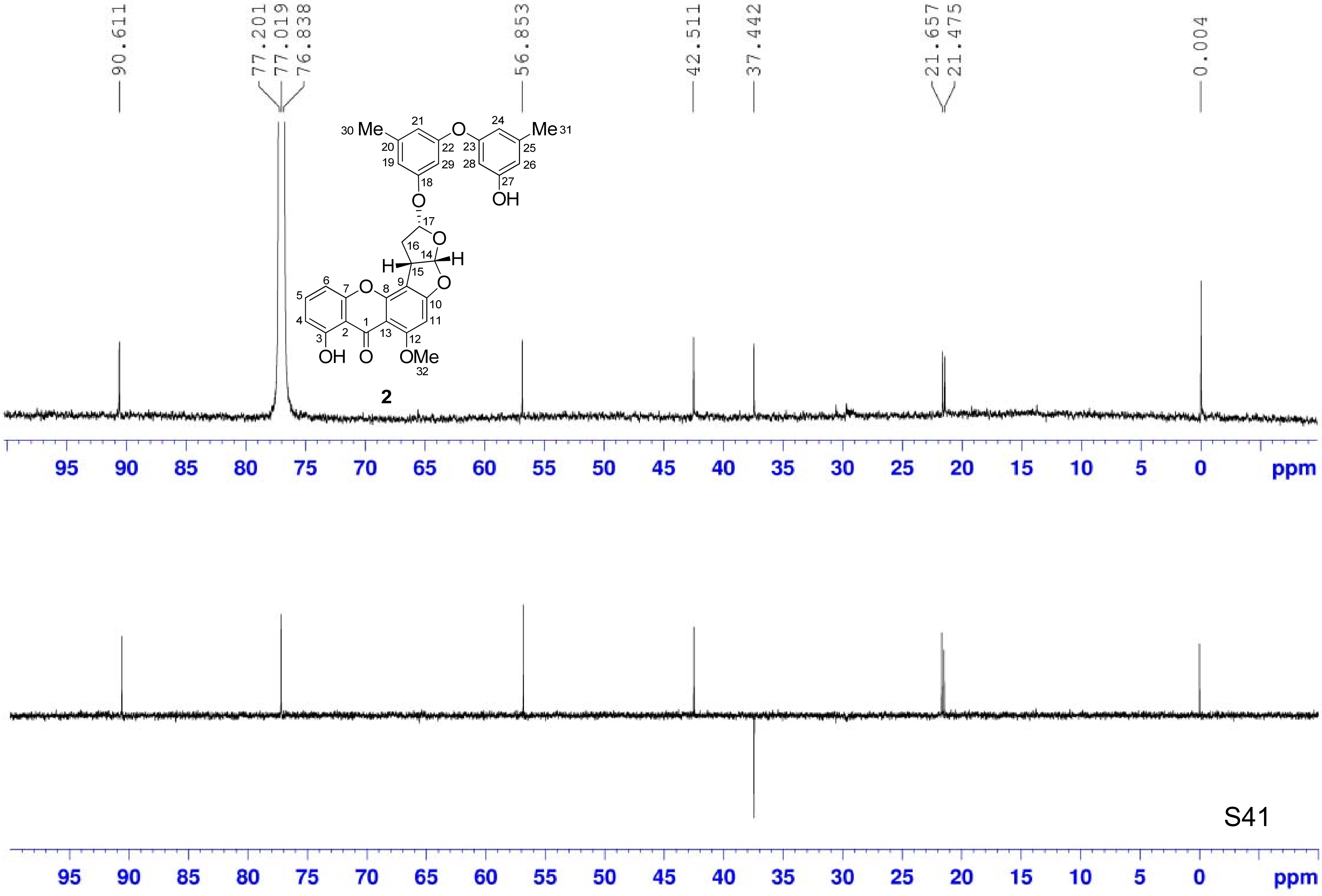

DEPT135 (175 MHz) spectrum of **2** in $CDCl_3$

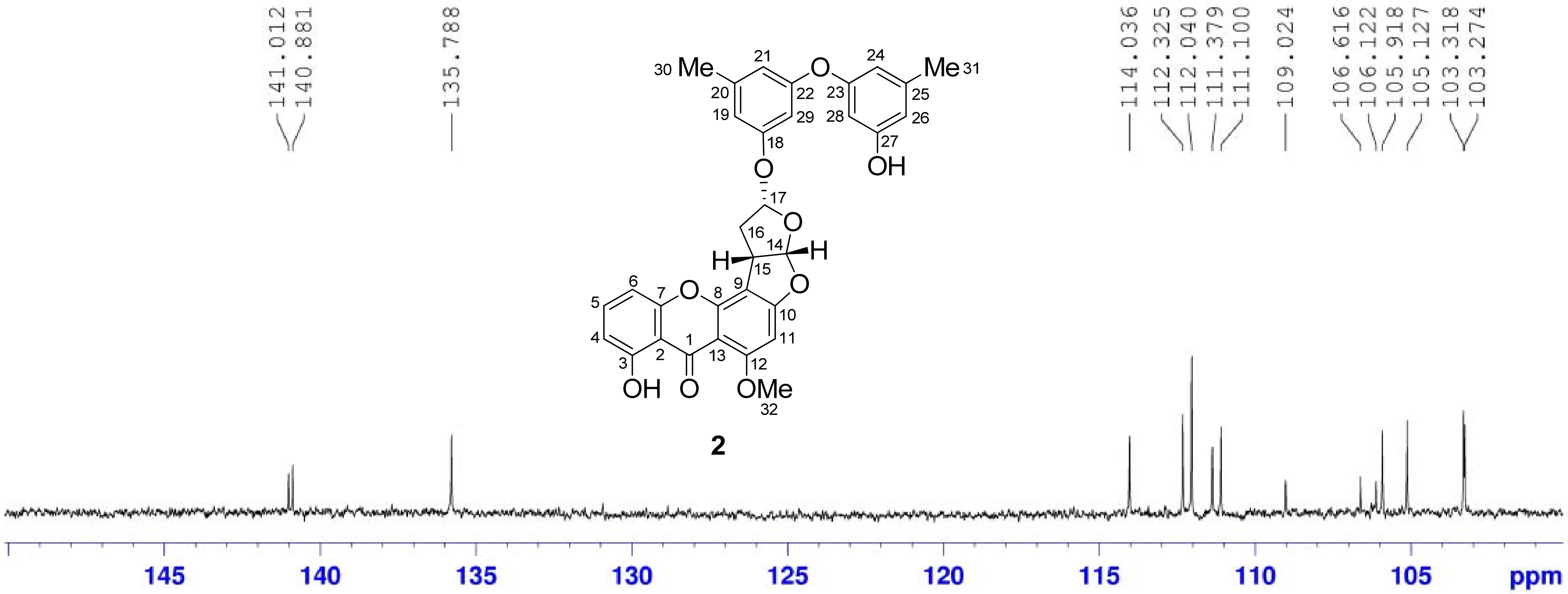

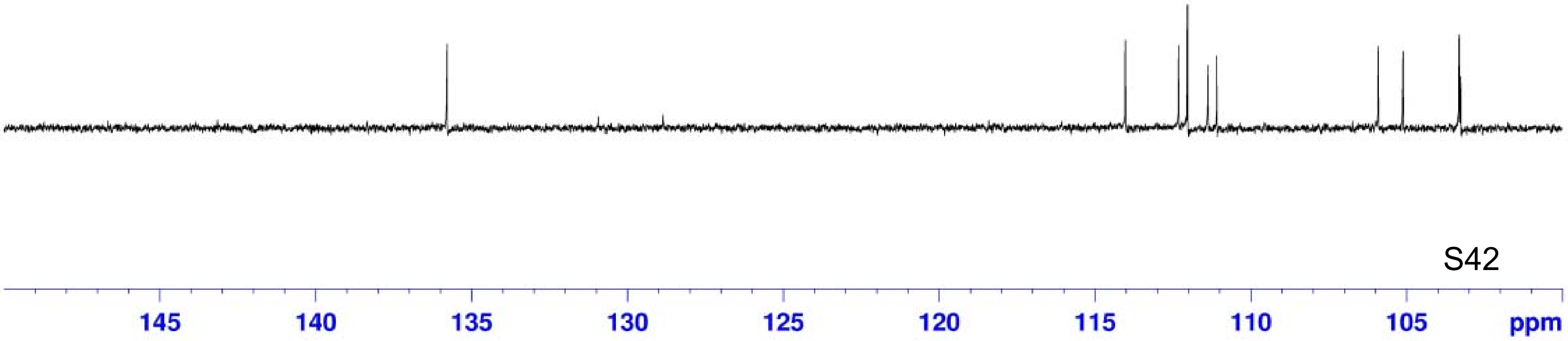

DEPT135 (175 MHz) spectrum of **2** in $CDCl_3$

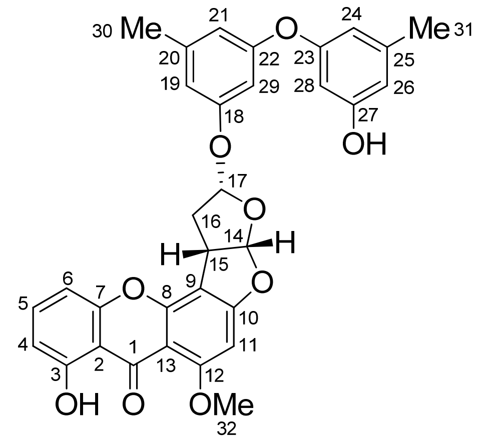

2

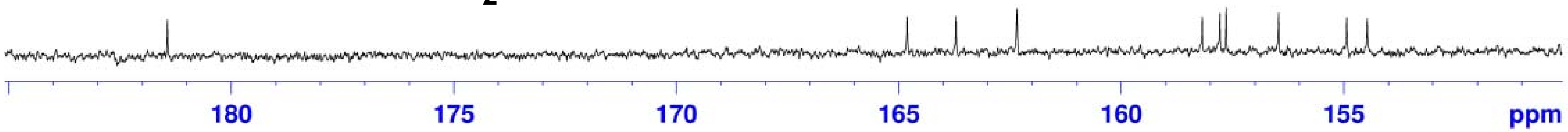

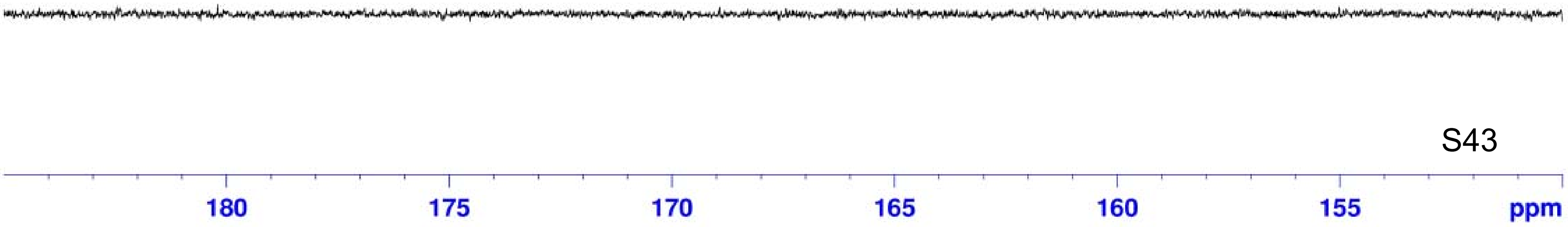

^{1}H–^{1}H COSY (700 MHz) spectrum of **2** in $CDCl_3$

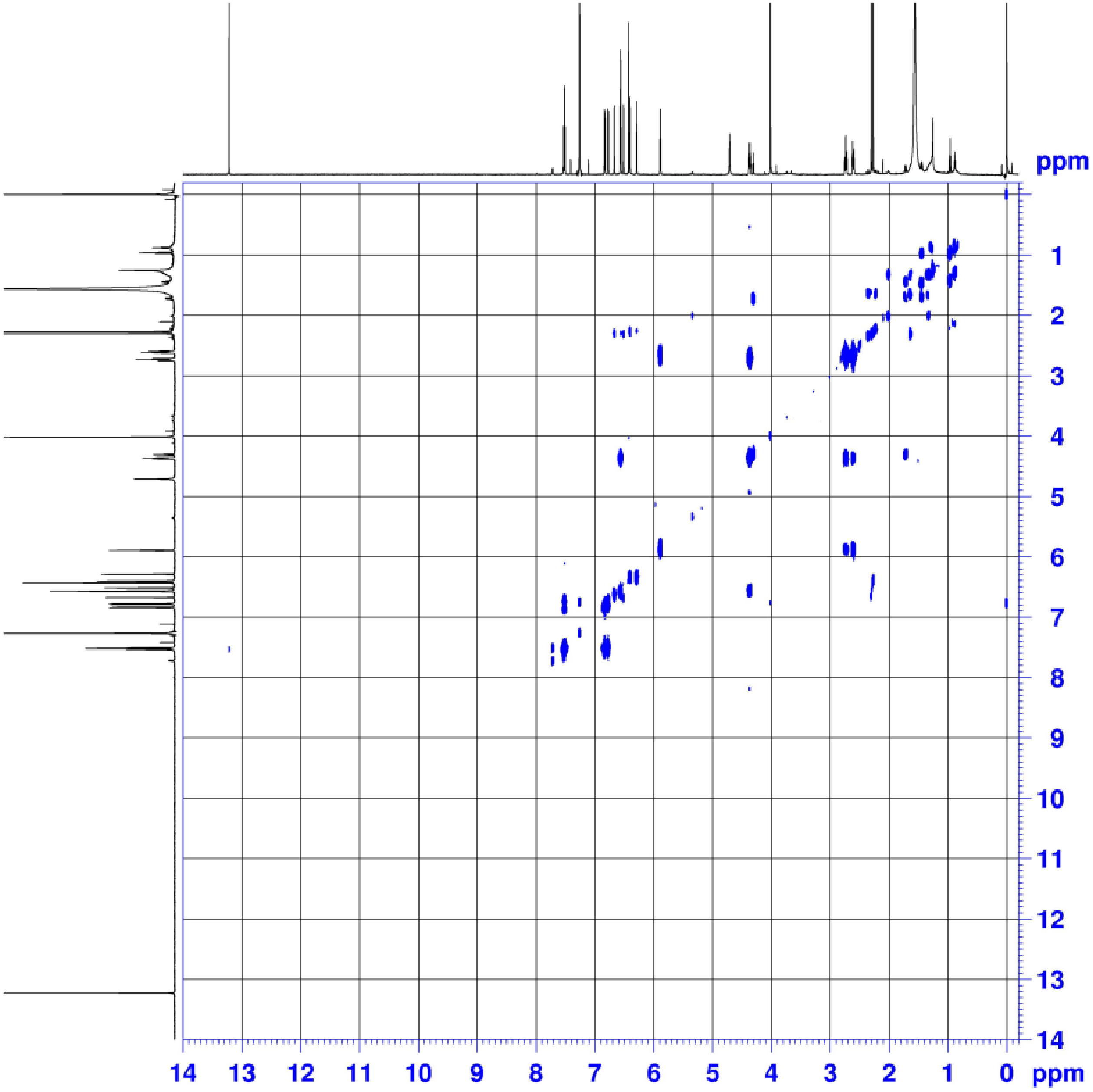

NAME	wubibbib-WBB-42-22-9
EXPNO	13
PROCNO	1
Date_	20210207
Time	19.39 h
INSTRUM	spect
PROBHD	Z120187_0028 (
PULPROG	cosygpmfqf
TD	2048
SOLVENT	CDCl3
NS	24
DS	16
SWH	11520.737 Hz
FIDRES	11.250720 Hz
AQ	0.0889332 sec
RG	181.26
DW	43.400 usec
DE	10.00 usec
TE	298.0 K
D0	0.00000300 sec
D1	1.00000000 sec
D13	0.00000400 sec
D16	0.00020000 sec
IN0	0.00008680 sec
ND0	1
TD	128
SFO1	700.1847 MHz
FIDRES	90.005760 Hz
SW	16.454 ppm
FnMODE	QF
SI	1024
SF	700.1800175 MHz
WDW	SINE
SSB	0
LB	0.00 Hz
GB	0
PC	1.40
SI	1024
MC2	QF
SF	700.1800175 MHz
WDW	SINE
SSB	0
LB	0.00 Hz
GB	0

^{1}H–^{1}H COSY (700 MHz) spectrum of **2** in $CDCl_3$

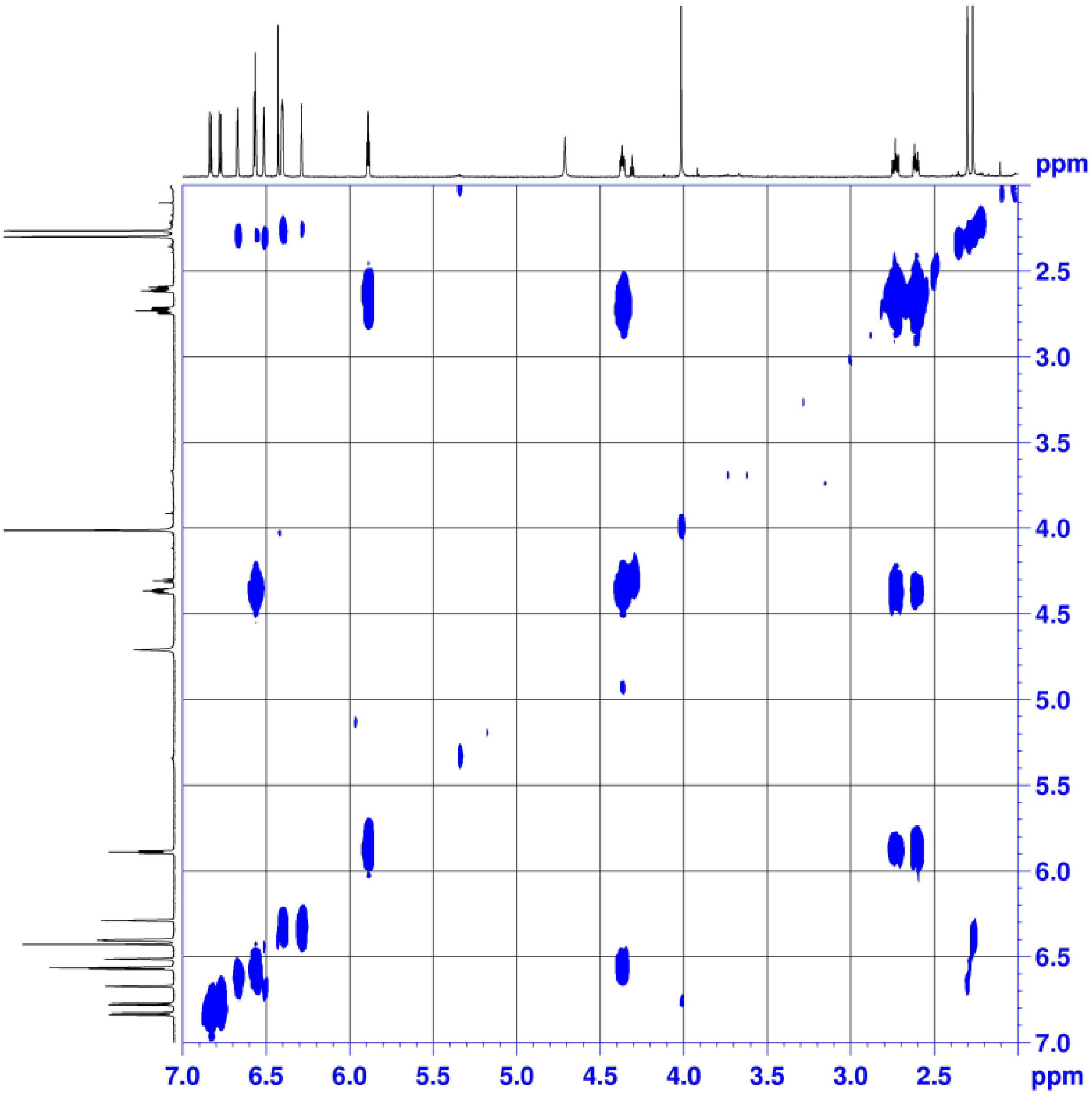

HSQC (700 MHz) spectrum of **2** in $CDCl_3$

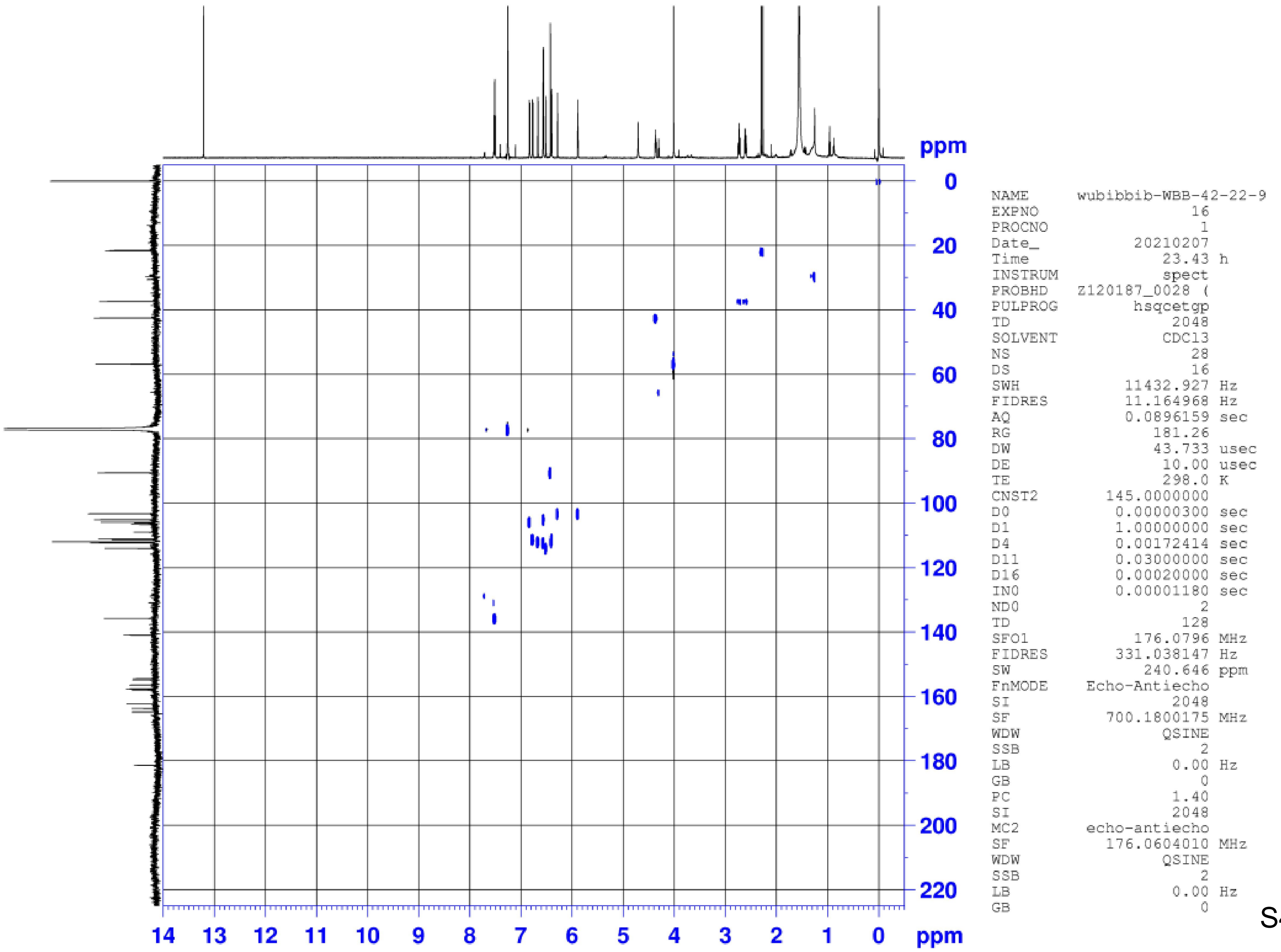

HSQC (700 MHz) spectrum of **2** in $CDCl_3$

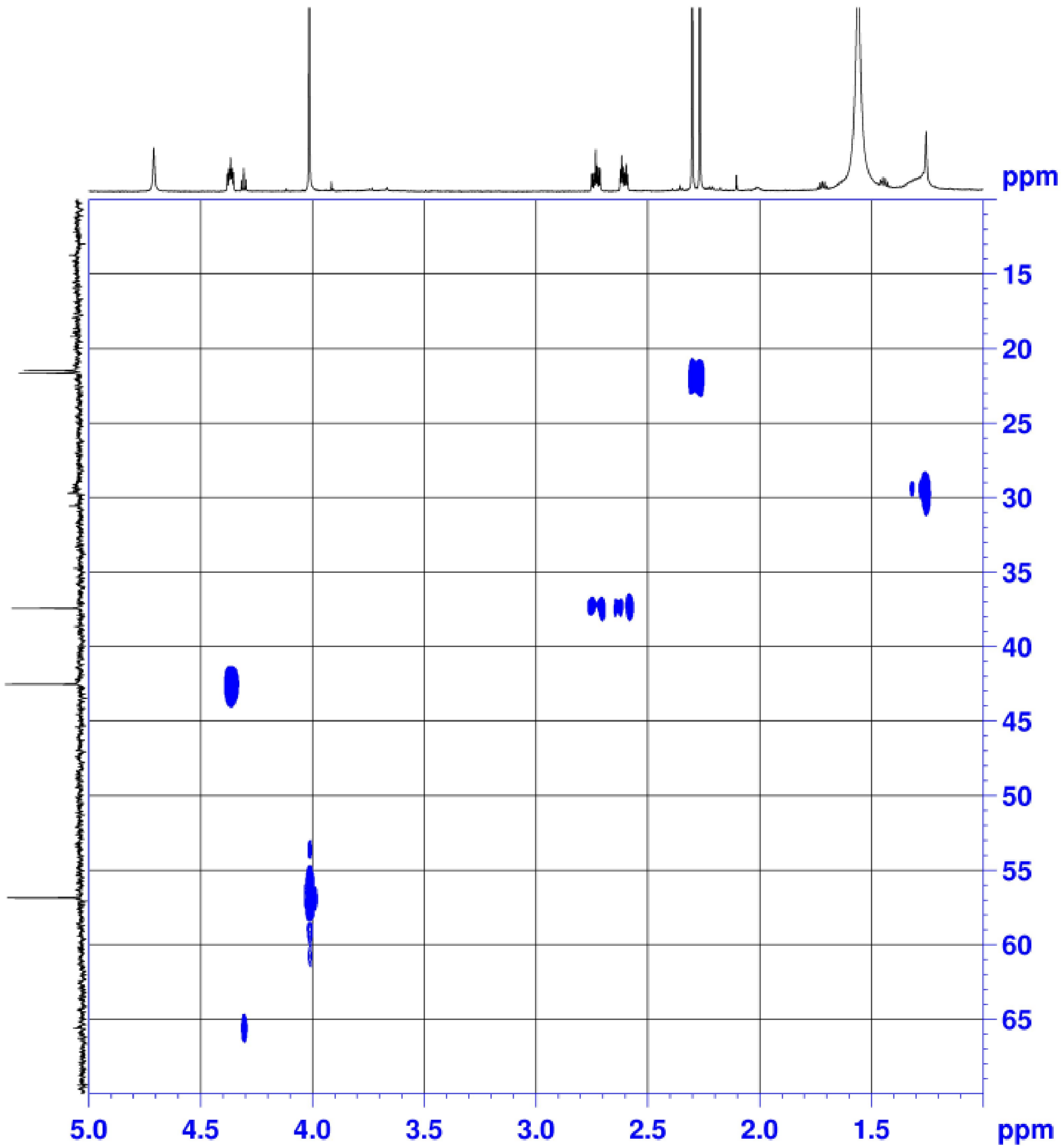

HSQC (700 MHz) spectrum of **2** in $CDCl_3$

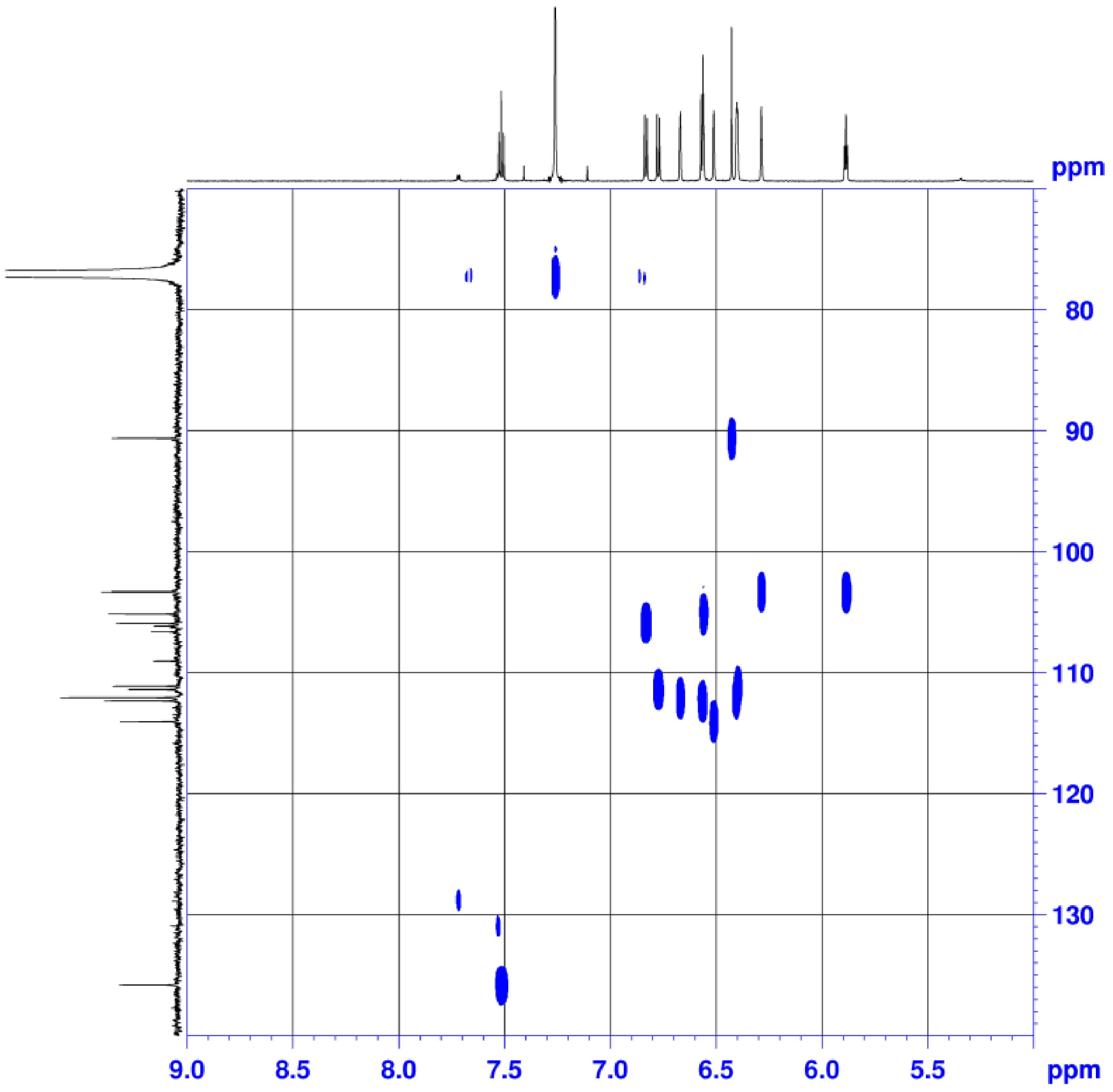

HMBC (700 MHz) spectrum of **2** in $CDCl_3$

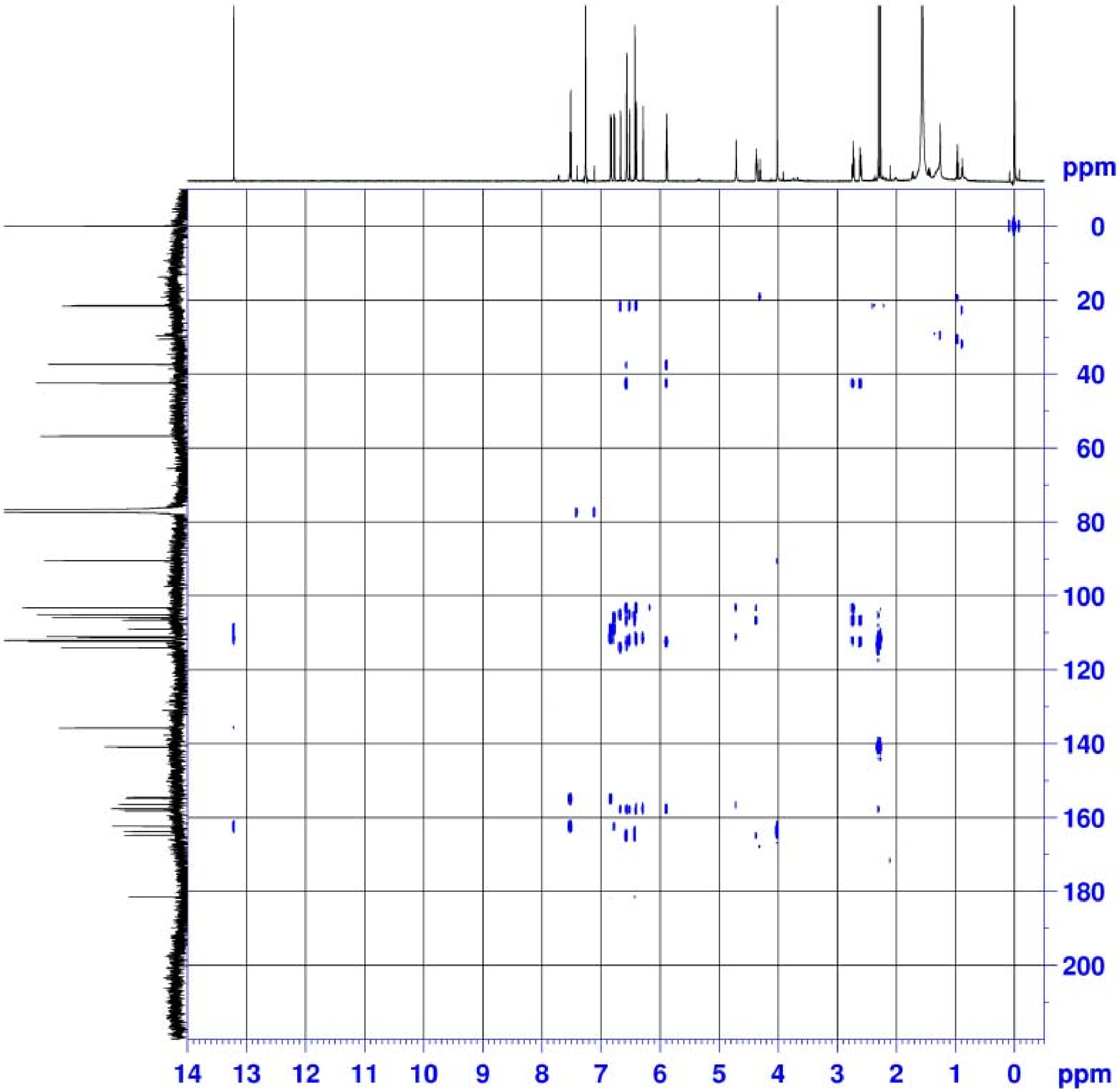

NAME	wubibbib-WBB-42-22-9
EXPNO	15
PROCNO	1
Date_	20210207
Time	22.05 h
INSTRUM	spect
PROBHD	Z120187_0028 (
PULPROG	hmbcetgpl3nd
TD	4096
SOLVENT	CDCl3
NS	36
DS	16
SWH	11432.927 Hz
FIDRES	5.582484 Hz
AQ	0.1791817 sec
RG	181.26
DW	43.733 usec
DE	10.00 usec
TE	298.0 K
CNST6	120.0000000
CNST7	165.0000000
CNST13	8.0000000
D0	0.00000300 sec
D1	1.00000000 sec
D6	0.06250000 sec
D16	0.00020000 sec
IN0	0.00001180 sec
ND0	2
TD	128
SFO1	176.0797 MHz
FIDRES	331.038147 Hz
SW	240.646 ppm
FnMODE	Echo-Antiecho
SI	2048
SF	700.1800175 MHz
WDW	SINE
SSB	4
LB	0.00 Hz
GB	0
PC	1.40
SI	1024
MC2	echo-antiecho
SF	176.0604010 MHz
WDW	QSINE
SSB	2
LB	0.00 Hz
GB	0

HMBC (700 MHz) spectrum of **2** in $CDCl_3$

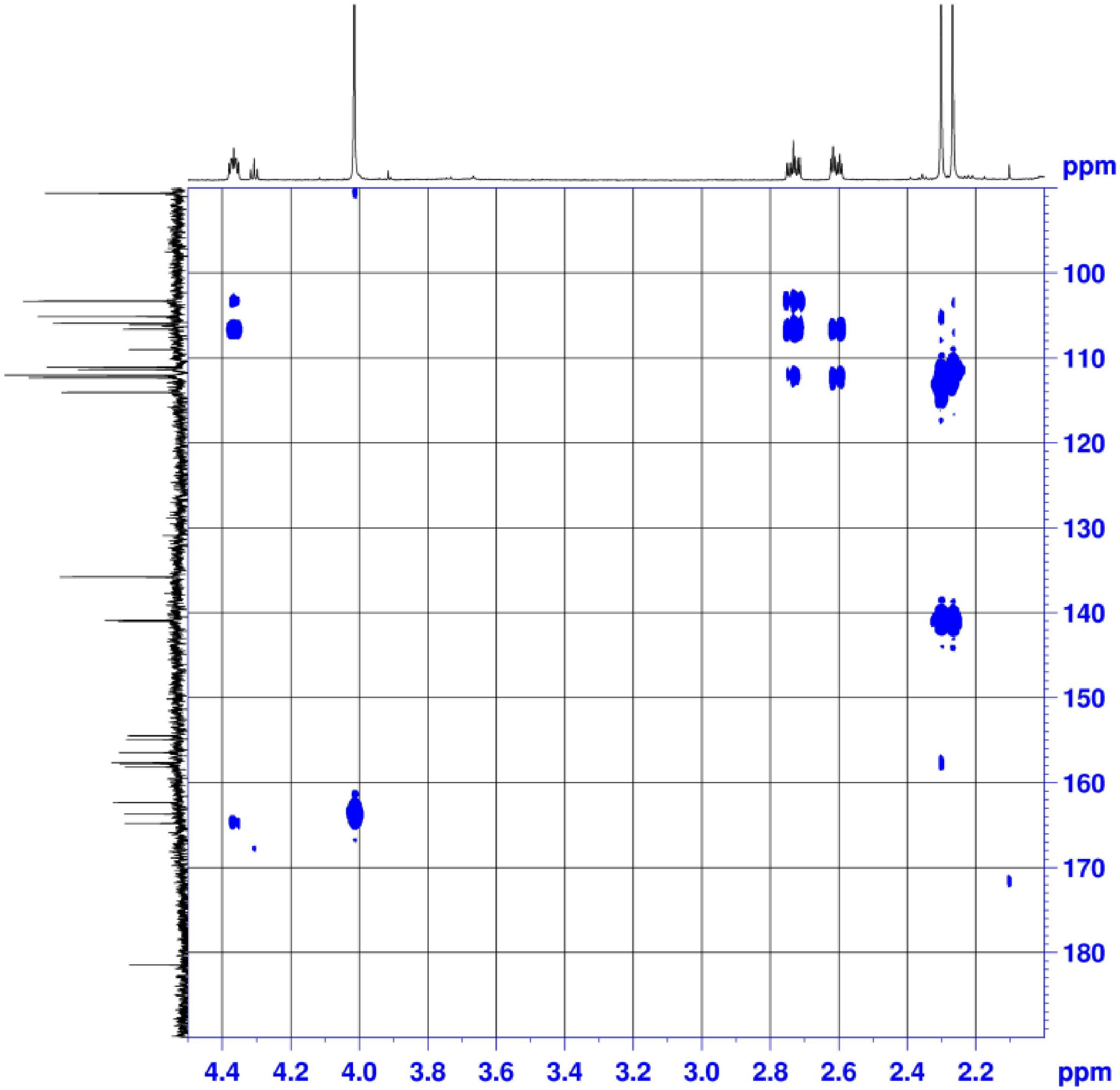

HMBC (700 MHz) spectrum of **2** in $CDCl_3$

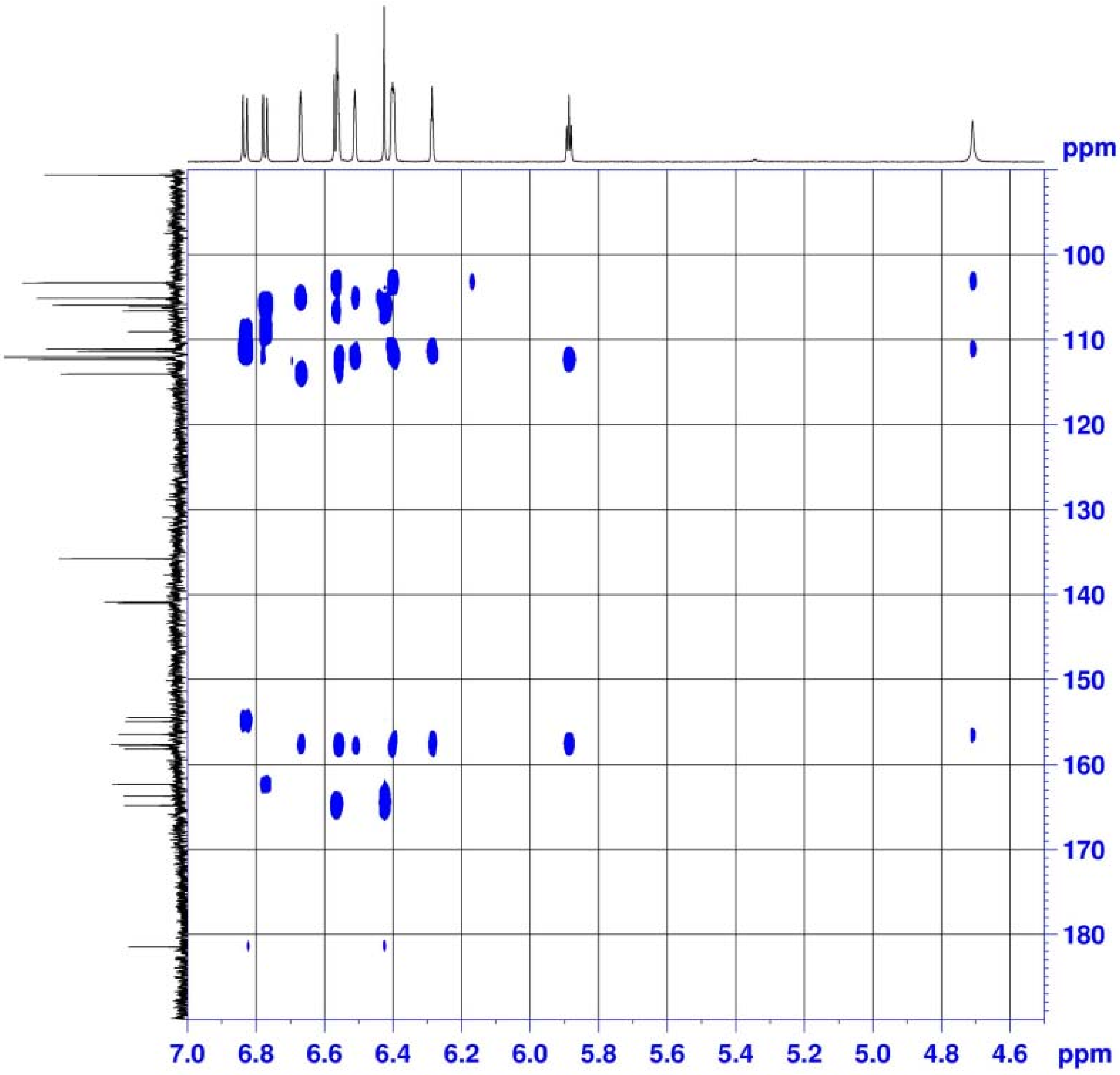

NOESY (700 MHz) spectrum of **2** in $CDCl_3$

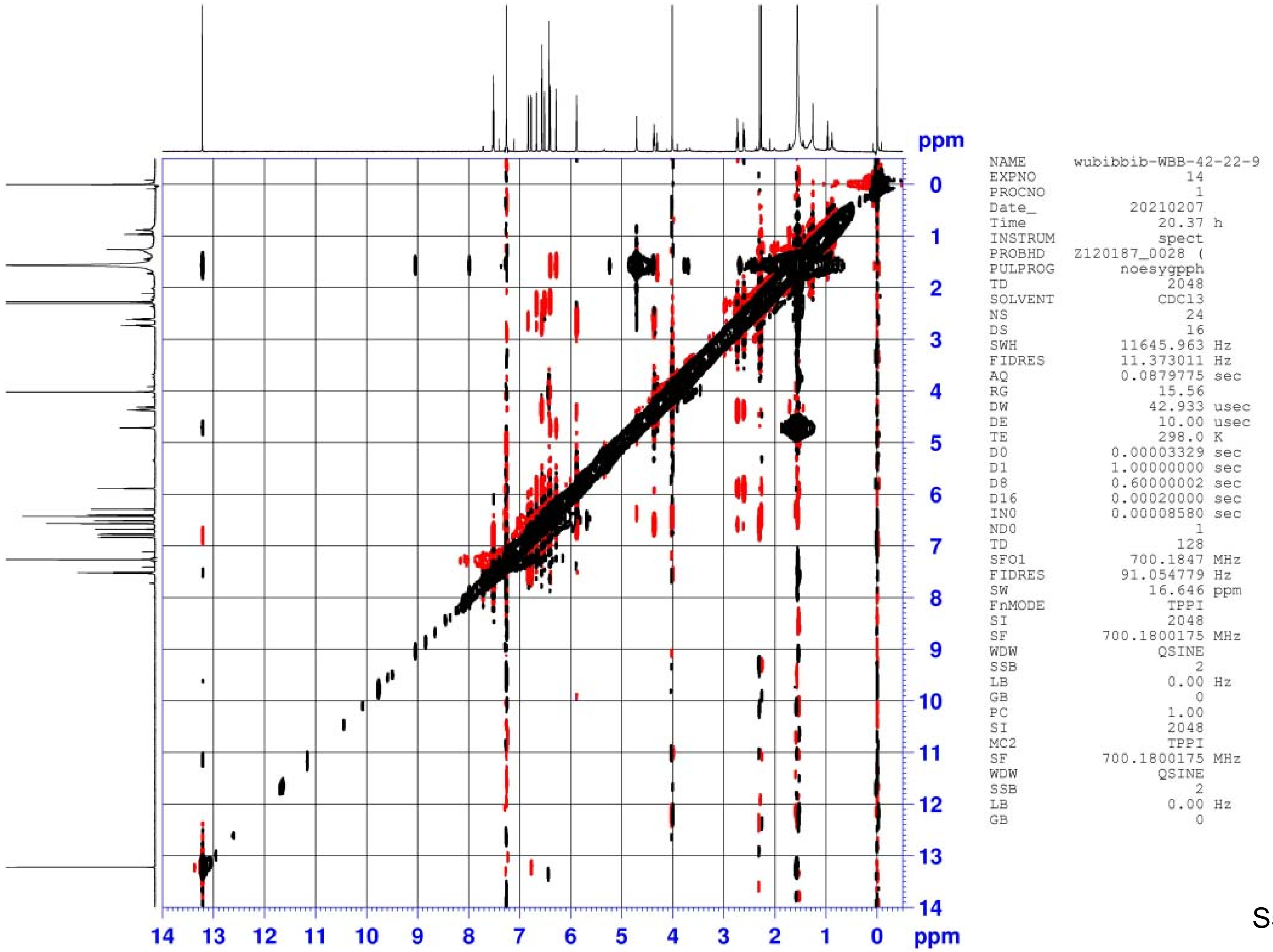

NOESY (700 MHz) spectrum of **2** in $CDCl_3$

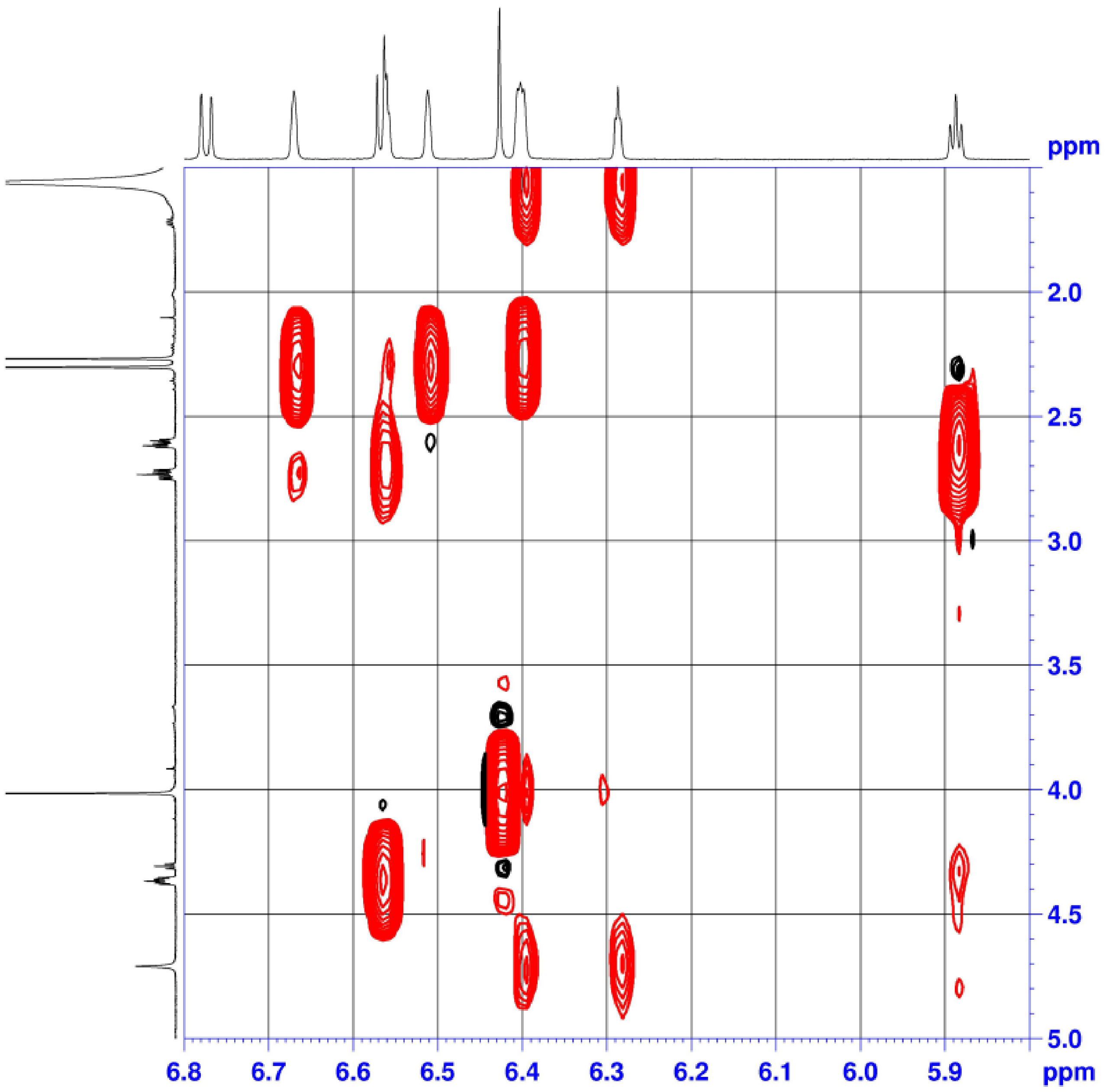

HR-ESIMS for **3**

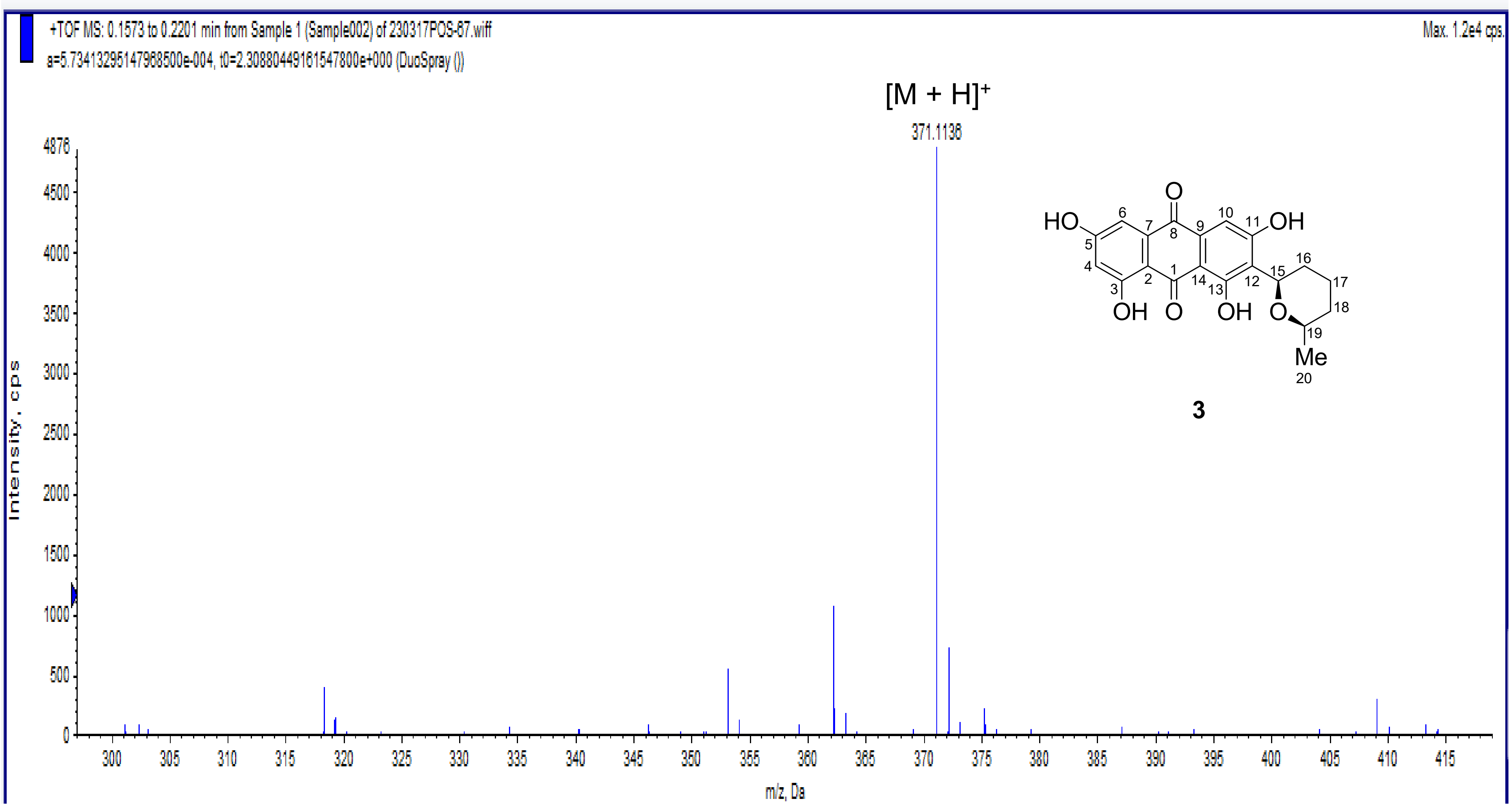

^{1}H (400 MHz) NMR spectrum of **3** in DMSO-d_6

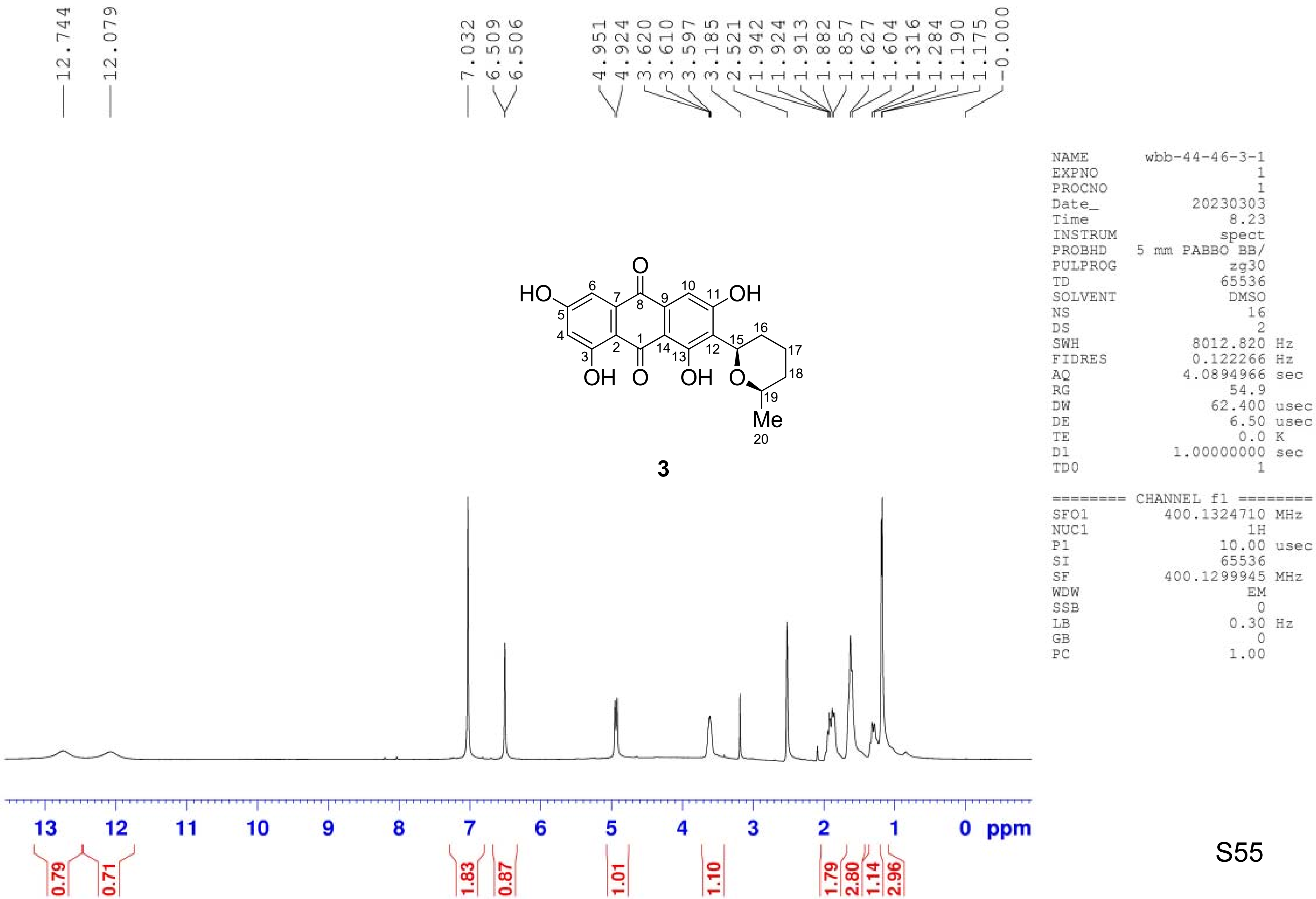

^{1}H (400 MHz) NMR spectrum of **3** in DMSO-d_6

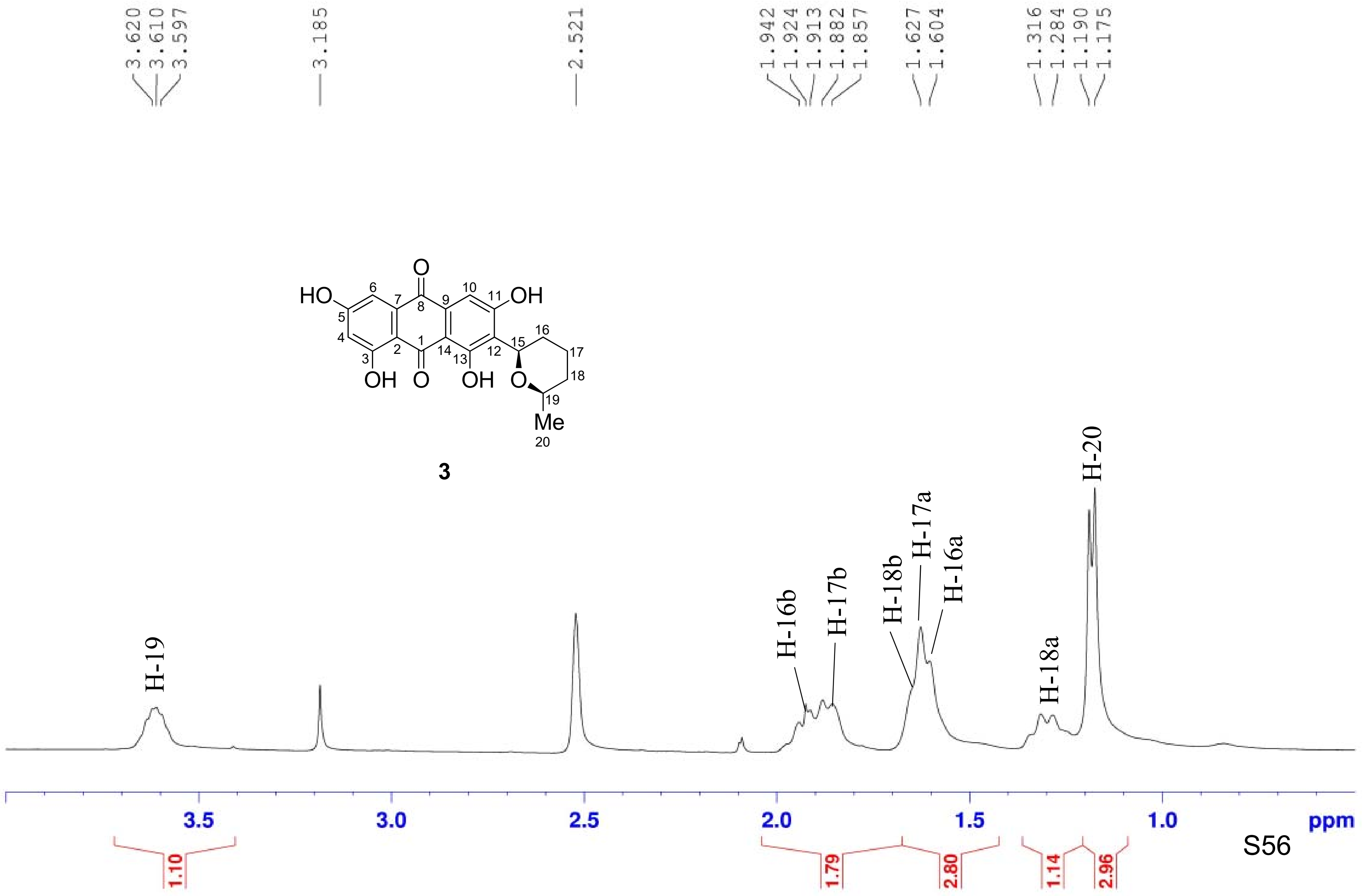

^{1}H (400 MHz) NMR spectrum of **3** in DMSO-d_6

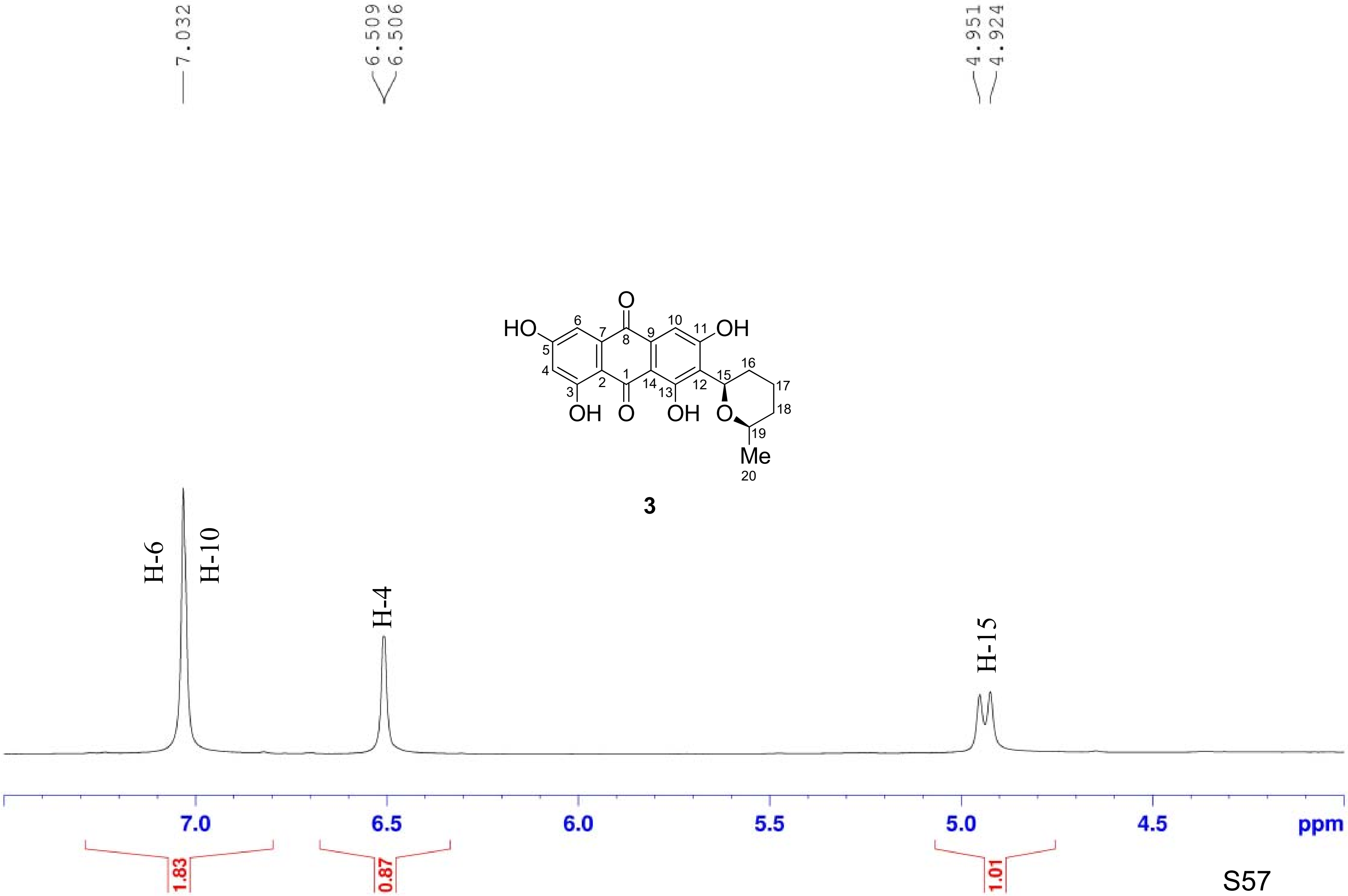

^{13}C (100 MHz) NMR spectrum of **3** in DMSO-d_6

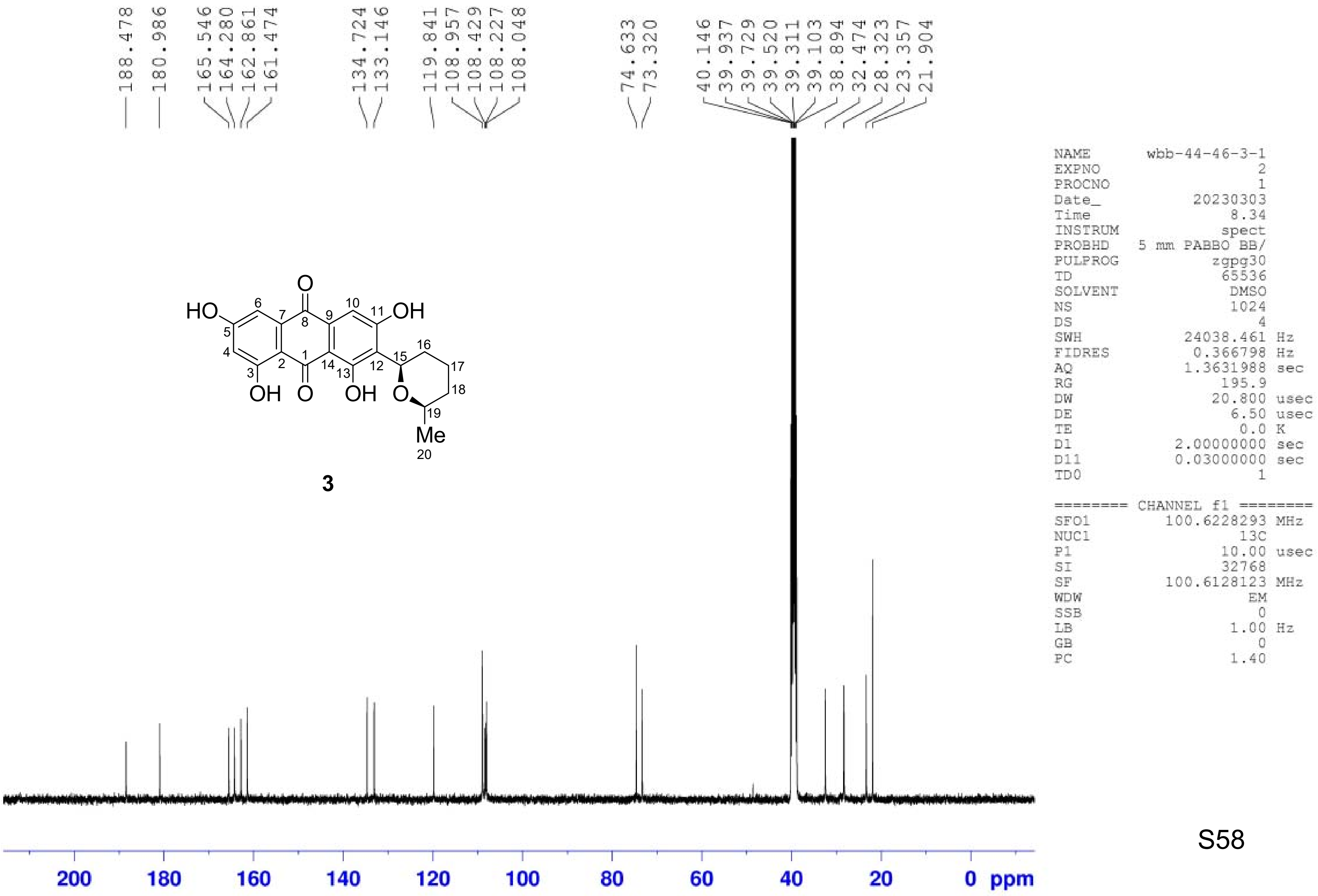

^{13}C (100 MHz) NMR spectrum of **3** in DMSO-d_6

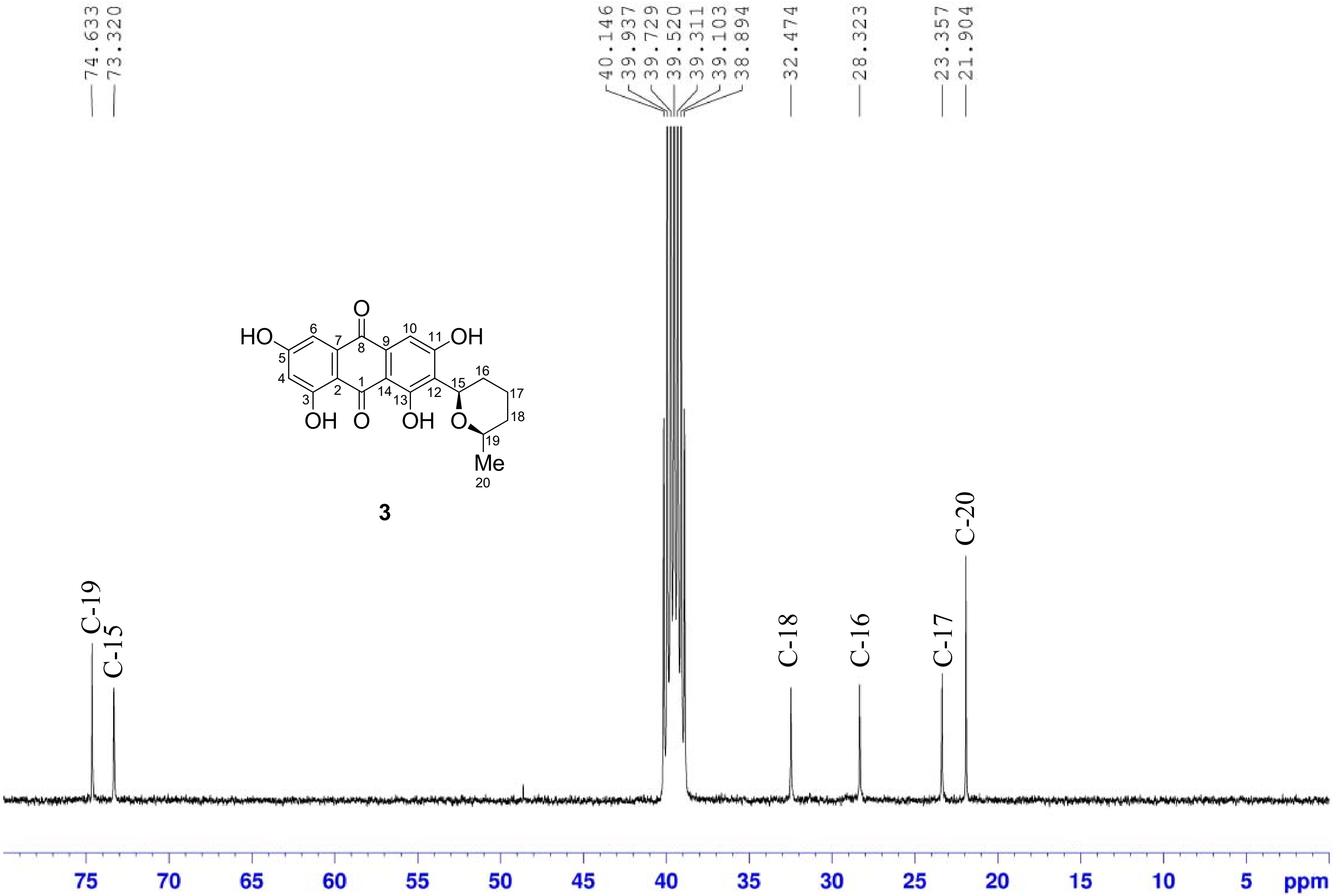

^{13}C (100 MHz) NMR spectrum of **3** in DMSO-d_6

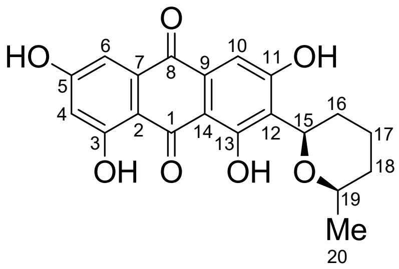

3

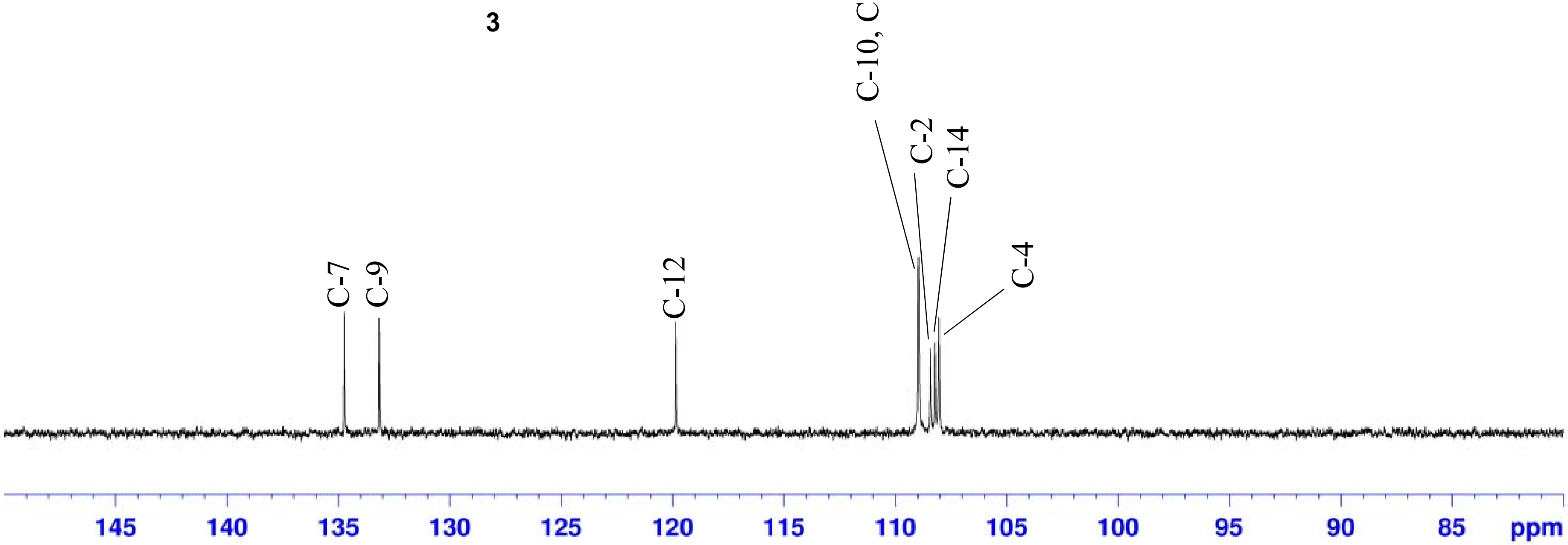

^{13}C (100 MHz) NMR spectrum of **3** in DMSO-d_6

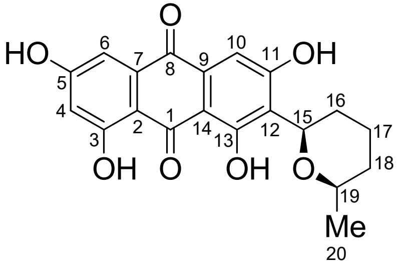

3

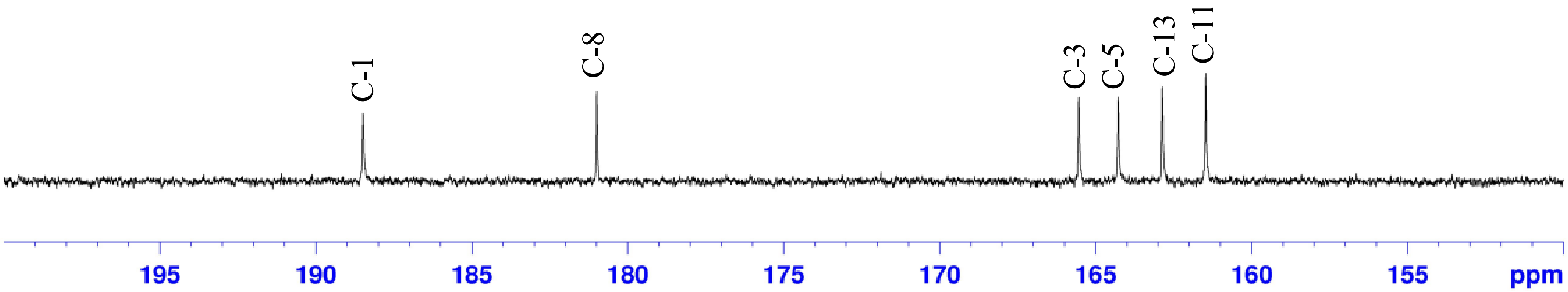

DEPT135 (100 MHz) spectrum of **3** in DMSO-d_6

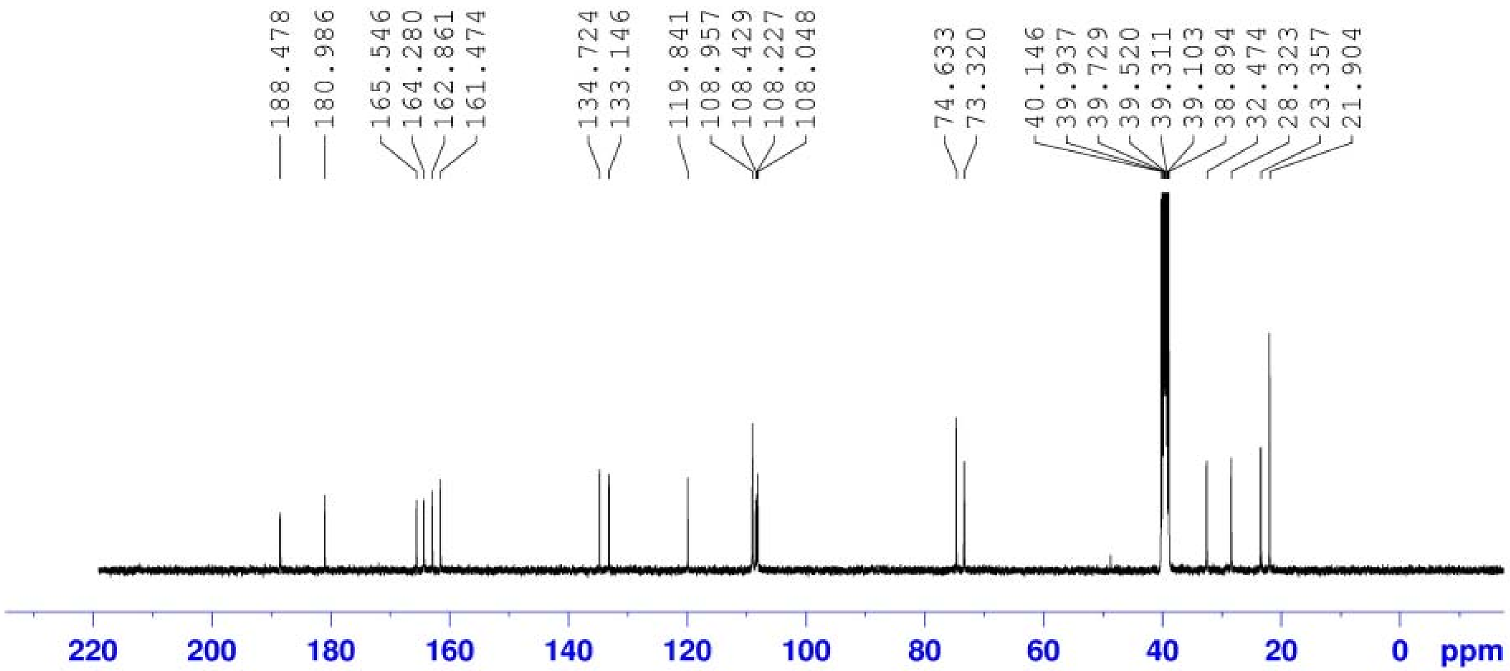

NAME	wbb-47-17-1-r
EXPNO	6
PROCNO	1
Date_	20210518
Time	4.37 h
INSTRUM	spect
PROBHD	Z108618_0231 (
PULPROG	deptsp135
TD	65536
SOLVENT	DMSO
NS	450
DS	8
SWH	16129.032 Hz
FIDRES	0.492219 Hz
AQ	2.0316660 sec
RG	2050
DW	31.000 usec
DE	6.50 usec
TE	298.8 K
CNST2	145.0000000
D1	2.00000000 sec
D2	0.00344828 sec
D12	0.00002000 sec
TD0	1
SFO1	100.6384205 MHz
NUC1	13C
P1	8.25 usec
P13	2000.00 usec
SI	32768
SF	100.6303705 MHz
WDW	EM
SSB	0
LB	1.00 Hz
GB	0
PC	1.40

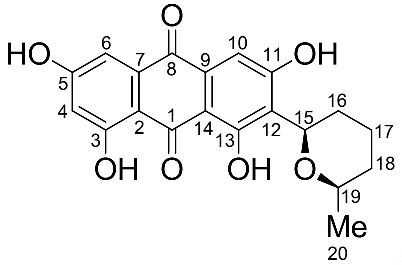

3

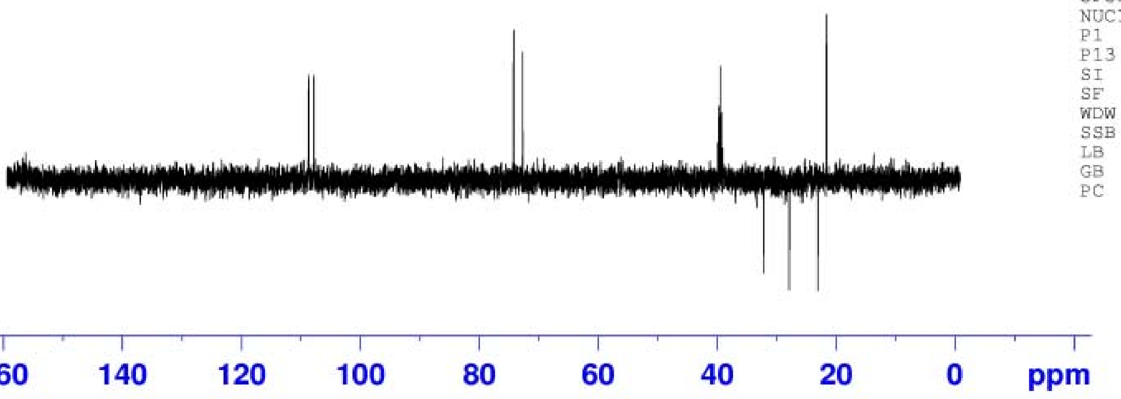

DEPT135 (100 MHz) spectrum of **3** in DMSO-d_6

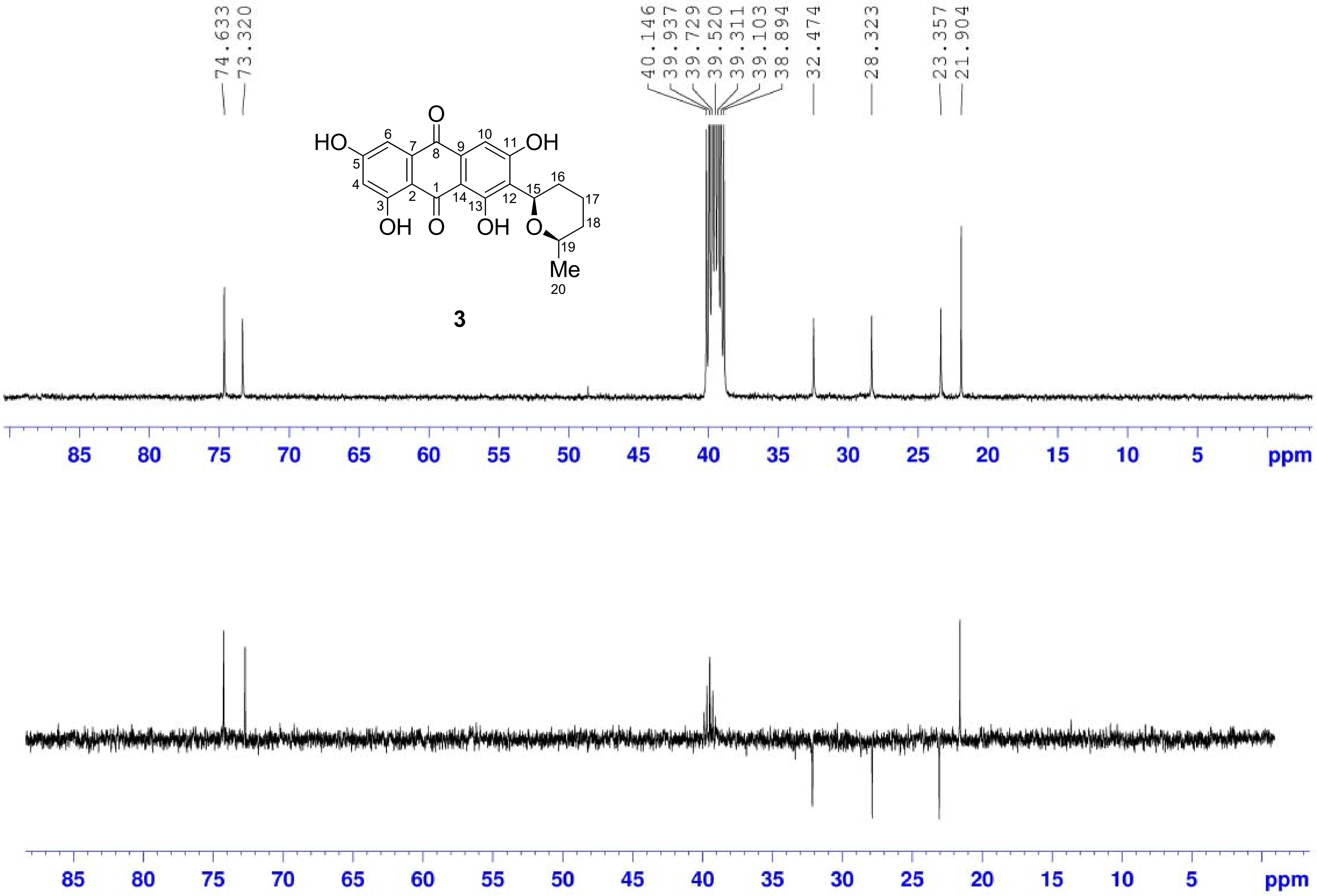

DEPT135 (100 MHz) spectrum of **3** in DMSO-d_6

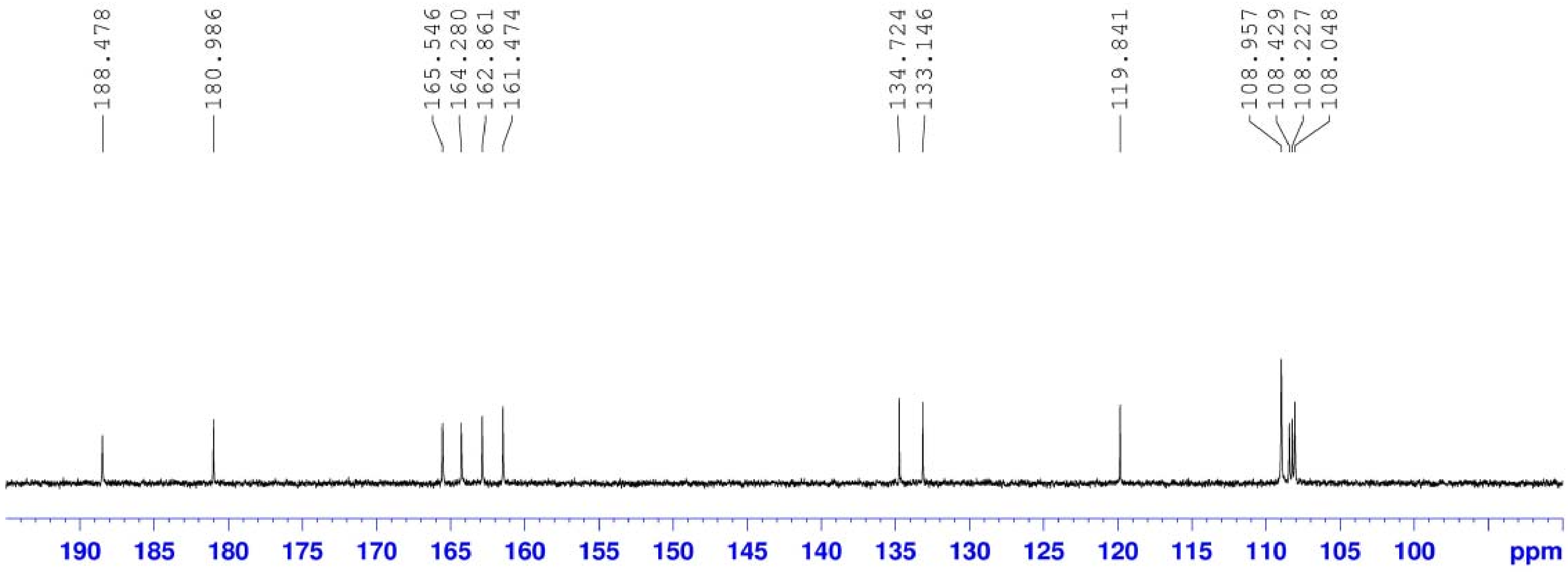

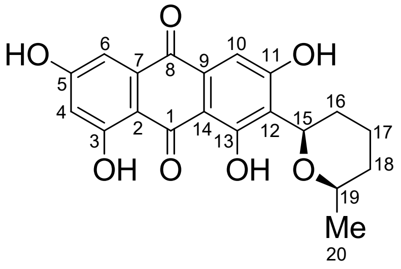

3

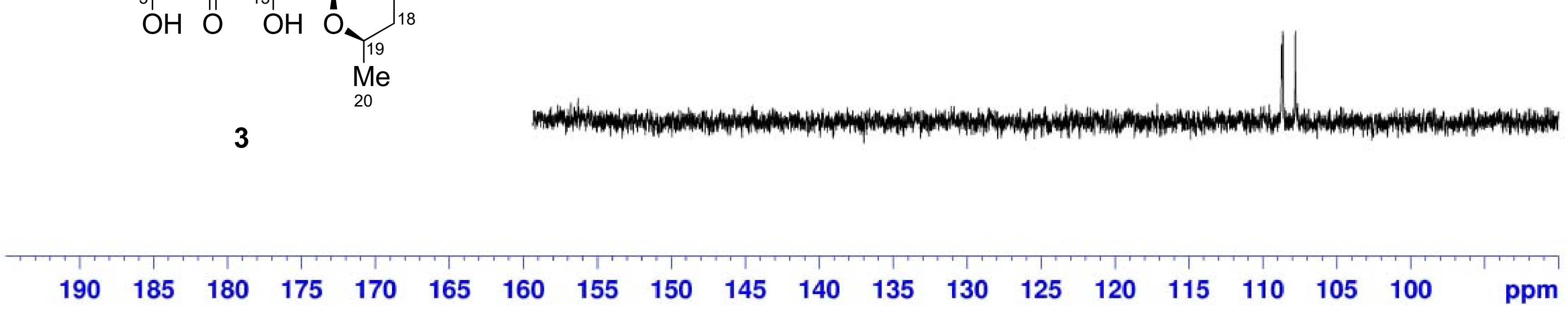

^{1}H–^{1}H COSY (400 MHz) spectrum of **3** in DMSO-d_6

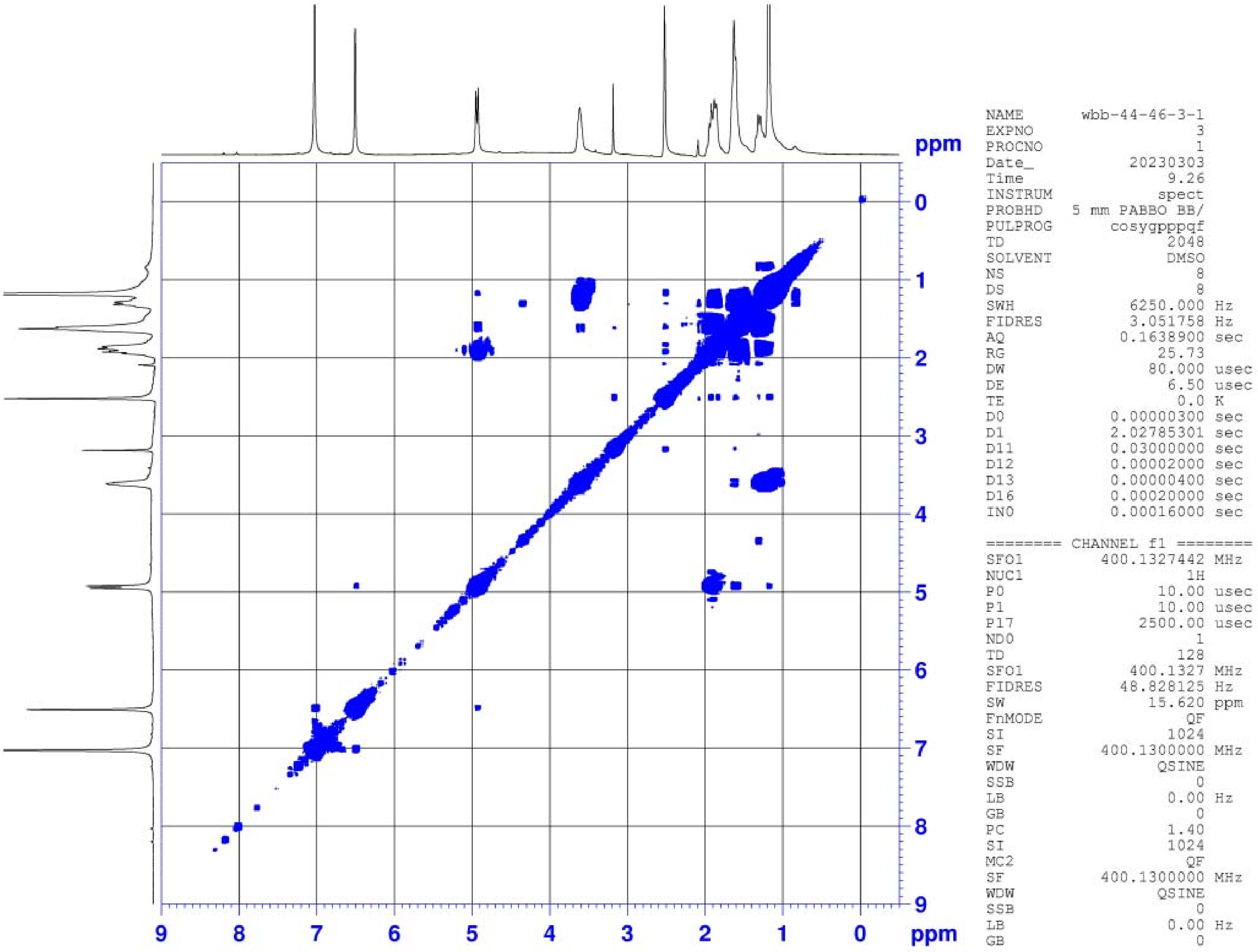

^1H–^1H COSY (400 MHz) spectrum of **3** in DMSO-d_6

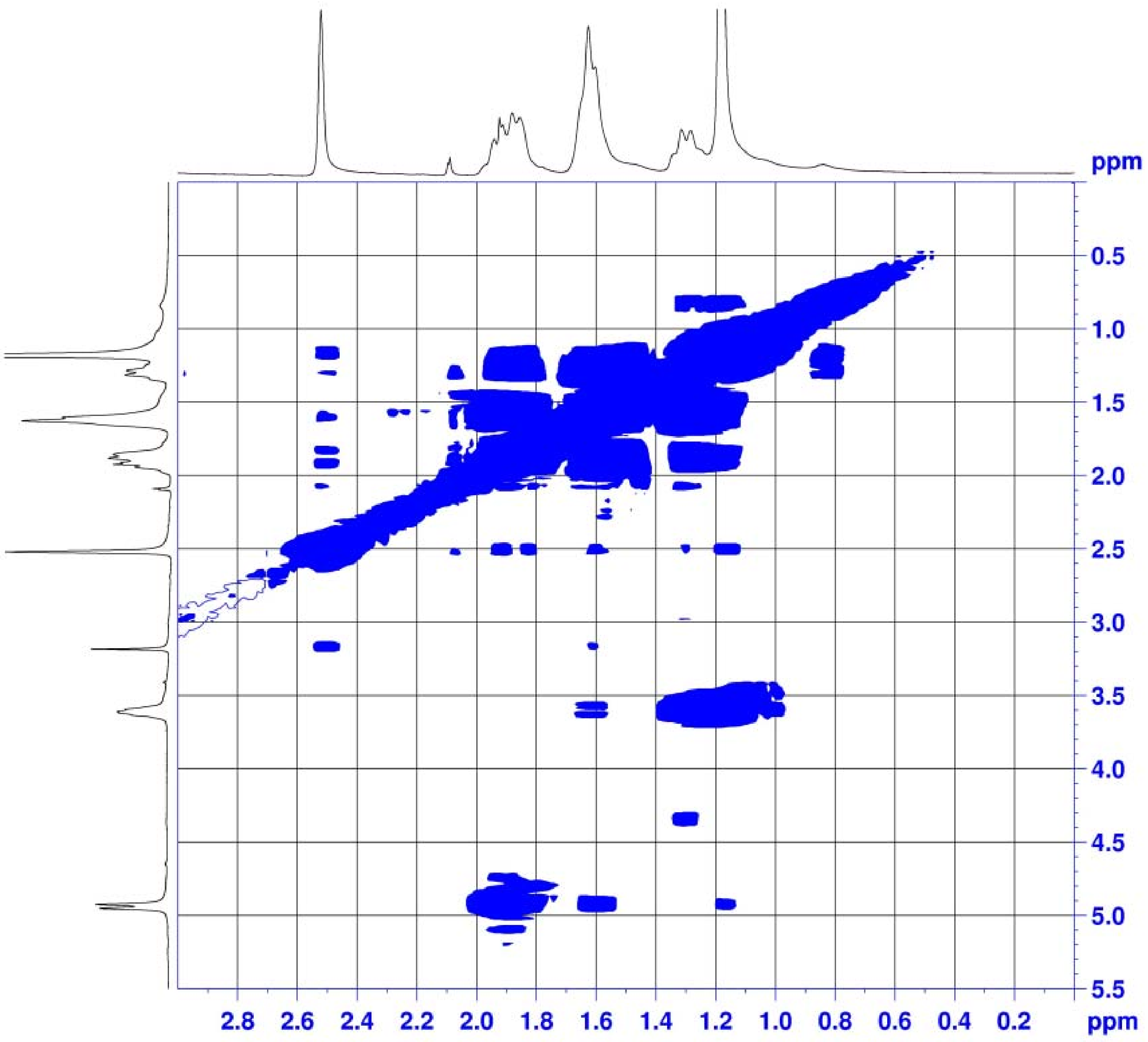

^{1}H–^{1}H COSY (400 MHz) spectrum of **3** in DMSO-d_6

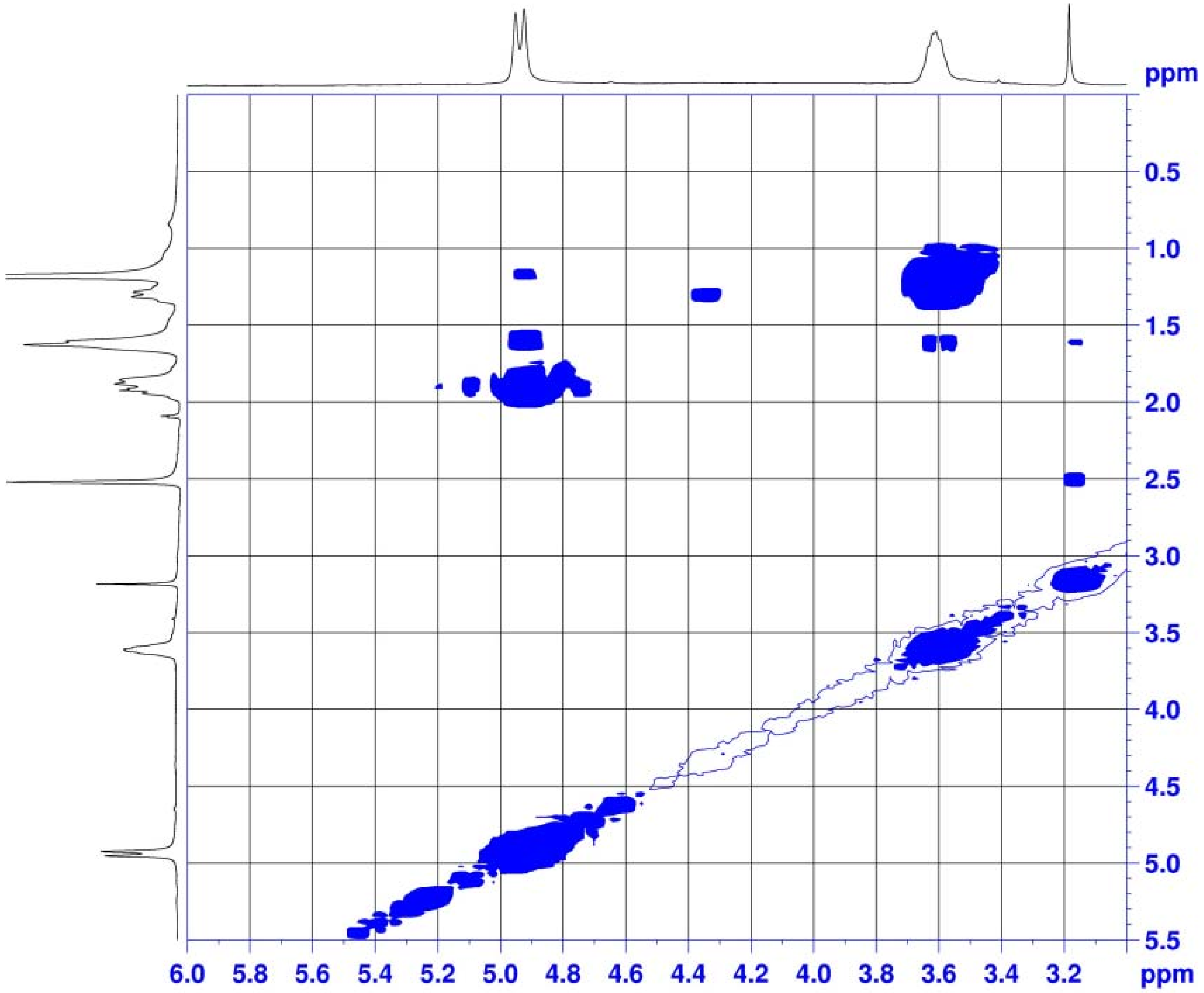

HSQC (400 MHz) spectrum of **3** in DMSO-d_6

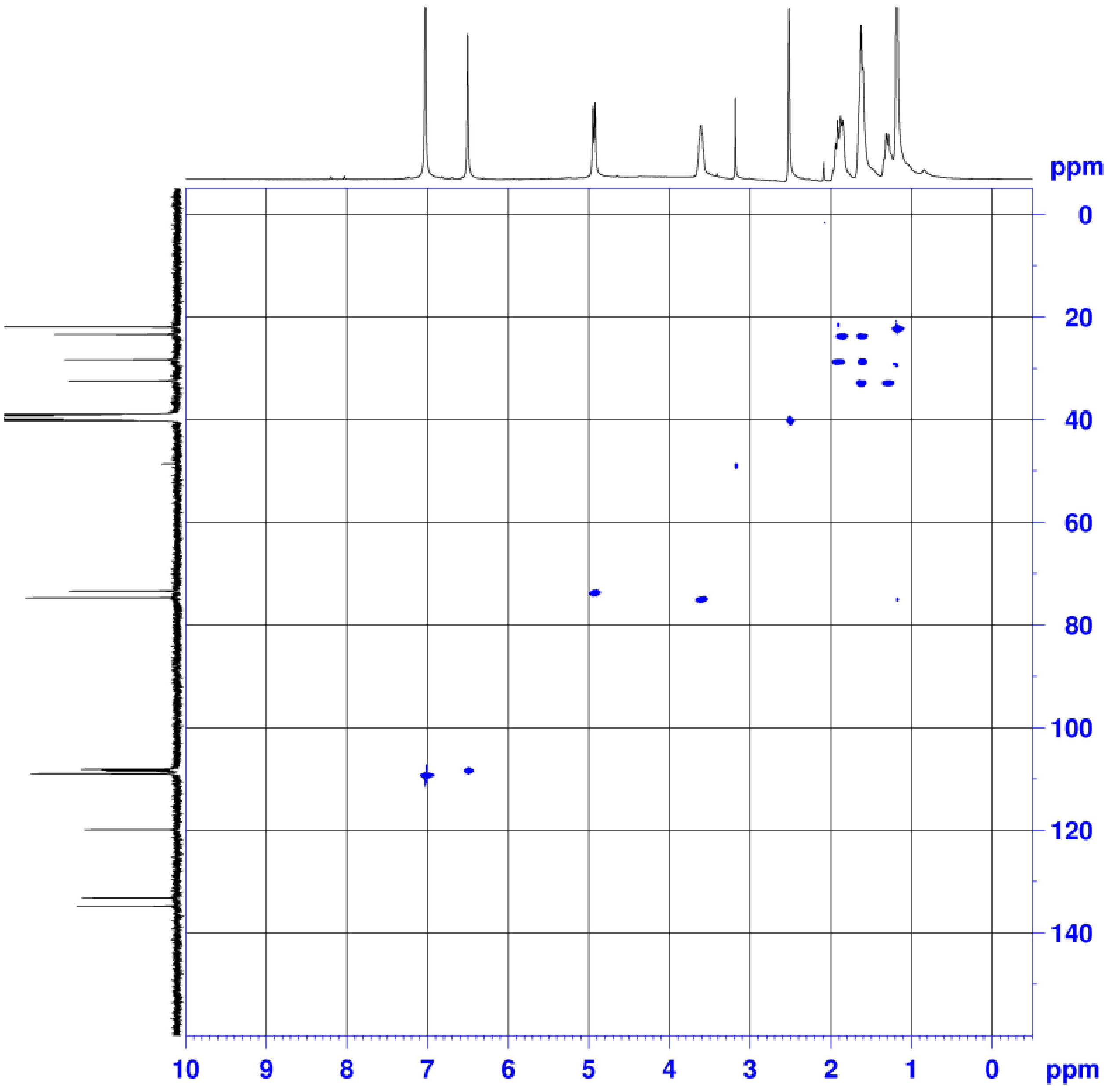

NAME	wbb-44-46-3-1	
EXPNO	5	
PROCNO	1	
Date_	20230303	
Time	10.40	
INSTRUM	spect	
PROBHD	5 mm PABBO BB/	
PULPROG	hsqcedetgpsisp2.3	
TD	2048	
SOLVENT	DMSO	
NS	4	
DS	32	
SWH	4795.396	Hz
FIDRES	2.341502	Hz
AQ	0.2135881	sec
RG	195.9	
DW	104.267	usec
DE	6.50	usec
TE	0.0	K
CNST2	145.0000000	
CNST17	-0.5000000	
D0	0.00000300	sec
D1	2.00000000	sec
D4	0.00172414	sec
D11	0.03000000	sec
D16	0.00020000	sec
D21	0.00360000	sec
D24	0.00089000	sec
IN0	0.00003010	sec
======== CHANNEL f1 ========		
SFO1	400.1318806	MHz
NUC1	1H	
P1	10.00	usec
P2	20.00	usec
P28	1000.00	usec
ND0	2	
TD	256	
SFO1	100.6198	MHz
FIDRES	64.887871	Hz
SW	165.090	ppm
FnMODE	Echo-Antiecho	
SI	1024	
SF	400.1300000	MHz
WDW	QSINE	
SSB	2	
LB	0.00	Hz
GB	0	
PC	1.40	
SI	1024	
MC2	echo-antiecho	
SF	100.6127685	MHz
WDW	QSINE	
SSB	2	
LB	0.00	Hz
GB	0	

HSQC (400 MHz) spectrum of **3** in DMSO-d_6

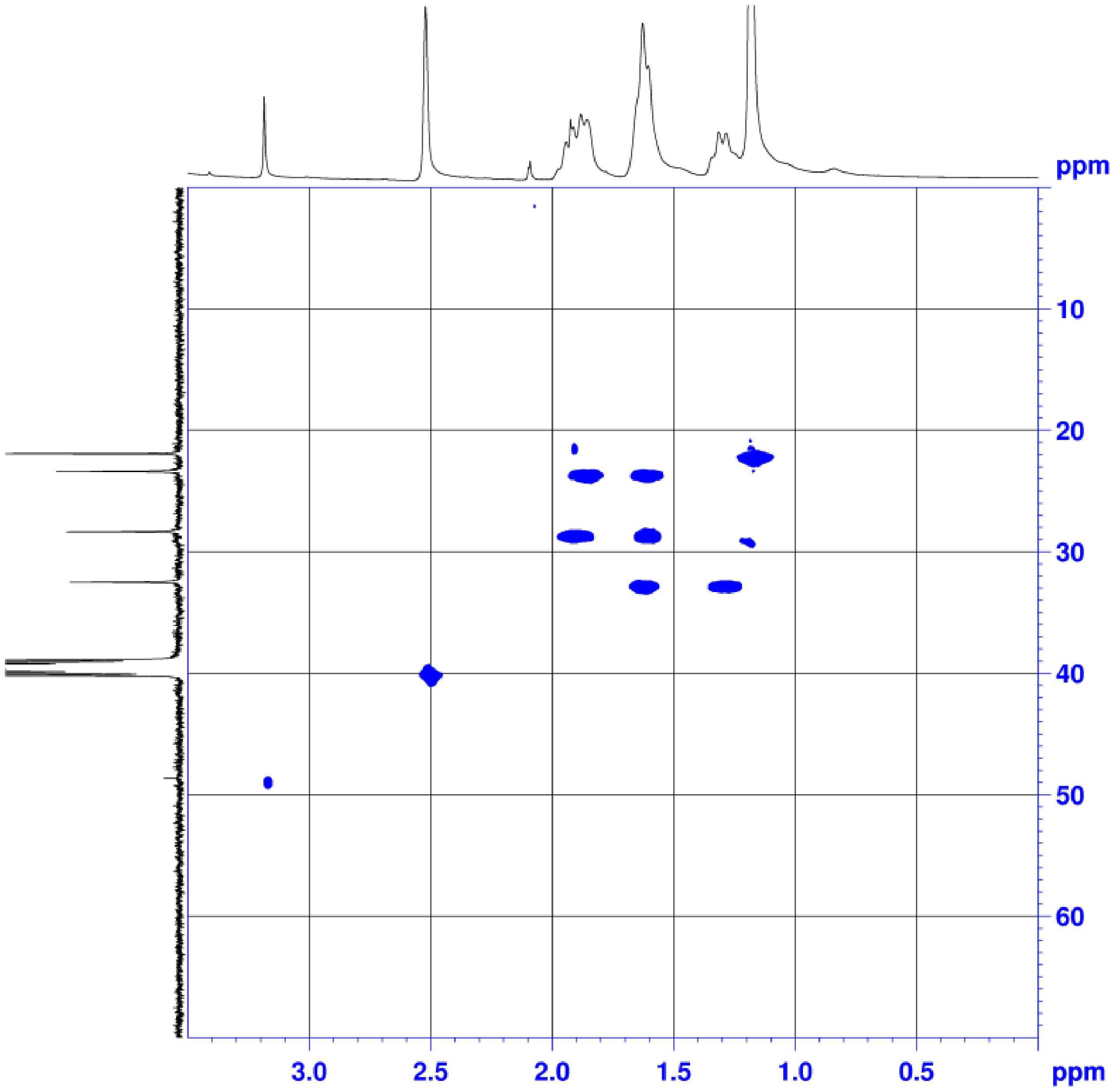

HSQC (400 MHz) spectrum of **3** in DMSO-d_6

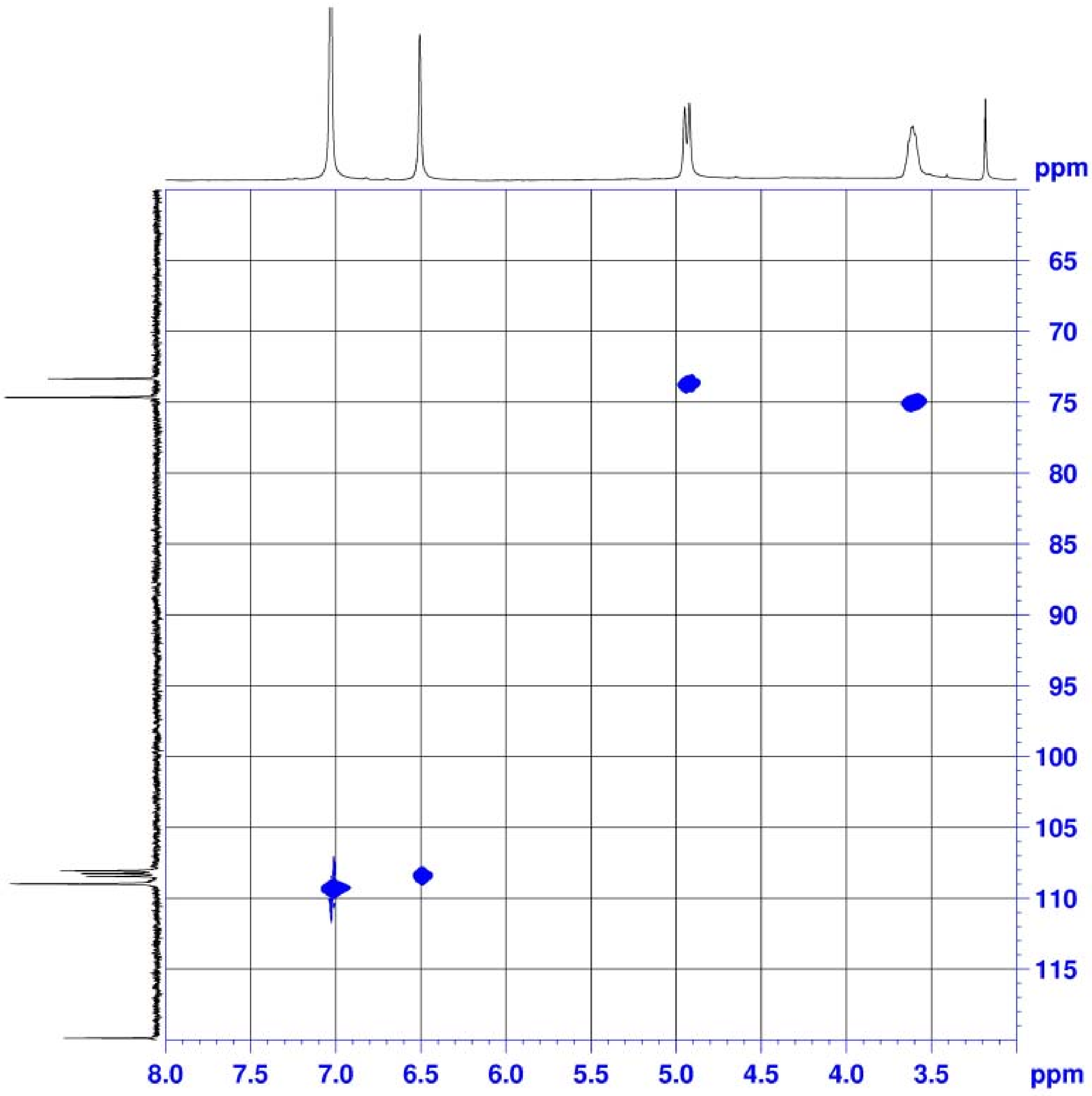

HMBC (400 MHz) spectrum of **3** in DMSO-d_6

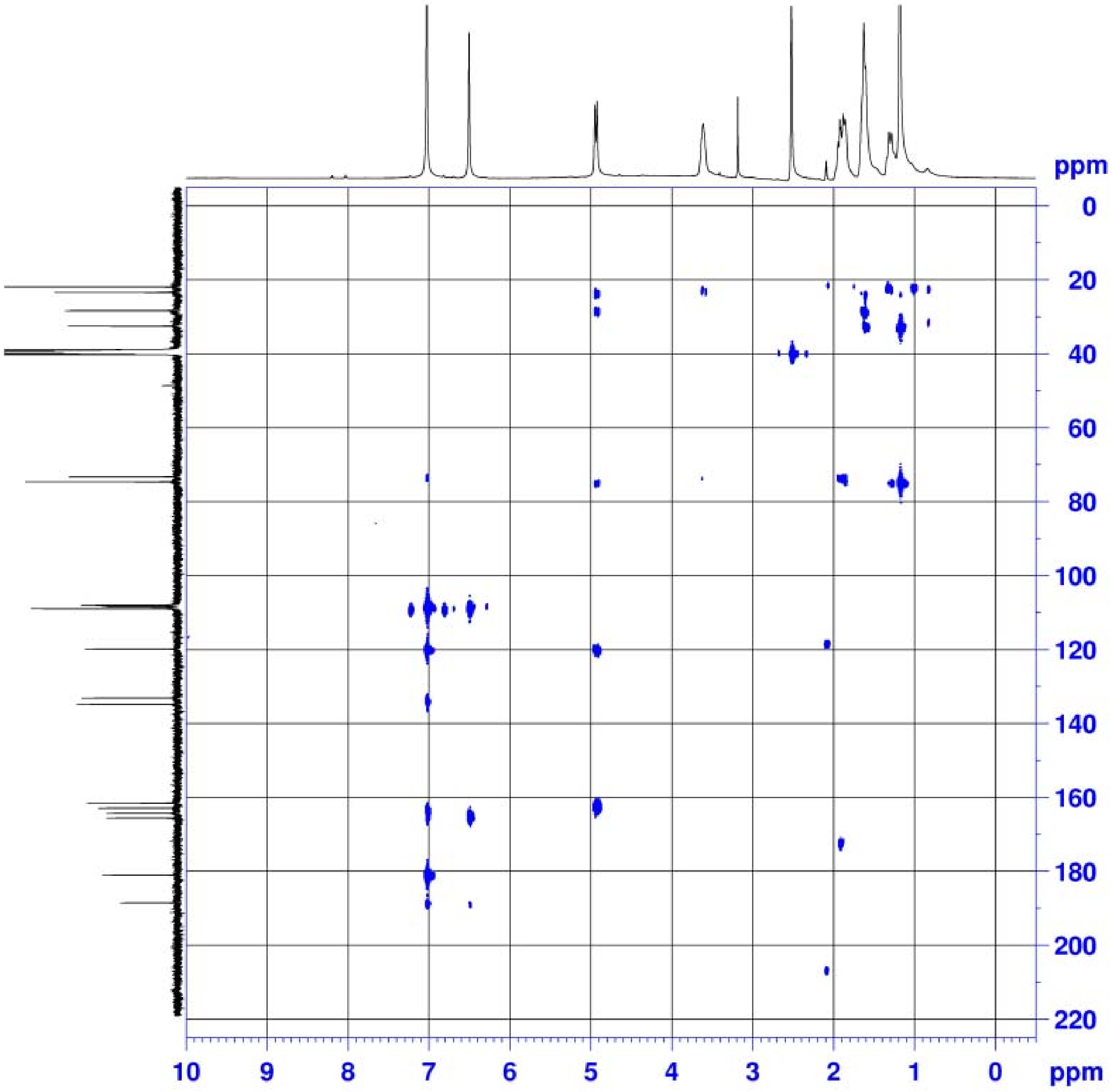

NAME	wbb-44-46-3-1
EXPNO	4
PROCNO	1
Date_	20230303
Time	10.07
INSTRUM	spect
PROBHD	5 mm PABBO BB/
PULPROG	hmbcgplpndqf
TD	2048
SOLVENT	DMSO
NS	8
DS	16
SWH	6250.000 Hz
FIDRES	3.051758 Hz
AQ	0.1638900 sec
RG	195.9
DW	80.000 usec
DE	6.50 usec
TE	0.0 K
CNST2	145.0000000
CNST13	10.0000000
D0	0.00000300 sec
D1	1.53317797 sec
D2	0.00344828 sec
D6	0.05000000 sec
D16	0.00020000 sec
IN0	0.00002240 sec
======== CHANNEL f1 ========	
SFO1	400.1327442 MHz
NUC1	1H
P1	10.00 usec
P2	20.00 usec
ND0	2
TD	128
SFO1	100.6228 MHz
FIDRES	174.386154 Hz
SW	221.833 ppm
FnMODE	QF
SI	2048
SF	400.1300000 MHz
WDW	SINE
SSB	0
LB	0.00 Hz
GB	0
PC	1.40
SI	1024
MC2	QF
SF	100.6127685 MHz
WDW	SINE
SSB	0
LB	0.00 Hz
GB	0

HMBC (400 MHz) spectrum of **3** in DMSO-d_6

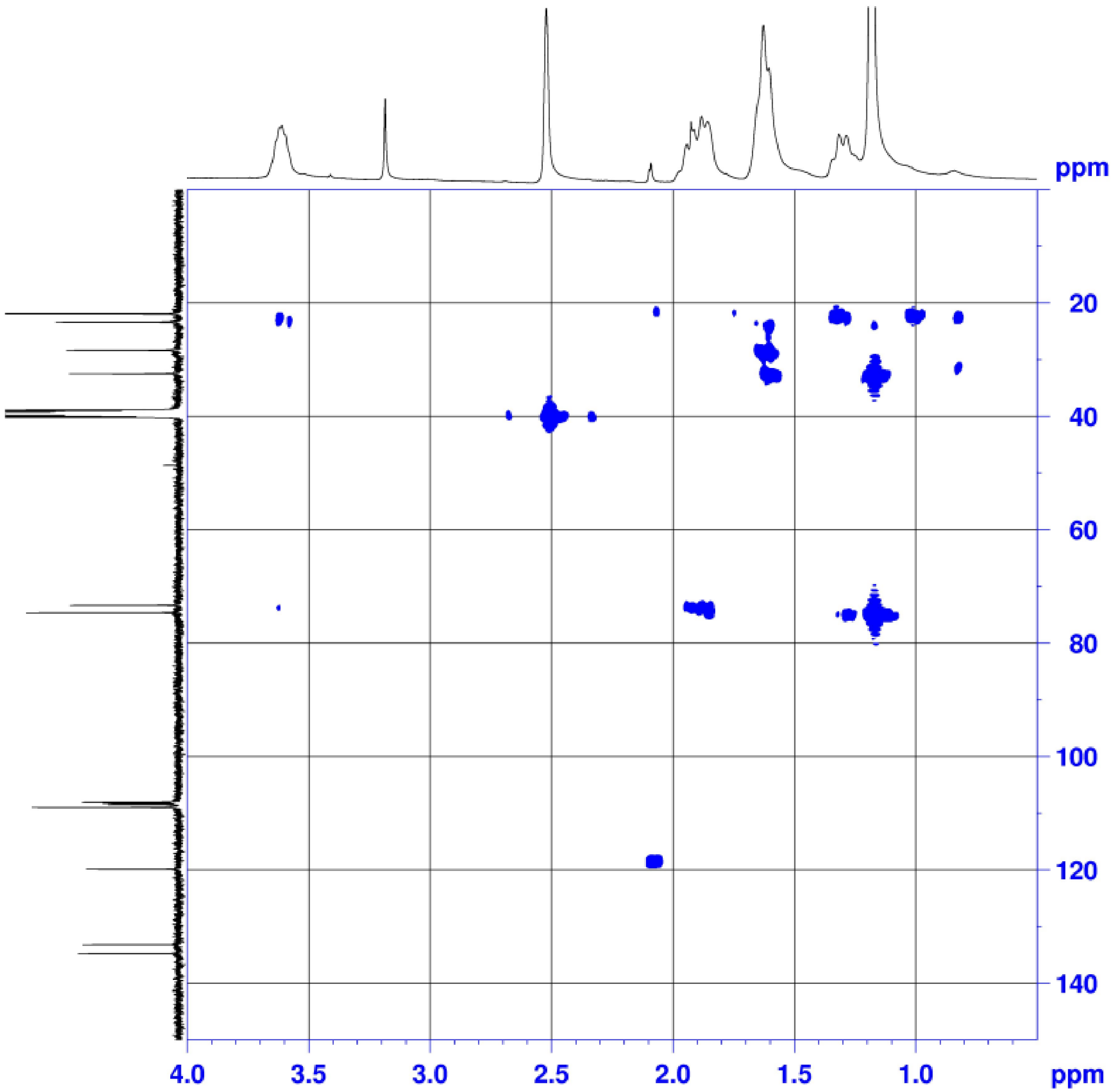

HMBC (400 MHz) spectrum of **3** in DMSO-d_6

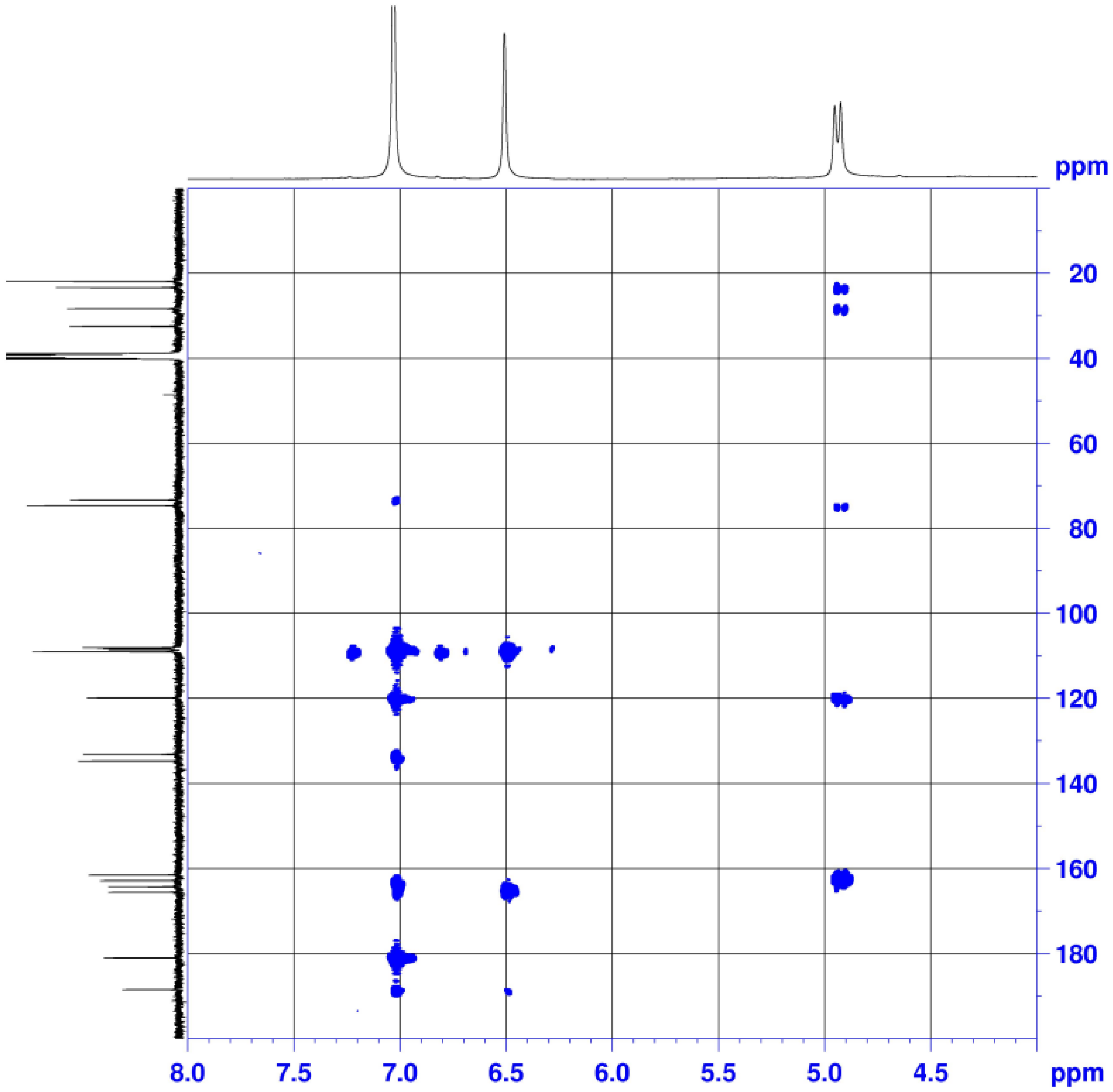

NOESY (400 MHz) spectrum of **3** in DMSO-d_6

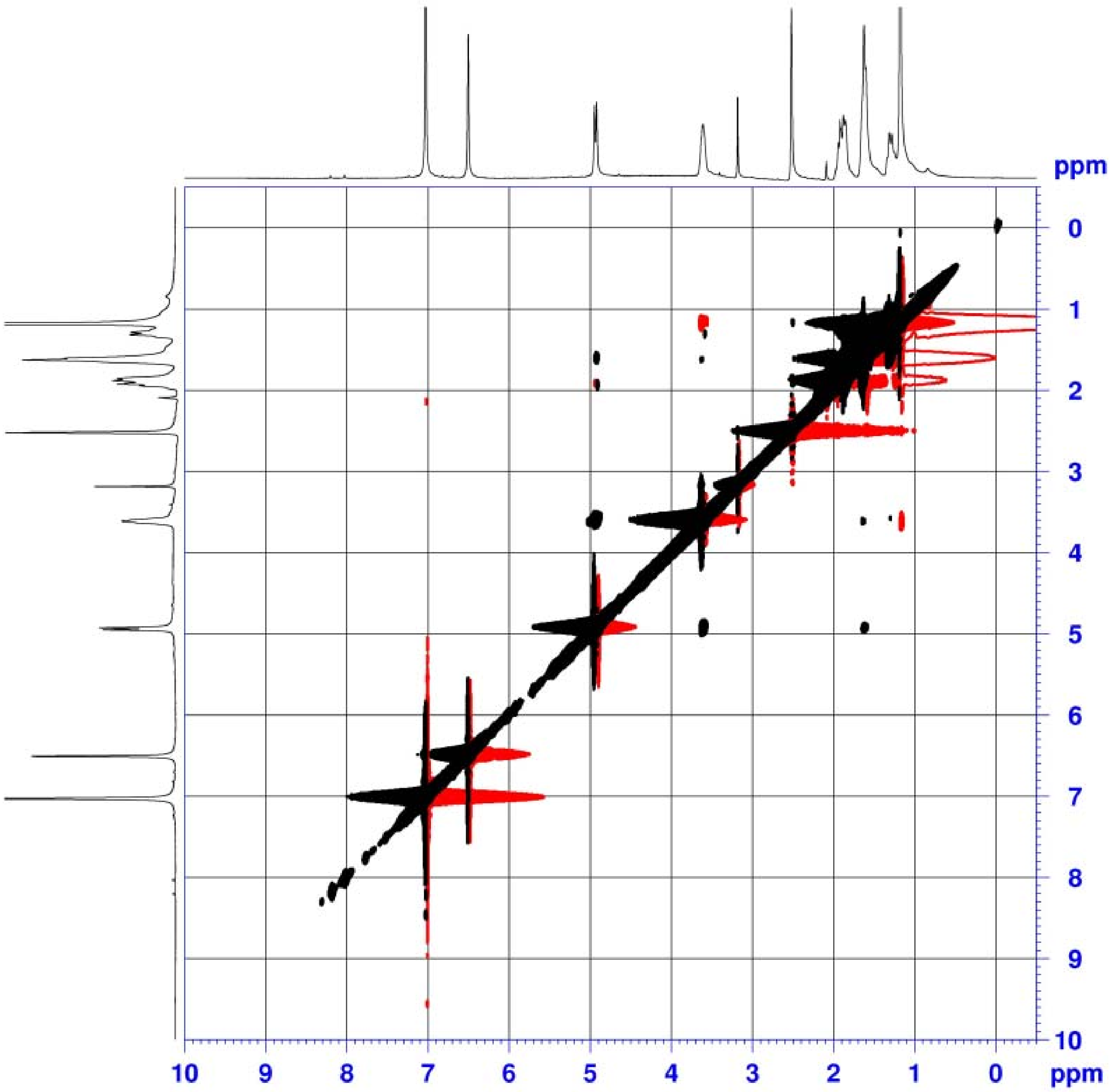


```
NAME          wbb-44-46-3-1
EXPNO                     6
PROCNO                    1
Date_              20230303
Time                  11.21
INSTRUM               spect
PROBHD     5 mm PABBO BB/
PULPROG        noesygpphpp
TD                     2048
SOLVENT                DMSO
NS                        4
DS                       32
SWH               6172.839 Hz
FIDRES            3.014082 Hz
AQ               0.1659380 sec
RG                    25.73
DW                   81.000 usec
DE                     6.50 usec
TE                      0.0 K
D0              0.00006827 sec
D1              2.09011102 sec
D8              0.30000001 sec
D11             0.03000000 sec
D12             0.00002000 sec
D16             0.00020000 sec
IN0             0.00016200 sec

======== CHANNEL f1 ========
SFO1           400.1327617 MHz
NUC1                     1H
P1                    10.00 usec
P2                    20.00 usec
P17                 2500.00 usec
ND0                       1
TD                      256
SFO1               400.1328 MHz
FIDRES            24.112654 Hz
SW                   15.427 ppm
FnMODE          States-TPPI
SI                     1024
SF              400.1300000 MHz
WDW                   QSINE
SSB                       2
LB                     0.00 Hz
GB                        0
PC                     1.00
SI                     1024
MC2             States-TPPI
SF              400.1300000 MHz
WDW                   QSINE
SSB                       2
LB                     0.00 Hz
GB                        0
```

NOESY (400 MHz) spectrum of **3** in DMSO-d_6

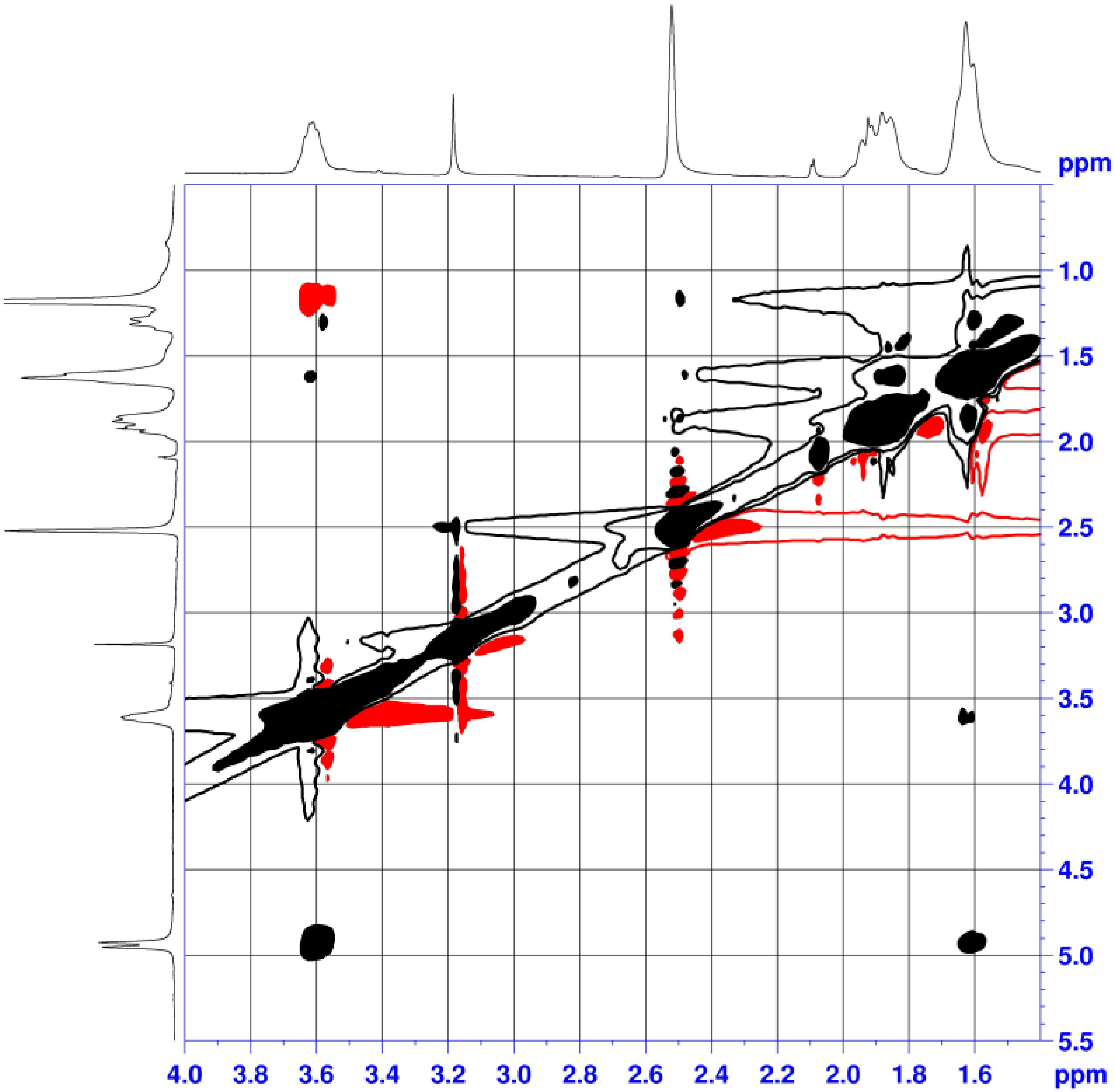

HR-ESIMS for **4**

Mass Spectrum SmartFormula Report

Analysis Info

Analysis Name	D:\Data\MS\data\202110\wubinbin_WBB-145-D-R-6_pos_51_01_11733.d	Acquisition Date	10/27/2021 4:38:12 PM	
Method	LC_Direct Infusion_pos_70-500mz.m	Operator	SCSIO	
Sample Name	wubinbin_WBB-145-D-R-6_pos	Instrument	maXis	255552.00029
Comment				

Acquisition Parameter

Source Type	ESI	Ion Polarity	Positive	Set Nebulizer	0.4 Bar
Focus	Active	Set Capillary	4500 V	Set Dry Heater	180 °C
Scan Begin	70 m/z	Set End Plate Offset	-500 V	Set Dry Gas	4.0 l/min
Scan End	1500 m/z	Set Charging Voltage	0 V	Set Divert Valve	Waste
		Set Corona	0 nA	Set APCI Heater	0 °C

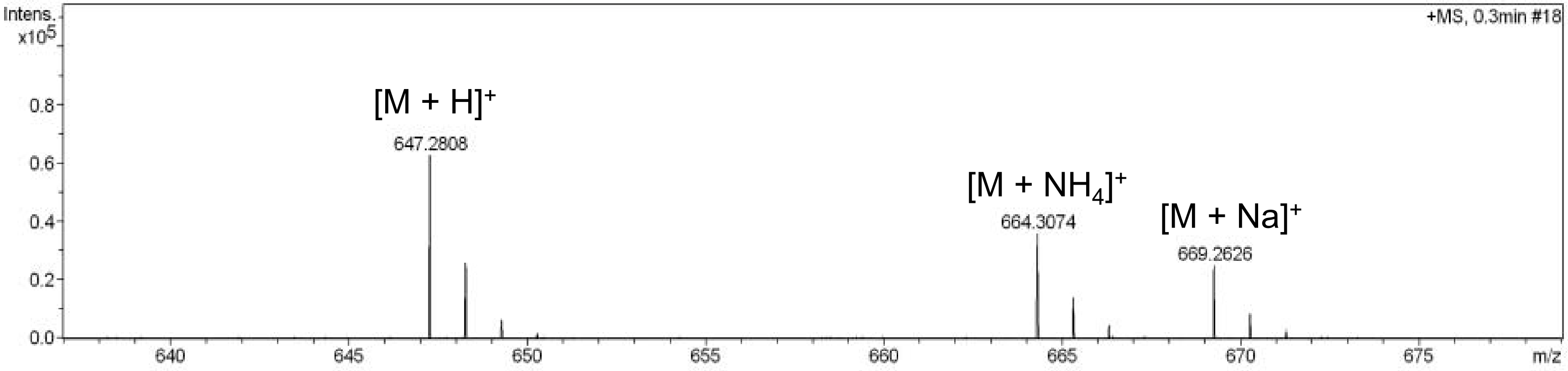

Meas. m/z	#	Ion Formula	Score	m/z	err [ppm]	err [mDa]	mSigma	rdb	e⁻ Conf	N-Rule
647.2808	1	C32H43N2O12	100.00	647.2811	-0.4	-0.2	26.3	12.5	even	ok
669.2626	1	C32H42N2NaO12	100.00	669.2630	-0.6	-0.4	14.4	12.5	even	ok

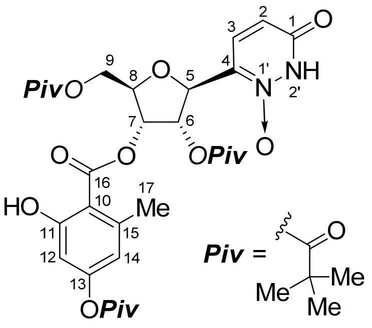

4

HR-ESIMS for **4**

Generic Display Report

Analysis Info

Analysis Name	D:\Data\MS\data\202110\wubinbin_WBB-145-D-R-6_pos_51_01_11733.d	Acquisition Date	10/27/2021 4:38:12 PM
Method	LC_Direct Infusion_pos_70-500mz.m	Operator	SCSIO
Sample Name	wubinbin_WBB-145-D-R-6_pos	Instrument	maXis
Comment			

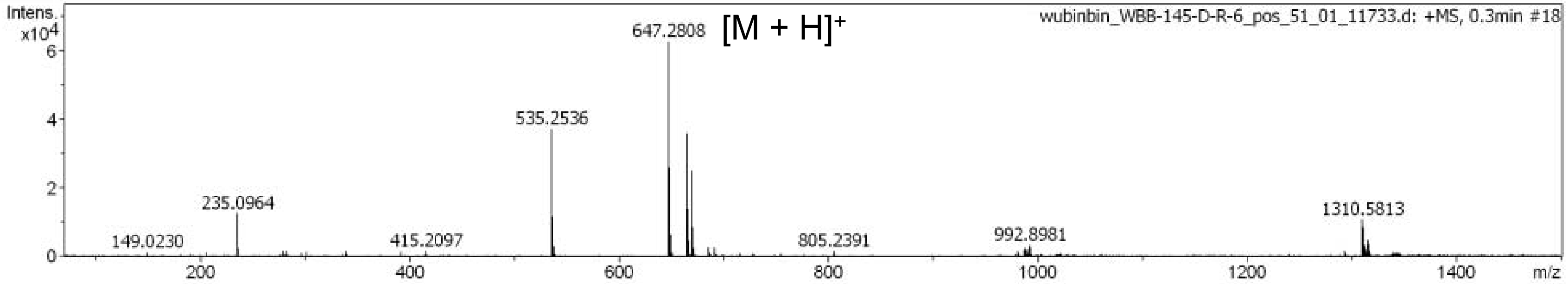

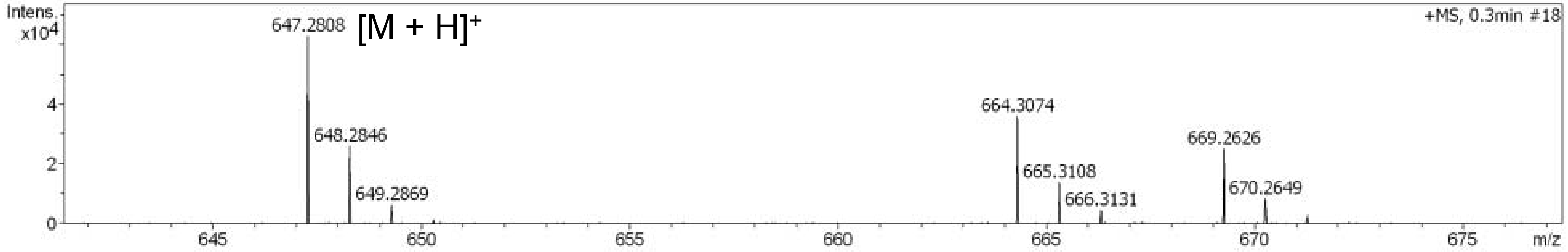

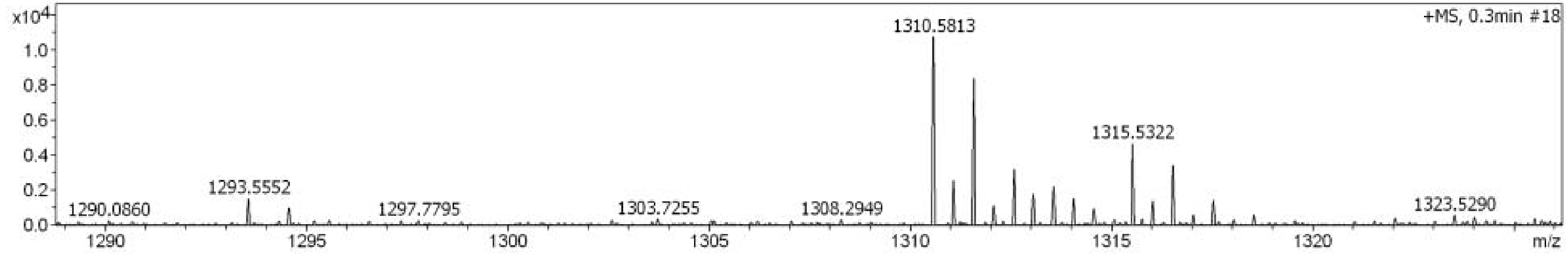

^{1}H (700 MHz) NMR spectrum of **4** in $CDCl_3$

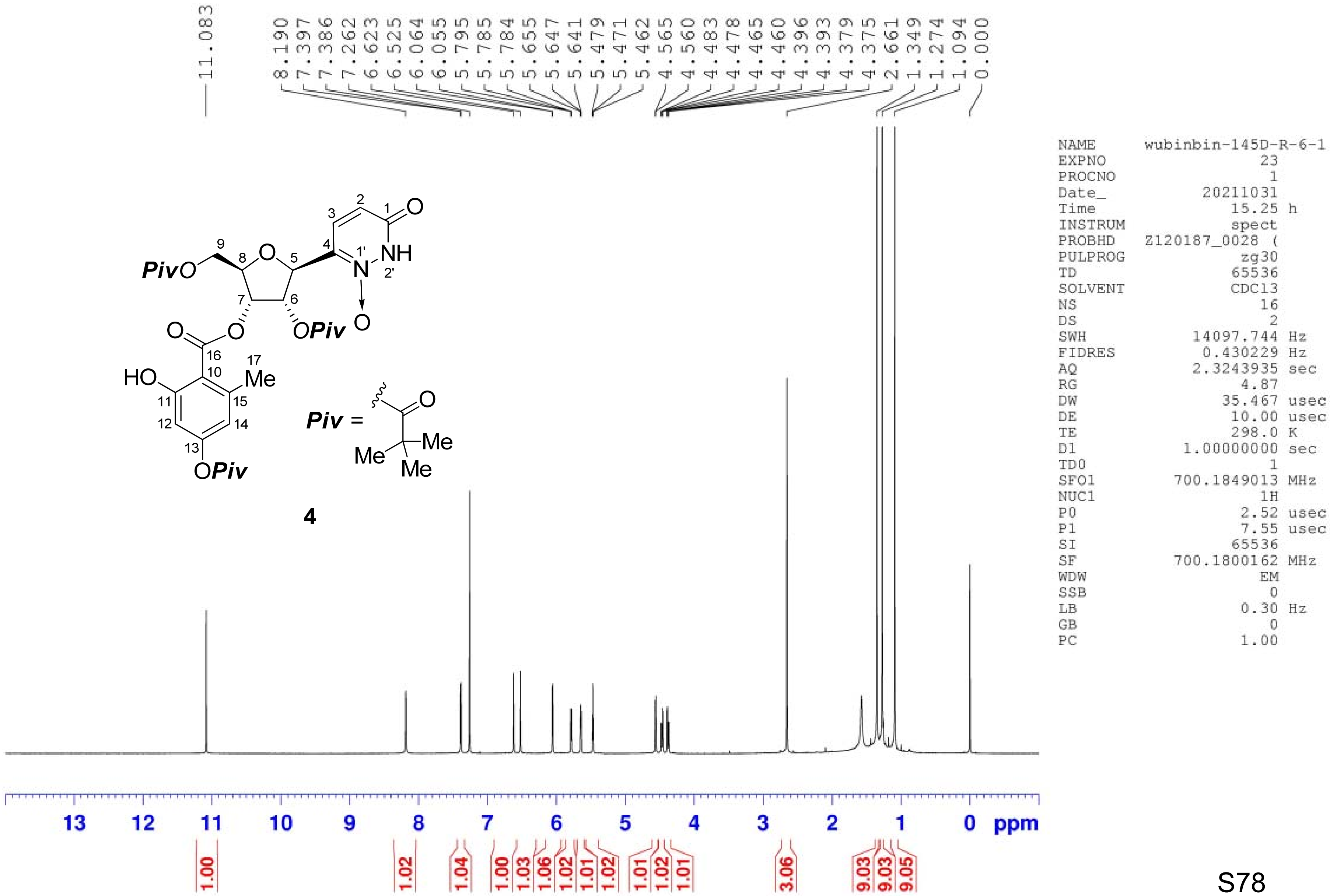

^{1}H (700 MHz) NMR spectrum of **4** in $CDCl_3$

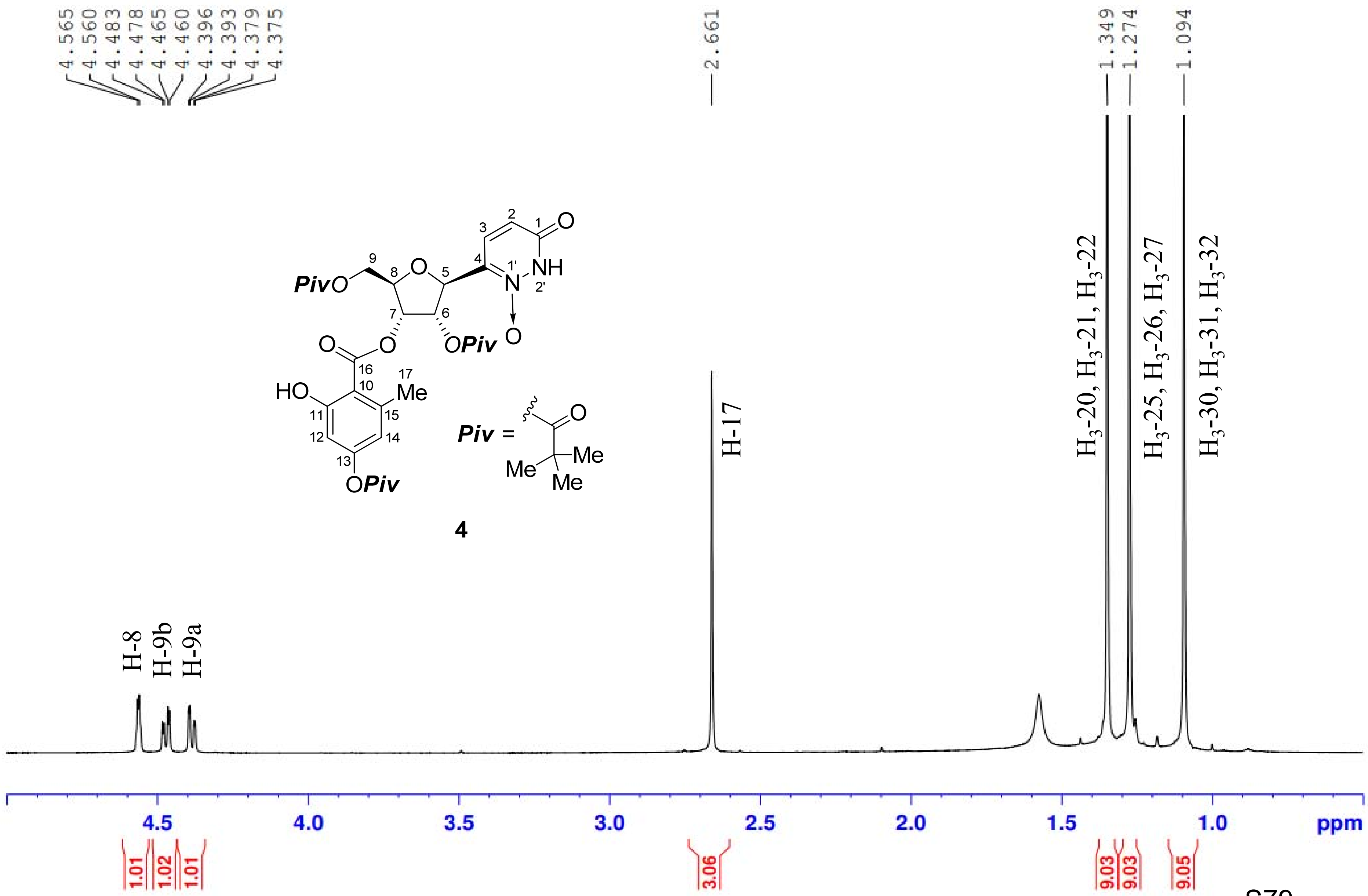

^{1}H (700 MHz) NMR spectrum of **4** in $CDCl_3$

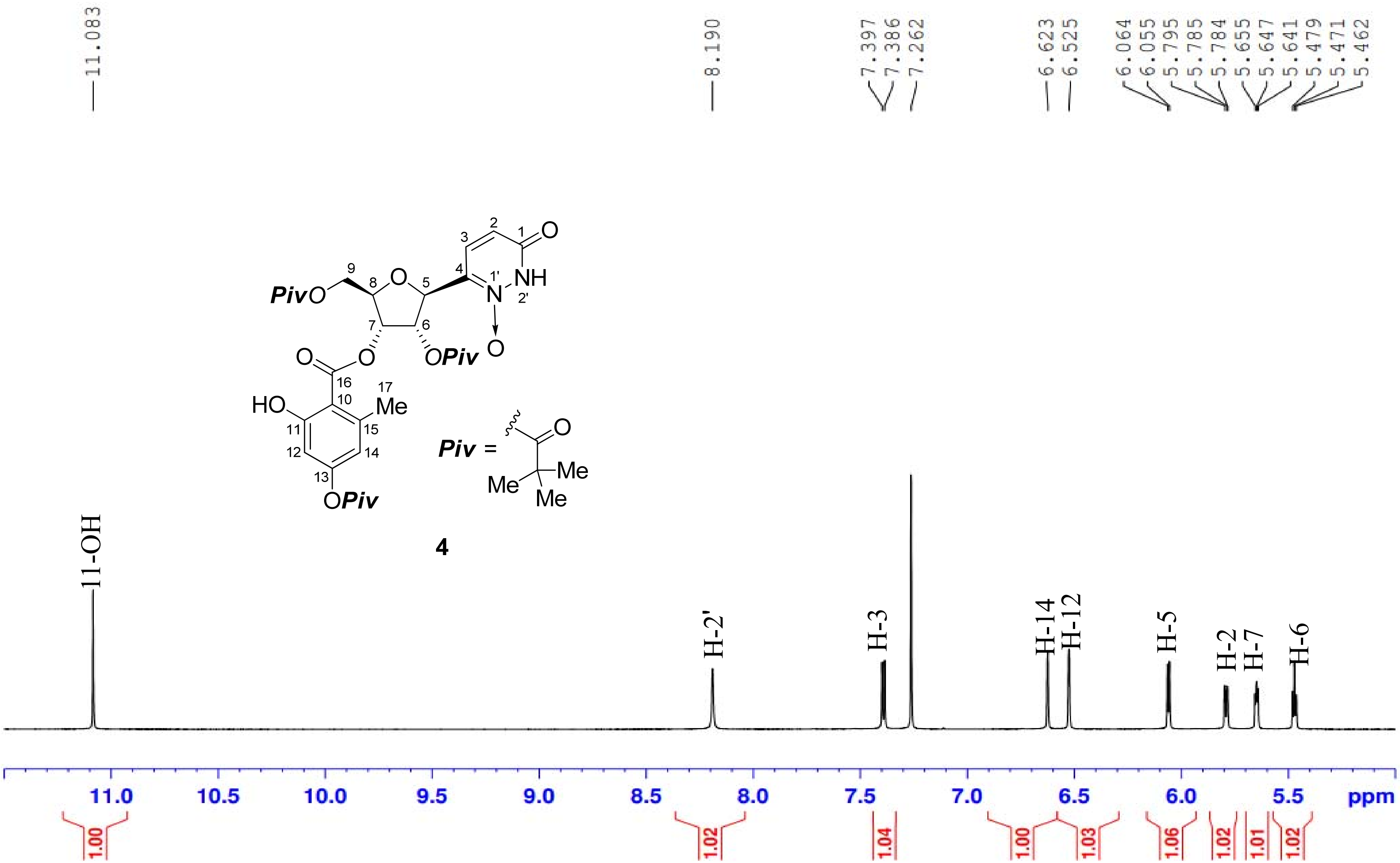

^{13}C (175 MHz) NMR spectrum of **4** in $CDCl_3$

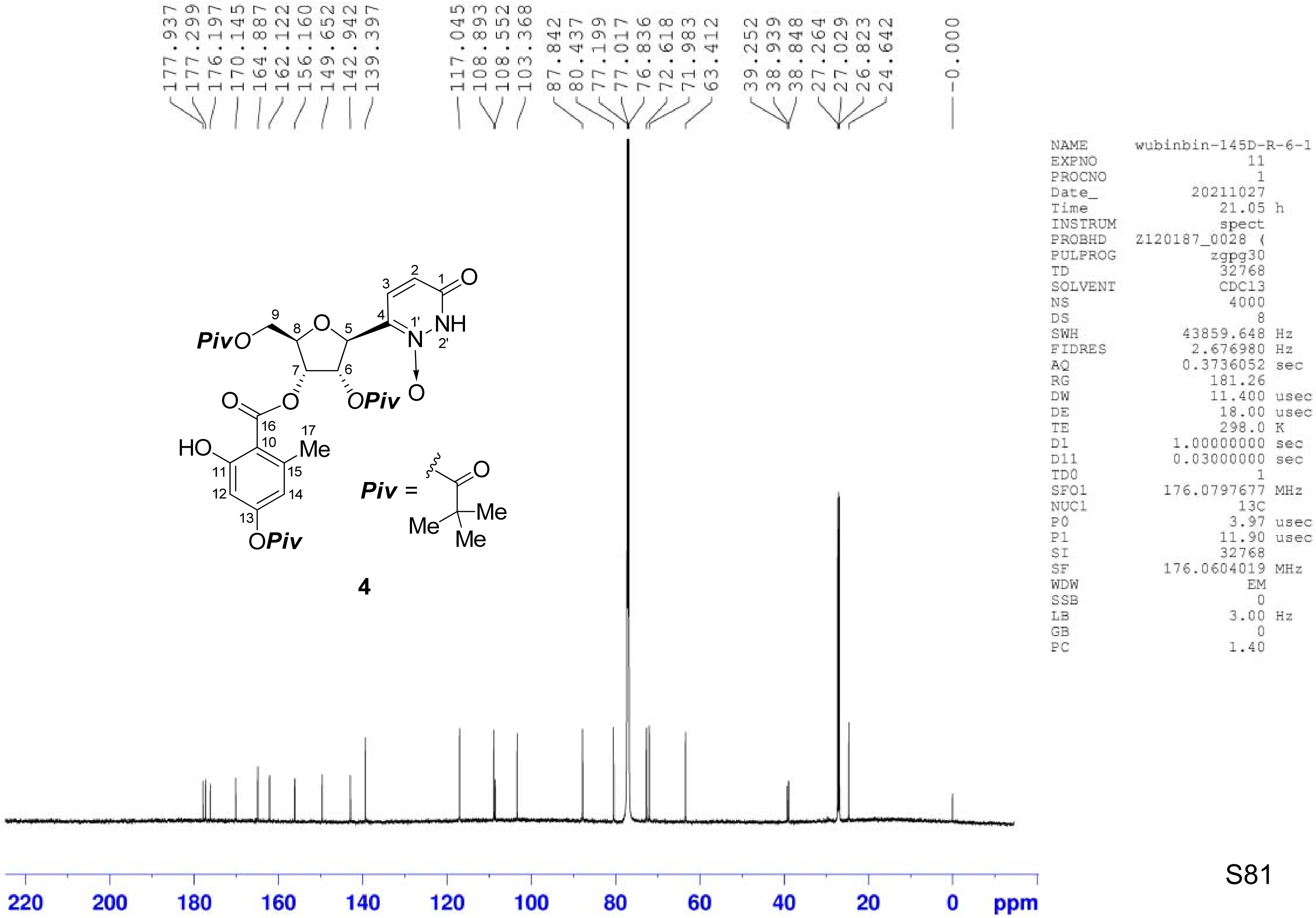

^{13}C (175 MHz) NMR spectrum of **4** in $CDCl_3$

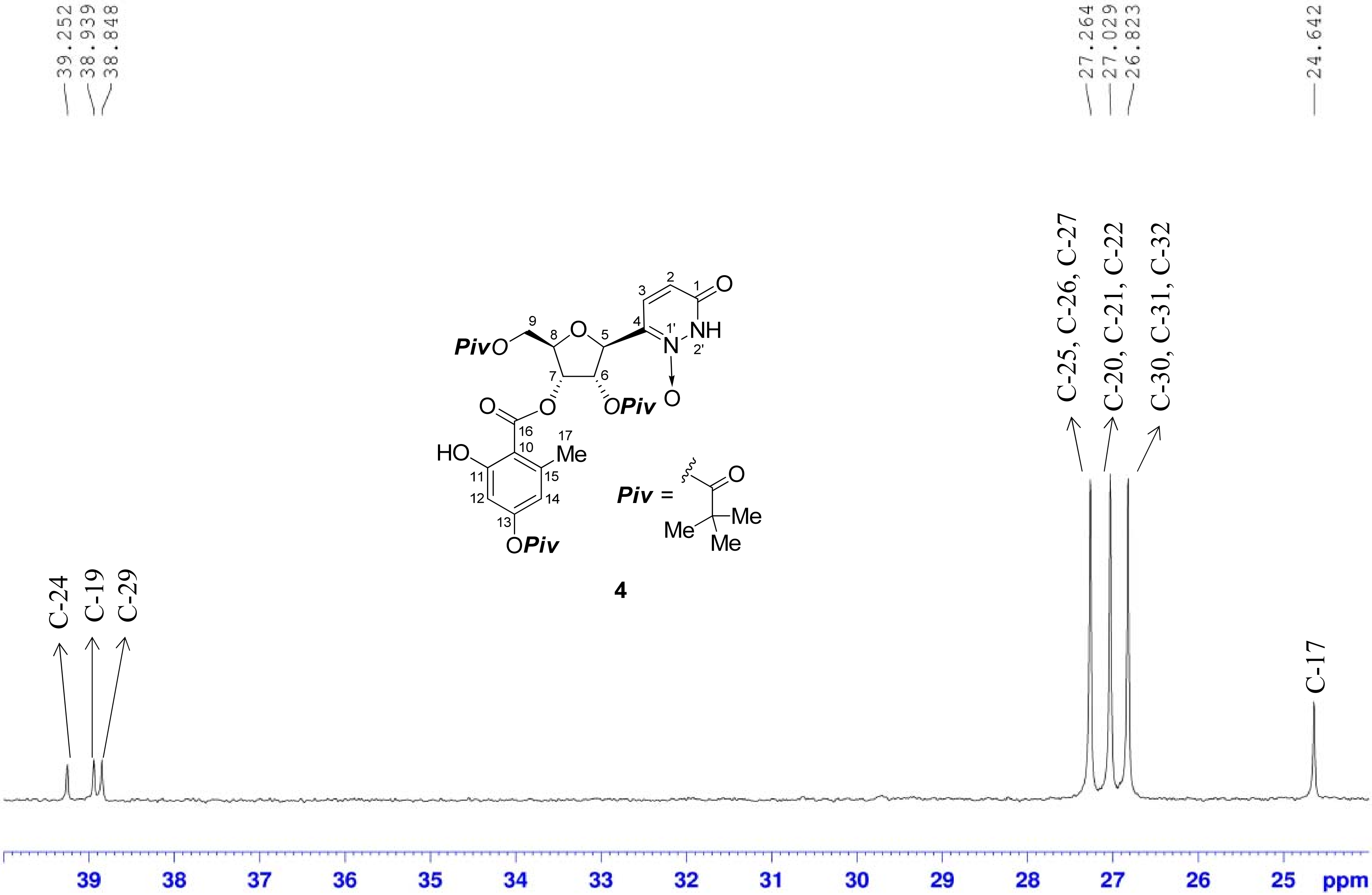

^{13}C (175 MHz) NMR spectrum of **4** in $CDCl_3$

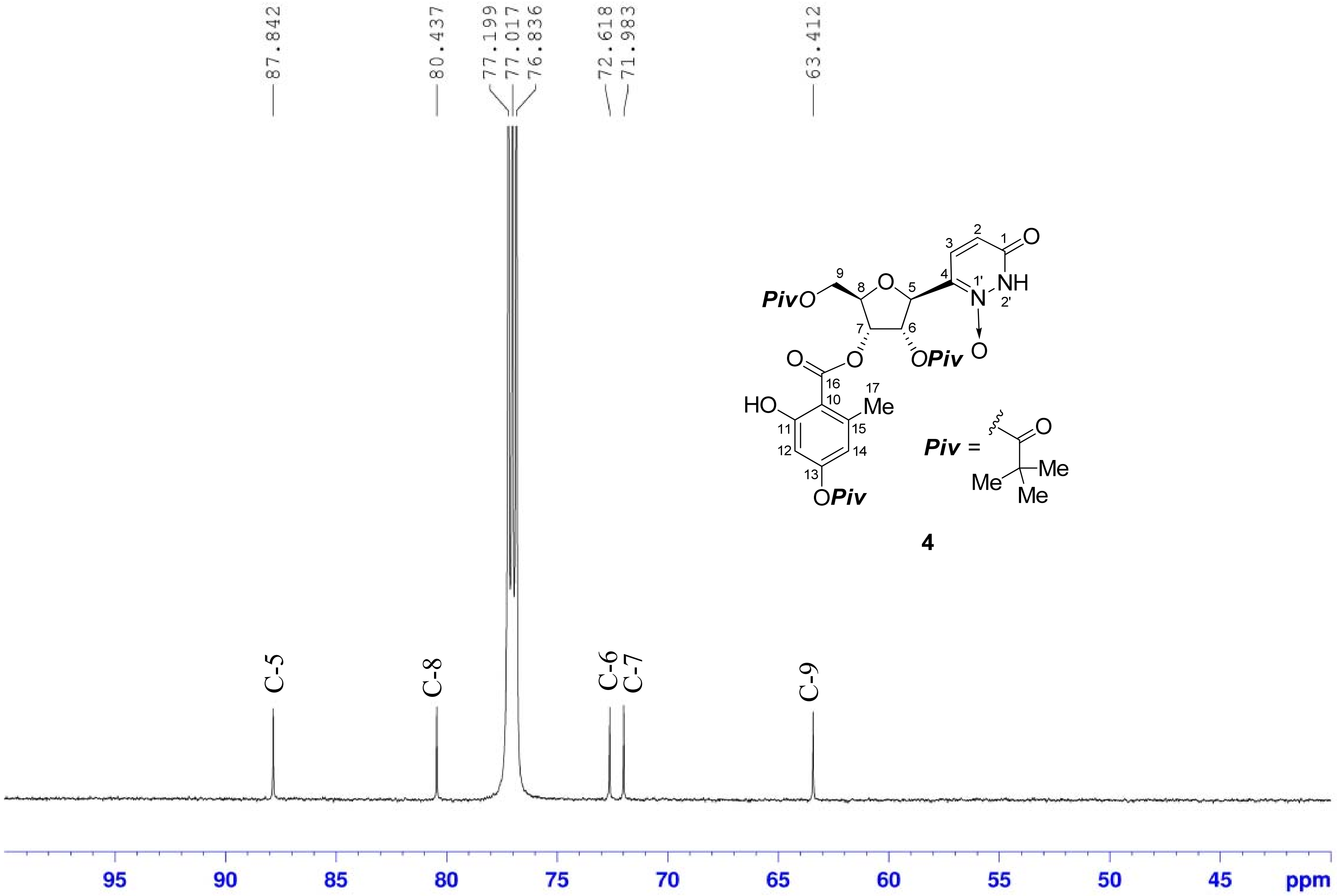

^{13}C (175 MHz) NMR spectrum of **4** in $CDCl_3$

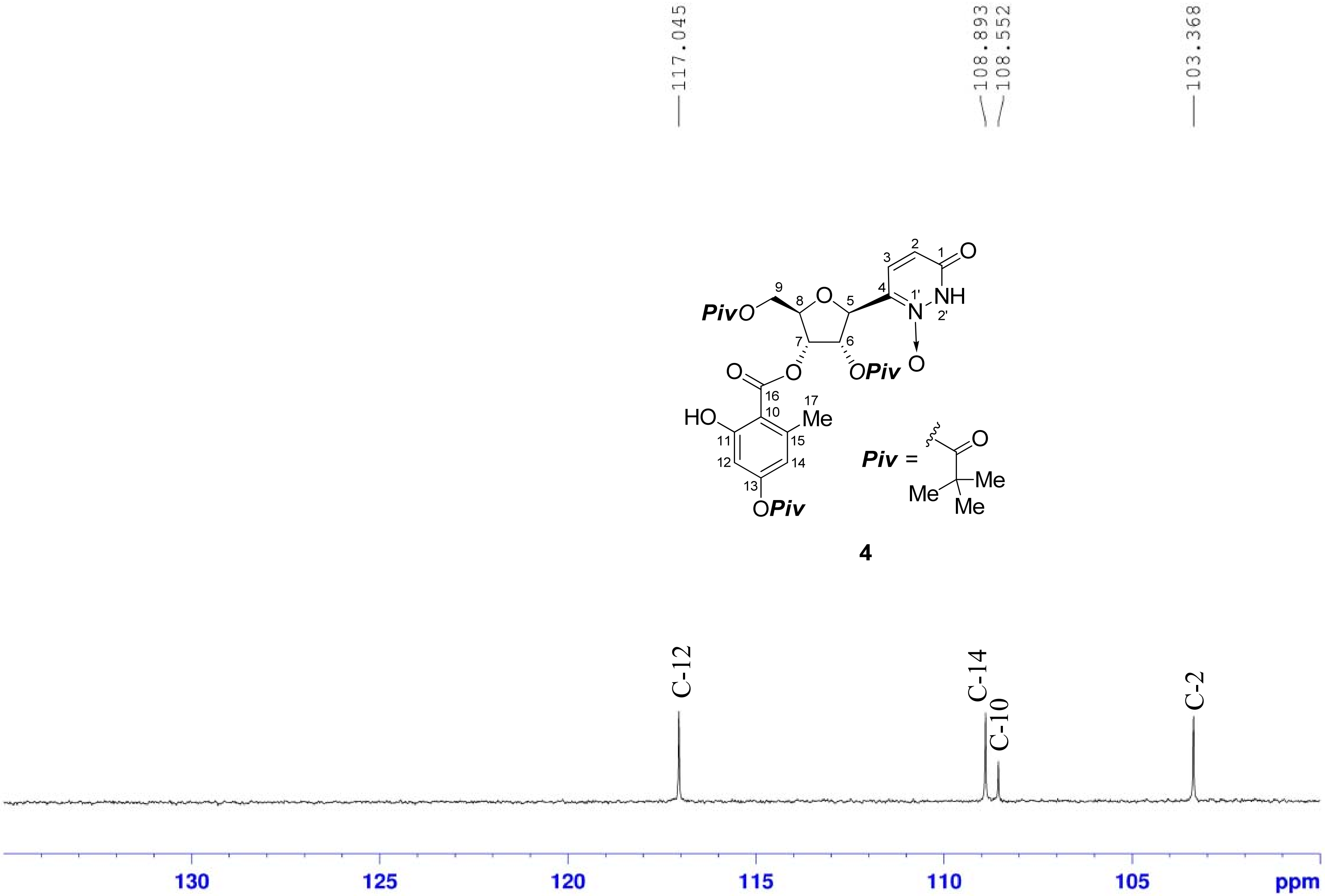

^{13}C (175 MHz) NMR spectrum of **4** in $CDCl_3$

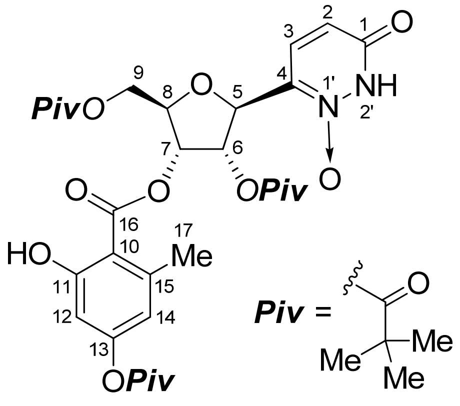

4

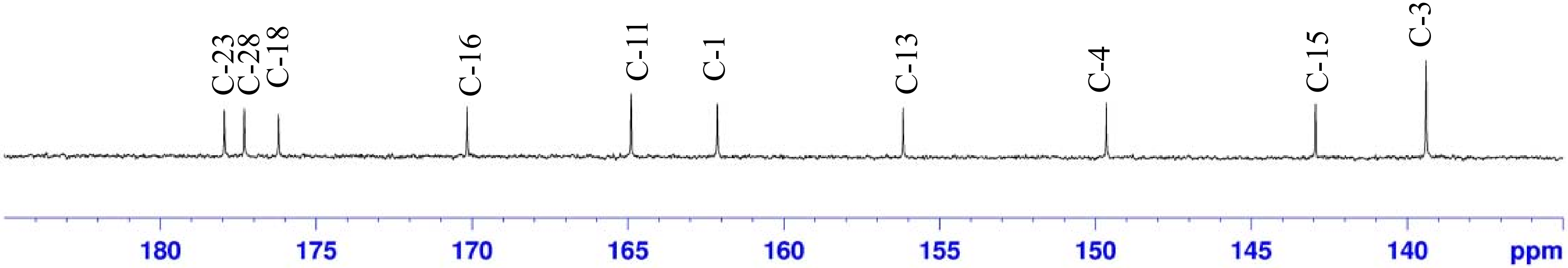

DEPT135 (175 MHz) spectrum of **4** in $CDCl_3$

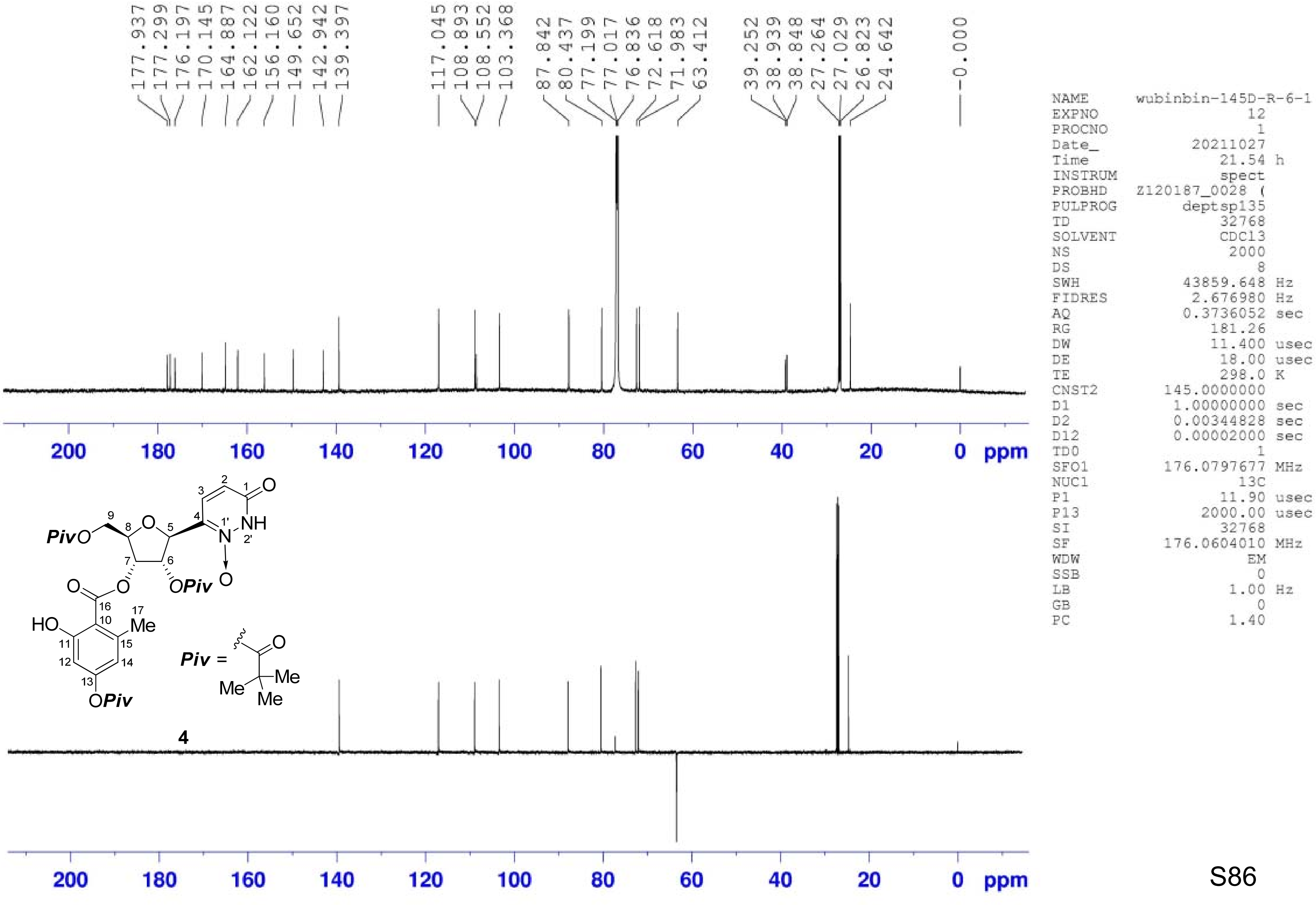

DEPT135 (175 MHz) spectrum of **4** in $CDCl_3$

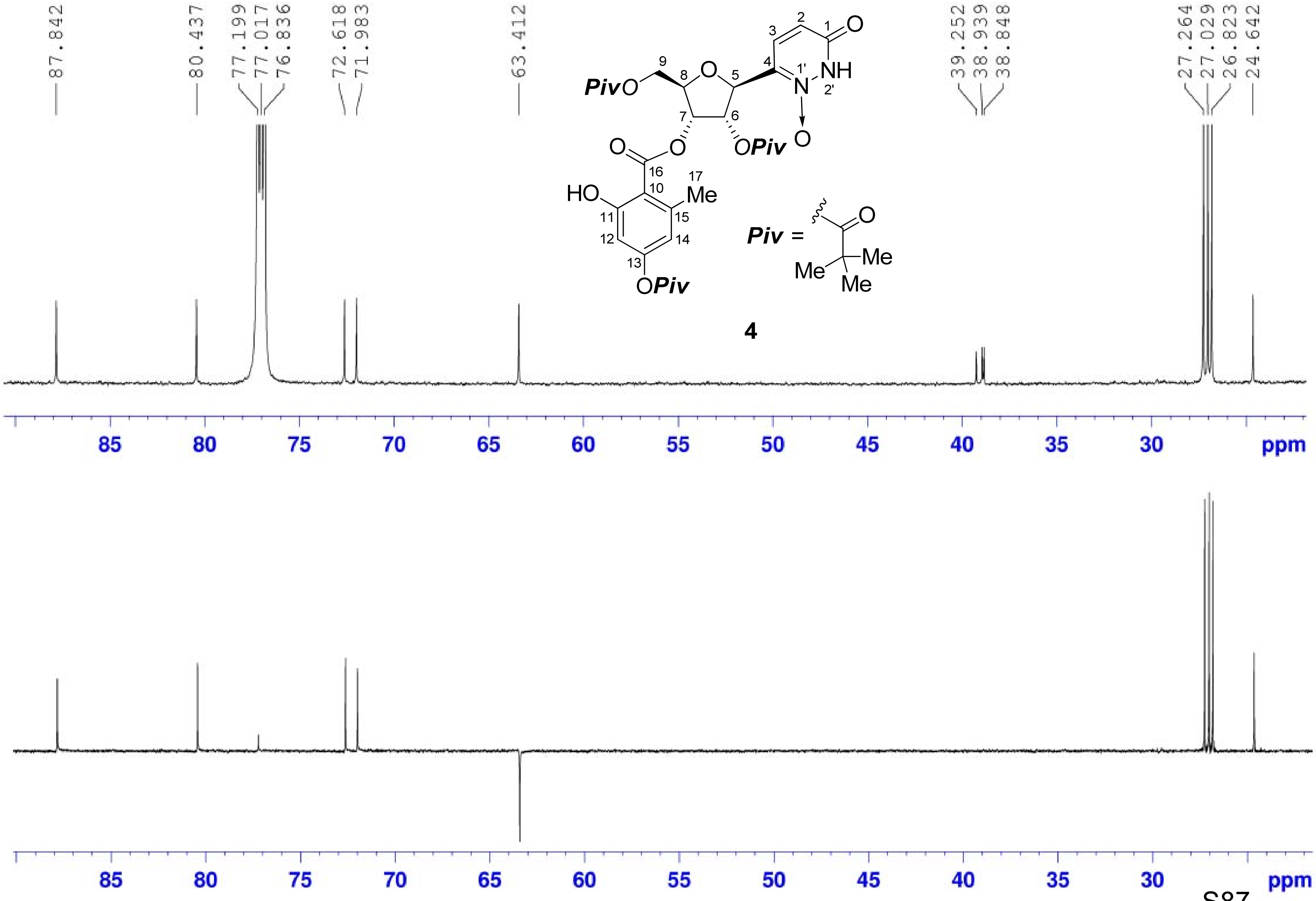

DEPT135 (175 MHz) spectrum of **4** in $CDCl_3$

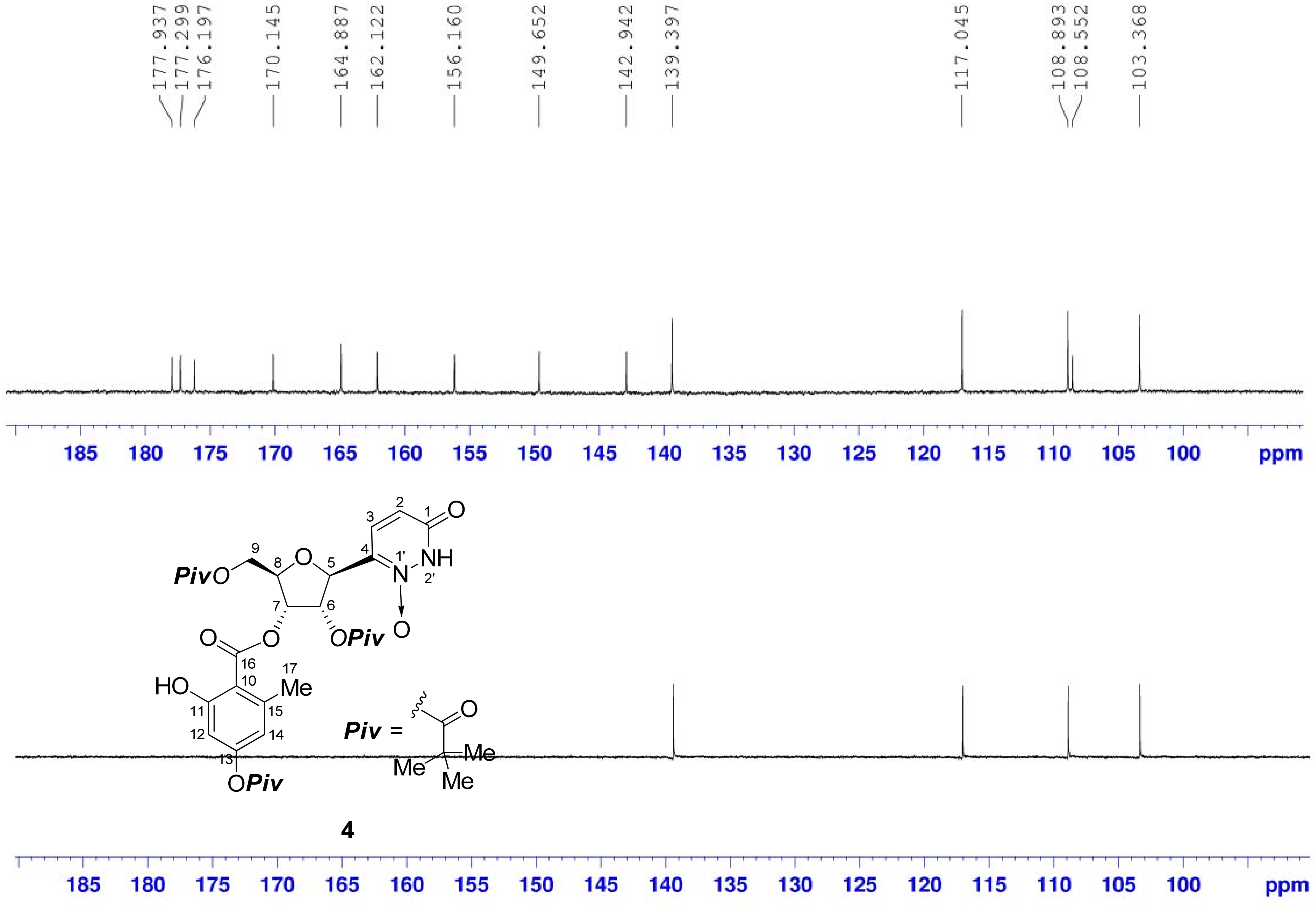

4

^{1}H–^{1}H COSY (700 MHz) spectrum of **4** in $CDCl_3$

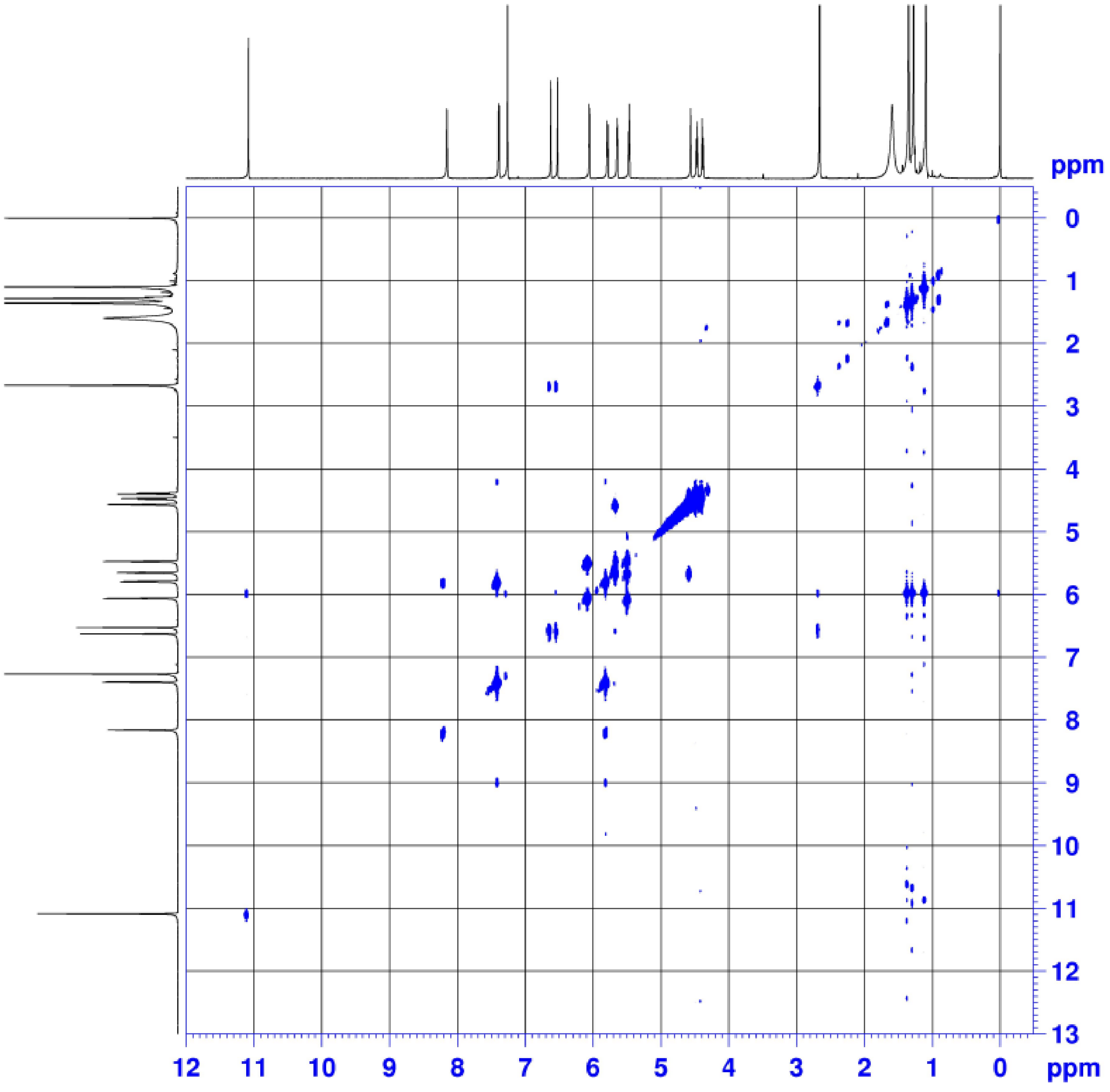

NAME	wubinbin-145D-R-6-1	
EXPNO	17	
PROCNO	1	
Date_	20211029	
Time	21.22	h
INSTRUM	spect	
PROBHD	Z120187_0028 (	
PULPROG	cosygpmfqf	
TD	2048	
SOLVENT	CDCl3	
NS	16	
DS	16	
SWH	9090.909	Hz
FIDRES	8.877841	Hz
AQ	0.1126900	sec
RG	181.26	
DW	55.000	usec
DE	10.00	usec
TE	298.0	K
D0	0.00000300	sec
D1	1.00000000	sec
D13	0.00000400	sec
D16	0.00020000	sec
IN0	0.00010980	sec
ND0	1	
TD	128	
SFO1	700.1842	MHz
FIDRES	71.152092	Hz
SW	13.007	ppm
FnMODE	QF	
SI	1024	
SF	700.1799963	MHz
WDW	SINE	
SSB	0	
LB	0.00	Hz
GB	0	
PC	1.40	
SI	1024	
MC2	QF	
SF	700.1799963	MHz
WDW	SINE	
SSB	0	
LB	0.00	Hz
GB	0	

^1H–^1H COSY (700 MHz) spectrum of **4** in $CDCl_3$

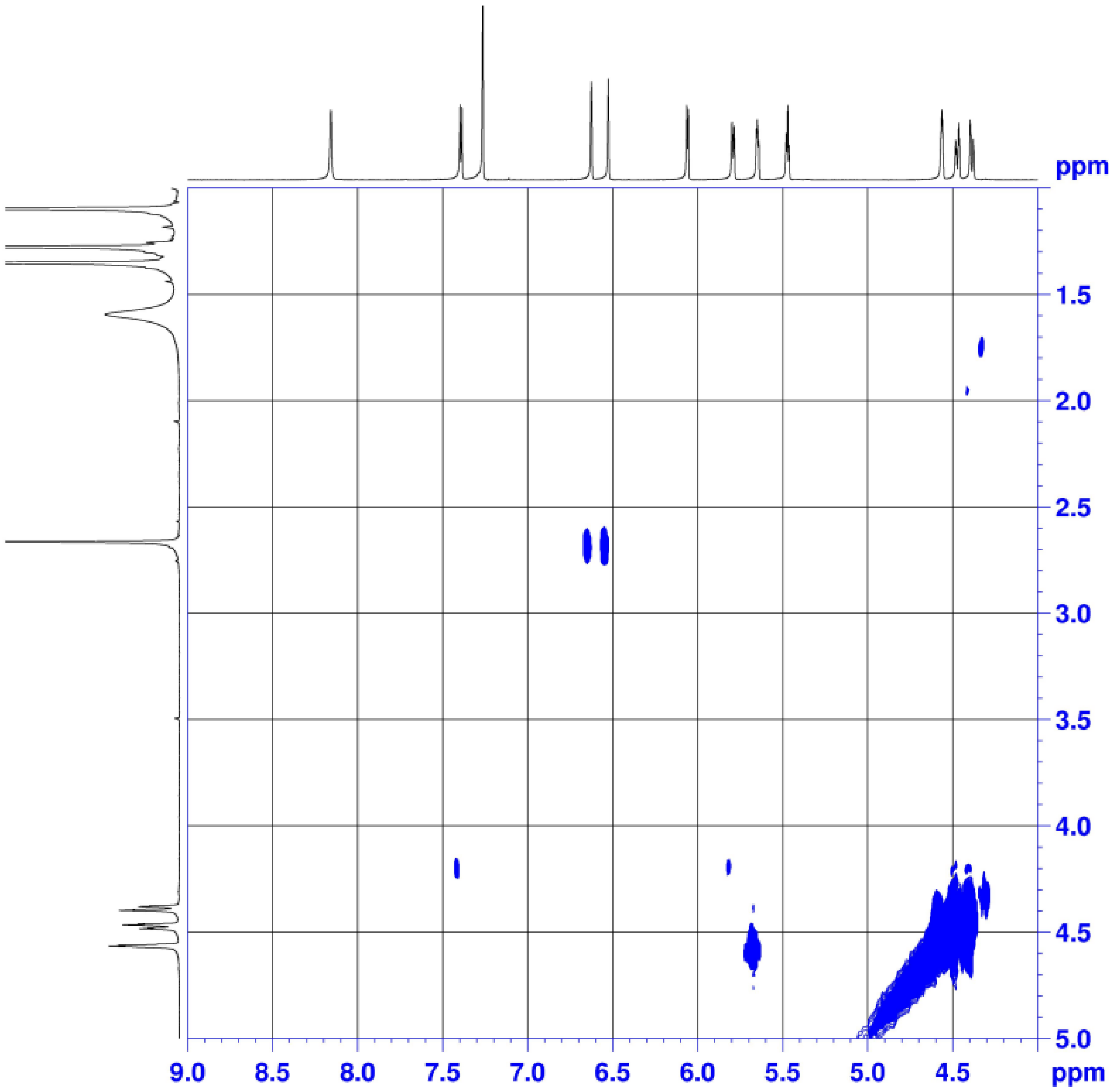

^1H–^1H COSY (700 MHz) spectrum of **4** in $CDCl_3$

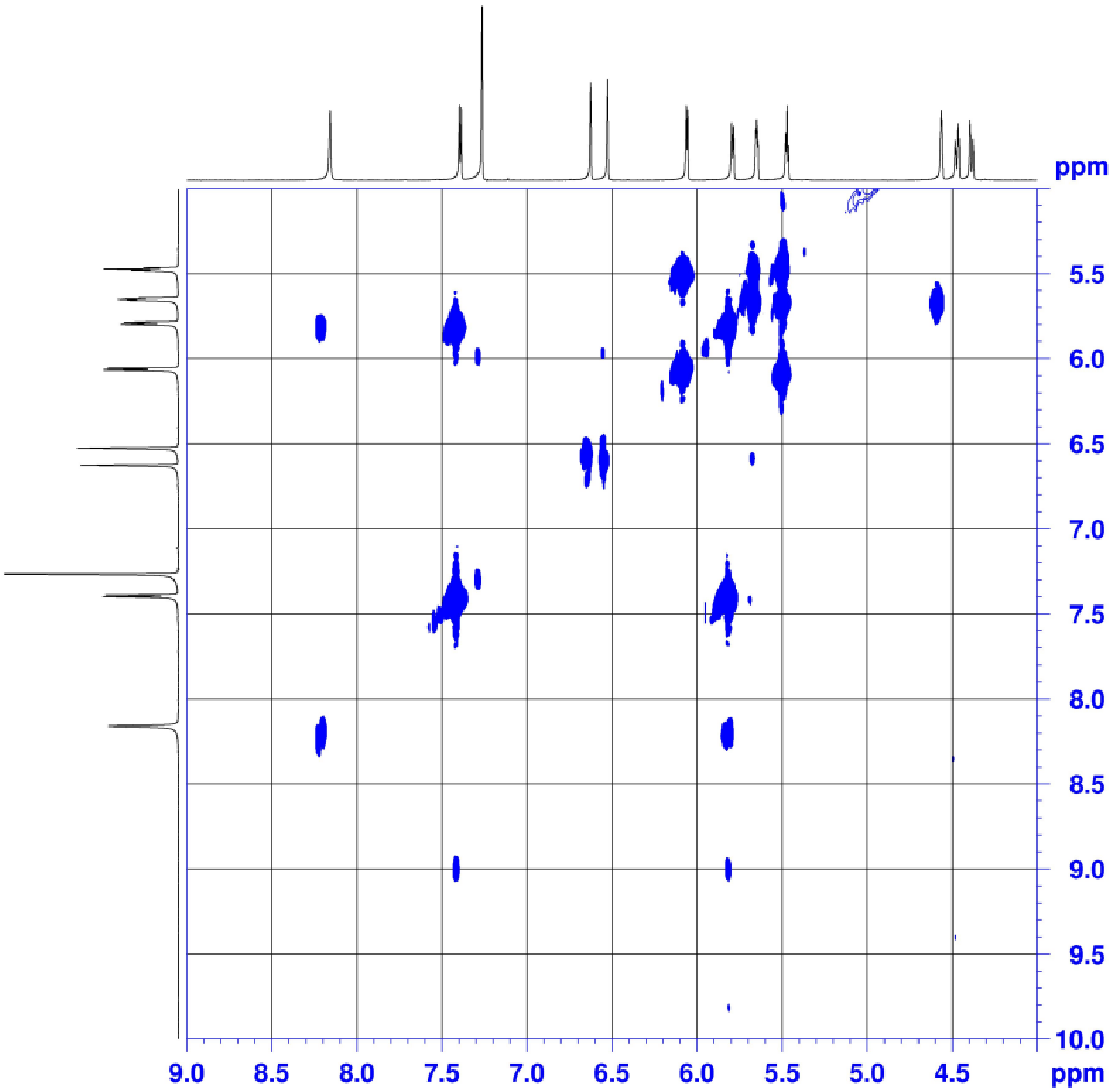

HSQC (700 MHz) spectrum of **4** in $CDCl_3$

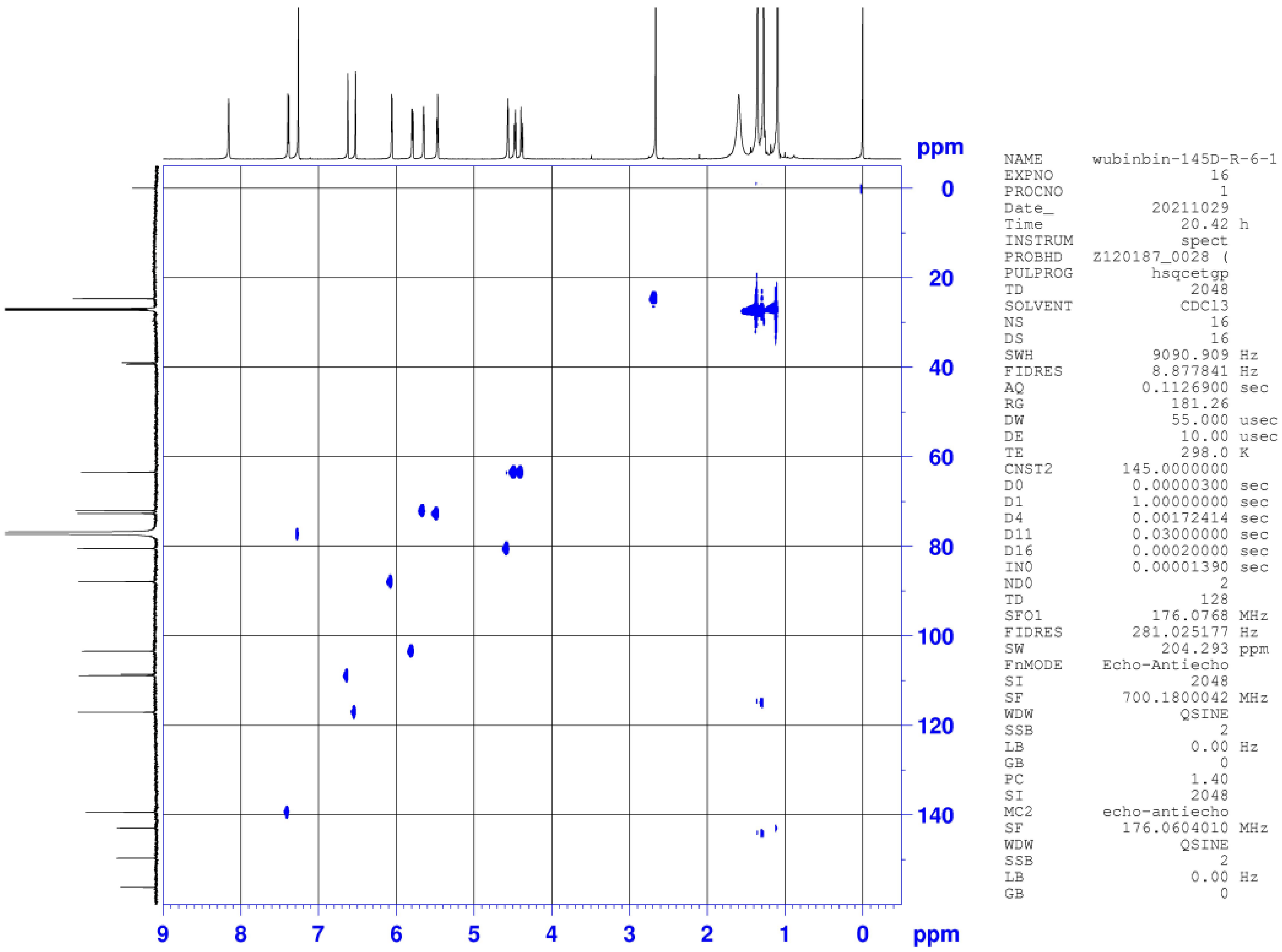

HSQC (700 MHz) spectrum of **4** in $CDCl_3$

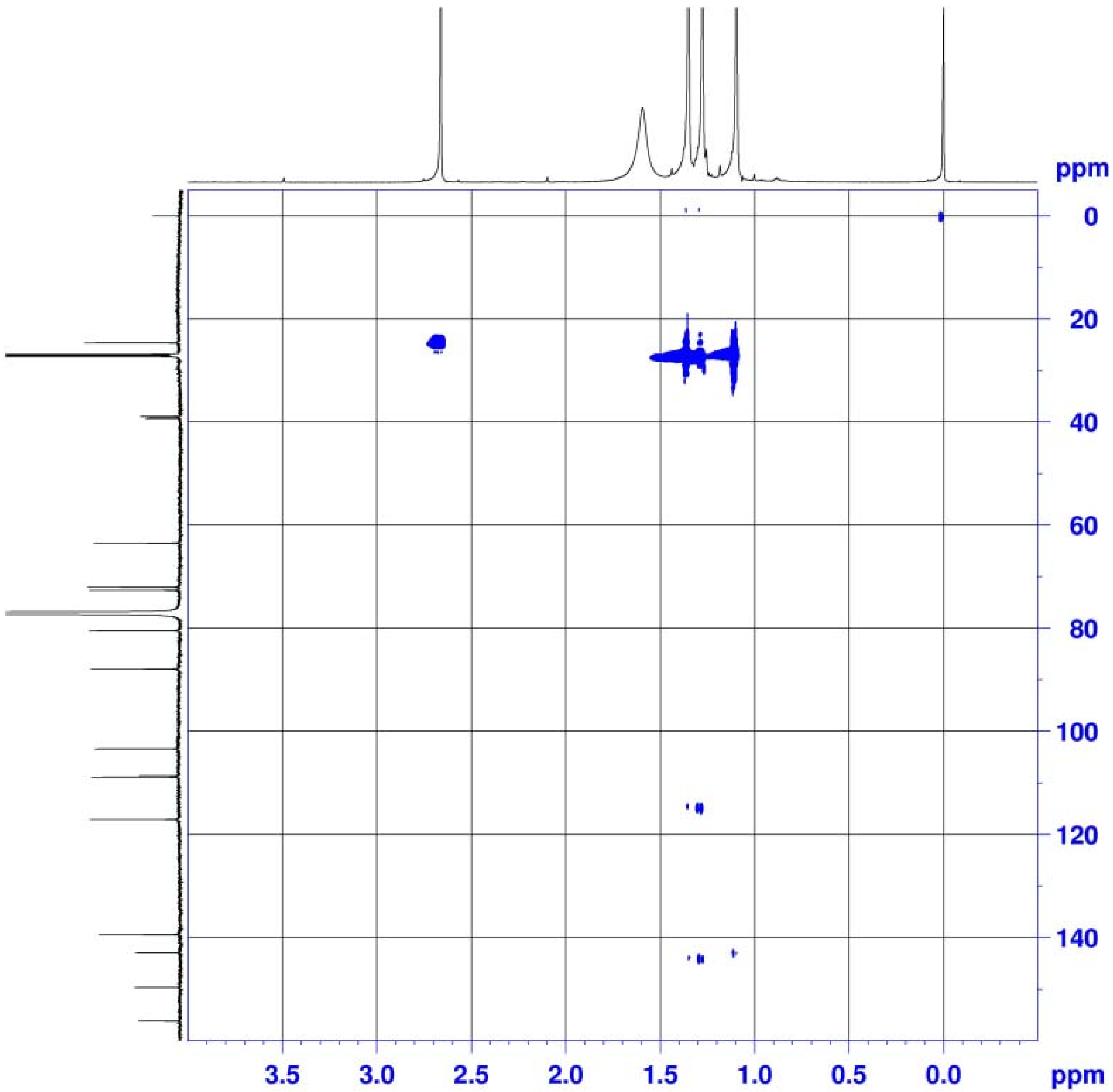

HSQC (700 MHz) spectrum of **4** in $CDCl_3$

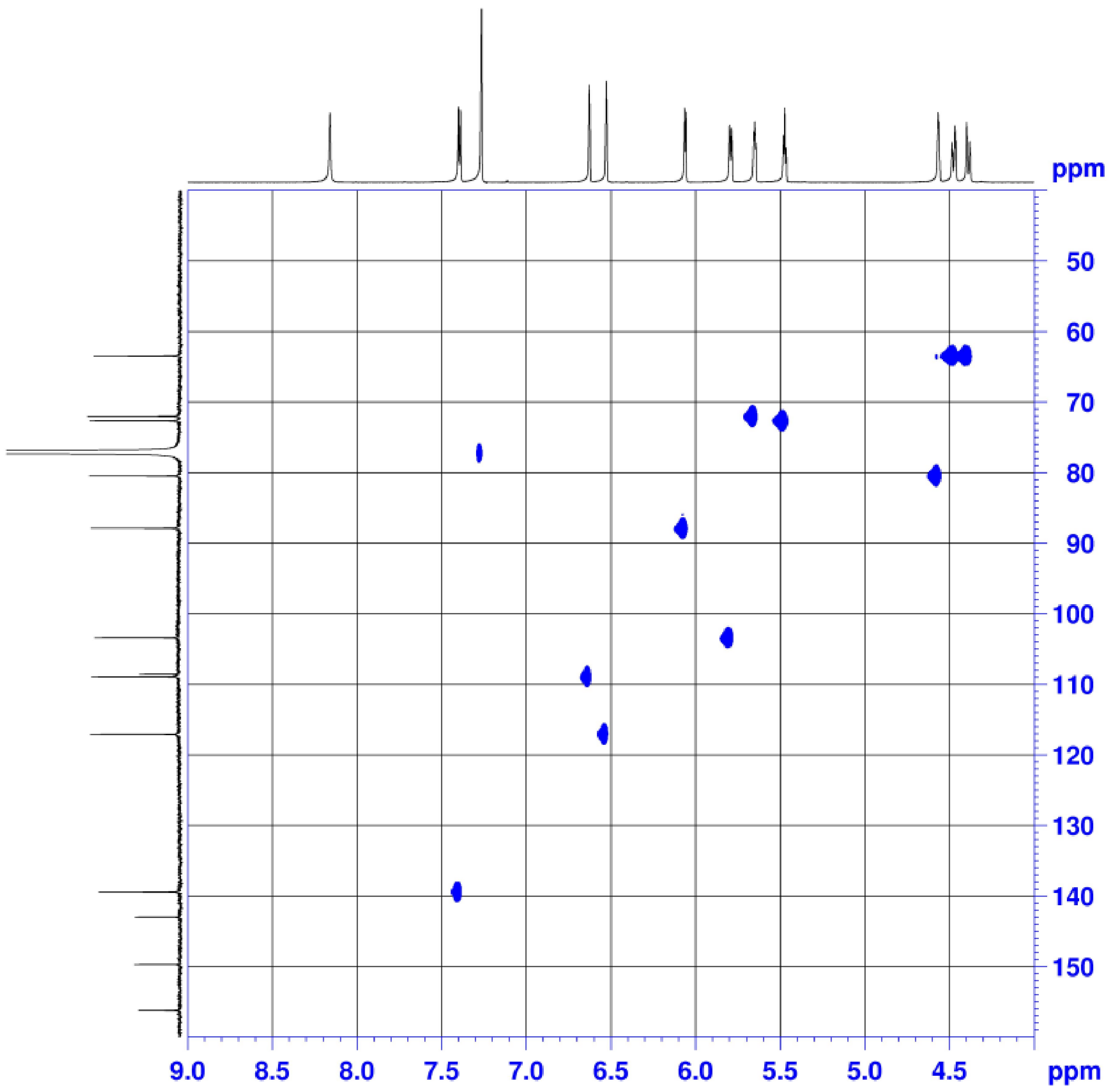

HMBC (700 MHz) spectrum of **4** in $CDCl_3$

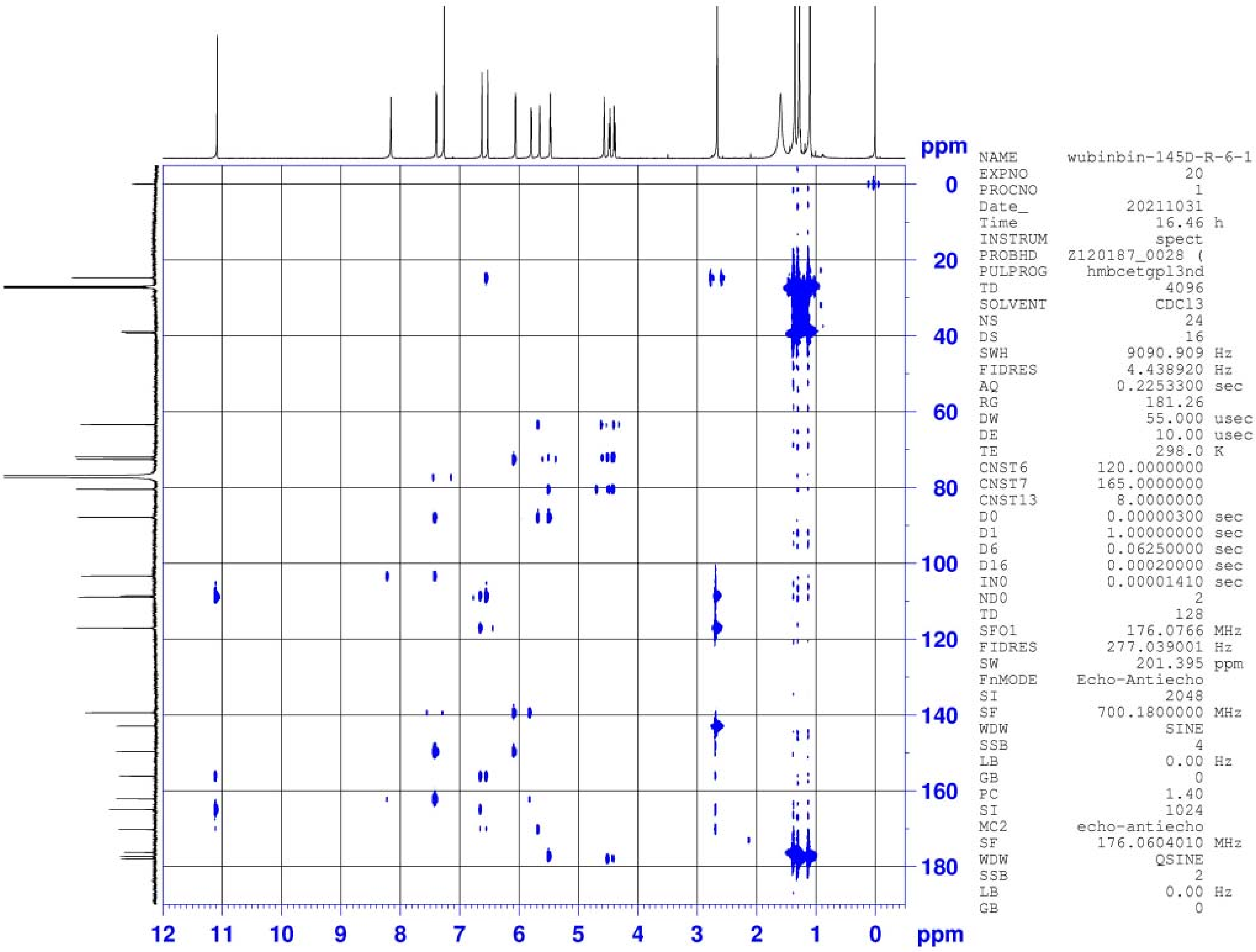

HMBC (700 MHz) spectrum of **4** in $CDCl_3$

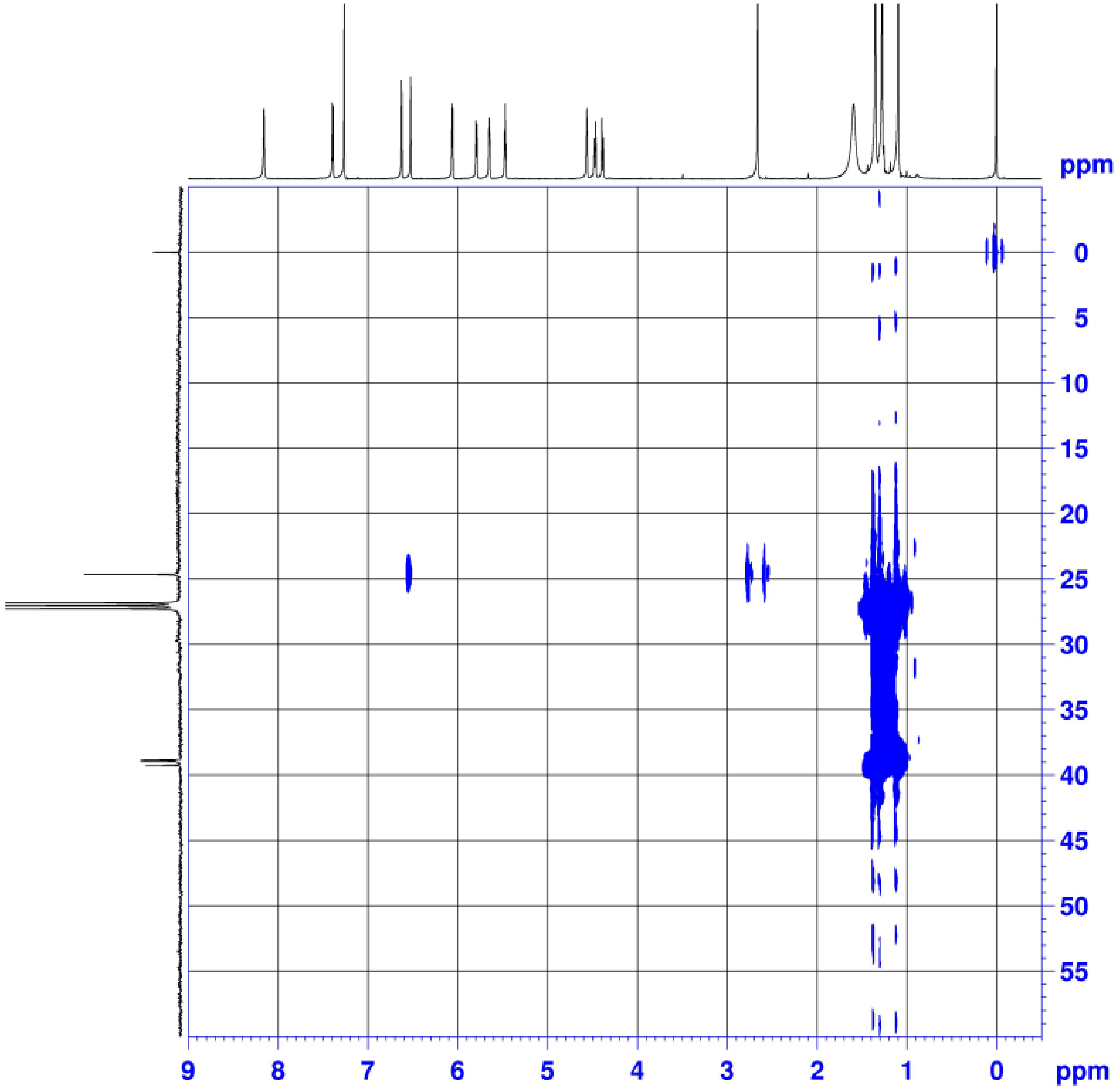

HMBC (700 MHz) spectrum of **4** in $CDCl_3$

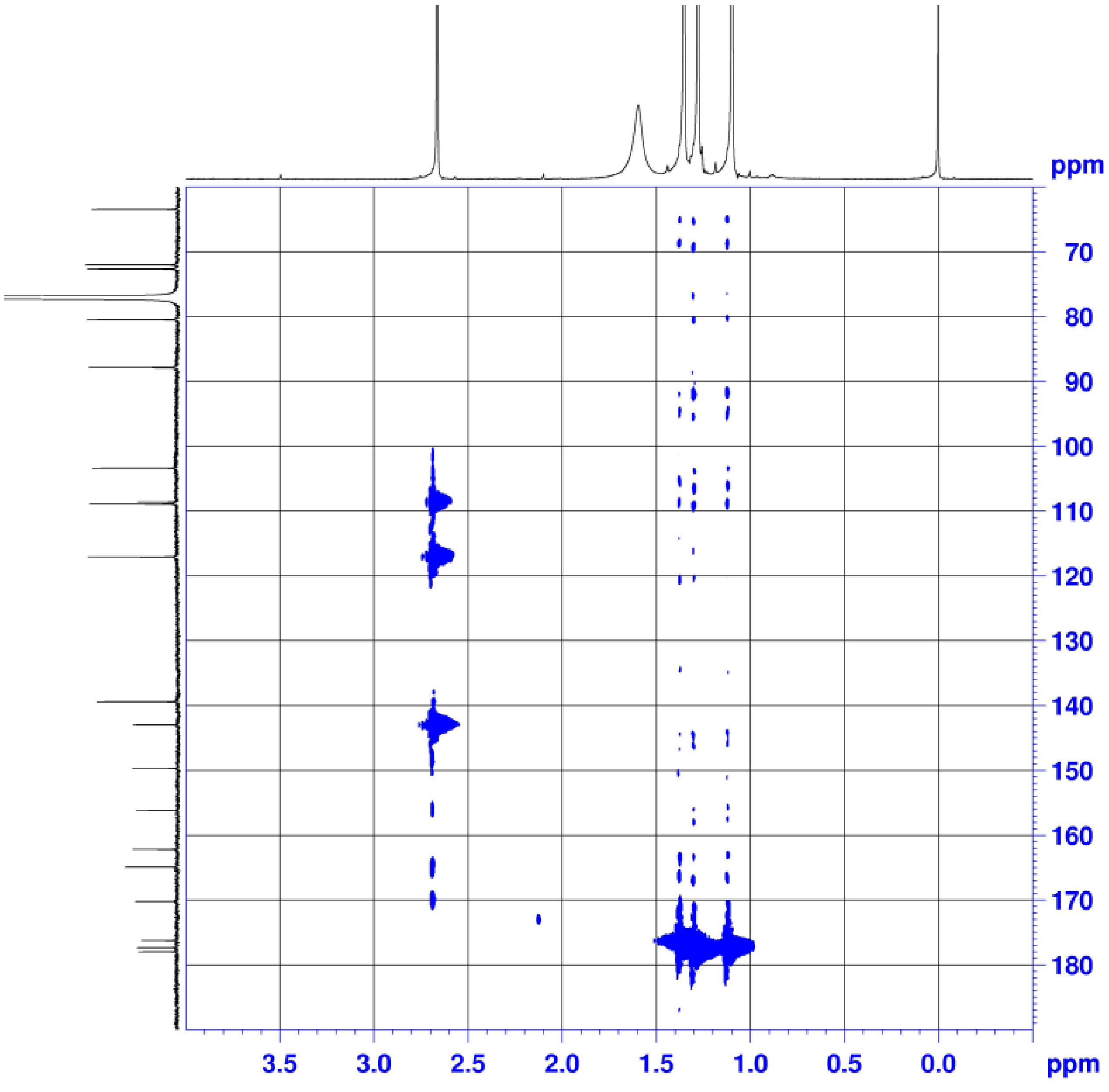

HMBC (700 MHz) spectrum of **4** in $CDCl_3$

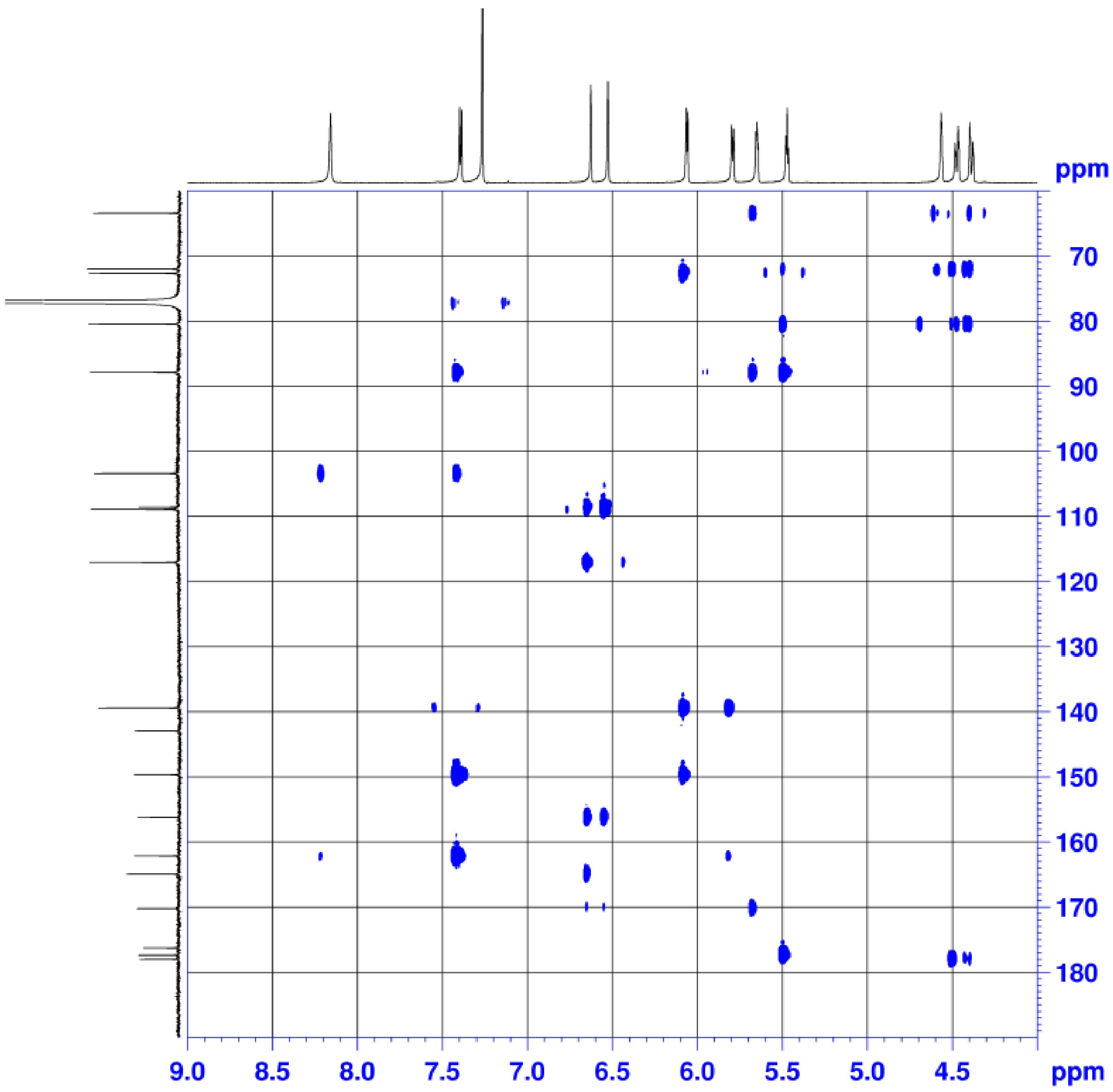

HMBC (700 MHz) spectrum of **4** in $CDCl_3$

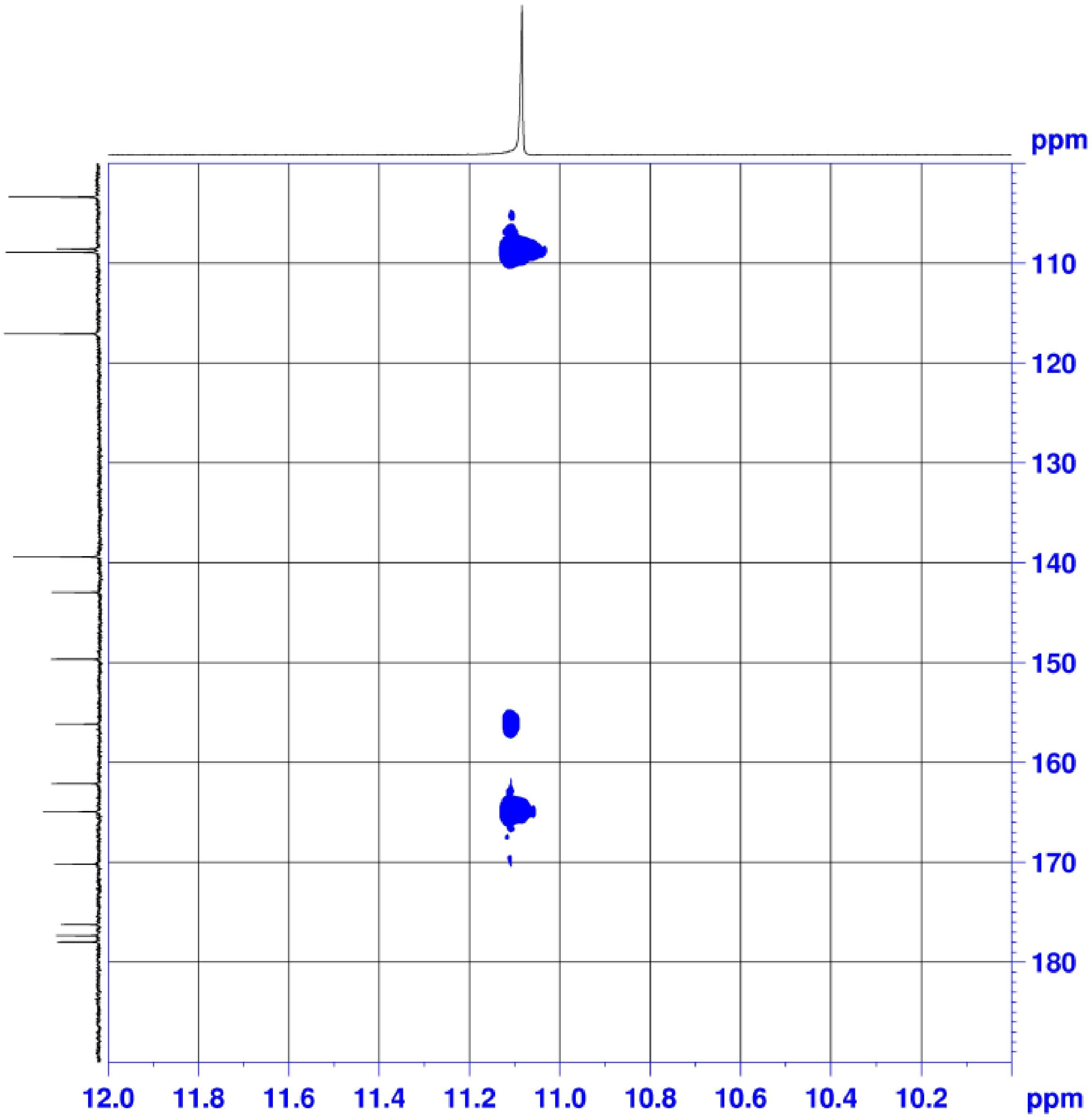

NOESY (700 MHz) spectrum of **4** in $CDCl_3$

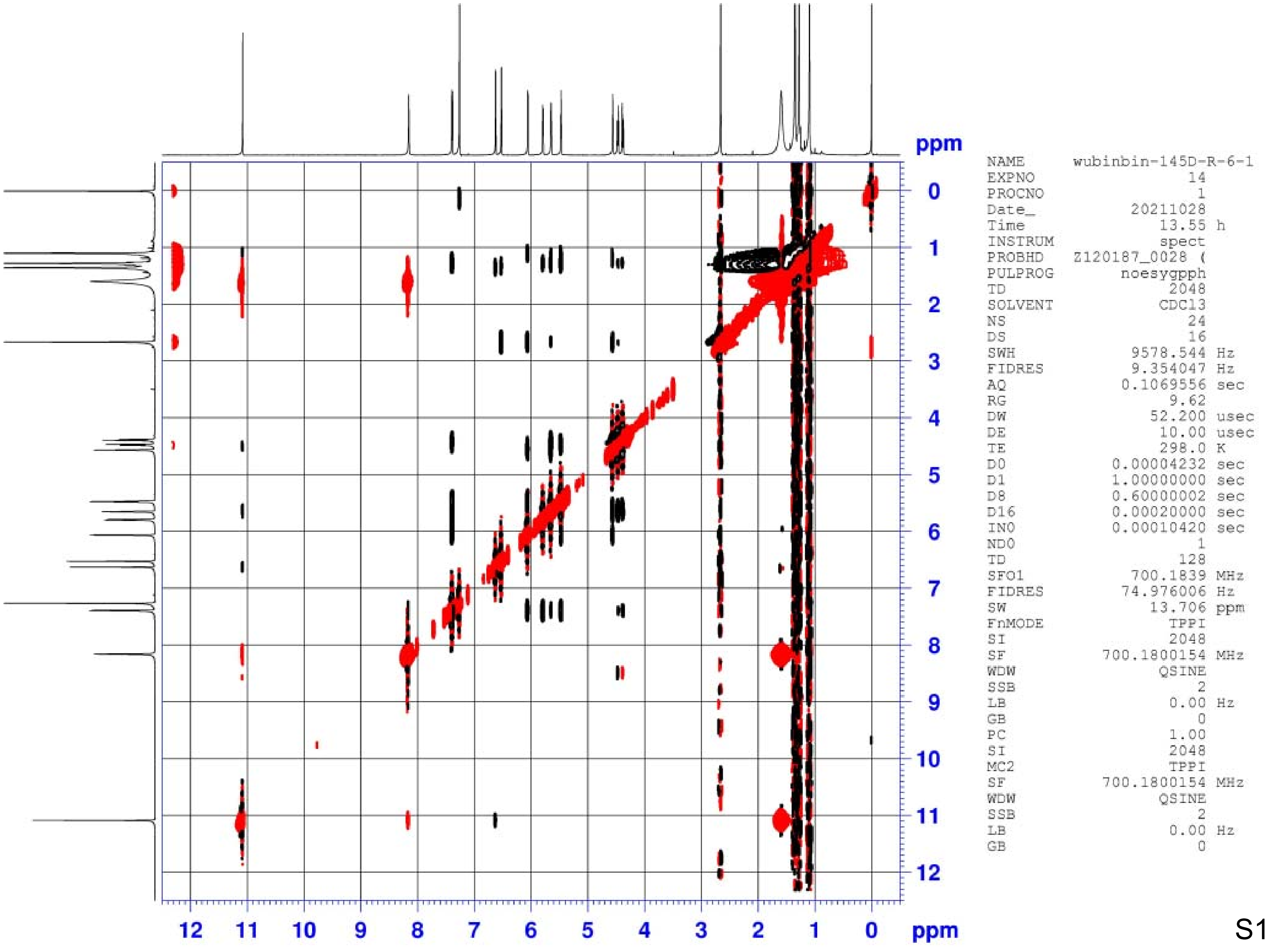

NOESY (700 MHz) spectrum of **4** in $CDCl_3$

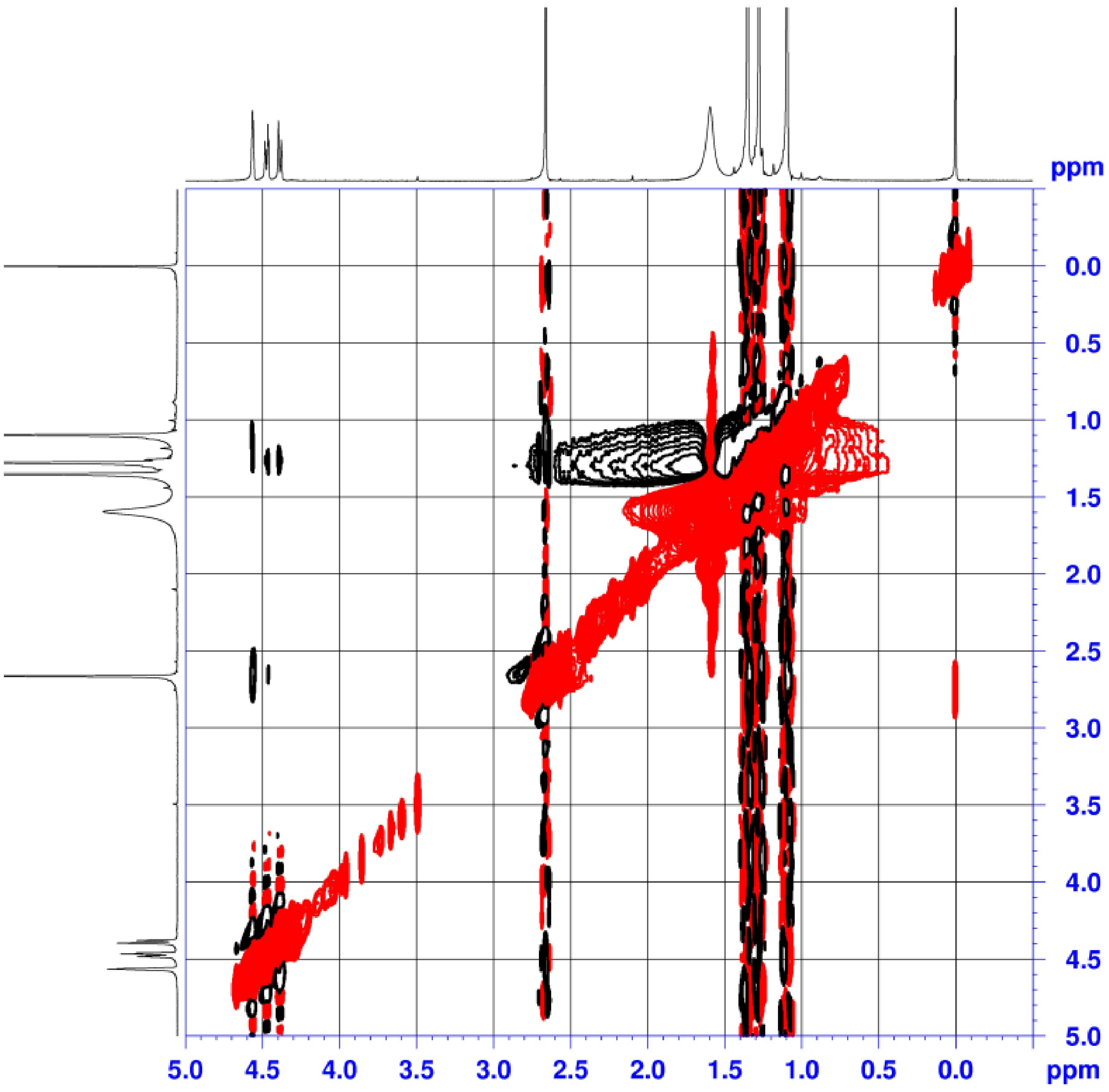

NOESY (700 MHz) spectrum of **4** in $CDCl_3$

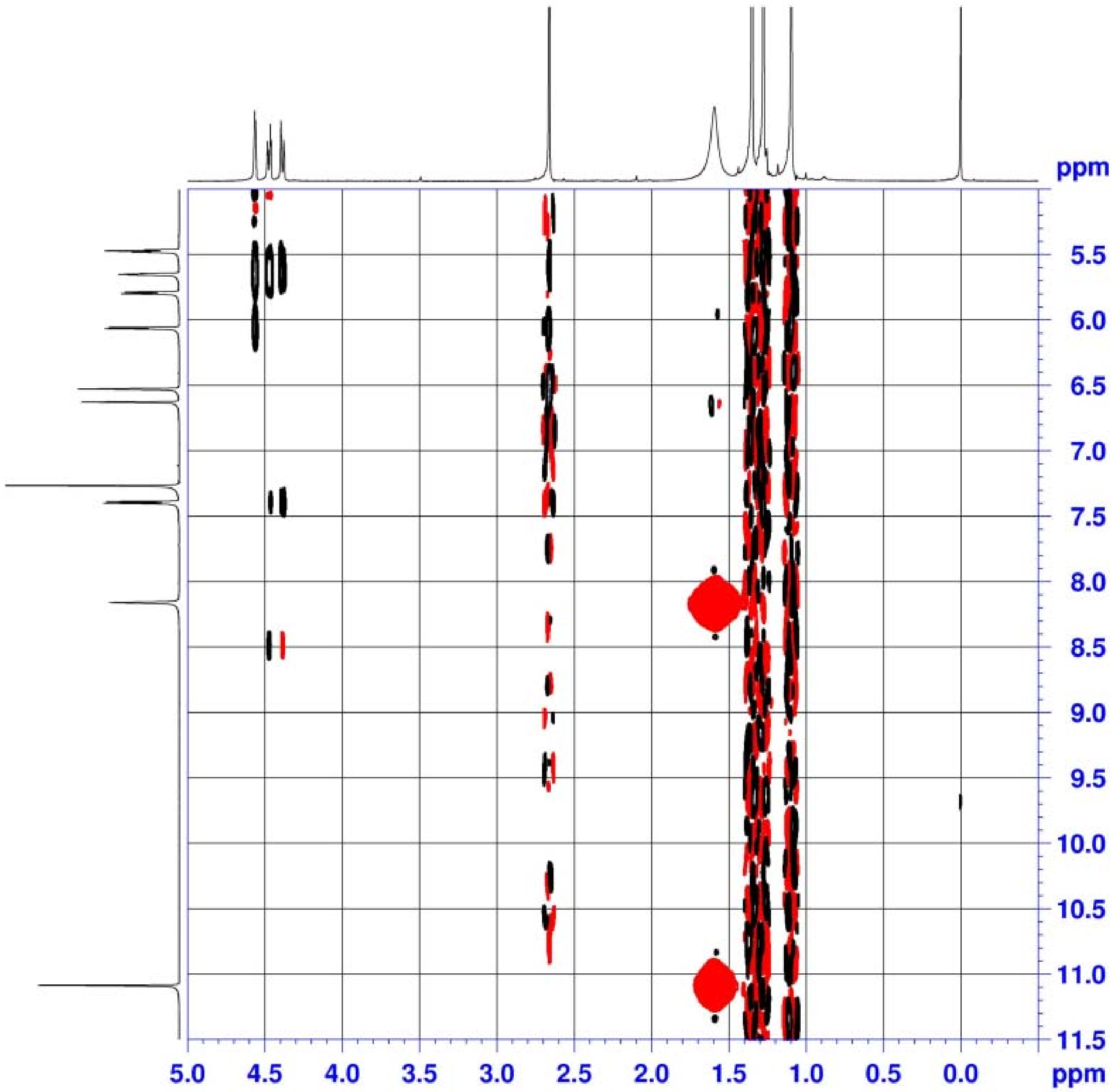

NOESY (700 MHz) spectrum of **4** in $CDCl_3$

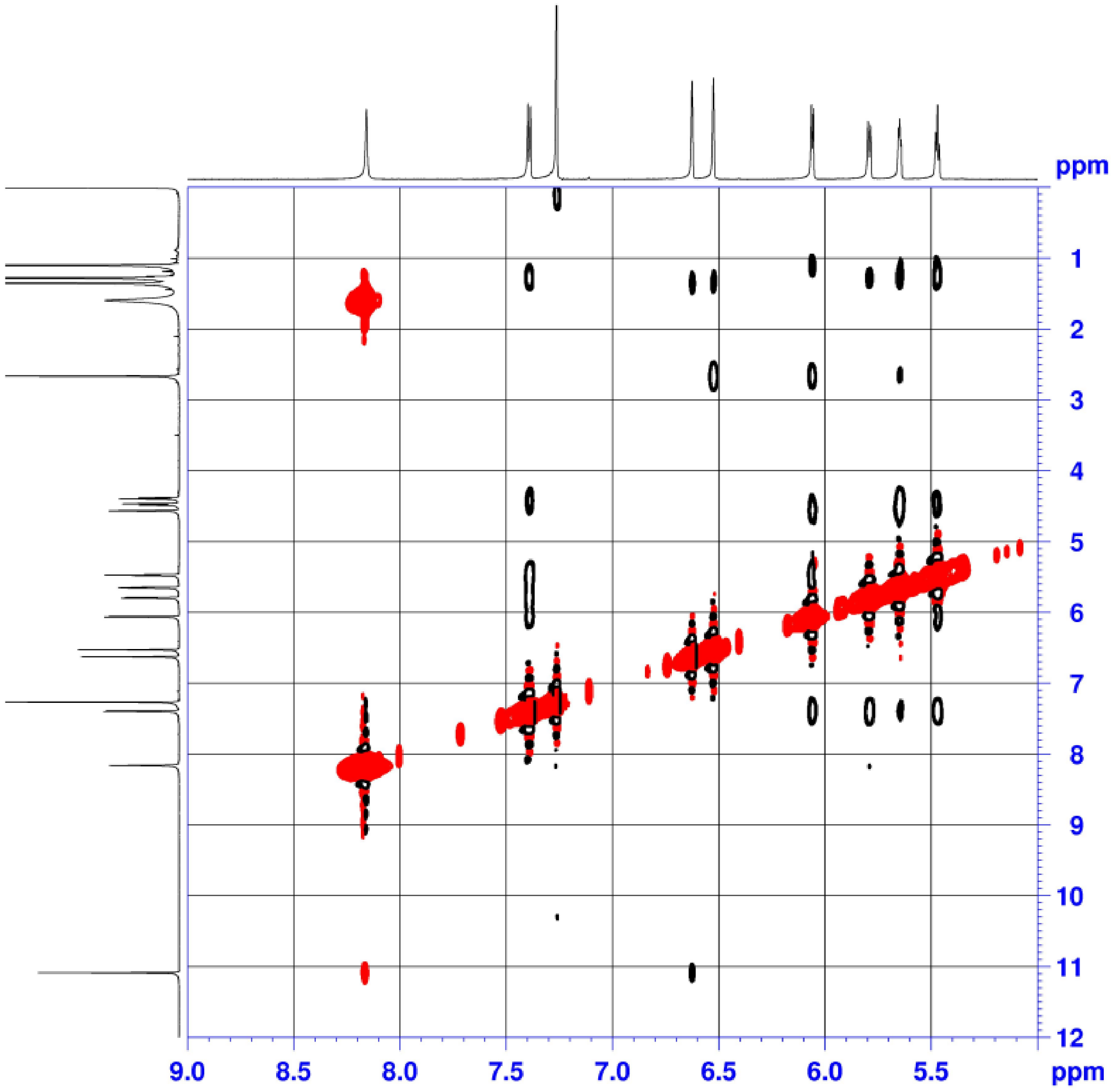

NOESY (700 MHz) spectrum of **4** in $CDCl_3$

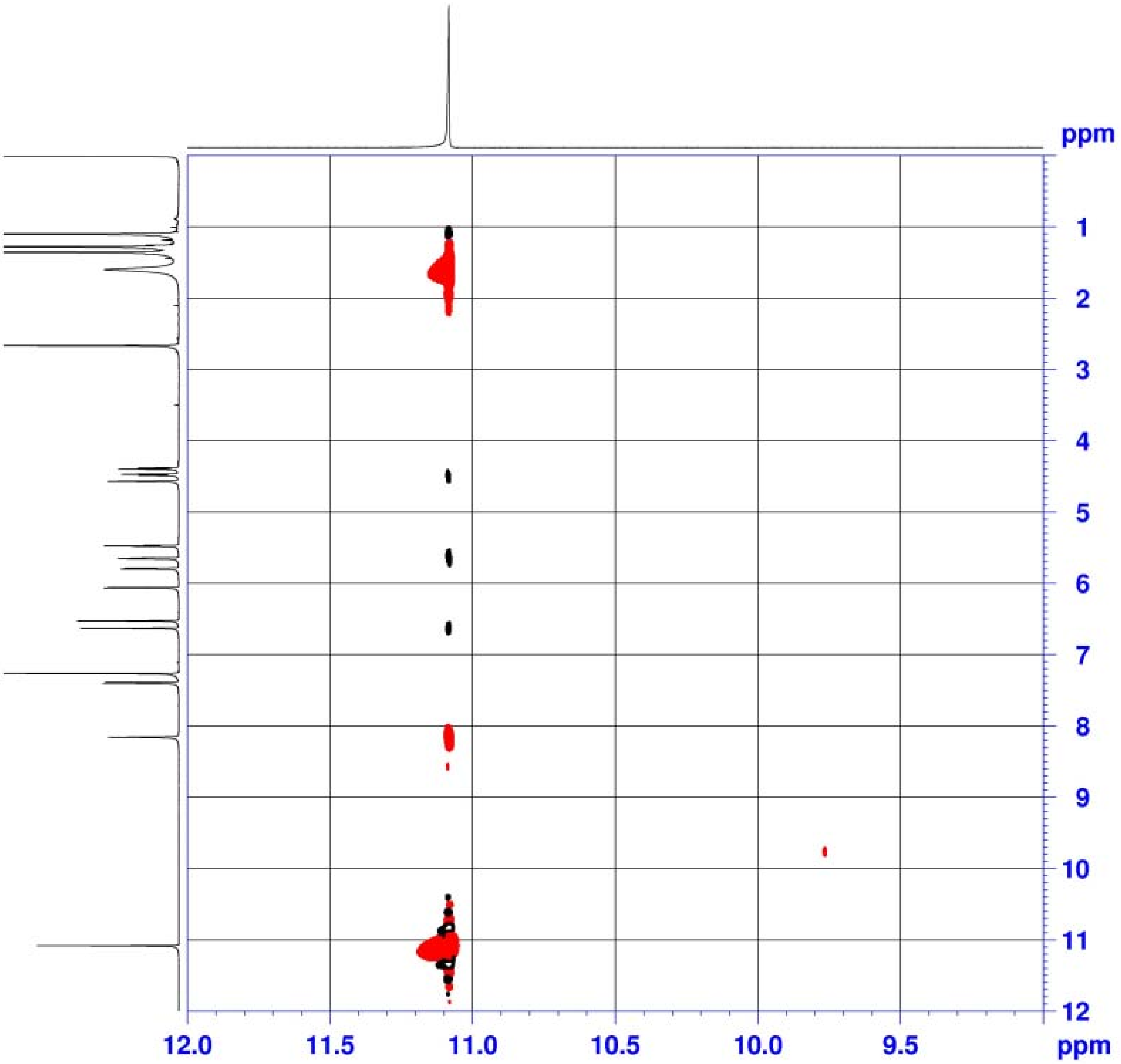

Figure S1. Energy analyses of conformers (14*S*,15*S*,17*R*)-2A to (14*S*,15*S*,17*R*)-2J

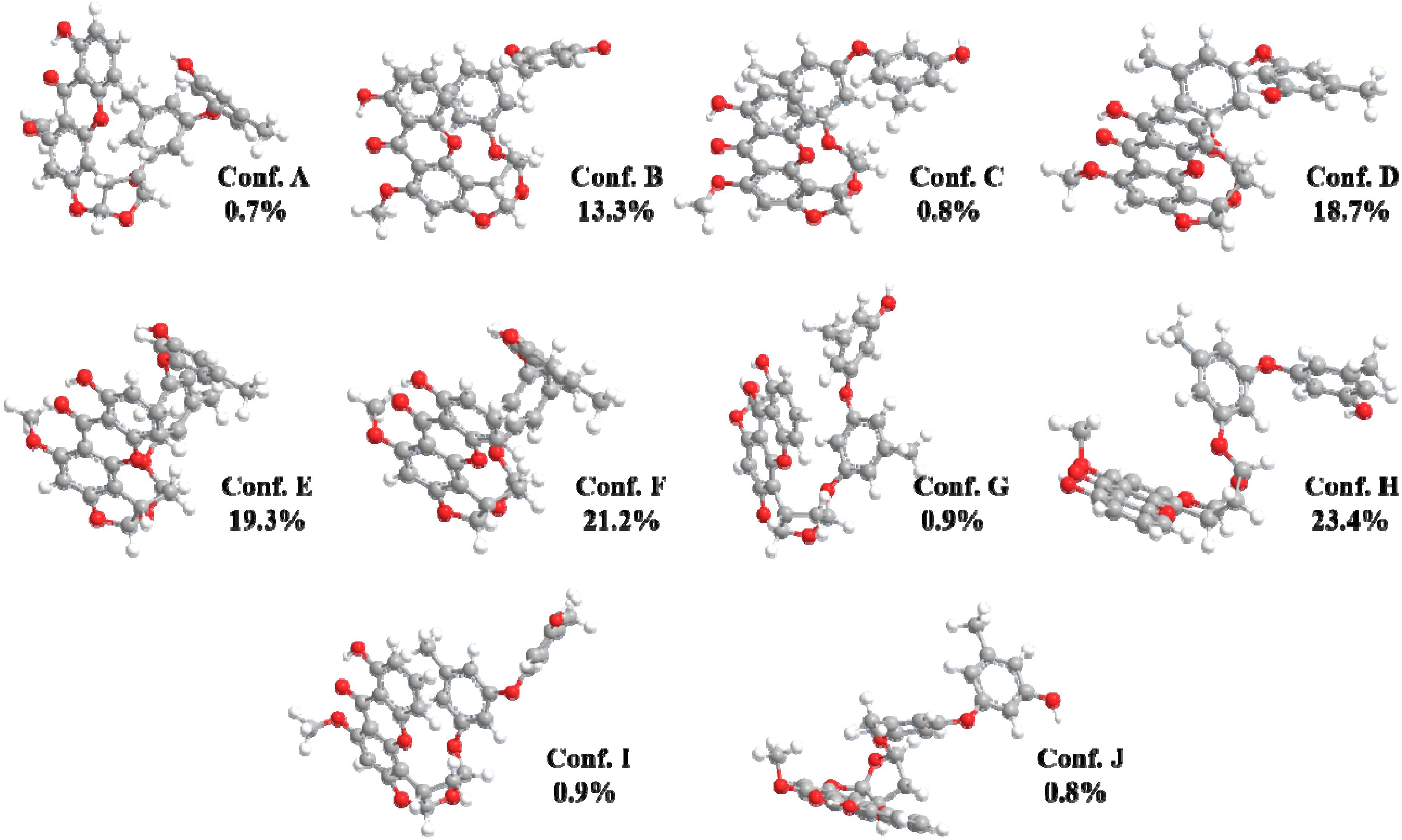

Figure S2. Energy analyses of conformers (15*R*,19*R*)-3A to (15*R*,19*R*)-3E

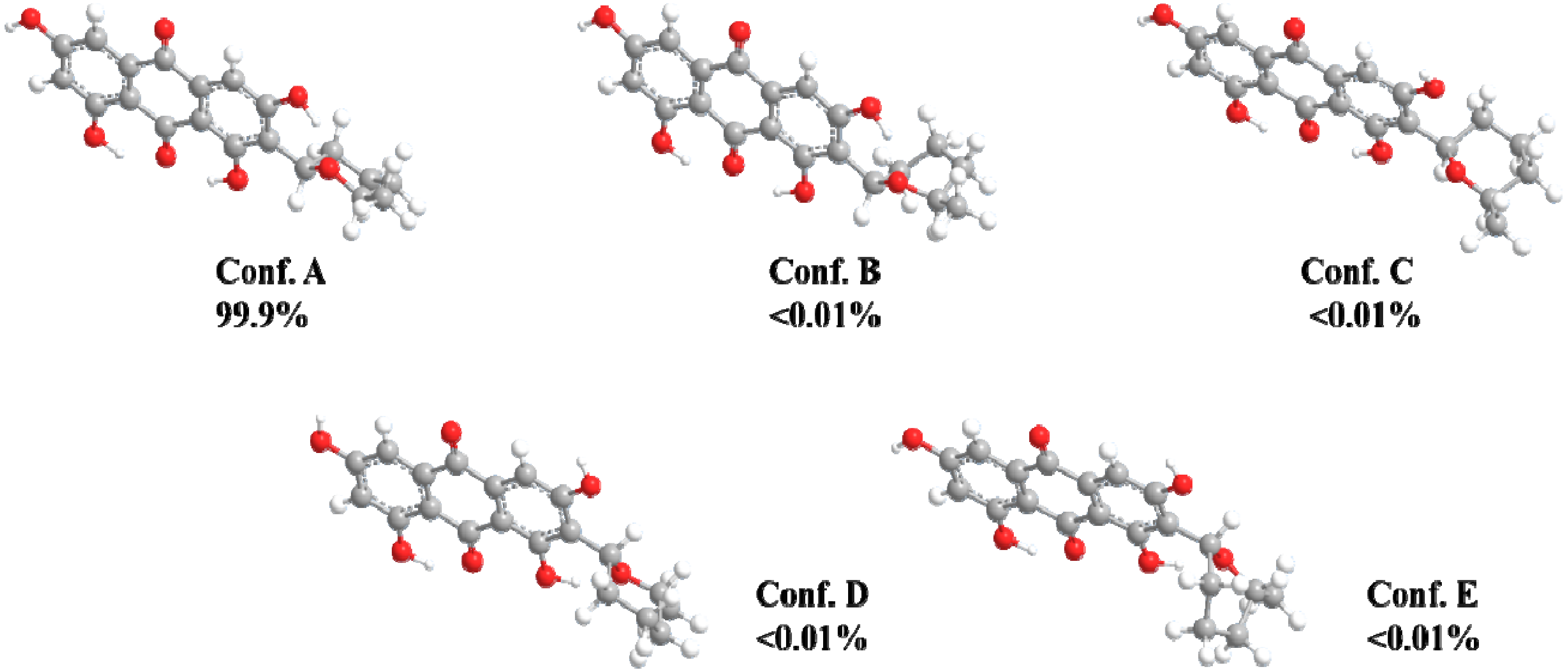

Figure S3. The UV spectrum of **1**

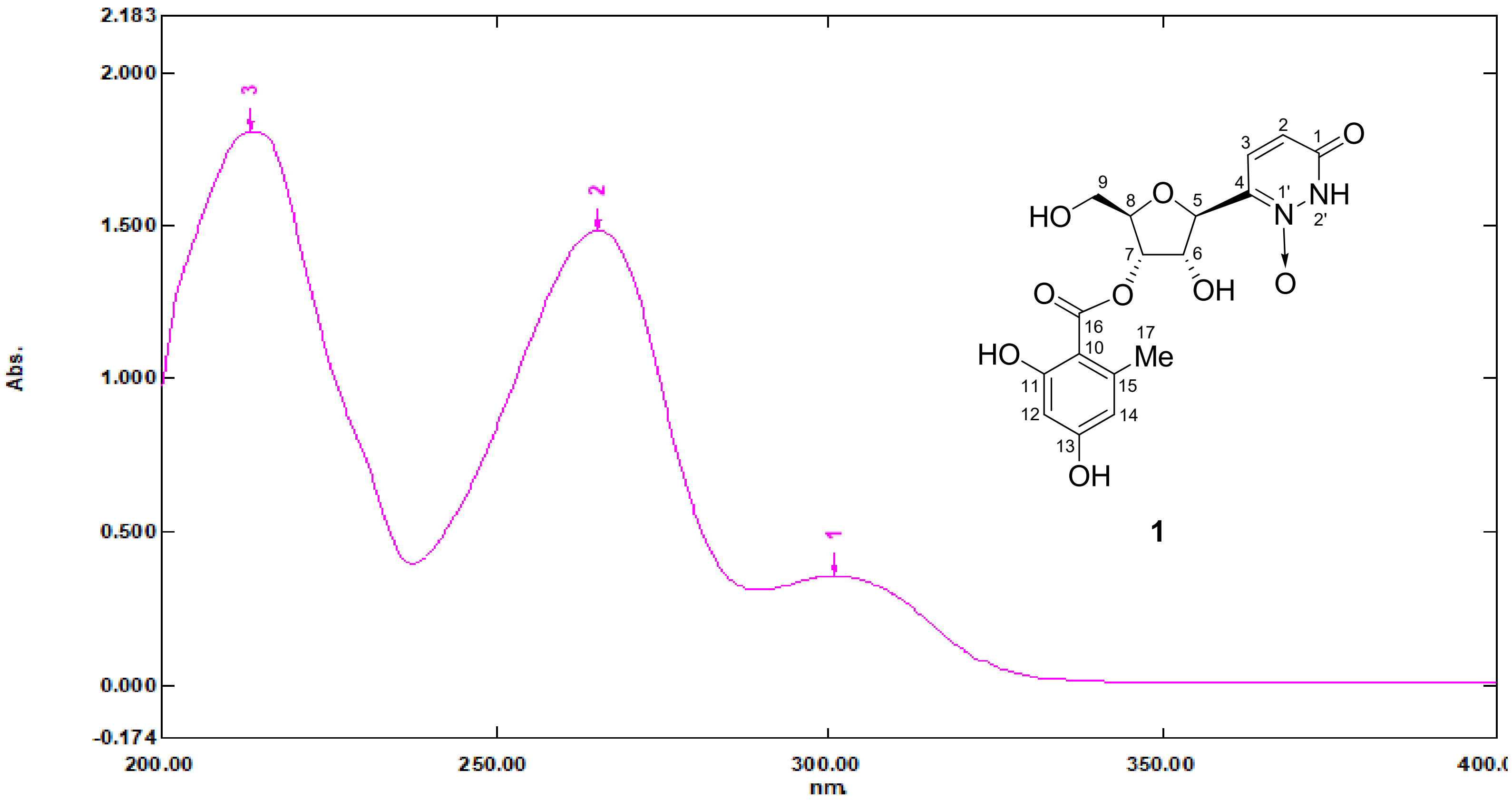

No.	P/V wavelength (nm)	Abs.
1	300.80	0.355
2	265.40	1.482
3	213.40	1.805

Figure S4. The UV spectrum of **2**

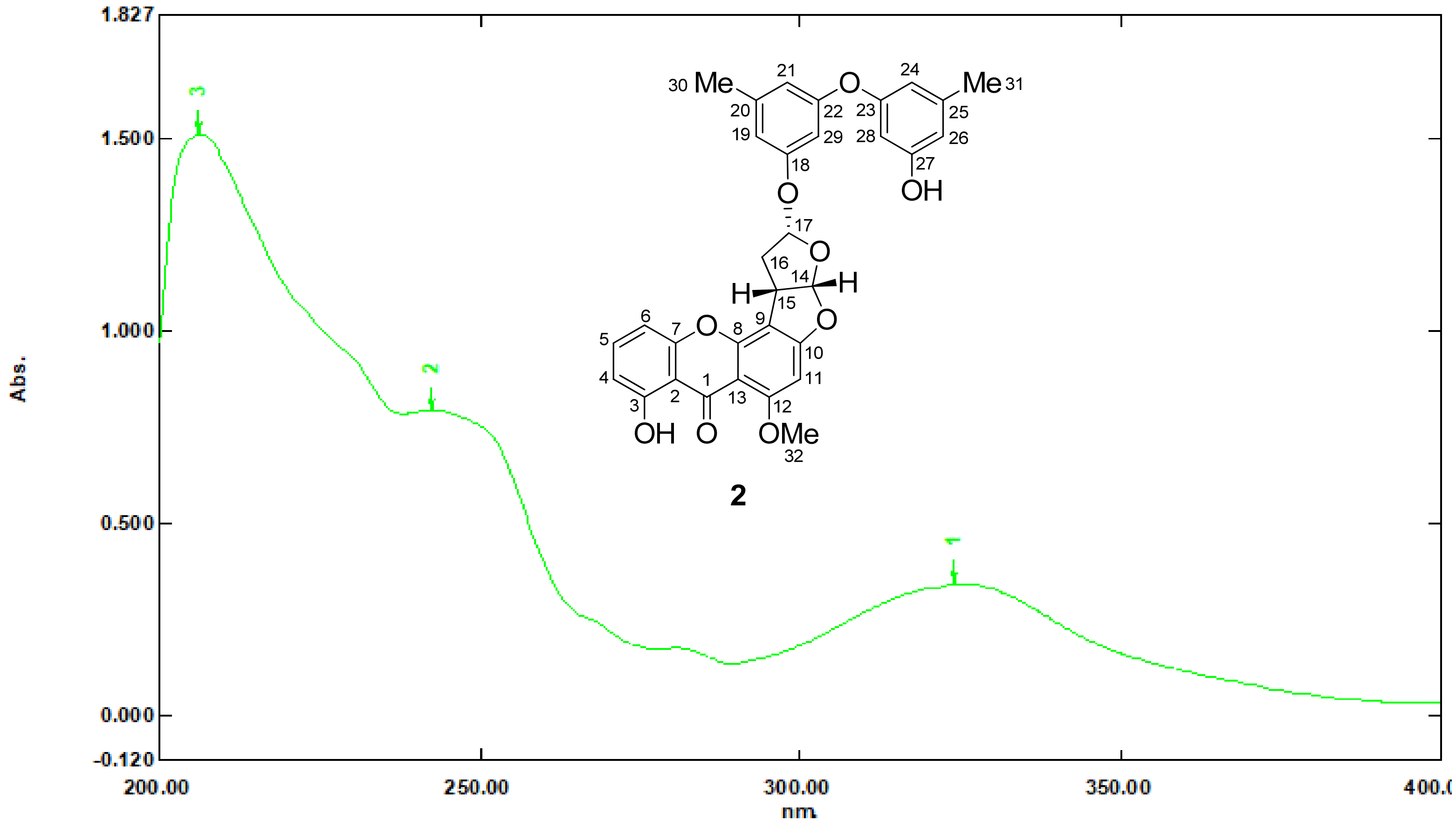

No.	P/V wavelength (nm)	Abs.
1	324.20	0.343
2	242.60	0.794
3	206.20	1.515

Figure S5. The UV spectrum of **3**

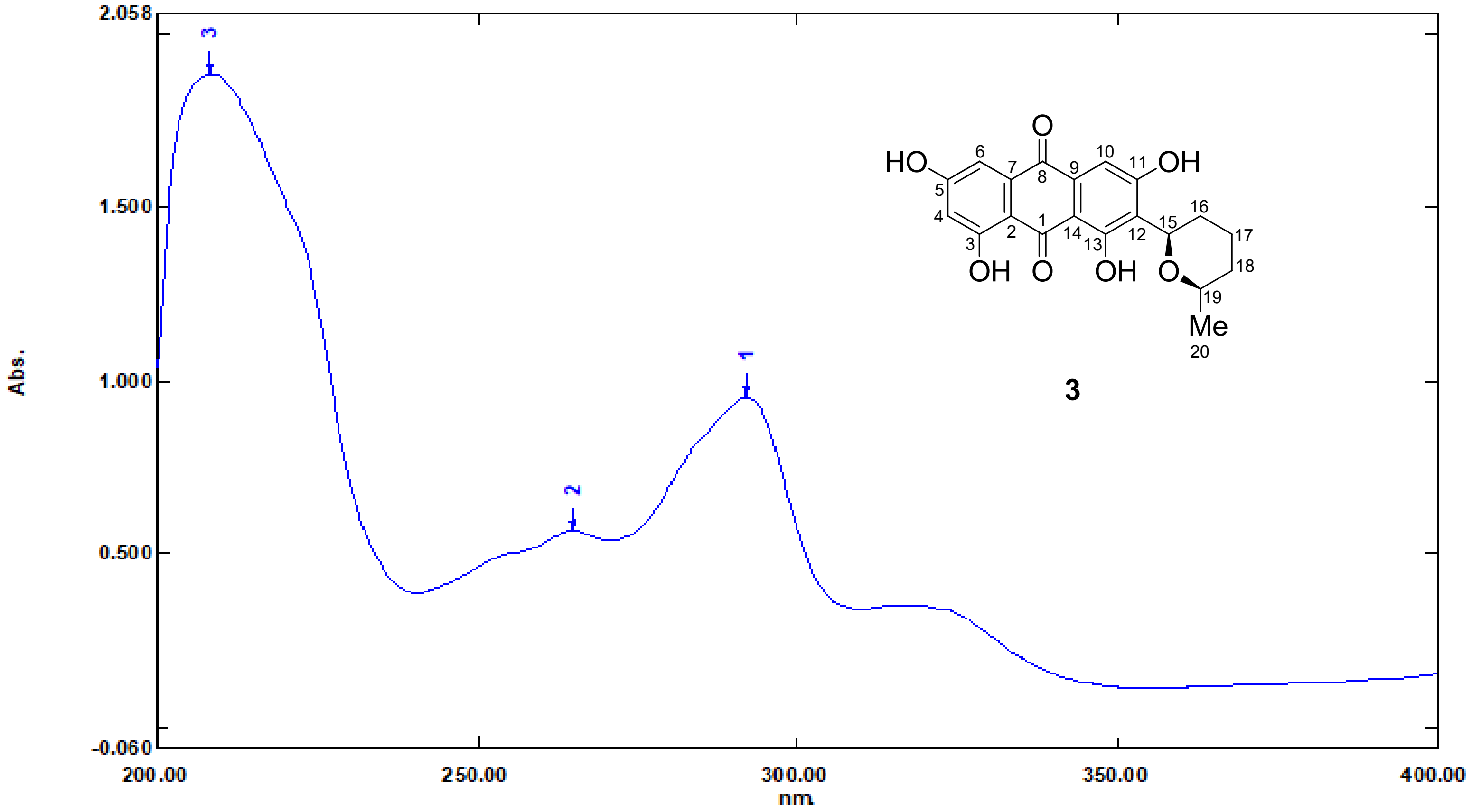

No.	P/V wavelength (nm)	Abs.
1	292.00	0.951
2	264.80	0.564
3	208.20	1.882

Figure S6. The IR spectrum of **1**

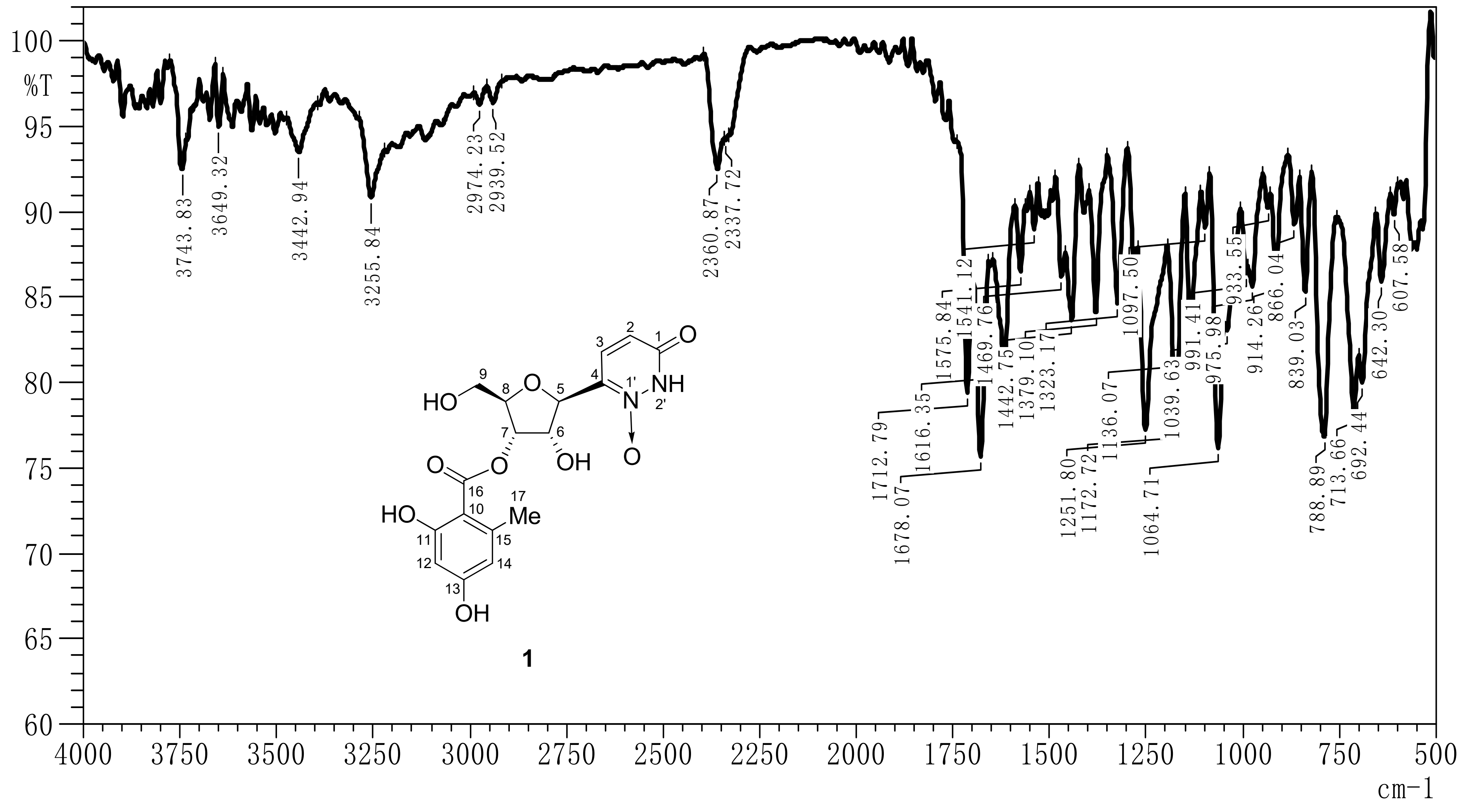

Figure S7. The IR spectrum of **2**

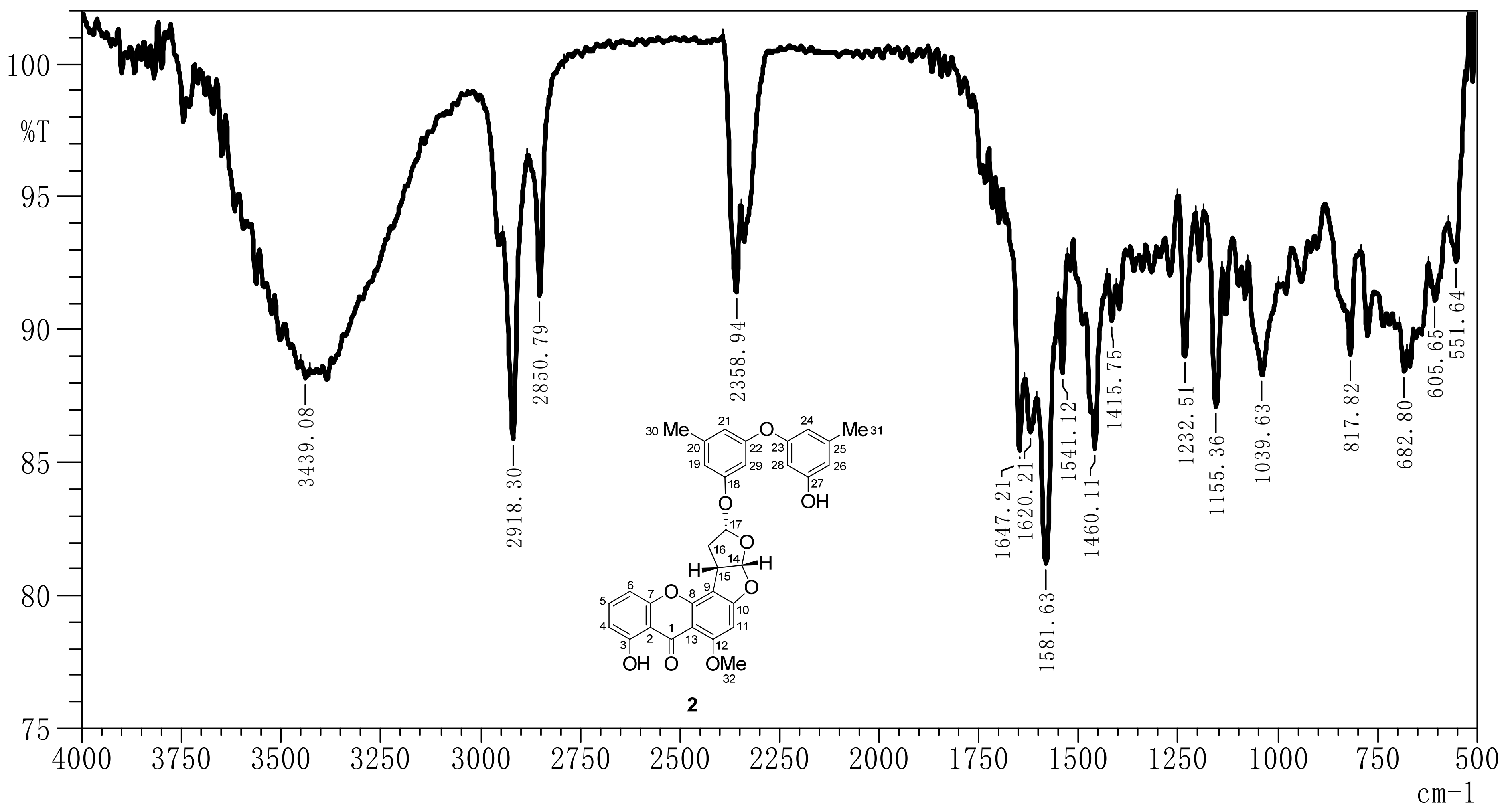

Figure S8. The IR spectrum of **3**

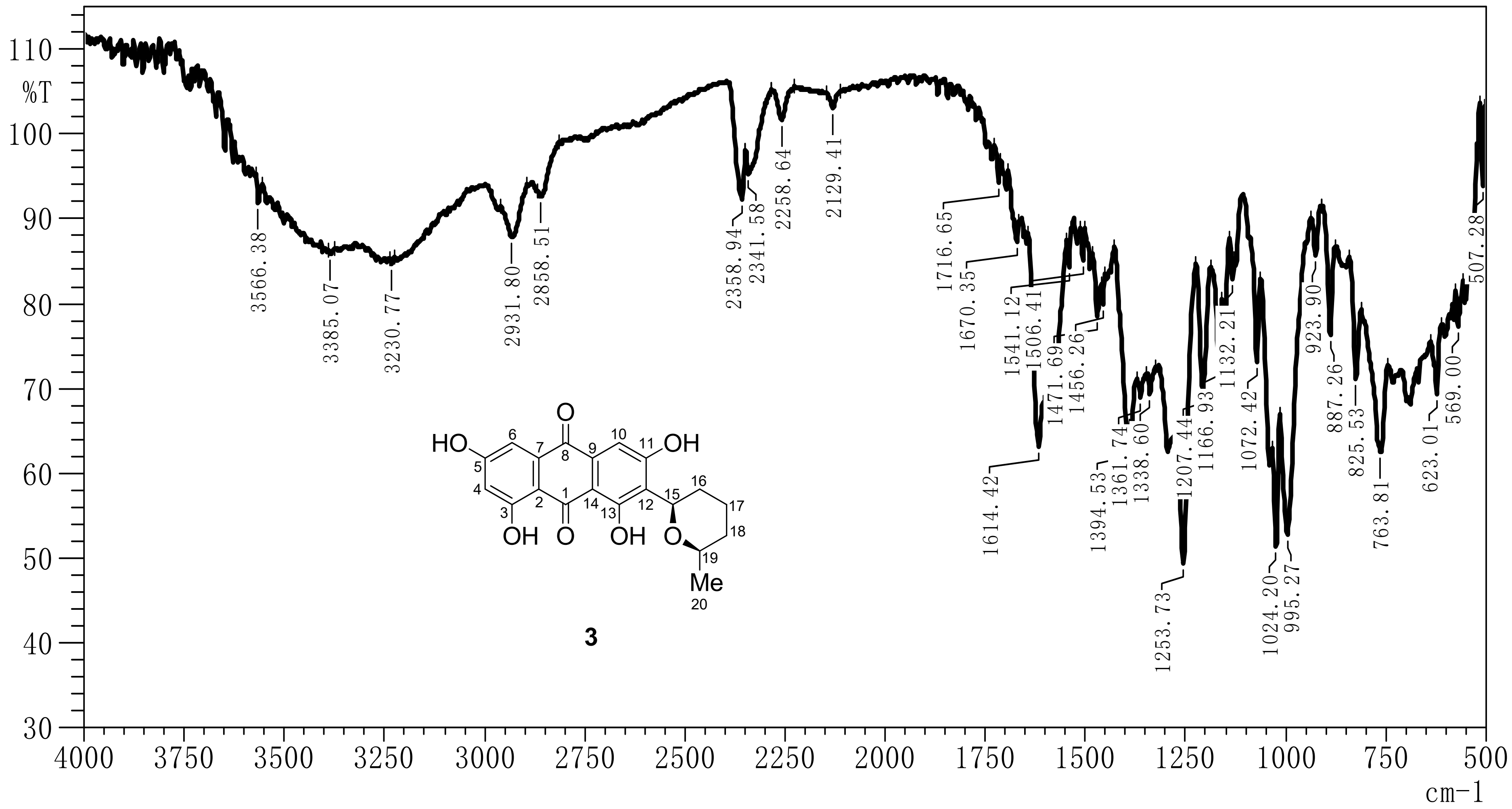

Supplement: Supplementary file 1 [file marinedrugs-21-00598-s001.zip › marinedrugs-2702683-supplementary.pdf]
